# Supplementary material for: Maternal BMI at the start of pregnancy and offspring epigenome-wide DNA methylation: findings from the pregnancy and childhood epigenetics (PACE) consortium
Source: Hum Mol Genet. 2017 Jul 21;26(20):4067–85. doi: 10.1093/hmg/ddx290 (PMC5656174; doi:10.1093/hmg/ddx290)
Supplement: Supplementary File 2 [file file_s2_ddx290.docx]

# Cohort-specific information (alphabetical order)

## Cohort-specific methods

**ALSPAC**

Design and study population

ALSPAC is a large, prospective cohort study based in the South West of England. 14,541 pregnant women resident in Avon, UK with expected dates of delivery 1st April 1991 to 31st December 1992 were recruited and detailed information has been collected on these women and their offspring at regular intervals. The study website contains details of all the data that is available through a fully searchable data dictionary (http://www.bris.ac.uk/alspac/researchers/data-access/data-dictionary/).

Written informed consent has been obtained for all ALSPAC participants. Ethical approval for the study was obtained from the ALSPAC Ethics and Law Committee and the Local Research Ethics Committees.

Maternal and paternal BMI

Maternal pre-pregnancy weight and height were self-reported, and paternal weight and height were reported by the mother. Data for partners who were not confirmed as being the biological father of the child by the mothers’ report were excluded. In order to explore the possibility that systematic bias in partner or self-report weight (e.g. those who are heavier systematically under-reporting their weight) might bias our findings, we used Bland-Altman plots (plots of mean and difference in mean), which suggested that the level of misreporting is similar for the majority of participants and is not markedly influenced by mean weight[1].

Methylation measurements

Cord blood was collected according to standard procedures, spun and frozen at -80˚C. DNA methylation analysis and data pre-processing were performed at the University of Bristol as part of the ARIES project (ariesepigenomics.org.uk). Following extraction, DNA was bisulfite converted using the Zymo EZ DNA MethylationTM kit (Zymo, Irvine, CA). Following conversion, the genome-wide methylation status of over 485,000 CpG sites was measured using the Illumina Infinium® HumanMethylation450k BeadChip assay according to the standard protocol. The arrays were scanned using an Illumina iScan and initial quality review was assessed using GenomeStudio (version 2011.1). The level of methylation is expressed as a “Beta” value (β-value), ranging from 0 (no cytosine methylation) to 1 (complete cytosine methylation). Samples from all time-points in ARIES were distributed across slides using a semi-random approach (sampling criteria were in place to ensure that all time-points were represented on each array) to minimize the possibility of confounding by batch effects. Samples failing quality control (average probe detection p-value ≥ 0.01) were repeated. As an additional quality control step genotype probes on the HumanMethylation450k were compared between samples from the same individual and against SNP-chip data to identify and remove any sample mismatches. Data were pre-processed in R (version 3.0.1) with the WateRmelon package according to the subset quantile normalization approach described by Touleimat & Tost in an attempt to reduce the non-biological differences between probes.

We removed probes that had a detection P-value >0.05 for >5% of samples (3034 probes), probes on the X or Y chromosomes and SNPs (rs probes). 471192 probes remained.

Covariates

Maternal age at delivery was derived from the mother’s report of her own and her baby’s dates of birth. Maternal social class was classified for this study as “attended university” or “did not attend university”. Parity was extracted from medical records and categorized for this study as nulliparous or parous; maternal smoking behaviour was assessed during pregnancy via questionnaire and categorised for this study as 1) never smoking during pregnancy, 2) any smoking during pregnancy. Five surrogate variables were generated and included in models to adjust for technical batch. Estimation of six different white blood cell types (CD8+ T and CD4+ T lymphocytes, CD56+ natural killer cells, CD19+ B cells, CD14+ monocytes, and granulocytes) by Houseman method[15] was performed using the default implementation of the *estimateCellCounts* function in the minfi package[16].

**BAMSE**

Design and study population

BAMSE is a prospective population-based cohort study of children recruited at birth and followed during childhood and adolescence. Details of the study design, inclusion criteria, enrolment and data collection are described elsewhere[2]. In short, 4,089 children born between 1994 and 1996 in four municipalities of Stockholm County were enrolled. At baseline, when the infant was approximately 2 months of age, parents completed a questionnaire that assessed residential characteristics, as well as socioeconomic and lifestyle factors. When children were 1, 2, 4, 8, 12 and 16 years, the parents completed questionnaires focusing on children’s symptoms related to wheezing and allergic diseases, as well as various exposures. The survey response rates were 96%, 94%, 91%, 84%, 82% and 78%, respectively[3]. Furthermore, blood was obtained at ages 4, 8 and 16 years from 2,605 (63.7%), 2,470 (60.4%) and 2,547 (62.2%) children, respectively. The baseline and follow-up studies were approved by the Regional Ethical Review Board, Karolinska Institutet, Stockholm, Sweden, and the parents of all participating children provided informed consent.

Maternal BMI

Maternal BMI was obtained through linkage to the Swedish Medical Birth Register containing information on weight and height recorded at the first visit to the antenatal-care clinic (usually around week 10 in pregnancy[3]. BMI was calculated as body weight in kilograms divided by height in metres squared (kg/m2).

Methylation measurements

Epigenome-wide DNA methylation was measured in 269 children (Caucasians only), using DNA extracted from blood samples collected at the age of 16 years. An aliquot (500 ng) of DNA per sample underwent bisulfite conversion using the EZ-96 DNA Methylation kit (Zymo Research Corporation, Irvine, USA). Samples were plated onto 96-well plates in randomized order. Samples were processed with the Illumina Infinium HumanMethylation450 BeadChip (Illumina Inc., San Diego, USA).

A series of steps were completed for quality control and data analysis. First, we implemented sample filtering to remove bad quality and mixed up samples. Samples were excluded in case of sample call rate <99% in 0.3% of the probes, colour balance >3, low staining efficiency, poor extension efficiency, poor hybridization performance, low stripping efficiency after extension and poor bisulfite conversion. We also applied multidimensional scaling (MDS) plot to evaluate gender outliers based on chromosome X data, that produced two separated clusters for male and female. Furthermore, we applied median intensity plot for methylated and unmethylated intensity by using the minfi R package. This QC steps led to exclusion of two samples. Second, 65 SNPs assays, the probes on sex chromosomes, the probes that mapped on multi-loci, and the probes containing SNPs at the target CpG sites with a MAF>5% were excluded. The multi-loci probes and probes containing SNPs were selected based on published data [4], resulting in the exclusion of 47,654 probes, leaving a total of 437,858 probes in the analysis.

Finally, we implemented “DASEN” recommended from watermelon package to do signal correction and normalization [5]. The Empirical Bayes method via ComBat was applied for batch correction using the sva package in R [6]. Out of 267 available DNA samples, 221 with complete data on maternal BMI, covariates and methylation profiles could be included in the present analyses.

Covariates

Data on maternal age, educational level and parity were collected from the baseline questionnaire (at birth of the child). Maternal smoking during pregnancy was assessed by questionnaires distributed when the children were around one year of age. Estimation of six different white blood cell types (CD8+ T and CD4+ T lymphocytes, CD56+ natural killer cells, CD19+ B cells, CD14+ monocytes, and granulocytes) by Houseman method[15] was performed using the default implementation of the *estimateCellCounts* function in the minfi package[16].

**CBC (Hispanic and White)**

The California Department of Public Health maintains a repository of neonatal birth bloods as blood dried on a filter paper (Guthrie card). These are available for qualified researchers to perform specified health research as monitored by local and State level institutional review boards. Our current research project using CBC resources is a case-control study of childhood leukemia, the California Childhood Leukemia Study (CCLS) which identifies children with leukemia and matched controls (birthdate, gender, and ethnicity) from around the State of California [7]. Parents were interviewed to provide data on pre-pregnancy, pregnancy, and early life exposures in the topics of diet, smoking, radiation, pesticides, infections, and other variables of interest for cancer exposure. Parents provided consent to obtain birth bloods, which were used for DNA methylation analyses.

Approximately 300-500 ng of high molecular weight DNA was extracted from a 1/4 section of a 1.5 cm^2^ archived neonatal DBS (stored at -20°C from the time of birth) using Qiagen blood card extraction protocol and bisulfite treated using the EZ DNA Methylation-Direct™ Kit (Zymo). Genome-wide DNA methylation was then measured in these bisulfite converted DNA samples using Illumina^(C)^ Infinium HumanMethylation450 BeadChip arrays. CpG sites with detection p-values > 0.01 were defined as bad CpG sites and discarded. CpG sites with >15% of absence of information (i.e. >15% of total samples) were totally excluded from the analysis. A total of 540 CpGs were excluded. Samples with >15% of bad CpG sites (of the 450K loci) were also excluded from the analysis. The DNA methylation data preprocessing consisted of functional normalization according to Fortin et al. [8] to control for batch and position effects. Additional correction for probe types was accomplished with BMIQ normalization [9]. Joo *et al.* have demonstrated that DNA methylation measured by the HM450k array on archived dried blood spots is fully correlated with DNA methylation measured by the same platform on same individuals’ matched frozen buffy coats (correlation coefficient = 0.99), therefore proving that this material is suitable for DNA methylation analyses [10]. Estimation of six different white blood cell types (CD8+ T and CD4+ T lymphocytes, CD56+ natural killer cells, CD19+ B cells, CD14+ monocytes, and granulocytes) by Houseman method[15] was performed using the default implementation of the *estimateCellCounts* function in the minfi package[16].

**CHAMACOS**

Design and study population

The Center for the Health Assessment of Mothers and Children of Salinas (CHAMACOS) study is a longitudinal birth cohort study of the effects of exposure to pesticides and environmental chemicals on the health and development of Mexican-American children living in the agricultural region of Salinas Valley, CA. Detailed description of the CHAMACOS cohort has previously been published[11,12]. Briefly, 601 pregnant women were enrolled in 1999-2000 at community clinics and 527 liveborn singletons were born. Follow up visits occurred at regular intervals throughout childhood. Study protocols were approved by the University of California, Berkeley Committee for Protection of Human Subjects and written informed consent was obtained from all mothers.

Maternal and paternal BMI

Structured interviews by bilingual, bicultural study staff were conducted with participants twice during pregnancy: at a baseline visit (~13 weeks gestation) and follow up interview (~26 weeks gestation). Maternal pre-pregnancy and paternal BMI was calculated using interview-reported weight and height.

Methylation measurements

DNA methylation was measured in DNA isolated from the cord blood of 378 CHAMACOS newborns by Illumina Infinium HumanMethylation450 (450K) BeadChips. DNA samples were bisulfite converted using Zymo Bisulfite Conversion Kits (Zymo Research, Irvine, CA), whole genome amplified, enzymatically fragmented, purified, and applied to the 450K BeadChips (Illumina, San Diego, CA) according to manufacturer protocol. 450K BeadChips were handled by robotics and analyzed using the Illumina Hi-Scan system. DNA methylation was measured at 485,512 CpG sites.

Probe signal intensities were extracted by Illumina GenomeStudio software (version XXV2011.1, Methylation Module 1.9) methylation module and background subtracted. QA/QC was performed systematically by assessment of assay repeatability batch effects using 38 technical replicates. Quality was also ensured by only retaining samples where 95% of sites assayed had detection P> 0.01. The same threshold (95% detection at p>0.01) was imposed on CpGs as well (n= 460 removed). Sites with annotated probe SNPs and with common SNPs (minor allele frequency >5%) within 50bp of the target identified in the MXL (Mexican ancestry in Los Angeles, California) HapMap population were excluded from analysis (n=49,748). This left a total of 435,369 CpGs in the analysis. Color channel bias, batch effects and difference in Infinium chemistry were minimized by application of ASMN algorithm[13], followed by BMIQ normalization[14].

Covariates

Parity and maternal education were assessed by participant interview at baseline visit. Parity was coded as a binary variable, with 0 and ≥1 as the two categories. We used maternal education level as a proxy for maternal social class. This was a categorical variable with three levels: no more than a 6^th^ grade education, 6^th^ grade education but not high school graduate, and high school graduate.

Information on maternal smoking status was obtained by participant interviews at baseline, follow-up, and delivery visits. Subjects were grouped into three categories: 1) sustained smokers if they indicated they had smoked since baseline during either follow up interview, 2) smoker who’d quit if smoking was reported at baseline but not at follow up interview or delivery, 3) not smokers if they never reported smoking during pregnancy. Analysis was also adjusted for batch effects by including 450K plate (n=10) as additional covariates.

Estimation of six different white blood cell types (CD8+ T and CD4+ T lymphocytes, CD56+ natural killer cells, CD19+ B cells, CD14+ monocytes, and granulocytes) by Houseman method[15] was performed using the default implementation of the *estimateCellCounts* function in the minfi package[16].

**EARLI**

Design and study sample

The Early Autism Risk Longitudinal Investigation (EARLI) is an enriched risk prospective pregnancy cohort to study autism etiology [17]. The EARLI study was reviewed and approved by Human Subjects Institutional Review Boards (IRBs) from each of the four study sites (Johns Hopkins University, Drexel University, University of California Davis, and Kaiser Permanente Northern California). This longitudinal study recruited mothers of confirmed ASD children who were early in a subsequent pregnancy or were trying to become pregnant. There were 232 mothers with a subsequent sibling born through this study. All children were born between November 2009 and March 2012. Demographics, maternal behaviors, food frequency, medical history were all collected via questionnaire. Biosamples and house samples were collected during pregnancy, at birth, and during development.

Maternal BMI measures

Mothers were asked early in pregnancy to report their prepregnancy weight. Mothers were also asked their height without shoes. BMI was calculated from these measurements (kg/m^2^).

Methylation measures

Biospecimens including cord blood and placenta were collected and archived at 213 births. Cord blood DNA was extracted using the DNA Midi kit (Qiagen, Valencia, CA) and samples were bisulfite treated and cleaned using the EZ DNA methylation gold kit (Zymo Research, Irvine, CA). DNA was plated randomly and assayed on the Infinium HumanMethylation450 BeadChip (Illumina, San Diego, CA) at the Johns Hopkins SNP Center, a shared lab and informatics operation with the Center for Inherited Disease Research (Johns Hopkins University). Methylation control gradients and between-plate repeated tissue controls were used.

We used the minfi library (version 1.18.2) in R (version 3.3) to process raw Illumina image files into noob background corrected methylation values [18,19]. Probes with failed detection P-value (>0.05) in >10% of samples were removed (n=508). Samples with discordant methylation predicted sex and observed sex were removed (n=2) as were samples that appeared as outliers on the first principal component of methylation data across the genome prior to normalization (n=2). We adjusted normalized data for batch effects using ComBat in the sva package (version 3.9.1) [20]. Estimation of six different white blood cell types (CD8+ T and CD4+ T lymphocytes, CD56+ natural killer cells, CD19+ B cells, CD14+ monocytes, and granulocytes) by Houseman method[15] was performed using the default implementation of the *estimateCellCounts* function in the minfi package[16].

**GECKO**

Design and study population

The Groningen Expert Center for Kids with Obesity (GECKO) Drenthe cohort is a population-based prospective birth cohort study in Drenthe, a northern province in the Netherlands. All mothers of babies born between April 2006 and April 2007 were invited to participate during the third trimester of pregnancy. Of all 4,778 infants born in this period, a total of 2,874 children (60%) participated in the study and are followed until adulthood. This study has been approved by the Medical Ethical Committee of the University Medical Center Groningen and parents of all participants gave written informed consent. Details about this cohort have been described elsewhere (PMID 18238823).

Maternal and paternal BMI

Maternal (and paternal) weight and height were self-reported, this information was derived from the questionnaires during the third trimester of pregnancy. BMI (kg/m^2^) was calculated from these measurements.

Covariates

Data on maternal age, educational level, parity and smoking were self-reported in questionnaires during early pregnancy.

Methylation measurements

Within the GECKO Drenthe birth cohort we selected 258 infants for the methylation study: 129 exposed to maternal smoking during pregnancy and 129 unexposed to both maternal and paternal smoking during pregnancy. From these 258 infants, we used DNA which was extracted from cord blood for the epigenome-wide DNA methylation analyses. To limit batch effects, we randomized all samples on gender and smoking status. Samples (500 ng per sample) were placed on three 96-well plates. Bisulfite conversion was performed using the EZ-96 DNA methylation kit (Zymo research Corporation, Irvine, USA). Then, we processed the samples with the Infinium HumanMethylation450 BeadChip (Illumina Inc., San Diego, USA). We used minfi to calculate betas and p values for all 485,577 CpGs. During the quality control, we excluded two males that clustered in the female group, based on X chromosome betas. We performed Illumina-suggested background normalization, colour correction and Subset-quantile Within Array Normalization (SWAN). We excluded one sample because it did not meet the criteria of ≥99% of the CpGs with detection p value <0.05. This resulted in 129 exposed and 126 unexposed children. We excluded control probes, probes on X or Y chromosomes and probes that did not meet our criteria of a detection p value of <0.05 in ≥99% of the samples, resulting in 465,891 remaining CpGs.

Cell type correction

In GECKO we used the Reinius-based Houseman method [15,21] in the minfi package[18] in R[22] to calculate relative proportions of six white blood cell subtypes (CD4+ T-lymphocytes, CD8+ T-lymphocytes, NK (natural killer) cells, B-lymphocytes, monocytes and granulocytes).

**Gen3G**

Design and study population

Gen3G (Genetics of Glycemic regulation in Gestation and Growth) is a prospective observational cohort study aiming to increase our understanding of biological, environmental, and genetic determinants of glucose regulation during pregnancy and their impact on fetal development and was described in details previously [23]. In brief, we recruited a total of 1034 pregnant women between January 2010 and June 2013 representing the general population of women in reproductive age receiving care at our institution. Women were excluded if they had non-singleton pregnancy, known pre-pregnancy diabetes or overt diabetes diagnosed based on biochemical screening that we performed at first trimester. The study protocol was approved by the Centre Hospitalier Universitaire de Sherbrooke (CHUS) ethic committee board and every participant gave written informed consent before enrolment in the study, in accordance with the Declaration of Helsinki.

Maternal BMI

At first trimester visit (between 5 and 16 weeks of gestation), trained research staff performed anthropometric measurements according to standardized procedures: height (in m) was measured with a wall stadiometer without shoes. Pre-pregnancy weight was self-reported. BMI was calculated as weight divided by squared height (kg/m^2^).

Methylation measurements

Among our overall population, we randomly selected 182 mother-child dyads with complete maternal and neonatal data and bio-samples, including cord blood samples. DNA samples were isolated using the Gentra Puregene Blood Kit (Qiagen, Mississauga, ON, Canada). DNA was quantified on a Beckman Coulter DTX 880 spectrophotometer using the Quant-iT™ PicoGreen® dsDNA assay kit (Life Technologies (Invitrogen), Burlington, ON, Canada) following the manufacturer’s standard procedure for a high-range standard curve. We used HumanMethylation450 BeadChips (Illumina, Inc., San Diego, CA, USA) to measure DNAm levels across the genome. We removed outliers (based on multidimensional scaling plot), sex mismatch, and samples with more than 5% of missing values across the epigenome (detection *P* value > 0.01). After quality control, DNAm levels from HumanMethylation450 BeadChips were available in 176 cord blood samples. At the end, 170 participants were included in the BMI continuous analysis after exclusion of missing data in covariates.

Covariates

Research staff collected demographic characteristics (maternal age), medical and obstetric history (parity), and lifestyle questionnaires (smoking) at the first trimester research visit. Maternal age is the age at the first trimester research visit. Parity is defined as the number of term pregnancies. Smoking was derived in three categories (1- no smoking in pregnancy, 2- stop in the beginning of pregnancy and 3- smoked during pregnancy) by comparison of the last menstrual period date and the smoking ending date (collected at first trimester). Five surrogate variables were generated and included in models to adjust for technical batch. Estimation of six different white blood cell types (CD8+ T and CD4+ T lymphocytes, CD56+ natural killer cells, CD19+ B cells, CD14+ monocytes, and granulocytes) by Houseman method[15] was performed using the default implementation of the *estimateCellCounts* function in the minfi package[16].

**Generation R (GENR)**

Design and study population

The Generation R Study is a population-based prospective cohort study from fetal life onwards in Rotterdam, the Netherlands, which has been described in detail elsewhere[24]. All included children were born between April 2002 and January 2006 and form a largely prenatally enrolled birth cohort that is being followed-up. A total of 9,778 mothers were included, most during pregnancy (response rate at birth 61%). The study has been approved by Medical Ethical Committee of Erasmus MC, University Medical Center Rotterdam and written consent was obtained for all participants.

Maternal and paternal BMI

Maternal and paternal weight and height were measured in early pregnancy without shoes and heavy clothing. BMI was calculated from these measurements (kg/m2).

Covariates

Data on maternal age, educational level and parity were collected by questionnaires in early pregnancy. Maternal smoking during pregnancy was assessed by questionnaires in each trimester of pregnancy.

Methylation measurements

DNA extracted (using the salting-out method) from cord blood from 979 European-ancestry children from the Generation R focus cohort was used for this analysis. 500 ng DNA per sample underwent bisulfite conversion using the EZ-96 DNA Methylation kit (Shallow) (Zymo Research Corporation, Irvine, USA). Samples were plated onto 96-well plates in no specific order. Samples were processed with the Illumina Infinium HumanMethylation450 BeadChip (Illumina Inc., San Diego, USA), which analyses methylation at 485,577 CpG sites.

Quality control of analyzed samples was performed using standardized criteria. Samples were excluded in case of low sample call rate (<99%, 6 samples excluded), colour balance >3 (no samples excluded), low staining efficiency (no samples excluded), poor extension efficiency (no samples excluded), poor hybridization performance (no samples excluded), low stripping efficiency after extension (no samples excluded) and poor bisulfite conversion (1 sample removed). In addition, 2 samples were excluded because of a gender mismatch and 1 sample was excluded because of a retracted informed consent, leaving a total of 969 Generation R samples in the analysis.

Probes with a single nucleotide polymorphism in the single base extension site with a frequency of > 1% in the GoNLv4 reference panel were excluded[25], as were probes with non-optimal binding (non-mapping or mapping multiple times to either the normal or the bisulphite-converted genome[26], resulting in the exclusion of 49,564 probes, leaving a total of 436,013 probes in the analysis.

We ran DASES normalization using a pipeline adapted from that developed by Touleimat and Tost[27]. DASES normalization includes background adjustment, between-array normalization applied to type I and type II probes separately, and dye bias correction applied to type I and type II probes separately and is based on the DASEN method described by Pidsley et al, but adds the dye bias correction, which is not included in DASEN[5].

Cell type correction

Cell type correction was applied using the reference-based Houseman method[15] in the minfi package[18]in R[22]. This method estimates the relative proportions of six white blood cell subtypes (CD4+ T-lymphocytes, CD8+ T-lymphocytes, NK (natural killer) cells, B-lymphocytes, monocytes and granulocytes), based on a standard reference population[21].

**GOYA**

Design and study population

The Genetics of Overweight Young Adults (GOYA) study is described previously by Paternoster et al. [28]. It is based on the Danish National Birth Cohort that included 92,000 pregnant women and their pregnancies during 1996-2002. Of 67,853 women who had given birth to a live born infant, had provided a blood sample during pregnancy and had BMI information available, 3.6% of these women with the largest residuals from the regression of BMI on age and parity (all entered as continuous variables) were selected for GOYA. The BMI for these 2451 women ranged from 32.6 to 64.4. From the remaining cohort a random sample of similar size (2450) was also selected. DNA methylation data were generated for the offspring of 1000 mothers in the GOYA study. I.e. “cases” had mothers with a BMI>32 and “controls” were sampled from the normal BMI distribution (can include mothers with a BMI>32).

Maternal and paternal BMI

Maternal pre-pregnancy BMI was calculated from self-reported height and weight collected during a telephone interview at ~16 weeks of gestation. Paternal weight and height were reported by the mother in a postpartum interview 18 months after birth and used to calculate paternal BMI. For continuous models, we selected a random sample of GOYA participants that gave a maternal BMI distribution similar to that seen in the whole population.

Methylation measurements

Cord blood was collected according to standard procedures, spun and frozen at -80˚C. DNA methylation analysis and data pre-processing were performed at the University of Bristol. Following extraction, DNA was bisulfite converted using the Zymo EZ DNA MethylationTM kit (Zymo, Irvine, CA). Following conversion, the genome-wide methylation status of over 485,000 CpG sites was measured using the Illumina Infinium® HumanMethylation450k BeadChip assay according to the standard protocol. The arrays were scanned using an Illumina iScan and initial quality review was assessed using GenomeStudio (version 2011.1). The level of methylation is expressed as a “Beta” value (β-value), ranging from 0 (no cytosine methylation) to 1 (complete cytosine methylation). Samples from all time-points in ARIES were distributed across slides using a semi-random approach (sampling criteria were in place to ensure that all time-points were represented on each array) to minimize the possibility of confounding by batch effects. Samples failing quality control (average probe detection p-value ≥ 0.01) were repeated. As an additional quality control step genotype probes on the HumanMethylation450k were compared between samples from the same individual and against SNP-chip data to identify and remove any sample mismatches. Data were normalized using the functional normalization approach in the Minfi R package.

We removed probes that had a detection P-value >0.05 for >5% of samples, probes on the X or Y chromosomes and SNPs (rs probes). 473864 probes remained.

Covariates

Data on covariates were collected via a telephone interview at around 16 weeks gestation. Maternal age was derived from the mother’s report of her own date of birth. Socioeconomic status was defined using maternal education or occupation: 1) manager/long or medium education, 2) work requiring a short training period, or skilled manual labour, 3) unskilled or public service. Parity was categorized for this study as nulliparous or parous. Maternal smoking in pregnancy was defined as any smoking in pregnancy or no smoking in pregnancy. Five surrogate variables were generated and included in models to adjust for technical batch. Estimation of six different white blood cell types (CD8+ T and CD4+ T lymphocytes, CD56+ natural killer cells, CD19+ B cells, CD14+ monocytes, and granulocytes) by Houseman method[15] was performed using the default implementation of the *estimateCellCounts* function in the minfi package[16].

**IOW Birth Cohort (IOW F1)**

Design and study population

A whole population birth cohort was established on the Isle of Wight, UK, in 1989 to prospectively study the natural history of allergic diseases from birth onwards. Both the Isle of Wight and the study population are 99% Caucasian. Ethics approvals were obtained from the Isle of Wight Local Research Ethics Committee (now named the National Research Ethics Service, NRES Committee South Central –Southampton B) at recruitment and for the 1, 2, 4, 10 and 18 years follow-up. Of the 1536 children born between January 1, 1989, and February 28, 1990, written informed consent was obtained from parents to enroll 1456 newborns. Children were followed up at the ages of 1 (n = 1167), 2 (n = 1174), 4 (n = 1218), 10 (n = 1373), and 18 years (n = 1313). Demographic information of parents and offspring, status of allergic diseases, phenotypic measures on allergic sensitization, IgE, and lung function, and environmental exposures, along with other phenotypic measures, were collected at birth and updated at each follow-up.

Maternal BMI

Maternal BMI was calculated from measured height and weight at the 1^st^ trimester.

Methylation measurements

For 367 aged 18 years subjects, we measured DNA methylation from whole blood processed with the Illumina Infinium HumanMethylation450 BeadChip (Illumina Inc., San Diego, USA).CPACOR [29] pipeline was used for QC and normalisation of the data. Methylation markers on 65 single nucleotide polymorphism (SNP) and sex chromosomes were removed. We applied Illumina background Correction to all intensity values. Any intensity values having detection p-values >= 10^-16^ were set as missing data. Samples with call rate < 98% were excluded. After the QC, 461,230 sites remain for the subsequent analysis. A quantile normalisation was applied using limma on intensity values separately based on six different probe-type categories (Type-I M red, Type-I U red, Type-I M green, Type-I U green, Type-II red, and Type-II green). Beta values were then calculated from these normalised intensity values.

Covariates

Covariates were collected via questionnaires collected at recruitment, before and during pregnancy. Maternal age was derived from mothers’ date of birth. In case of maternal BMI, we used BMI at 1^st^ trimester. Maternal smoking status and parity were collected from the responses from the questionnaires. Maternal smoking status in pregnancy (Yes/No) was defined as any smoking in pregnancy or no smoking in pregnancy. Socioeconomic status was defined using maternal socio-economic cluster information (high, low, low-low, low-mid, and mid). Indicator of different batches that DNA methylation data were generated were included as a covariate to adjust for batch effect. Estimation of six different white blood cell types (CD8+ T and CD4+ T lymphocytes, CD56+ natural killer cells, CD19+ B cells, CD14+ monocytes, and granulocytes) by Houseman method[15] was performed using the default implementation of the *estimateCellCounts* function in the minfi package[16].

**IOW 3^rd^ generation cohort (IOW F2)**

Design and study population

The recruitment of newborns started from April 2010. Data used in the analyses were from infants born between April 201 to May 2014. In total, 200 newborns were recruited such that at least one of their parents is in the IOW birth cohort (IOW F1) and the recruitment is ongoing. For each infant, along with other phenotypic information such as gender and birth weight, status of wheezing and eczema was recorded, measures of wheal size from skin prick test as well as IgE were recorded.

Maternal BMI

Maternal BMI was calculated from measured height and weight at recruitment in the 1^st^ trimester.

Methylation measurements

We measured epigenome-wide DNA methylation of 53 new borns using DNA extracted from cord blood. One thousand ng DNA per sample underwent bisulfite conversion using the EZ-96 DNA Methylation kit (Shallow) (Zymo Research Corporation, Irvine, USA). Samples were plated onto 96-well plates in random order. Samples were processed with the Illumina Infinium HumanMethylation450 BeadChip (Illumina Inc., San Diego, USA). Quality control of analyzed samples was performed using standardized criteria. All samples are over 99.8% probes detected. Samples were deleted if more than 75% CpG sites of that sample with detection P-value larger than or equal to 10^-5^, CpG sites were deleted if 10% of the samples with detection P-value larger than or equal to 0.01. CpG sites with missing values caused by insufficient copies of a probe binding to the sample DNA were excluded from the study. Also, DNA methylation from the 53 subjects were measured in four batches. After excluding missing values in each batch, in total 358, 214 CpG sites were remained for subsequent studies. Data were pre-processed using IMA package in R including quantile normalization and type I and type II probe peak correction. The R package ComBat built upon an empirical Bayes framework was used to remove batch effects. Beta-values were calculated for all CpG sites.

Covariates

Data on covariates were collected via questionnaires collected at recruitment during the 1^st^ trimester of pregnancy, including maternal age, socioeconomic status, smoking status during pregnancy, and parity. Maternal age was derived from the mother’s report of her own date of birth. Socioeconomic status was defined using maternal education: 1) left before general certificate of secondary education, 2) completed education at 16 years, 3) completed education at 18 years, and 4) Other, e.g., vocational training . Subjects who completed education at 16 years or above were included into group 1. Maternal smoking status in pregnancy (Yes/No) was defined as any smoking in pregnancy or no smoking in pregnancy. Indicator of different batches that DNA methylation data were generated were included as a covariate to adjust for batch effect. Estimation of six different white blood cell types (CD8+ T and CD4+ T lymphocytes, CD56+ natural killer cells, CD19+ B cells, CD14+ monocytes, and granulocytes) by Houseman method[15] was performed using the default implementation of the *estimateCellCounts* function in the minfi package[16].

**MeDALL**

Two cohorts participating in The Mechanisms of the Development of ALLergy (MeDALL) consortium were pooled for this analysis: INMA from Spain and EDEN from France. Information about this consortium is available through <http://consortiapedia.fastercures.org/consortia/medall/>

**MeDALL INMA**

Design and study population

INMA (Childhood and Environment) The INMA—INfancia y Medio Ambiente—(Environment and Childhood) Project is a network of birth cohorts in Spain that aim to study the role of environmental pollutants in air, water and diet during pregnancy and early childhood in relation to child growth and development [30]. More information about the INMA project is available through our webpage <http://www.proyectoinma.org/>

Maternal and paternal BMI

Maternal pre-pregnancy BMI was calculated from measured height and self-reported weight collected using a questionnaire at enrolment (week 12 of pregnancy).[31] Reported pre-pregnancy weight was highly correlated with measured weight at 12 weeks of pregnancy in INMA (r= 0.96; P < 0.0001). Paternal weight and height were reported by the mother at this time and used to calculate paternal BMI.

Methylation measurements

A total of 178 children with maternal BMI information and cord blood DNA methylation information were included. Blood at birth was obtained in EDTA tubes and extracted using the Chemagic DNA Blood Kits (Perkin Elmer) in a Chemagen Magnetic Separation Module 1 station at the Spanish National Genotyping Center (CEGEN, http://www.usc.es/cegen/). As part of the MeDALL consortium some of the samples underwent a precipitation-based concentration and purification using GlycoBlue (Ambion) to uniform the concentration and purity between cohorts. DNA concentration was determined by Nanodrop measurement and picogreen quantification. After normalization of the concentration, the samples were randomized to reduce batch effects. Standard male and female DNA samples were included in this step as internal controls. 500 ng of DNA of each sample were bisulfite- converted using the EZ 96-DNA methylation kit following the manufacturer’s standard protocol. After verification of the bisulfite conversion using Sanger Sequencing, the DNA methylation was measured using the Illumina Infinium HumanMethylation450 BeadChip. Idat files were preprocessed imported using minfi. The preprocessing steps are summarized below: 1) sample filtering to remove bad quality and mixed up samples, 2) 65 SNPs probes, the probes on sex chromosomes, potential cross reactive probes, and probes containing SNPs at the target CpG sites with a MAF>10% were excluded (cross reactive and polymorphic probes according to the list of Chen et al) [4]. A total of 439,306 CpG probes were retained for downstream analyses, 3) Signal correction and normalization was performed using “DASEN” (wateRmelon).

Covariates

Information on parity, maternal age and maternal education was collected by questionnaire at enrolment (week 12 of pregnancy). Maternal age was used as a continuous covariate. Parity was categorized into 0 or ≥1. Maternal socioeconomic status was based on maternal occupation at pregnancy, it was categorized into three levels: low (levels V/VI semi-skilled/unskilled occupations), medium (levels III/IV skilled manual/non-manual) or high (managers/technicians). Pregnant women were asked whether they were current smokers (at week 32 of pregnancy) and if so, how much. They were also asked if they had stopped smoking due to pregnancy and when (before pregnancy or at what month of pregnancy). Any smoking was defined as smoking any number of cigarettes at any time during pregnancy. Estimation of six different white blood cell types (CD8+ T and CD4+ T lymphocytes, CD56+ natural killer cells, CD19+ B cells, CD14+ monocytes, and granulocytes) by Houseman method[15] was performed using the default implementation of the *estimateCellCounts* function in the minfi package[16].

**MeDALL EDEN**

Design and study population

The EDEN (Etude des Déterminants pré et post natals du développement et de la santé de l′Enfant) study is a prospective Birth Cohort Study (https://eden.vjf.inserm.fr/), which has been described in detail elsewhere[32]. Pregnant women seen for a prenatal visit at the departments of Obstetrics and Gynecology of the University Hospital of Nancy and Poitiers before their twenty-fourth week of amenorrhea were invited to participate. Enrolment started in February 2003 in Poitiers and September 2003 in Nancy; it lasted 27 months in each centre. Among eligible women, 55% (2002 women) accepted to participate. The study has been approved by the ethical committees Comité Consultatif pour la Protection des Personnes dans la Recherche Biomédicale, Le Kremlin-Bicêtre University hospital, and Commission Nationale de l’Informatique et des Libertés.

Maternal and paternal BMI

Maternal pre-pregnancy BMI and paternal current BMI were calculated from self-reported height and weight collected using a questionnaire at enrolment (between 24–28 week gestation).

Methylation measurements

DNA has been extracted from 1367 cord blood samples and 836 blood samples in 5–6-year-old children; 682 children had DNA both from cord blood and at 5–6 years. Amplified and genomic DNA samples are now stored in 96-well plates at -80°C. More than 40 single nucleotide polymorphisms (SNPs) have been genotyped either from genomic or from amplified DNA. The samples underwent bisulfite treatment using the EZ-96 DNA Methylation kit (Zymo Research Corporation, Irvine, USA), and were subsequently processed with the Illumina Infinium Human Methylation 450 BeadChip (Illumina Inc., San Diego, USA). In total, 439,306 CpGs are available in children with DNA measurements.

Covariates

Maternal age was used as a continuous variable. Parity was categorized into 0 or ≥1. Maternal smoking was categorised as follow: no smoking in pregnancy; smoking, but stopped in early pregnancy; and smoking throughout pregnancy. Maternal socioeconomic status was categorized into three levels according to the educational level, which referred to the highest diploma obtained (low level: less than high school, medium level: high school diploma to some university (at least a 2-year university degree) and high level: university degree (at least a 3-year university degree). These information were obtained by interview at enrolment (between 24–28 week gestation). Estimation of six different white blood cell types (CD8+ T and CD4+ T lymphocytes, CD56+ natural killer cells, CD19+ B cells, CD14+ monocytes, and granulocytes) by Houseman method[15] was performed using the default implementation of the *estimateCellCounts* function in the minfi package[16].

**MoBa1, 2, and 3**

Materials and Methods

Participants represent three subsets of mother-offspring pairs from the national Norwegian Mother and Child Cohort Study (MoBa) [33–35]. The years of birth for MoBa participants ranged from 1999-2009. MoBa mothers provided written informed consent. Each subset is referred to here as MoBa1, MoBa2, and MoBa3. MoBa1 is a subset of a larger study within MoBa that included a cohort random sample and cases of asthma at age three years[36]. We previously reported an association between maternal smoking during pregnancy and differential DNA methylation in MoBa1 newborns[37]. We subsequently measured DNA methylation in additional newborns (MoBa2) in the same laboratory (Illumina, San Diego, CA)[38]. MoBa2 included cohort random sample plus cases of asthma at age seven years and nonasthmatic controls. MoBa3 was designed to evaluate the association between differential cord blood DNA methylation and later childhood cancer status. Methylation measurements for MoBa3 were made at the International Agency for Research on Cancer (IARC) (6). Years of birth were 2002-2004 for children in MoBa1, 2000-2005 for MoBa2, and 2000-2008 for MoBa3. All three studies were approved by the Regional Committee for Ethics in Medical Research, Norway. In addition, MoBa1 and MoBa2 were approved by the Institutional Review Board of the National Institute of Environmental Health Sciences, USA.

Details of the DNA methylation measurements and quality control for the MoBa1 participants were previously described [37] and the same protocol was implemented for the MoBa2 participants. Briefly, umbilical cord blood samples were collected and frozen at birth at -80**°**C. All biological material was obtained from the Biobank of the MoBa study [34]. Bisulfite conversion was performed using the EZ-96 DNA Methylation kit (Zymo Research Corporation, Irvine, CA) and DNA methylation was measured at 485577 CpGs in cord blood using Illumina’s Infinium HumanMethylation450 BeadChip[39] . Raw intensity (.idat) files were handled in R using the *minfi* package19 to calculate the methylation level at each CpG as the beta-value (β=intensity of the methylated allele (M)/(intensity of the unmethylated allele (U) + intensity of the methylated allele (M) + 100)) and the data was exported for quality control and processing. Probe and sample-specific quality control was performed in the MoBa1, MoBa2, and MoBa3 datasets separately. Similar protocols were applied to MoBa1 and Moba2, as follows: Control probes (N=65) and probes on X (N=11 230) and Y (N=416) chromosomes were excluded in both datasets. Remaining CpGs missing > 10% of methylation data were also removed (N=20 in MoBa1, none in MoBa2). Samples indicated by Illumina to have failed or have an average detection p value across all probes < 0.05 (N=49 MoBa1, N=35 MoBa2) and samples with gender mismatch (N=13 MoBa1, N=8 MoBa2) were also removed. For MoBa1 and MoBa2, we accounted for the two different probe designs by applying the intra-array normalization strategy Beta Mixture Quantile dilation (BMIQ)[14]. The Empirical Bayes method via *ComBat* was applied separately in each dataset for batch correction using the *sva* package in *R* [6]*.*After quality control exclusions, the sample sizes were 1,068 for MoBa1 and 685 for MoBa2.

For MoBa3, bisulfite conversion and methylation measurements were done at the International Agency for Research on Cancer (Lyon, France). Similar data quality control and processing was applied with some slight differences. Methylation features were filtered from (i) cross-reactive probes, (ii) probes mapping to sex chromosomes and (iii) probes overlapping with a known single nucleotide polymorphism (SNP) with an allele frequency of at least 5% in the overall population (all ethnic groups), resulting in the exclusion of 36 231 probes. Data quality was further assessed using box plots for the distribution of methylated and unmethylated signals, and multidimensional scaling plots and unsupervised clustering were used to check for sample outliers. After background correction and color-bias adjustment, type I and type II probe distributions were aligned using the intra-array BMIQ [6] from the watermelon package. Batch effects were corrected by surrogate variable analysis (SVA)[40]. After quality control, the sample size for MoBa3 was 253.

Maternal BMI

Maternal pre-pregnancy BMI was assessed by maternal self-report of pre-pregnancy height and weight in MoBa questionnaire one distributed to all participating women around gestational week 17 for all three datasets.

Covariates

For all three datasets, information on maternal age, parity, maternal education and smoking was collected via questionnaires completed by the mother or from birth registry records as previously described (4). Maternal age was included as a continuous variable. Parity was categorized as 0, or ≥ 1 births. Maternal educational level was categorized into four groups based on years of education: less than high school/secondary school, high school/secondary school completion, some college or university, or 4 years of college/university or more. Maternal smoking status during pregnancy was classified into three groups: non-smoker, stopped smoking in early pregnancy, and smoked throughout pregnancy. Estimation of six different white blood cell types (CD8+ T and CD4+ T lymphocytes, CD56+ natural killer cells, CD19+ B cells, CD14+ monocytes, and granulocytes) by Houseman method[15] was performed using the default implementation of the *estimateCellCounts* function in the minfi package[16].

The current analyses include the children who had cord blood DNA methylation measurements, BMI and covariate data (N=995 from MoBa1; N=647 from MoBa2; N=231 from MoBa3), and each dataset was analyzed independently.

**NEST**

Design and study population

The Newborn Epigenetics STudy (NEST) is a multiethnic birth cohort designed to identify the effects of early exposures on epigenetic profiles and phenotypic outcomes. Pregnant women were recruited from prenatal clinics serving Duke University Hospital and Durham Regional Hospital Obstetrics facilities in Durham, North Carolina from April 2005 to July 2009. Gestational age at enrollment ranged from 6 to 42 weeks (median 30 weeks). Eligibility criteria were women aged 18 years or older, English speaking, pregnant, and an intention to use one of the two obstetrics facilities. Among these, women infected with HIV or intending to give up custody of the offspring of index pregnancy were excluded. Current smokers were targeted for the first ~200 participants. Of the 1,101 women who met eligibility criteria and were approached, 895 (81%) were enrolled and umbilical cord blood was collected from 741 infants. The current analysis was limited to the 384 infants with 450k and covariate data; this includes 23 children from multiple birth pregnancies. This study was approved by the Duke Institutional Review Board.

Maternal and paternal BMI

Maternal pre-pregnancy weight and height were self-reported by the mother as part of a standardized questionnaire completed at enrolment. BMI was calculated as pre-pregnancy weight in kilograms divided by height in square meters (kg/m^2^).

Covariates

Covariates considered as potential confounders were, maternal age at delivery (continuous), educational attainment, smoking status, and parity. Maternal age at delivery was calculated from the maternal date of birth and the date of delivery. Maternal educational attainment was reported by the mom on the baseline questionnaire with a response to the following question: “What is the highest grade or year of school you have completed?” (Less than high school, high school graduate/GED, some college, college graduate, or graduate education). Four questions were used to ascertain smoking status. Women were first asked if they ever smoked and whether they were current smokers by responding to questions “Have you ever smoked 100 cigarettes or more in your lifetime?” (Yes/ No), followed by, “Do you smoke now?” (Yes/No). To determine the timing of cigarette smoking exposure to the offspring, women who reported being smokers were then asked to respond to the question, “Did you smoke anytime in the year before you found out you were pregnant?” (Yes/ No). Women also responded to the question “After you found out you were pregnant, which of the following best describes your behavior?” The four possible responses were, “I continue to smoke,” or “I stopped during the first/second/third trimester.” From these responses, three categories of maternal cigarette smoking were created as follows: (a) “smokers during pregnancy” were women who reported having ever smoked 100 cigarettes or more and smoking past early pregnancy; (b) “quitters during pregnancy” were women who reported having ever smoked 100 cigarettes or more, smoking during the year of pregnancy, and stopping smoking during early pregnancy; (c) “non-smokers during pregnancy” were women who reported never smoking during the pregnancy. Parity was obtained from medical records after delivery and categorized as 0 or >= 1. Estimation of six different white blood cell types (CD8+ T and CD4+ T lymphocytes, CD56+ natural killer cells, CD19+ B cells, CD14+ monocytes, and granulocytes) by Houseman method[15] was performed using the default implementation of the *estimateCellCounts* function in the minfi package[16].

Methylation measurements

Genomic DNA from buffy coat specimens was extracted from umbilical cord blood using Puregene Reagents (Qiagen, Valencia, CA). Bisulfite conversion was performed using the EZ-96 DNA Methylation Kit (Zymo Research Corporation) and DNA methylation was measured at 485,577 CpGs using Illumina’s Infinium Human- Methylation450 BeadChip. Illumina’s GenomeStudio Methylation module version 1.0 (Illumina Inc.) was used to calculate the methylation level at each CpG as the beta value. The RnBeads package in R was used to load and normalize the Illumina idat ﬁles and to conduct basic QC analyses. Normalization used the SWAN algorithm as implemented in the RnBeads R package. Quality control procedures included removed of probes on X (N=11,230) and Y (N=416) chromosomes. Samples indicated by Illumina to have failed were also removed.

**NFCS**

Design and study population

Participants involved in the analysis are infants born in Norway from Norway Facial Clefts Study (NCL). The analysis included 418 infants with orofacial clefts and 480 controls randomly selected among all live births in Norway. All infants were born between year 1996 to 2001. The left-over portion of the infant`s heel stick blood samples collected for PKU testing were used to profile methylation level.

Maternal and paternal BMI

Maternal weight and height were measured during early pregnancy visit. BMI was calculated from these measurements (kg/m2).

Methylation measurements

Genomic DNA had been extracted from blood clot using automated equipment (Autopure LS, Gentra Systems). Extracted DNA was quantified using Quant-iT^TM^ PicoGreen dsDNA reagent (Invitrogen) and stored at -20°C. One microgram of DNA was bisulfite converted using the EZ-DNA Methylation kit (Zymo Research) following the manufacturer’s protocol.  The Illumina HumanMethylation450 BeadChip was used to profile methylation level at 485,577 CpG sites.

Covariates

Maternal smoking, education, and parity information were obtained through questionnaire about 4 months after their delivery. Estimation of six different white blood cell types (CD8+ T and CD4+ T lymphocytes, CD56+ natural killer cells, CD19+ B cells, CD14+ monocytes, and granulocytes) by Houseman method[15] was performed using the default implementation of the *estimateCellCounts* function in the minfi package[16].

**NHBCS**

Design and Study Population:

The New Hampshire Birth Cohort Study (NHBCS) is an ongoing prospective study that began in 2009 and includes over 1500 women receiving prenatal care in New Hampshire, USA, enrolled between approximately 24-28 weeks gestation. Mothers were recruited into the cohort if they were literate in English, between 18–45 years old, and reported using a private, unregulated well as the primary source of home drinking water. Infants included in the cohort were singleton pregnancies. Pre- and post-delivery questionnaires were administered to collect self-reported sociodemographic, lifestyle, and medical history data, and a structured medical records review was employed to collect information from the pregnancy and delivery. Cord blood samples are collected on >80% of eligible deliveries. This study consisted of the first participants born in the study with available cord blood samples for DNA methylation analysis and mothers that were not missing data for self-reported body mass index (BMI) (n=118).

Covariates:

Self-reported pre-pregnancy height and weight were used to calculate maternal BMI (min = 17.81 kg/m^2^, 10^th^ percentile = 19.56 kg/m^2^, 25^th^ percentile = 21.59 kg/m^2^, median = 23.61 kg/m^2^, 75^th^ percentile = 26.61 kg/m^2^, 90^th^ percentile = 29.91 kg/m^2^, max = 39.85 kg/m^2^). There were no BMIs >= +/- 5 SD from the mean. Only 3 participants were classified as underweight; all other BMI sub-categories (normal, overweight, and obese) had at least 5 participants. Maternal socioeconomic status was approximated via highest educational attainment (*High school graduation or less* vs. *At least some post high school education*). Parity was defined as the number of prior birth (*Ever previously birth* vs. *Never previously birth*). Maternal smoking during pregnancy was approximated via self-reported smoking history (*Any smoking during pregnancy* vs. *No smoking during pregnancy*).

Methylation Measurements:

DNA was bisulfite con­verted using the EZ DNA Methylation kit and subsequently subjected to epigenome-wide DNA methyla­tion assessment using the Illumina Infinium HumanMethylation450 BeadChip at the University of Minnesota Genomics Core Facility following standardized protocols. Post-array processing was conducted in the ‘minfi’ package in R. Array control probes were used to assess the quality of our samples and evaluate potential poor bisulfite conversion or color-specific issues for each array. Probes with detection p-values > 0.01 in at least one sample were removed. Data was then normalized using functional normalization (funNorm). Batch effects were removed from the data via ComBat; removal of batch effects was confirmed with principal components analysis. The normalized and batch-corrected beta-values were utilized for the following analyses.

Cell Type Corrections:

Proportions of cell types were estimated from the 450K DNAM via the *estimateCellCounts* function within the ‘minfi’ package in R. Due to the sum of the six estimated cell proportions adding to 1.0 for each participant, only five of the cell-types were added to the models as covariates (granulocytes were excluded).

**PIAMA**

The PIAMA (The prevention and incidence of asthma and mite allergy) study is a birth cohort study of children in the general population, born between 1996-1997. Details of the study design have been published previously[41]. Recruitment took place during the first trimester of pregnancy: 10,232 pregnant women completed a validated screening questionnaire at their prenatal health care clinic (n=52). Mothers reporting a history of asthma, current hay fever or allergy to pets or house dust mite were defined as allergic. All allergic mothers and a large subsample of the non-allergic women (total n=7,862) were invited to participate, of whom 4,146 women agreed and gave informed consent. Questionnaire based follow-up of the children took place at 3 months of age, yearly from 1 to 8 years of age, and at 11, 14, 16, and 17/18 years of age, with clinical investigations in subsamples of the study population at ages 4, 8, 12 and 16 years.

Methylation measurements and preprocessing

In the PIAMA study, peripheral blood samples were collected from all consenting cohort participants, and DNA from peripheral blood was isolated by the laboratories participating in the MEDALL study using different methods. To uniform the concentration and purity the samples underwent a precipitation-based concentration and purification using GlycoBlue (Ambion) if needed. DNA concentration was determined by Nanodrop measurement and picogreen quantification. After normalization of the concentration, the samples were randomized to avoid batch effects. Standard male and female DNA samples were included in this step for control reasons. 500 ng of DNA of each sample was bisulfite-converted using the EZ 96-DNA methylation kit following the manufacturer’s standard protocol. After verification of the bisulfite conversion using Sanger Sequencing, the DNA methylation was measured using the Illumina Infinium HumanMethylation450 beadchip.

Data preprocessing was performed using the Minfi package [18]. We implemented sample filtering to remove bad quality (call rate <99%) and mixed up samples

Moreover, we used 65 SNP probes to check for concordances between paired DNA samples with Nasal brush sample from the same individual and assessed the methylation distribution of X-chromosome to verify gender. Paired samples which show Pearson correlation coefficient <0.9 were regarded as sample mixed ups and were excluded from the study. 644 good quality 16 years PIAMA samples have been used in the analysis. During processing, the probes on sex chromosomes, the probes that mapped on multi-loci, 65 SNPs assays and the probes containing SNPs at the target CpG sites with a MAF>10% were excluded. This led to a total number of 439306 CpG sites. Third, we implemented “DASEN” to perform signal correction and normalization [5].

Covariates

Maternal age is defined as continuous variables. Maternal social class is defined as three categories: highest attained educational level mother 1=primary school, lower vocational or lower secondary education (low) 2=intermediate vocational education or intermediate/higher secondary education (intermediate) 3= higher vocational education and university (high). Parity is defined as older siblings living in the PIAMA home. Pre-pregnancy maternal BMI was calculated using height and weight of the mother before pregnancy, self-reported in the questionnaire when the child was 1 year of age. Maternal smoking was coded as 1= no smoking in pregnancy, 2=smoking but stopped in the first 16 weeks, 3=smoking for longer than 16 weeks.

Models

The final robust linear regression models were adjusted for maternal age, maternal social class, maternal smoking status and batch (2 batches). Cell type correction was applied using the reference-based Houseman method [15] in the minfi package. This method estimates the relative proportions of six white blood cell subtypes (CD4+ T-lymphocytes, CD8+ T-lymphocytes, NK (natural killer) cells, B-lymphocytes, monocytes and granulocytes.

**RAINE**

Design and study population

The Western Australia Pregnancy Cohort (Raine) study (http://www.rainestudy.org.au) is a longitudinal Australian birth cohort that has serially assessed the offspring of 2900 pregnant women from 18 weeks gestation in utero. Follow-up of the offspring has been undertaken at 1, 2, 3, 5, 8, 10, 14, 17 and 24 years [42,43].

Maternal and paternal BMI

Maternal pre-pregnancy weight and height were self-reported.

DNA methylation

DNA was extracted from whole blood samples obtained at 17 year old follow up. Bisulphite conversion was prepared from whole blood cells by standard phenol:chloroform extraction and ethanol precipitation. Processing of the Illumina Infinium HumanMethylation450 BeadChips was carried out by the Centre for Molecular Medicine and Therapeutics (CMMT) <http://www.cmmt.ubc.ca>. The raw IDAT files were imported into R using the rnb.run.import() function available in the *RnBeads* package[44]. Two packages were used to perform quality control checks of the samples; *shinyMethyl* [45] and *MethylAid* [46]. And three samples were evident as outliers and removed. We removed intentional SNP probes (n=65), sex chromosome probes (n=11,648), probes with a detection *p*-value greater than 0.05 in any sample (n=10,777). A further 160 probes with low bead counts (bead counts less than 3 in more than 5% of samples) were removed. Probes were normalized using BMIQ[14] and an additional 94 probes were removed resulting in 462,833 probes for analysis.

Covariates

Data on maternal age, educational level and parity and maternal smoking during pregnancy were assessed by questionnaires at 18 and 34 weeks pregnancy. Maternal age at delivery was derived from the mother’s report of her own and her baby’s dates of birth. We used plate for technical batch variable.

Cell type correction

Cell type correction was determined using the reference-based Houseman method in the minfi package in R. This method estimates the relative proportions of six white blood cell subtypes (CD4+ T-lymphocytes, CD8+ T-lymphocytes, NK (natural killer) cells, B-lymphocytes, monocytes and granulocytes), based on a standard reference population.

**RICHS**

Design and Study Population:

Study participants are part of the Rhode Island Child Health Study (RICHS), which enrolled mother-infant pairs following delivery at Women and Infants Hospital (Providence, RI, USA) from 2009-2014. A total of 840 mother-infant pairs were enrolled. All subjects provided written informed consent approved by the Institutional Review Boards at Women and Infants Hospital and Dartmouth College. Mothers were between the ages of 18 and 40, free of life threatening conditions, and there were no congenital or chromosomal abnormalities. Infants were singleton births, with gestation to term (≥ 37 weeks). Term infants born small for gestational age (SGA, <10^th^ percentile), or large for gestational age (LGA, >90^th^ percentile), based on birth weight and gestational age calculated from the Fenton growth chart, were selected; infants appropriate for gestational age (AGA, ≥10^th^ percentile and ≤90^th^ percentile) matched on gender, gestational age (±3 days), and maternal age (±2 years) were also enrolled. This design led to an overrepresentation of SGA and LGA infants within the cohort. A structured chart review was conducted to collect maternal inpatient information from the delivery. Anthropometric and clinical data was collected from the inpatient medical record from delivery. After delivery but prior to discharge, mothers participated in an interviewer-administered structured questionnaire to obtain information on exposures, demographics, and lifestyle factors. Cord blood samples were obtained as residual tissues from samples collected at delivery and maintained in the Department of Pathology for potential clinical use. Once the infant was discharged, remaining cord blood samples were collected for the study. A total of 450 newborn cord blood samples were collected, and the major demographic features of the infants with cord blood samples did not differ from those of the whole cohort. This study included a random subset of infants for which cord blood samples were obtained for DNA methylation analyses and whose mothers were not missing for self-reported body mass index (BMI) (N=96).

Covariates:

Self-reported pre-pregnancy height and weight were used to calculate maternal BMI (min = 15.93 kg/m^2^, 10^th^ percentile = 18.76 kg/m^2^, 25^th^ percentile = 20.85 kg/m^2^, median = 23.81 kg/m^2^, 75^th^ percentile = 29.60 kg/m^2^, 90^th^ percentile =36.86 kg/m^2^, max = 45.93 kg/m^2^). There were no BMIs >= +/- 5 SD from the mean and all sub-categories of BMI (underweight, normal, overweight, and obese) had at least 5 participants. Maternal socioeconomic status was approximated via highest educational attainment (*High school graduation or less* vs. *At least some post high school education*). Parity was approximated via previous pregnancy history (*Ever previously pregnant* vs. *Never previously pregnant*). Maternal smoking during pregnancy was measured via self-reported smoking history (*Any smoking during pregnancy* vs. *No smoking during pregnancy*).

Methylation Measurements:

DNA was bisulfite con­verted using the EZ DNA Methylation kit and subsequently subjected to epigenome-wide DNA methyla­tion assessment using the Illumina Infinium HumanMethylation450 BeadChip at the University of Minnesota Genomics Core Facility following standardized protocols. Post-array processing was conducted in the ‘minfi’ package in R. Array control probes were used to assess the quality of our samples and evaluate potential poor bisulfite conversion or color-specific issues for each array. Probes with detection p-values > 0.01 in at least one sample were removed. Data was then normalized using functional normalization (funNorm). Batch effects were removed from the data via ComBat; removal of batch effects was confirmed with principal components analysis. The normalized and batch-corrected beta-values were utilized for the following analyses.

Cell Type Corrections:

Proportions of cell types were estimated from the 450K DNAM via the *estimateCellCounts* function within the ‘minfi’ package in R. Due to the sum of the six estimated cell proportions adding to 1.0 for each participant, only five of the cell-types were added to the models as covariates (granulocytes were excluded).

**Project Viva**

Design and study population

Project Viva is a prospective pre-birth cohort of mothers and their children recruited from a multispecialty group practice in Eastern Massachusetts, USA, which has been described in detail elsewhere[47]. The Institutional Review Board of Harvard Pilgrim Health Care approved the study and participating women provided written informed consent. Eligibility requirements were: ability to answer questions in English, at <22 weeks of gestation at study entry, and a singleton pregnancy. Women were enrolled from 1999 to 2002 and enrollment included a total of 2128 live births. Follow up of the children through adolescence is ongoing.

Cord blood DNA methylation assays were completed in 2014 for 507 Viva infants with genetic consent. The current maternal pre-pregnancy BMI analyses were restricted to 343 mothers who reported as non-Hispanic White.

Maternal and paternal BMI

Data on maternal pre-pregnancy BMI is based on self-report at enrollment of height and pre-pregnancy weight. Of the 343 mothers included, 41 were categorized as obese, 77 as overweight, 215 as normal weight, and 10 as underweight.

Methylation measurements

Trained medical personnel obtained venous umbilical cord blood samples immediately after delivery, which they promptly stored in a dedicated refrigerator (4ºC) and transported for processing within 24 hours. Trained laboratory staff processed the samples on the same day, and extracted DNA by using the Qiagen Puregene Kit (Valencia, CA). Aliquots were then stored at -80ºC until analysis.

DNA samples were arranged using a stratified randomization to ensure balance of cohort characteristics across sample plates/batches. Samples were bisulfite converted using the EZ-96 DNA Methylation kit (Zymo Research Corporation, Irvine, USA). Illumina FastTrack Microarray Services (San Diego, CA) performed the analyses using the Illumina Infinium HumanMethylation450 BeadChip (Illumina Inc., San Diego, USA). Failing samples were rerun and passing arrays were defined as having >99% of probes with a detection p value <0.05. Samples with identity concerns (inconsistent genotyping and/or inferred sex) were excluded. Standard sample preprocessing included the exclusion of allosomal probes, non-CpG probes, and failing probes (<99% of samples with detection p values <0.05). Further pre-processing and normalization steps included background adjustment via the normal-exponential out-of-band (“noob”) background correction method with dye-bias equalization[48], and further within-array type II probe adjustment using the Beta-Mixture Quantile Dilation (BMIQ) approach[14].

Covariates

The ComBat method was used to adjust the methylation data for sample plate, to reduce potential for bias due to batch effects. Robust regression analyses were then run, adjusting for continuous maternal age (reported at enrolment), smoking status (categorized as never, former, smoked any time during pregnancy), educational status (college graduate vs. not a college graduate), parity (categorized as 0 [nulliparous] vs. 1 or more [multiparous]), and estimated cell type proportions obtained via the statistical deconvolution method of Houseman *et al*. Estimation of six different white blood cell types (CD8+ T and CD4+ T lymphocytes, CD56+ natural killer cells, CD19+ B cells, CD14+ monocytes, and granulocytes) by Houseman method[15] was performed using the default implementation of the *estimateCellCounts* function in the minfi package[16] (granulocytes were excluded).

References

1. Sharp GC, Lawlor DA, Richmond RC, Fraser A, Simpkin A, Suderman M, et al. Maternal pre-pregnancy BMI and gestational weight gain, offspring DNA methylation and later offspring adiposity: findings from the Avon Longitudinal Study of Parents and Children. Int. J. Epidemiol. 2015;

2. Wickman M, Kull I, Pershagen G, Nordvall SL. The BAMSE project: presentation of a prospective longitudinal birth cohort study. Pediatr. Allergy Immunol. 2002;11–3.

3. Ekström S, Magnusson J, Kull I, Lind T, Almqvist C, Melén E, et al. Maternal body mass index in early pregnancy and offspring asthma, rhinitis and eczema up to 16 years of age. Clin. Exp. Allergy. 2015;45:283–91.

4. Chen Y, Lemire M, Choufani S, Butcher DT, Grafodatskaya D, Zanke BW, et al. Discovery of cross-reactive probes and polymorphic CpGs in the Illumina Infinium HumanMethylation450 microarray. Epigenetics. 2013;8:203–9.

5. Pidsley R, Y Wong CC, Volta M, Lunnon K, Mill J, Schalkwyk LC. A data-driven approach to preprocessing Illumina 450K methylation array data. BMC Genomics. 2013;14:293.

6. Johnson WE, Li C, Rabinovic A. Adjusting batch effects in microarray expression data using empirical Bayes methods. Biostatistics. 2007;8:118–27.

7. Ma X, Buffler PA, Wiemels JL, Selvin S, Metayer C, Loh M, et al. Ethnic difference in daycare attendance, early infections, and risk of childhood acute lymphoblastic leukemia. Cancer Epidemiol. Biomarkers Prev. 2005;14:1928–34.

8. Fortin J-P, Labbe A, Lemire M, Zanke BW, Hudson TJ, Fertig EJ, et al. Functional normalization of 450k methylation array data improves replication in large cancer studies. Genome Biol. 2014;15:503.

9. Rahmani E, Zaitlen N, Baran Y, Eng C, Hu D, Galanter J, et al. Sparse PCA corrects for cell type heterogeneity in epigenome-wide association studies. Nat. Methods. 2016;13:443–5.

10. Joo JE, Wong EM, Baglietto L, Jung C-H, Tsimiklis H, Park DJ, et al. The use of DNA from archival dried blood spots with the Infinium HumanMethylation450 array. BMC Biotechnol. 2013;13:23.

11. Eskenazi B, Bradman A, Gladstone E a., Jaramillo S, Birch K, Holland N. CHAMACOS, A Longitudinal Birth Cohort Study: Lessons from the Fields. J. Child. Heal. 2003;1:3–27.

12. Eskenazi B, Harley K, Bradman A, Weltzien E, Jewell NP, Barr DB, et al. Association of in utero organophosphate pesticide exposure and fetal growth and length of gestation in an agricultural population. Environ. Health Perspect. 2004;112:1116–24.

13. Yousefi P, Huen K, Schall RA, Decker A, Elboudwarej E, Quach H, et al. Considerations for normalization of DNA methylation data by Illumina 450K BeadChip assay in population studies. Epigenetics. Landes Bioscience; 2013;8:11.

14. Teschendorff AE, Marabita F, Lechner M, Bartlett T, Tegner J, Gomez-Cabrero D, et al. A beta-mixture quantile normalization method for correcting probe design bias in Illumina Infinium 450 k DNA methylation data. Bioinformatics. 2013;29:189–96.

15. Houseman EA, Accomando WP, Koestler DC, Christensen BC, Marsit CJ, Nelson HH, et al. DNA methylation arrays as surrogate measures of cell mixture distribution. BMC Bioinformatics. 2012;13:86.

16. Jaffe AE, Irizarry RA. Accounting for cellular heterogeneity is critical in epigenome-wide association studies. Genome Biol. 2014;15:R31.

17. Newschaffer CJ, Croen LA, Fallin MD, Hertz-Picciotto I, Nguyen D V, Lee NL, et al. Infant siblings and the investigation of autism risk factors. J. Neurodev. Disord. 2012;4:7.

18. Aryee MJ, Jaffe AE, Corrada-Bravo H, Ladd-Acosta C, Feinberg AP, Hansen KD, et al. Minfi: a flexible and comprehensive Bioconductor package for the analysis of Infinium DNA methylation microarrays. Bioinformatics. 2014;30:1363–9.

19. Triche T. IlluminaHumanMethylation450k.db: Illumina Human Methylation 450k annotation data. 2014.

20. Leek JT, Storey JD. Capturing heterogeneity in gene expression studies by surrogate variable analysis. PLoS Genet. 2007;3:1724–35.

21. Reinius LE, Acevedo N, Joerink M, Pershagen G, Dahlén SE, Greco D, et al. Differential DNA methylation in purified human blood cells: Implications for cell lineage and studies on disease susceptibility. PLoS One. 2012;7.

22. R Core Team, R Development Core Team. R: A language and environment for statistical computing. Vienna, Austria: R Foundation for Statistical Computing; 2012.

23. Guillemette L, Allard C, Lacroix M, Patenaude J, Battista M-C, Doyon M, et al. Genetics of Glucose regulation in Gestation and Growth (Gen3G): a prospective prebirth cohort of mother-child pairs in Sherbrooke, Canada. BMJ Open. 2016;6:e010031.

24. Jaddoe VW V, van Duijn CM, Franco OH, van der Heijden AJ, van Ijzendoorn MH, de Jongste JC, et al. The Generation R Study: design and cohort update 2012. Eur. J. Epidemiol. 2012;27:739–56.

25. Genome of the Netherlands Consortium. Whole-genome sequence variation, population structure and demographic history of the Dutch population. Nat. Genet. 2014;46:818–25.

26. Bonder MJ, Kasela S, Kals M, Tamm R, Lokk K, Barragan I, et al. Genetic and epigenetic regulation of gene expression in fetal and adult human livers. BMC Genomics. 2014;15:860.

27. Touleimat N, Tost J. Complete pipeline for Infinium(®) Human Methylation 450K BeadChip data processing using subset quantile normalization for accurate DNA methylation estimation. Epigenomics. Future Medicine Ltd London, UK; 2012;4:325–41.

28. Paternoster L, Evans DM, Nohr EA, Holst C, Gaborieau V, Brennan P, et al. Genome-wide population-based association study of extremely overweight young adults--the GOYA study. PLoS One. Public Library of Science; 2011;6:e24303.

29. Lehne B, Drong AW, Loh M, Zhang W, Scott WR, Tan S-T, et al. A coherent approach for analysis of the Illumina HumanMethylation450 BeadChip improves data quality and performance in epigenome-wide association studies. Genome Biol. 2015;16:37.

30. Guxens M, Ballester F, Espada M, Fernández MF, Grimalt JO, Ibarluzea J, et al. Cohort Profile: the INMA--INfancia y Medio Ambiente--(Environment and Childhood) Project. Int. J. Epidemiol. 2012;41:930–40.

31. Casas M, Chatzi L, Carsin A-E, Amiano P, Guxens M, Kogevinas M, et al. Maternal pre-pregnancy overweight and obesity, and child neuropsychological development: two Southern European birth cohort studies. Int. J. Epidemiol. 2013;42:506–17.

32. Heude B, Forhan A, Slama RR, Douhaud L, Bedel S, Saurel-Cubizolles M-JJ, et al. Cohort Profile: The EDEN mother-child cohort on the prenatal and early postnatal determinants of child health and development. Int. J. Epidemiol. 2016;45:353–63.

33. Magnus P, Birke C, Vejrup K, Haugan A, Alsaker E, Daltveit AK, et al. Cohort Profile Update: The Norwegian Mother and Child Cohort Study (MoBa). Int. J. Epidemiol. 2016;45:382–8.

34. Rønningen KS, Paltiel L, Meltzer HM, Nordhagen R, Lie KK, Hovengen R, et al. The biobank of the Norwegian Mother and Child Cohort Study: a resource for the next 100 years. Eur. J. Epidemiol. 2006;21:619–25.

35. Magnus P, Irgens LM, Haug K, Nystad W, Skjaerven R, Stoltenberg C, et al. Cohort profile: the Norwegian Mother and Child Cohort Study (MoBa). Int. J. Epidemiol. 2006;35:1146–50.

36. Håberg SE, London SJ, Nafstad P, Nilsen RM, Ueland PM, Vollset SE, et al. Maternal folate levels in pregnancy and asthma in children at age 3 years. J. Allergy Clin. Immunol. 2011;127:262–4, 264.e1.

37. Joubert BR, Håberg SE, Nilsen RM, Wang X, Vollset SE, Murphy SK, et al. 450K epigenome-wide scan identifies differential DNA methylation in newborns related to maternal smoking during pregnancy. Environ. Health Perspect. 2012;120:1425–31.

38. Joubert BR, Felix JF, Yousefi P, Bakulski KM, Just AC, Breton C, et al. DNA Methylation in Newborns and Maternal Smoking in Pregnancy: Genome-wide Consortium Meta-analysis. Am. J. Hum. Genet. 2016;

39. Bibikova M, Barnes B, Tsan C, Ho V, Klotzle B, Le JM, et al. High density DNA methylation array with single CpG site resolution. Genomics. 2011;98:288–95.

40. Leek JT, Johnson WE, Parker HS, Jaffe AE, Storey JD. The sva package for removing batch effects and other unwanted variation in high-throughput experiments. Bioinformatics. 2012;28:882–3.

41. Wijga AH, Kerkhof M, Gehring U, de Jongste JC, Postma DS, Aalberse RC, et al. Cohort profile: The Prevention and Incidence of Asthma and Mite Allergy (PIAMA) birth cohort. Int. J. Epidemiol. 2014;43:527–35.

42. Newnham JP, Evans SF, Michael CA, Stanley FJ, Landau LI. Effects of frequent ultrasound during pregnancy: a randomised controlled trial. Lancet (London, England). 1993;342:887–91.

43. Huang R-C, Mori TA, Beilin LJ. Early life programming of cardiometabolic disease in the Western Australian pregnancy cohort (Raine) study. Clin. Exp. Pharmacol. Physiol. 2012;39:973–8.

44. Assenov Y, Müller F, Lutsik P, Walter J, Lengauer T, Bock C. Comprehensive analysis of DNA methylation data with RnBeads. Nat. Methods. 2014;11:1138–40.

45. Fortin J-P, Fertig E, Hansen K. shinyMethyl: interactive quality control of Illumina 450k DNA methylation arrays in R. F1000Research. 2014;3:175.

46. van Iterson M, Tobi EW, Slieker RC, den Hollander W, Luijk R, Slagboom PE, et al. MethylAid: visual and interactive quality control of large Illumina 450k datasets. Bioinformatics. 2014;30:3435–7.

47. Oken E, Baccarelli AA, Gold DR, Kleinman KP, Litonjua AA, De Meo D, et al. Cohort profile: project viva. Int. J. Epidemiol. 2015;44:37–48.

48. Triche TJ, Weisenberger DJ, Van Den Berg D, Laird PW, Siegmund KD. Low-level processing of Illumina Infinium DNA Methylation BeadArrays. Nucleic Acids Res. 2013;41:e90.

## Supplemental Acknowledgements

**ALSPAC:** We are extremely grateful to all the families who took part in this study, the midwives for their help in recruiting them, and the whole ALSPAC team, which includes interviewers, computer and laboratory technicians, clerical workers, research scientists, volunteers, managers, receptionists, and nurses. We would like to acknowledge Tom Gaunt, Oliver Lyttleton, Sue Ring, Nabila Kazmi, and Geoff Woodward for their earlier contribution to the generation of ARIES data (ALSPAC methylation data).

**BAMSE:** We would like thank all the families for their participation in the BAMSE study. In addition, we would like to thank Eva Hallner, Sara Nilsson and André Lauber at the BAMSE secretary for invaluable support, as well as the Genome Analysis Facility at the Dept. of Genetics, UMCG, Groningen, the Netherlands for genome-wide methylation analysis. The computations were performed on resources provided by SNIC through Uppsala Multidisciplinary Center for Advanced Computational Science (UPPMAX) under Project b2014110.

**CBC (Hispanic and white):** We acknowledge Prof. Catherine Metayer for her support advice regarding resampling methods. The CCLS is supported by our clinical collaborators and participating hospitals, which includes: University of California Davis Medical Center (Dr. Jonathan Ducore), University of California San Francisco (Dr. Mignon Loh and Dr. Katherine Matthay), Children’s Hospital of Central California (Dr. Vonda Crouse), Lucile Packard Children’s Hospital (Dr. Gary Dahl), Children’s Hospital Oakland (Dr. James Feusner), Kaiser Permanente Sacramento (Dr. Vincent Kiley), Kaiser Permanente Santa Clara (Dr. Carolyn Russo and Dr. Alan Wong), Kaiser Permanente San Francisco (Dr. Kenneth Leung), and Kaiser Permanente Oakland (Dr. Stacy Month), and the families of the study participants.

**CHAMACOS:** We are grateful to the CHAMACOS staff, students, community partners, participants and their families. We would also like to thank Dr. Kim Harley and Ms. Katherine Kogut for their contributions to this study.

**EARLI:** We thank the families, clinicians, and study staff who participated in EARLI. We thank JHBR for sample processing and the JHU SNP Center for performing the methylation assays.

**GECKO:** We are grateful to the families who took part in the GECKO Drenthe study, the midwives, gyneacologists, nurses and GPs for their help for recruitment and measurement of participants, and the whole team from the GECKO Drenthe study.

**GEN3G:** Gen3G investigators acknowledge the Blood sampling in pregnancy clinic at the Centre Hospitalier de l'Universite de Sherbrooke (CHUS), and the assistance of clinical research nurses for recruiting women and obtaining consent for the study at the Research Center of CHUS. They also thank the CHUS Research in obstetrics services (collaborator JC Pasquier) for organization of biosamples collection at delivery.

**GENR:** The Generation R Study is conducted by the Erasmus Medical Center in close collaboration with the School of Law and Faculty of Social Sciences of the Erasmus University Rotterdam, the Municipal Health Service Rotterdam area, Rotterdam, the Rotterdam Homecare Foundation, Rotterdam and the Stichting Trombosedienst & Artsenlaboratorium Rijnmond (STAR-MDC), Rotterdam. We gratefully acknowledge the contribution of children and parents, general practitioners, hospitals, midwives and pharmacies in Rotterdam. The study protocol was approved by the Medical Ethical Committee of the Erasmus Medical Centre, Rotterdam. Written informed consent was obtained for all participants. The generation and management of the Illumina 450K methylation array data (EWAS data) for the Generation R Study was executed by the Human Genotyping Facility of the Genetic Laboratory of the Department of Internal Medicine, Erasmus MC, the Netherlands. We thank Ms. Sarah Higgins, Ms. Mila Jhamai, Dr. Marjolein Peters, Dr. Lisette Stolk, Mr. Michael Verbiest, and Mr. Marijn Verkerk for their help in creating the EWAS database and the analysis pipeline.

**GOYA:** GOYA (Genomics of Obesity in Young Adults) was sampled as a case-cohort study within the Danish National Birth Cohort. The Danish National Birth Cohort was established with a significant grant from the Danish National Research Foundation. Additional support was obtained from the Danish Regional Committees, the Pharmacy Foundation, the Egmont Foundation, the March of Dimes Birth Defects Foundation, the Health Foundation and other minor grants. The DNBC Biobank has been supported by the Novo Nordisk Foundation and the Lundbeck Foundation.

**IOW Birth Cohort (IOW F1):** We would like to thank all the participants of the Isle of Wight birth cohort, the research team at David Hide Asthma & Allergy Research Centre (Isle of Wight) for collecting the data, Nikki Graham for technical support and other members of the IoW research group for valuable discussion. DNA methylation data was generated by the Oxford Genomics Centre at the Wellcome Trust Centre for Human Genetics.

**IOW 3^rd^ generation cohort (IOW F2):** We are sincerely thankful to all the families who took part in this study, the nurses for their help in recruiting them, and the whole IOW team. In particular, we would like to thank Stephen Porter, Sharon Matthews, Frances Mitchell.

**MeDALL INMA:** INMA researchers would like to thank all the participants for their generous collaboration. INMA researchers are grateful to Silvia Fochs, Nuria Pey, and Muriel Ferrer for their assistance in contacting the families and administering the questionnaires. The study was approved by the Ethical Committee of the Municipal Institute of Medical Investigation and by the Ethical Committee of the hospitals involved in the study. The pregnant women received information of the study both written and orally. Their informed consent of the participants was asked in each of the visits. A full roster of the INMA Project Investigators can be found at <http://www.proyectoinma.org/presentacion-inma/listado-investigadores/en_listado-investigadores.html>.

**MeDALL EDEN:** The analysis for EDEN is the result of a Collaboration INSERM et CEA-IG- CNG Epigenetique. On behalf of the EDEN Mother-Child Cohort Study Group, we thank the study participants and staff for their participation in this cohort.

**MOBA (1,2,3):** We are grateful to all the participating families in Norway who take part in this on-going cohort study.

**NEST:** We thank the parents and other caregivers of the Newborn Epigenetics Study. We also thank the field and laboratory staff for their effort.

**NFCS:** We would like to thank all individuals for participating in the Norway Facial Clefts Study.  This research was supported by the Intramural Research Program of the NIH, National Institute of Environmental Health Sciences.

**NHBCS:** We would like to thank all the families that participated in NHBCS, as well as the staff involved in recruitment, field work and data collection.

**PIAMA:** The authors thank all the children and their parents for their cooperation. The authors also thank all the field workers and laboratory personnel involved for their efforts, Marieke Oldenwening for logistic coordination and Marjan Tewis and Ada Vos for data management.

**RAINE:** The authors are grateful to the Raine Study participants and their families, and the Raine Study management team for cohort co-ordination and data collection.

**RICHS:** We would like to thank all the families that participated in the RICHS study, as well as the clinical and research staff, particularly Joyce Lee (Department of Pediatrics, Women’s and Infants Hospital), involved in recruitment and data collection.

**Project Viva:** We are indebted to the Project Viva mothers, children and families

## Funding Support

**ALSPAC:** The UK Medical Research Council and the Wellcome Trust (Grant ref: 102215/2/13/2) and the University of Bristol provide core support for ALSPAC. The Accessible Resource for Integrated Epigenomics Studies (ARIES) which generated large scale methylation data was funded by the UK Biotechnology and Biological Sciences Research Council (BB/I025751/1 and BB/I025263/1). Additional epigenetic profiling on the ALSPAC cohort was supported by the UK Medical Research Council Integrative Epidemiology Unit and the University of Bristol (MC_UU_12013_1, MC_UU_12013_2, MC_UU_12013_5 and MC_UU_12013_8), the Wellcome Trust (WT088806) and the United States National Institute of Diabetes and Digestive and Kidney Diseases (R01 DK10324).The funders had no role in study design, data collection and analysis, decision to publish, or preparation of the manuscript.

**BAMSE:** BAMSE was supported by The Swedish Research Council, The Swedish Heart-Lung Foundation, MeDALL (Mechanisms of the Development of ALLergy) a collaborative project conducted within the European Union (grant agreement No. 261357), Stockholm County Council (ALF), the Strategic Research Programme (SFO) in Epidemiology at Karolinska Institutet, The Swedish Research Council Formas and the Swedish Environment Protection Agency.

**CBC (Hispanic and white):** This work was supported by the National Institute of Environmental Health Sciences and the Environmental Protection Agency [grant number: P01ES018172 to C.M., R.R. and J.L.W.]; The National Institute of Environmental Health Sciences [grant number: R01ES09137 to C.M., R.R. and J.L.W.]; The National Cancer Institute [grant number: R01CA155461 to R.R. and J.L.W.]; The Swiss Cancer League [grant number: BIL KLS 3124–02–2013 to S. G.]; The Swiss Science National Foundation [grants numbers: P2LAP3_158674 to S.G., P2LAP3_148434 to S.N.]; The SICPA Foundation to S.G.; the Sutter-Stottner Foundation to S.G.; a Cancer Center Support Grant (P30CA82103 to R.R. The content is solely the responsibility of the authors and does not necessarily represent the official views of the National Institutes of Health or the Environmental Protection Agency.

**CHAMACOS:** The CHAMACOS study was supported by the NIH grants P01 ES009605 and R01 ES021369 and EPA grants RD 82670901 and RD 83451301.

**EARLI:** Funding for this work was provided by the National Institutes of Health (R01ES017646, R01ES16443) and Autism Speaks (grant #260377).

**GECKO:** The GECKO Drenthe birth cohort was funded by an unrestricted grant of Hutchison Whampoa Ld, Hong Kong and supported by the University of Groningen, Well Baby Clinic Foundation Icare, Noordlease and Youth Health Care Drenthe. This methylation project in the GECKO Drenthe cohort was supported by the Biobanking and Biomolecular Research Infrastructure Netherlands (CP2011-19).

**GEN3G:** Gen3G was supported by a Fonds de recherche du Québec en santé (FRQ-S) operating grant (grant #20697); a Canadian Institute of Health Reseach (CIHR) Operating grant (grant #MOP 115071); a Diabète Québec grant. M.F.H. is supported by an American Diabetes Association (ADA) Accelerator Award (#1-15-ACE-26).

**GENR:** The Generation R Study is made possible by financial support from the Erasmus Medical Center, Rotterdam, the Erasmus University Rotterdam and the Netherlands Organization for Health Research and Development. The EWAS data was funded by a grant to VWJ from the Netherlands Genomics Initiative (NGI)/Netherlands Organisation for Scientific Research (NWO) Netherlands Consortium for Healthy Aging (NCHA; project nr. 050-060-810) and by funds from the Genetic Laboratory of the Department of Internal Medicine, Erasmus MC. V.W.J. received a grant from the Netherlands Organization for Health Research and Development (VIDI 016.136.361) and a Consolidator Grant from the European Research Council (ERC-2014-CoG-64916). J.F.F. has received funding from the European Union’s Horizon 2020 research and innovation programme under grant agreement No 633595 (DynaHEALTH). The Generation R Study received funding from the European Union’s Horizon 2020 research and innovation programme (733206, LIFECYCLE).

**GOYA:** Genotyping for the GOYA Study was funded by the Wellcome Trust (Grant ref: 084762MA). Generation of DNA methylation data was funded by the MRC Integrative Epidemiology Unit which is supported by the Medical Research Council (MC_UU_12013/1-9) and the University of Bristol.

**IOW Birth Cohort (IOW F1):** The IoW 1989 (IOW F2) cohort was supported by the National Institute of Allergy and Infectious Diseases under award number R01 AI091905 (PI: Wilfried Karmaus) and R01 AI061471 (PI: Susan Ewart). The 18-year follow-up by a grant from the National Heart and Blood Institute (R01 HL082925, PI: S. Hasan Arshad).

**IOW 3^rd^ generation cohort (IOW F2):** The third generation study was funded by the National Institute of Allergy and Infectious Diseases (NIAID) at the National Institute of Health, R01 AI091905 (PI: Wilfried Karmaus). The work of Hongmei Zhang and John Holloway is also supported by the fund from NIAID/NIH (R01AI121226, MPI: Hongmei Zhang and John Holloway). Mr. Quraishi’s work was also partially funded by Hongmei Zhang’s start up fund from the School of Public Health, University of Memphis.

**MeDALL:** The MeDALL (Mechanisms of the Development of ALLergy) study was supported by the European Union under the Health Cooperation Work Programme of the 7th Framework programme (grant agreement number 261357).

**MeDALL INMA**: INMA was funded by grants from Instituto de Salud Carlos III (Red INMA G03/176, CB06/02/0041), Spanish Ministry of Health (FIS-PI041436, FIS-PI081151), Generalitat de Catalunya-CIRIT 1999SGR 00241, Fundació La marató de TV3 (090430).LAS was supported through a Colciencias PhD Scholarship, Colombia (Grant: 529/2011). CR-A was supported by a FI fellowship from Catalan Government (#016FI_B 00272).

**MeDALL EDEN:** Fonds de Dotation "Recherche en Santé Respiratoire". French Ministry of Research: IFR program, INSERM Nutrition Research Program, French Ministry of Health: Perinatality Program, French National Institute for Population Health Surveillance (INVS), Paris–Sud University, French National Institute for Health Education (INPES), Nestlé, Mutuelle Générale de l’Education Nationale (MGEN), French speaking association for the study of diabetes and metabolism (Alfediam), grant # 2012/51290-6 Sao Paulo Research Foundation (FAPESP), EU funded MedAll project.

**MOBA (1,2,3):** The Norwegian Mother and Child Cohort Study are supported by the Norwegian Ministry of Health and Care Services and the Ministry of Education and Research, NIH/NIEHS (contract no N01-ES-75558), NIH/NINDS (grant no.1 UO1 NS 047537-01 and grant no.2 UO1 NS 047537-06A1). MoBa 1 and 2 were supported by the Intramural Research Program of the NIH, National Institute of Environmental Health Sciences (Z01-ES-49019) and the Norwegian Research Council/BIOBANK (grant no 221097). The work in MoBa3 was supported in part by a Postdoctoral Fellowship grant from the Ullevål Hospitals Research Council (now under Oslo University Hospital) and travel grants from the Unger -Vetlesens foundation and the Norwegian American Womens Club, all to MCMK. MoBa3 data was funded by INCA/INSERM-Plan Cancer, France, and the International Childhood Cancer Cohort Consortium (I4C). The work performed by the Epigenetics Group at the International Agency for Research on Cancer (IARC, Lyon, France), ZH and AG were supported by the grant from INCa/INSERM-Plan Cancer (France, 2015) to ZH. AG was also supported by the IARC Postdoctoral Fellowship, partially supported by the EC FP7 Marie Curie Actions-People-Co-funding of regional, national and international programmes (COFUND). SZ, SJL, AG are supported by the Intramural Research Program of the NIH, National Institute of Environmental Health Sciences.

**NEST:** The NEST study was funded by NIEHS grants R21ES014947 and R01ES016772 and NIDDK grant R01DK085173. CH, SKM, and RLM received funding from the National Institute of Environmental Health Sciences under Award Number P01ES022831 and by USEPA grant RD-83543701. The content is solely the responsibility of the authors and does not necessarily represent the official views of the NIH or the USEPA.). CW received funding from the Office of Research on Women’s Health (K12 HD043446).

**NFCS:** This research was supported by the Intramural Research Program of the NIH, National Institute of Environmental Health Sciences.

**NHBCS:** The NHBCS was supported by NIH-NIEHS P01 ES022832, US EPA grant RD83544201, NIH-NIGMS P20GM104416, and NCI R25CA134286.

**PIAMA:** The PIAMA study was supported by The Netherlands Organization for Health Research and Development; The Netherlands Organization for Scientific Research; The Netherlands Asthma Fund; The Netherlands Ministry of Spatial Planning, Housing, and the Environment; and The Netherlands Ministry of Health, Welfare, and Sport. We also thank the financial supports from Dutch Lung Foundation (AF 4.1.14.001).

**RAINE:** The authors acknowledge the contributions to core funding of the Raine Study by the University of Western Australia, the Telethon Kids Institute, the Raine Medical Research Foundation, the Faculty of Medicine, Dentistry and Health Science (UWA), the Women and Infants Research Foundation, Curtin University, and Edith Cowan University. The authors also acknowledge the long-term support of the National Health and Medical Research Council of Australia. The epigenetic data collection is supported by NHMRC grant #1059711. Rae-Chi Huang supported by NHMRC fellowships 1053384.

**RICHS:** RICHS was supported by the National Institutes of Health NIH-NIMH R01MH094609, NIH-NIEHS R01ES022223, NIH-NIEHS R01ES025145.

**Project Viva:** The Project Viva cohort is funded by NIH grants R01 HL111108, R01 NR013945, and R01 HD034568.
