# Supplementary material for: Maternal BMI at the start of pregnancy and offspring epigenome-wide DNA methylation: findings from the pregnancy and childhood epigenetics (PACE) consortium
Source: Hum Mol Genet. 2017 Jul 21;26(20):4067–85. doi: 10.1093/hmg/ddx290 (PMC5656174; doi:10.1093/hmg/ddx290)
Supplement: Supplementary File 3 [file file_s3_ddx290.pdf]

Supplementary Figures

Contents

1) QQ plots for the cell-adjusted models [Pages 2-5]

2) Forest plots for the cell-adjusted continuous BMI model (top 86 sites) [Pages 6-21]

3) Leave-one-out analyses for the cell-adjusted continuous BMI model (top 86 sites) [Pages 22-37]

4) Density plots of the top 86 sites for

a) ALSPAC data [Pages 38-44]

b) GOYA data [Pages 45-51]

QQ-plots for cell-adjusted continuous BMI model

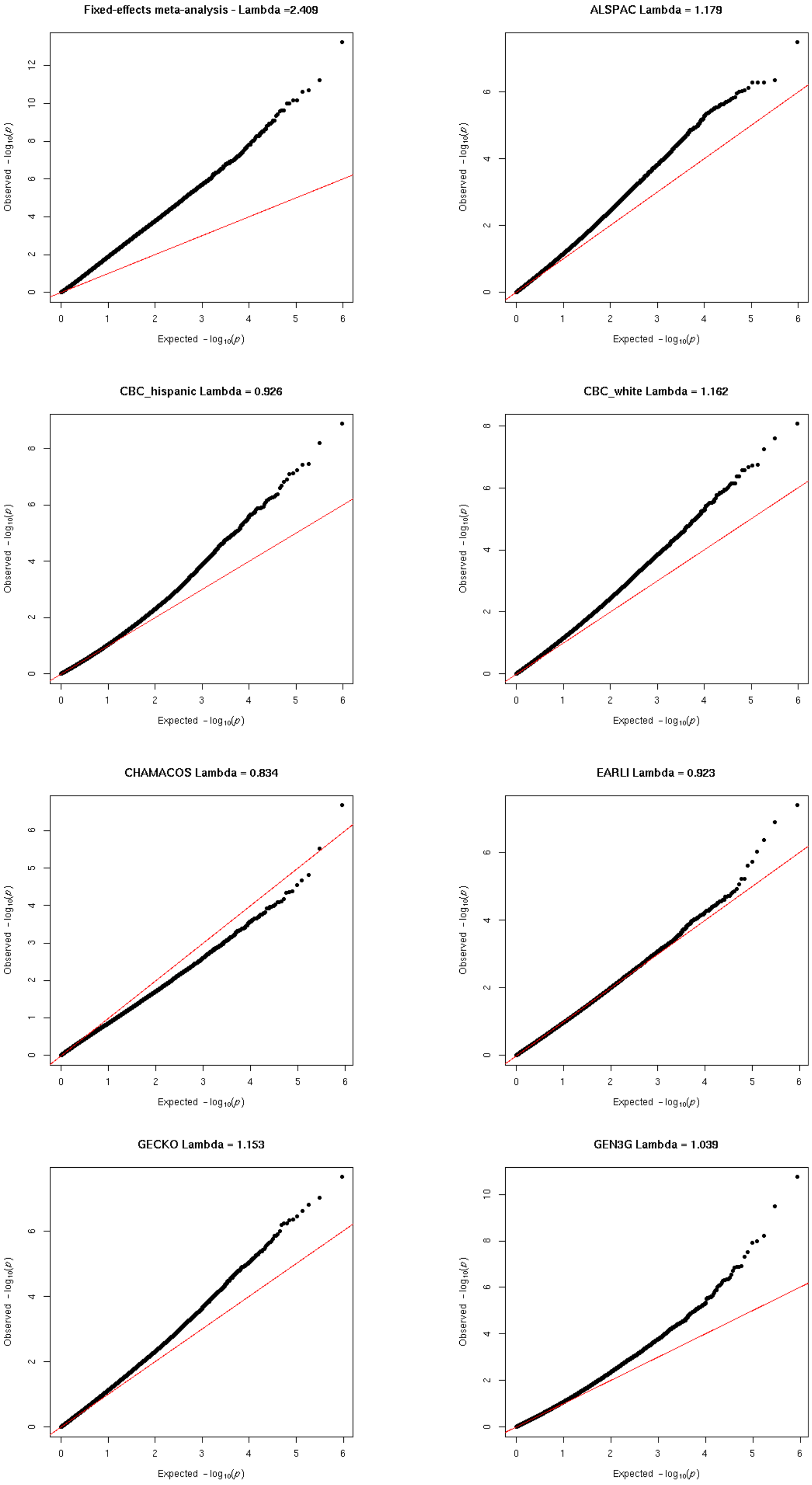

Fixed-effects meta-analysis - Lambda =2.409
ALSPAC Lambda = 1.179
CBC_hispanic Lambda = 0.926
CBC_white Lambda = 1.162
CHAMACOS Lambda = 0.834
EARLI Lambda = 0.923
GECKO Lambda = 1.153
GEN3G Lambda = 1.039
Observed $-\log_{10}(p)$
Expected $-\log_{10}(p)$

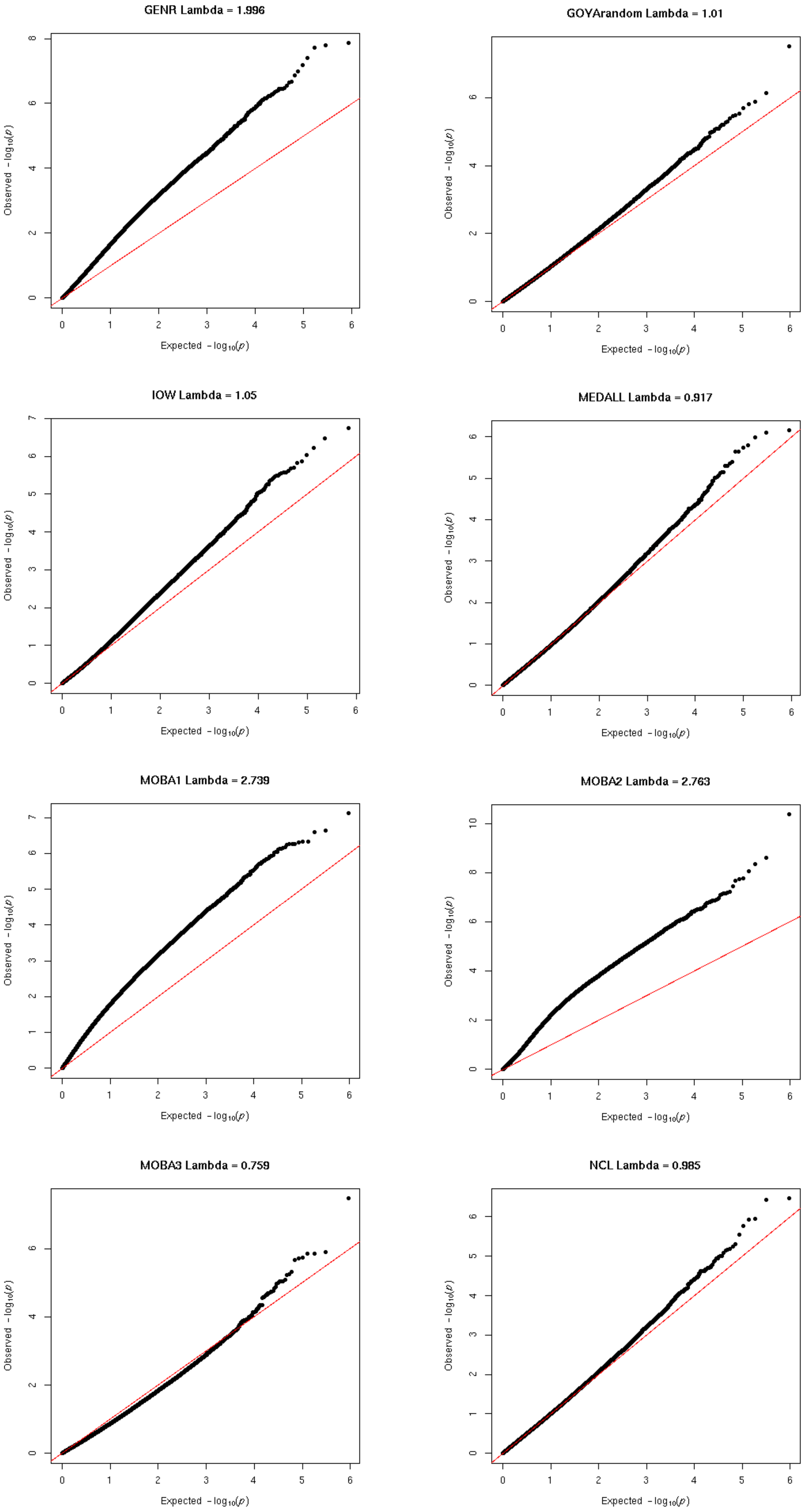
GENR Lambda = 1.996
GOYArandom Lambda = 1.01
IOW Lambda = 1.05
MEDALL Lambda = 0.917
MOBA1 Lambda = 2.739
MOBA2 Lambda = 2.763
MOBA3 Lambda = 0.759
NCL Lambda = 0.985
Observed $-\log_{10}(p)$
Expected $-\log_{10}(p)$

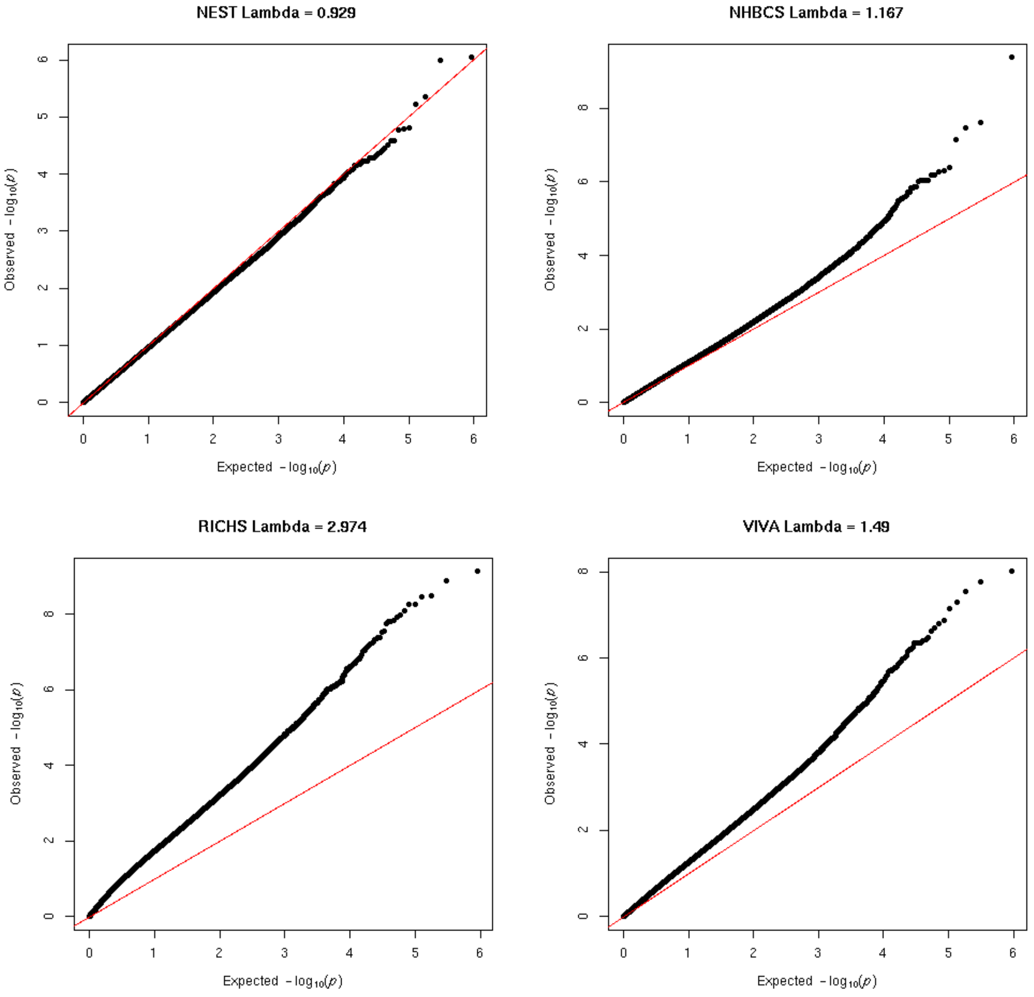
NEST Lambda = 0.929
NHBCS Lambda = 1.167
RICHS Lambda = 2.974
VIVA Lambda = 1.49
Observed −log10(p)
Expected −log10(p)

Forest plots for the cell-adjusted continuous BMI model

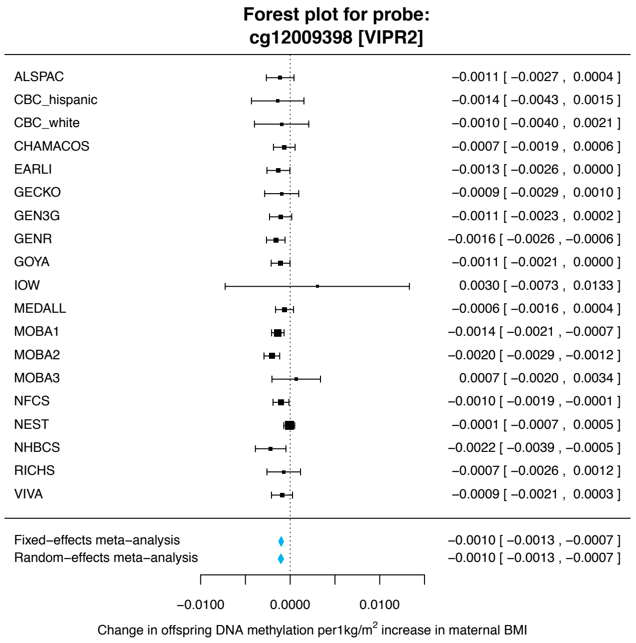
Forest plot for probe:
cg12009398 [VIPR2]
ALSPAC −0.0011 [−0.0027 , 0.0004]
CBC_hispanic −0.0014 [−0.0043 , 0.0015]
CBC_white −0.0010 [−0.0040 , 0.0021]
CHAMACOS −0.0007 [−0.0019 , 0.0006]
EARLI −0.0013 [−0.0026 , 0.0000]
GECKO −0.0009 [−0.0029 , 0.0010]
GEN3G −0.0011 [−0.0023 , 0.0002]
GENR −0.0016 [−0.0026 , −0.0006]
GOYA −0.0011 [−0.0021 , 0.0000]
IOW 0.0030 [−0.0073 , 0.0133]
MEDALL −0.0006 [−0.0016 , 0.0004]
MOBA1 −0.0014 [−0.0021 , −0.0007]
MOBA2 −0.0020 [−0.0029 , −0.0012]
MOBA3 0.0007 [−0.0020 , 0.0034]
NFCS −0.0010 [−0.0019 , −0.0001]
NEST −0.0001 [−0.0007 , 0.0005]
NHBCS −0.0022 [−0.0039 , −0.0005]
RICHS −0.0007 [−0.0026 , 0.0012]
VIVA −0.0009 [−0.0021 , 0.0003]
Fixed−effects meta−analysis −0.0010 [−0.0013 , −0.0007]
Random−effects meta−analysis −0.0010 [−0.0013 , −0.0007]
−0.0100 0.0000 0.0100
Change in offspring DNA methylation per1kg/m2 increase in maternal BMI

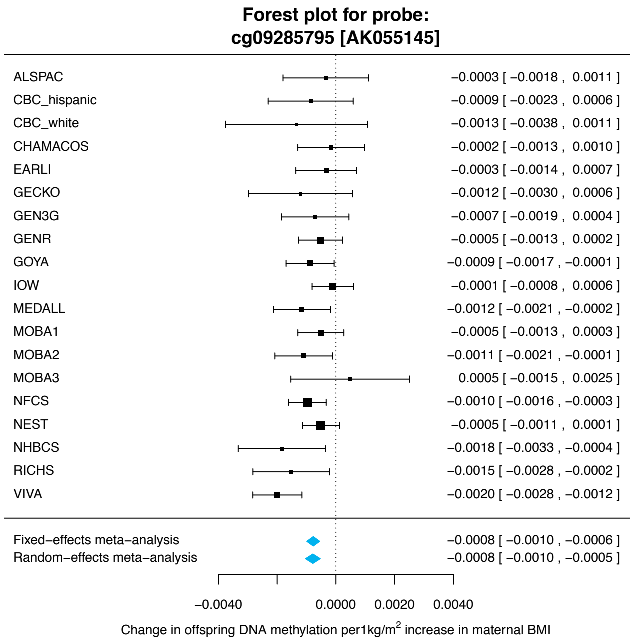
Forest plot for probe:
cg09285795 [AK055145]
ALSPAC −0.0003 [−0.0018 , 0.0011]
CBC_hispanic −0.0009 [−0.0023 , 0.0006]
CBC_white −0.0013 [−0.0038 , 0.0011]
CHAMACOS −0.0002 [−0.0013 , 0.0010]
EARLI −0.0003 [−0.0014 , 0.0007]
GECKO −0.0012 [−0.0030 , 0.0006]
GEN3G −0.0007 [−0.0019 , 0.0004]
GENR −0.0005 [−0.0013 , 0.0002]
GOYA −0.0009 [−0.0017 , −0.0001]
IOW −0.0001 [−0.0008 , 0.0006]
MEDALL −0.0012 [−0.0021 , −0.0002]
MOBA1 −0.0005 [−0.0013 , 0.0003]
MOBA2 −0.0011 [−0.0021 , −0.0001]
MOBA3 0.0005 [−0.0015 , 0.0025]
NFCS −0.0010 [−0.0016 , −0.0003]
NEST −0.0005 [−0.0011 , 0.0001]
NHBCS −0.0018 [−0.0033 , −0.0004]
RICHS −0.0015 [−0.0028 , −0.0002]
VIVA −0.0020 [−0.0028 , −0.0012]
Fixed−effects meta−analysis −0.0008 [−0.0010 , −0.0006]
Random−effects meta−analysis −0.0008 [−0.0010 , −0.0005]
−0.0040 0.0000 0.0020 0.0040
Change in offspring DNA methylation per1kg/m2 increase in maternal BMI

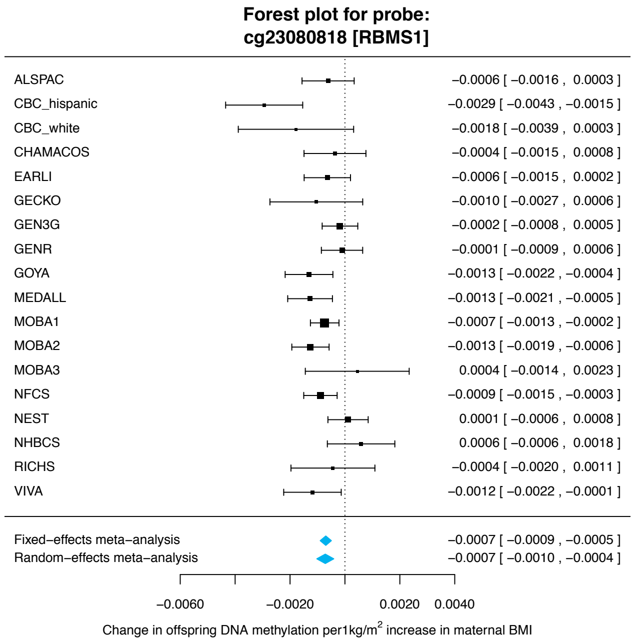
Forest plot for probe:
cg23080818 [RBMS1]
ALSPAC −0.0006 [−0.0016 , 0.0003]
CBC_hispanic −0.0029 [−0.0043 , −0.0015]
CBC_white −0.0018 [−0.0039 , 0.0003]
CHAMACOS −0.0004 [−0.0015 , 0.0008]
EARLI −0.0006 [−0.0015 , 0.0002]
GECKO −0.0010 [−0.0027 , 0.0006]
GEN3G −0.0002 [−0.0008 , 0.0005]
GENR −0.0001 [−0.0009 , 0.0006]
GOYA −0.0013 [−0.0022 , −0.0004]
MEDALL −0.0013 [−0.0021 , −0.0005]
MOBA1 −0.0007 [−0.0013 , −0.0002]
MOBA2 −0.0013 [−0.0019 , −0.0006]
MOBA3 0.0004 [−0.0014 , 0.0023]
NFCS −0.0009 [−0.0015 , −0.0003]
NEST 0.0001 [−0.0006 , 0.0008]
NHBCS 0.0006 [−0.0006 , 0.0018]
RICHS −0.0004 [−0.0020 , 0.0011]
VIVA −0.0012 [−0.0022 , −0.0001]
Fixed−effects meta−analysis −0.0007 [−0.0009 , −0.0005]
Random−effects meta−analysis −0.0007 [−0.0010 , −0.0004]
−0.0060 −0.0020 0.0020 0.0040
Change in offspring DNA methylation per1kg/m2 increase in maternal BMI

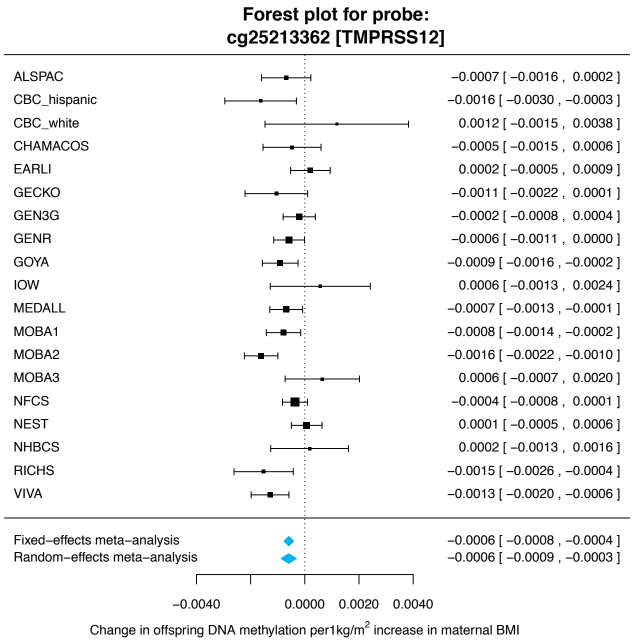
Forest plot for probe:
cg25213362 [TMPRSS12]
ALSPAC −0.0007 [−0.0016 , 0.0002]
CBC_hispanic −0.0016 [−0.0030 , −0.0003]
CBC_white 0.0012 [−0.0015 , 0.0038]
CHAMACOS −0.0005 [−0.0015 , 0.0006]
EARLI 0.0002 [−0.0005 , 0.0009]
GECKO −0.0011 [−0.0022 , 0.0001]
GEN3G −0.0002 [−0.0008 , 0.0004]
GENR −0.0006 [−0.0011 , 0.0000]
GOYA −0.0009 [−0.0016 , −0.0002]
IOW 0.0006 [−0.0013 , 0.0024]
MEDALL −0.0007 [−0.0013 , −0.0001]
MOBA1 −0.0008 [−0.0014 , −0.0002]
MOBA2 −0.0016 [−0.0022 , −0.0010]
MOBA3 0.0006 [−0.0007 , 0.0020]
NFCS −0.0004 [−0.0008 , 0.0001]
NEST 0.0001 [−0.0005 , 0.0006]
NHBCS 0.0002 [−0.0013 , 0.0016]
RICHS −0.0015 [−0.0026 , −0.0004]
VIVA −0.0013 [−0.0020 , −0.0006]
Fixed−effects meta−analysis −0.0006 [−0.0008 , −0.0004]
Random−effects meta−analysis −0.0006 [−0.0009 , −0.0003]
−0.0040 0.0000 0.0020 0.0040
Change in offspring DNA methylation per1kg/m2 increase in maternal BMI

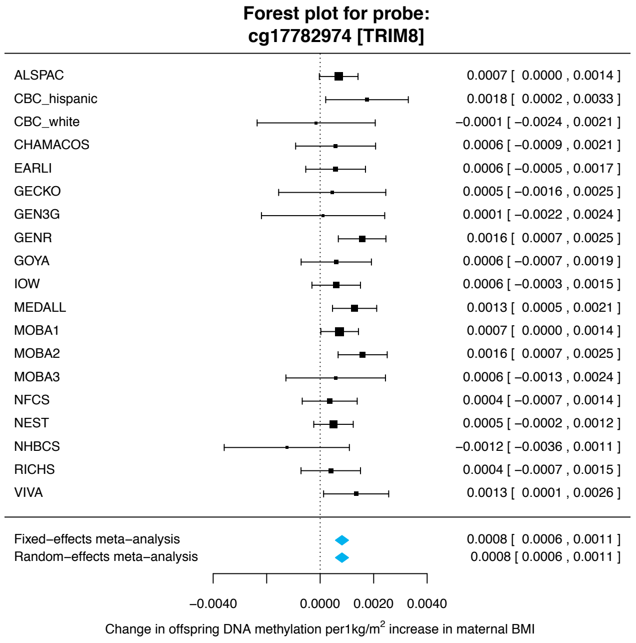
Forest plot for probe:
cg17782974 [TRIM8]
ALSPAC 0.0007 [0.0000 , 0.0014]
CBC_hispanic 0.0018 [0.0002 , 0.0033]
CBC_white −0.0001 [−0.0024 , 0.0021]
CHAMACOS 0.0006 [−0.0009 , 0.0021]
EARLI 0.0006 [−0.0005 , 0.0017]
GECKO 0.0005 [−0.0016 , 0.0025]
GEN3G 0.0001 [−0.0022 , 0.0024]
GENR 0.0016 [0.0007 , 0.0025]
GOYA 0.0006 [−0.0007 , 0.0019]
IOW 0.0006 [−0.0003 , 0.0015]
MEDALL 0.0013 [0.0005 , 0.0021]
MOBA1 0.0007 [0.0000 , 0.0014]
MOBA2 0.0016 [0.0007 , 0.0025]
MOBA3 0.0006 [−0.0013 , 0.0024]
NFCS 0.0004 [−0.0007 , 0.0014]
NEST 0.0005 [−0.0002 , 0.0012]
NHBCS −0.0012 [−0.0036 , 0.0011]
RICHS 0.0004 [−0.0007 , 0.0015]
VIVA 0.0013 [0.0001 , 0.0026]
Fixed−effects meta−analysis 0.0008 [0.0006 , 0.0011]
Random−effects meta−analysis 0.0008 [0.0006 , 0.0011]
−0.0040 0.0000 0.0020 0.0040
Change in offspring DNA methylation per1kg/m2 increase in maternal BMI

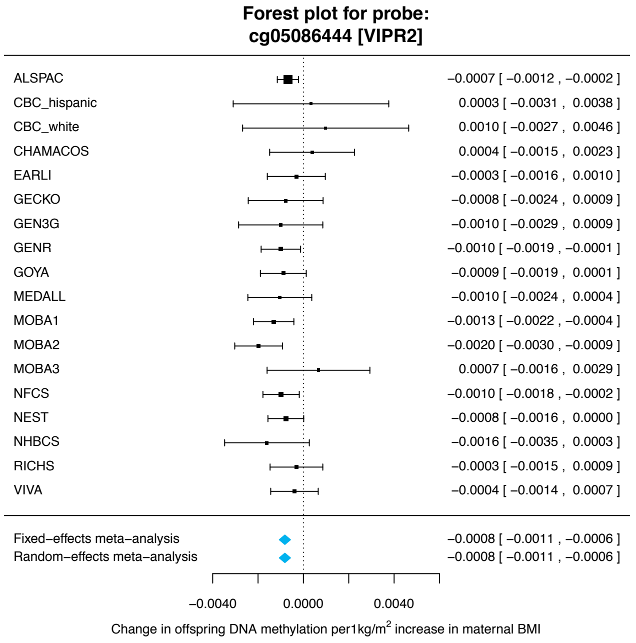
Forest plot for probe:
cg05086444 [VIPR2]
ALSPAC −0.0007 [−0.0012 , −0.0002]
CBC_hispanic 0.0003 [−0.0031 , 0.0038]
CBC_white 0.0010 [−0.0027 , 0.0046]
CHAMACOS 0.0004 [−0.0015 , 0.0023]
EARLI −0.0003 [−0.0016 , 0.0010]
GECKO −0.0008 [−0.0024 , 0.0009]
GEN3G −0.0010 [−0.0029 , 0.0009]
GENR −0.0010 [−0.0019 , −0.0001]
GOYA −0.0009 [−0.0019 , 0.0001]
MEDALL −0.0010 [−0.0024 , 0.0004]
MOBA1 −0.0013 [−0.0022 , −0.0004]
MOBA2 −0.0020 [−0.0030 , −0.0009]
MOBA3 0.0007 [−0.0016 , 0.0029]
NFCS −0.0010 [−0.0018 , −0.0002]
NEST −0.0008 [−0.0016 , 0.0000]
NHBCS −0.0016 [−0.0035 , 0.0003]
RICHS −0.0003 [−0.0015 , 0.0009]
VIVA −0.0004 [−0.0014 , 0.0007]
Fixed−effects meta−analysis −0.0008 [−0.0011 , −0.0006]
Random−effects meta−analysis −0.0008 [−0.0011 , −0.0006]
−0.0040 0.0000 0.0040
Change in offspring DNA methylation per1kg/m2 increase in maternal BMI

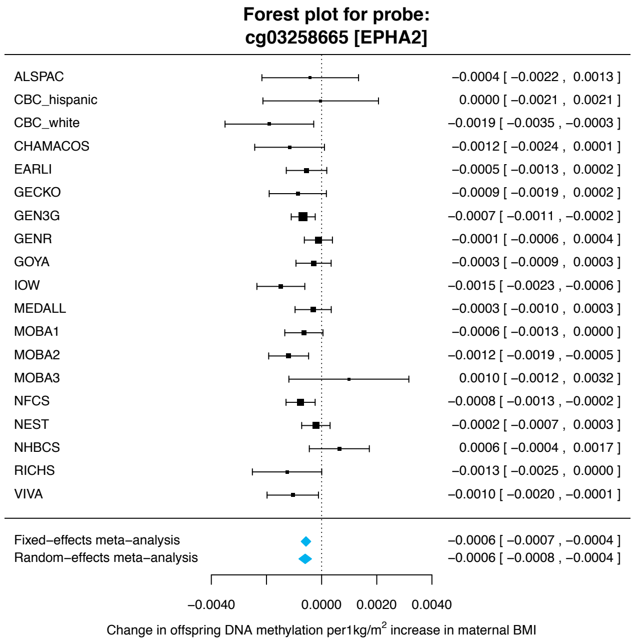
Forest plot for probe:
cg03258665 [EPHA2]
ALSPAC −0.0004 [−0.0022 , 0.0013]
CBC_hispanic 0.0000 [−0.0021 , 0.0021]
CBC_white −0.0019 [−0.0035 , −0.0003]
CHAMACOS −0.0012 [−0.0024 , 0.0001]
EARLI −0.0005 [−0.0013 , 0.0002]
GECKO −0.0009 [−0.0019 , 0.0002]
GEN3G −0.0007 [−0.0011 , −0.0002]
GENR −0.0001 [−0.0006 , 0.0004]
GOYA −0.0003 [−0.0009 , 0.0003]
IOW −0.0015 [−0.0023 , −0.0006]
MEDALL −0.0003 [−0.0010 , 0.0003]
MOBA1 −0.0006 [−0.0013 , 0.0000]
MOBA2 −0.0012 [−0.0019 , −0.0005]
MOBA3 0.0010 [−0.0012 , 0.0032]
NFCS −0.0008 [−0.0013 , −0.0002]
NEST −0.0002 [−0.0007 , 0.0003]
NHBCS 0.0006 [−0.0004 , 0.0017]
RICHS −0.0013 [−0.0025 , 0.0000]
VIVA −0.0010 [−0.0020 , −0.0001]
Fixed−effects meta−analysis −0.0006 [−0.0007 , −0.0004]
Random−effects meta−analysis −0.0006 [−0.0008 , −0.0004]
−0.0040
0.0000
0.0020
0.0040
Change in offspring DNA methylation per1kg/m2 increase in maternal BMI

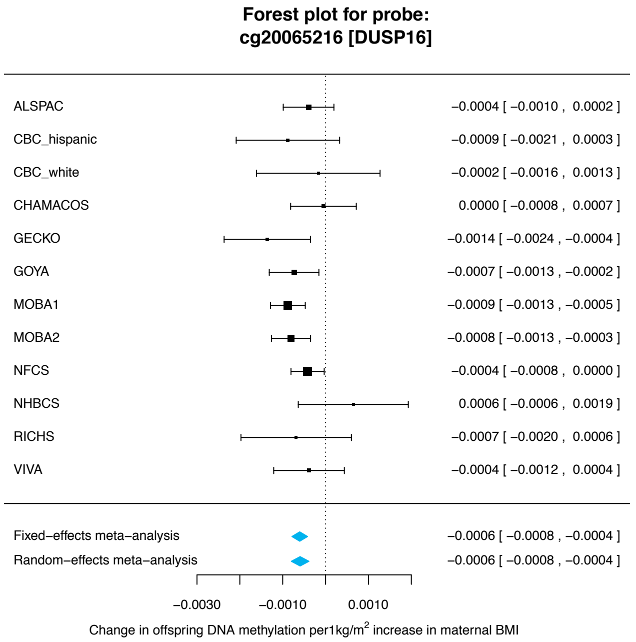
Forest plot for probe:
cg20065216 [DUSP16]
ALSPAC −0.0004 [−0.0010 , 0.0002]
CBC_hispanic −0.0009 [−0.0021 , 0.0003]
CBC_white −0.0002 [−0.0016 , 0.0013]
CHAMACOS 0.0000 [−0.0008 , 0.0007]
GECKO −0.0014 [−0.0024 , −0.0004]
GOYA −0.0007 [−0.0013 , −0.0002]
MOBA1 −0.0009 [−0.0013 , −0.0005]
MOBA2 −0.0008 [−0.0013 , −0.0003]
NFCS −0.0004 [−0.0008 , 0.0000]
NHBCS 0.0006 [−0.0006 , 0.0019]
RICHS −0.0007 [−0.0020 , 0.0006]
VIVA −0.0004 [−0.0012 , 0.0004]
Fixed−effects meta−analysis −0.0006 [−0.0008 , −0.0004]
Random−effects meta−analysis −0.0006 [−0.0008 , −0.0004]
−0.0030
−0.0010
0.0010
Change in offspring DNA methylation per1kg/m2 increase in maternal BMI

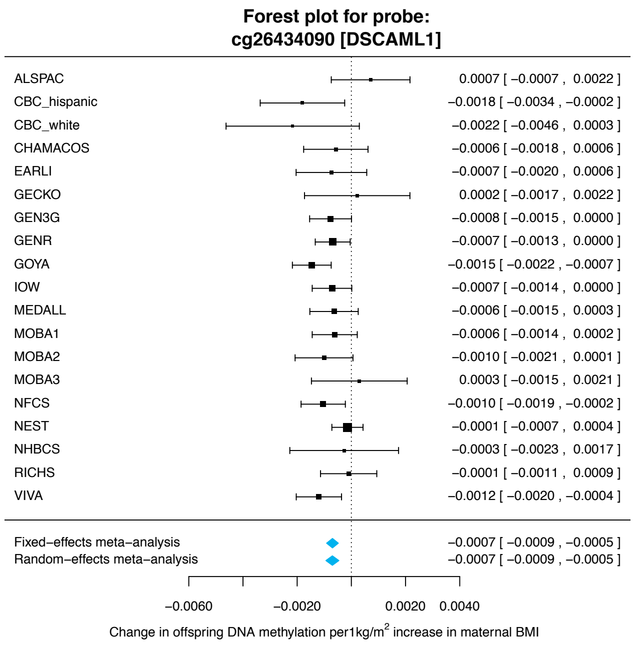
Forest plot for probe:
cg26434090 [DSCAML1]
ALSPAC 0.0007 [−0.0007 , 0.0022]
CBC_hispanic −0.0018 [−0.0034 , −0.0002]
CBC_white −0.0022 [−0.0046 , 0.0003]
CHAMACOS −0.0006 [−0.0018 , 0.0006]
EARLI −0.0007 [−0.0020 , 0.0006]
GECKO 0.0002 [−0.0017 , 0.0022]
GEN3G −0.0008 [−0.0015 , 0.0000]
GENR −0.0007 [−0.0013 , 0.0000]
GOYA −0.0015 [−0.0022 , −0.0007]
IOW −0.0007 [−0.0014 , 0.0000]
MEDALL −0.0006 [−0.0015 , 0.0003]
MOBA1 −0.0006 [−0.0014 , 0.0002]
MOBA2 −0.0010 [−0.0021 , 0.0001]
MOBA3 0.0003 [−0.0015 , 0.0021]
NFCS −0.0010 [−0.0019 , −0.0002]
NEST −0.0001 [−0.0007 , 0.0004]
NHBCS −0.0003 [−0.0023 , 0.0017]
RICHS −0.0001 [−0.0011 , 0.0009]
VIVA −0.0012 [−0.0020 , −0.0004]
Fixed−effects meta−analysis −0.0007 [−0.0009 , −0.0005]
Random−effects meta−analysis −0.0007 [−0.0009 , −0.0005]
−0.0060
−0.0020
0.0020
0.0040
Change in offspring DNA methylation per1kg/m2 increase in maternal BMI

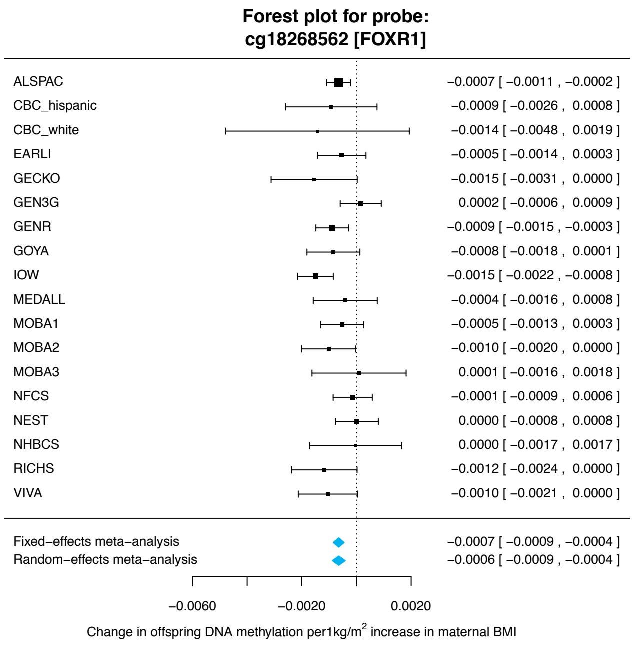
Forest plot for probe:
cg18268562 [FOXR1]
ALSPAC −0.0007 [−0.0011 , −0.0002]
CBC_hispanic −0.0009 [−0.0026 , 0.0008]
CBC_white −0.0014 [−0.0048 , 0.0019]
EARLI −0.0005 [−0.0014 , 0.0003]
GECKO −0.0015 [−0.0031 , 0.0000]
GEN3G 0.0002 [−0.0006 , 0.0009]
GENR −0.0009 [−0.0015 , −0.0003]
GOYA −0.0008 [−0.0018 , 0.0001]
IOW −0.0015 [−0.0022 , −0.0008]
MEDALL −0.0004 [−0.0016 , 0.0008]
MOBA1 −0.0005 [−0.0013 , 0.0003]
MOBA2 −0.0010 [−0.0020 , 0.0000]
MOBA3 0.0001 [−0.0016 , 0.0018]
NFCS −0.0001 [−0.0009 , 0.0006]
NEST 0.0000 [−0.0008 , 0.0008]
NHBCS 0.0000 [−0.0017 , 0.0017]
RICHS −0.0012 [−0.0024 , 0.0000]
VIVA −0.0010 [−0.0021 , 0.0000]
Fixed−effects meta−analysis −0.0007 [−0.0009 , −0.0004]
Random−effects meta−analysis −0.0006 [−0.0009 , −0.0004]
−0.0060
−0.0020
0.0020
Change in offspring DNA methylation per1kg/m2 increase in maternal BMI

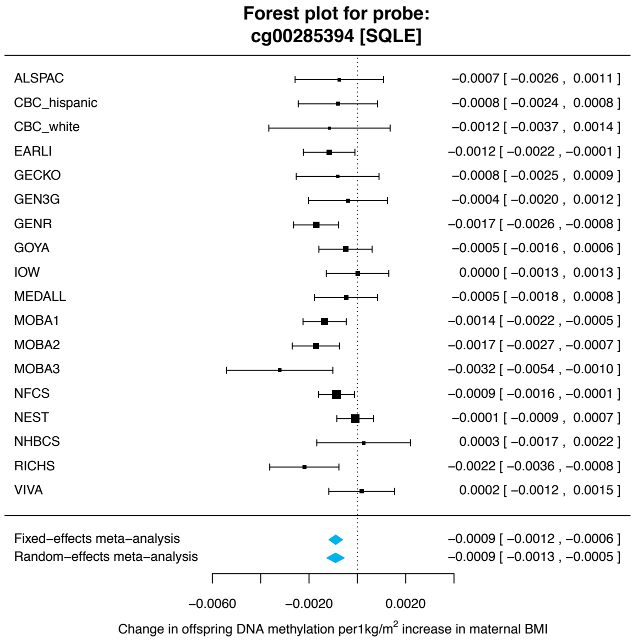
Forest plot for probe:
cg00285394 [SQLE]
ALSPAC −0.0007 [−0.0026 , 0.0011]
CBC_hispanic −0.0008 [−0.0024 , 0.0008]
CBC_white −0.0012 [−0.0037 , 0.0014]
EARLI −0.0012 [−0.0022 , −0.0001]
GECKO −0.0008 [−0.0025 , 0.0009]
GEN3G −0.0004 [−0.0020 , 0.0012]
GENR −0.0017 [−0.0026 , −0.0008]
GOYA −0.0005 [−0.0016 , 0.0006]
IOW 0.0000 [−0.0013 , 0.0013]
MEDALL −0.0005 [−0.0018 , 0.0008]
MOBA1 −0.0014 [−0.0022 , −0.0005]
MOBA2 −0.0017 [−0.0027 , −0.0007]
MOBA3 −0.0032 [−0.0054 , −0.0010]
NFCS −0.0009 [−0.0016 , −0.0001]
NEST −0.0001 [−0.0009 , 0.0007]
NHBCS 0.0003 [−0.0017 , 0.0022]
RICHS −0.0022 [−0.0036 , −0.0008]
VIVA 0.0002 [−0.0012 , 0.0015]
Fixed−effects meta−analysis −0.0009 [−0.0012 , −0.0006]
Random−effects meta−analysis −0.0009 [−0.0013 , −0.0005]
−0.0060
−0.0020
0.0020
Change in offspring DNA methylation per1kg/m2 increase in maternal BMI

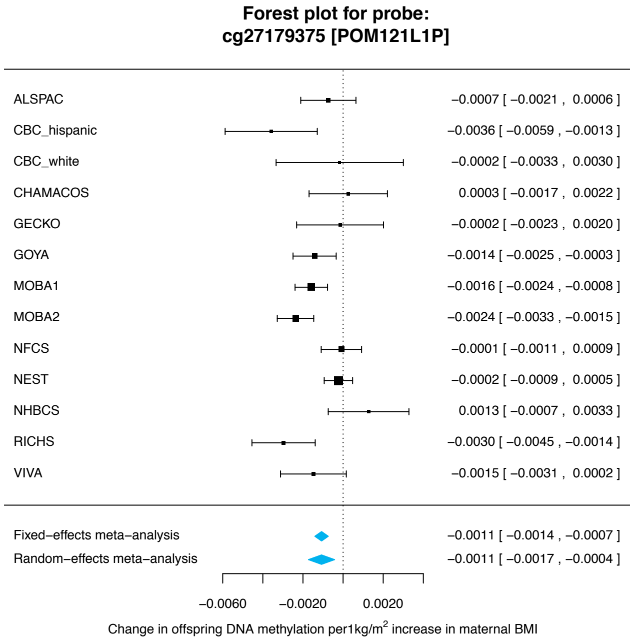
Forest plot for probe:
cg27179375 [POM121L1P]
ALSPAC −0.0007 [−0.0021 , 0.0006]
CBC_hispanic −0.0036 [−0.0059 , −0.0013]
CBC_white −0.0002 [−0.0033 , 0.0030]
CHAMACOS 0.0003 [−0.0017 , 0.0022]
GECKO −0.0002 [−0.0023 , 0.0020]
GOYA −0.0014 [−0.0025 , −0.0003]
MOBA1 −0.0016 [−0.0024 , −0.0008]
MOBA2 −0.0024 [−0.0033 , −0.0015]
NFCS −0.0001 [−0.0011 , 0.0009]
NEST −0.0002 [−0.0009 , 0.0005]
NHBCS 0.0013 [−0.0007 , 0.0033]
RICHS −0.0030 [−0.0045 , −0.0014]
VIVA −0.0015 [−0.0031 , 0.0002]
Fixed−effects meta−analysis −0.0011 [−0.0014 , −0.0007]
Random−effects meta−analysis −0.0011 [−0.0017 , −0.0004]
−0.0060
−0.0020
0.0020
Change in offspring DNA methylation per1kg/m2 increase in maternal BMI

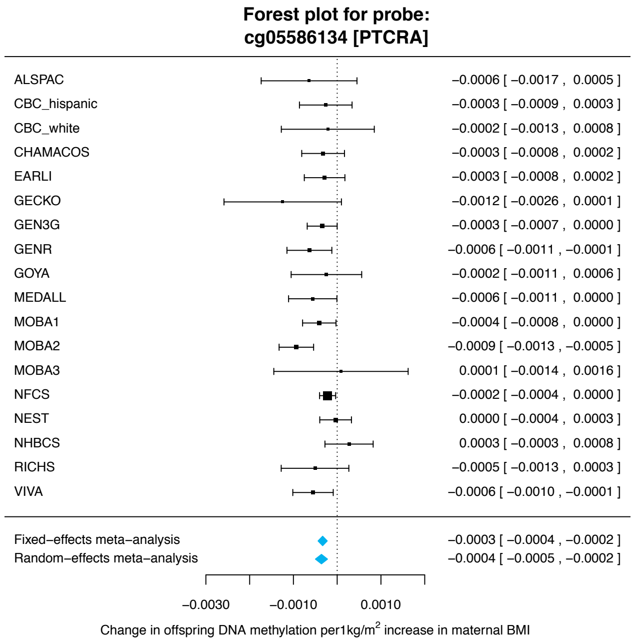
Forest plot for probe:
cg05586134 [PTCRA]
ALSPAC −0.0006 [−0.0017 , 0.0005]
CBC_hispanic −0.0003 [−0.0009 , 0.0003]
CBC_white −0.0002 [−0.0013 , 0.0008]
CHAMACOS −0.0003 [−0.0008 , 0.0002]
EARLI −0.0003 [−0.0008 , 0.0002]
GECKO −0.0012 [−0.0026 , 0.0001]
GEN3G −0.0003 [−0.0007 , 0.0000]
GENR −0.0006 [−0.0011 , −0.0001]
GOYA −0.0002 [−0.0011 , 0.0006]
MEDALL −0.0006 [−0.0011 , 0.0000]
MOBA1 −0.0004 [−0.0008 , 0.0000]
MOBA2 −0.0009 [−0.0013 , −0.0005]
MOBA3 0.0001 [−0.0014 , 0.0016]
NFCS −0.0002 [−0.0004 , 0.0000]
NEST 0.0000 [−0.0004 , 0.0003]
NHBCS 0.0003 [−0.0003 , 0.0008]
RICHS −0.0005 [−0.0013 , 0.0003]
VIVA −0.0006 [−0.0010 , −0.0001]
Fixed−effects meta−analysis −0.0003 [−0.0004 , −0.0002]
Random−effects meta−analysis −0.0004 [−0.0005 , −0.0002]
−0.0030
−0.0010
0.0010
Change in offspring DNA methylation per1kg/m2 increase in maternal BMI

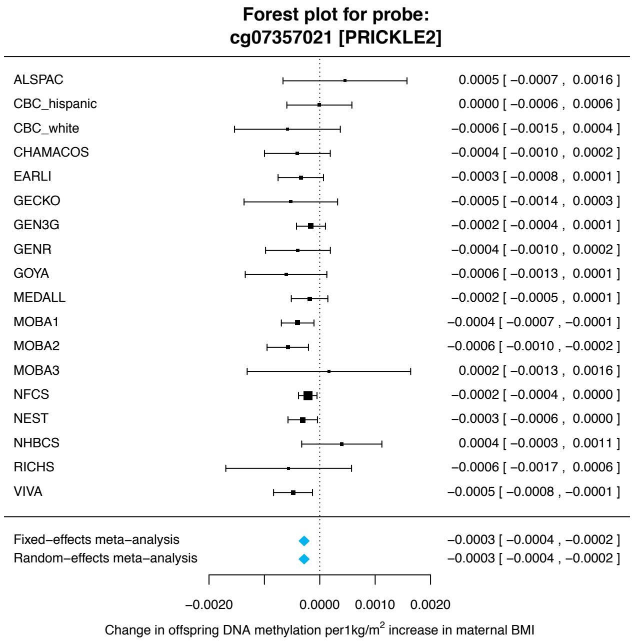
Forest plot for probe:
cg07357021 [PRICKLE2]
ALSPAC 0.0005 [−0.0007 , 0.0016]
CBC_hispanic 0.0000 [−0.0006 , 0.0006]
CBC_white −0.0006 [−0.0015 , 0.0004]
CHAMACOS −0.0004 [−0.0010 , 0.0002]
EARLI −0.0003 [−0.0008 , 0.0001]
GECKO −0.0005 [−0.0014 , 0.0003]
GEN3G −0.0002 [−0.0004 , 0.0001]
GENR −0.0004 [−0.0010 , 0.0002]
GOYA −0.0006 [−0.0013 , 0.0001]
MEDALL −0.0002 [−0.0005 , 0.0001]
MOBA1 −0.0004 [−0.0007 , −0.0001]
MOBA2 −0.0006 [−0.0010 , −0.0002]
MOBA3 0.0002 [−0.0013 , 0.0016]
NFCS −0.0002 [−0.0004 , 0.0000]
NEST −0.0003 [−0.0006 , 0.0000]
NHBCS 0.0004 [−0.0003 , 0.0011]
RICHS −0.0006 [−0.0017 , 0.0006]
VIVA −0.0005 [−0.0008 , −0.0001]
Fixed−effects meta−analysis −0.0003 [−0.0004 , −0.0002]
Random−effects meta−analysis −0.0003 [−0.0004 , −0.0002]
−0.0020
0.0000
0.0010
0.0020
Change in offspring DNA methylation per1kg/m2 increase in maternal BMI

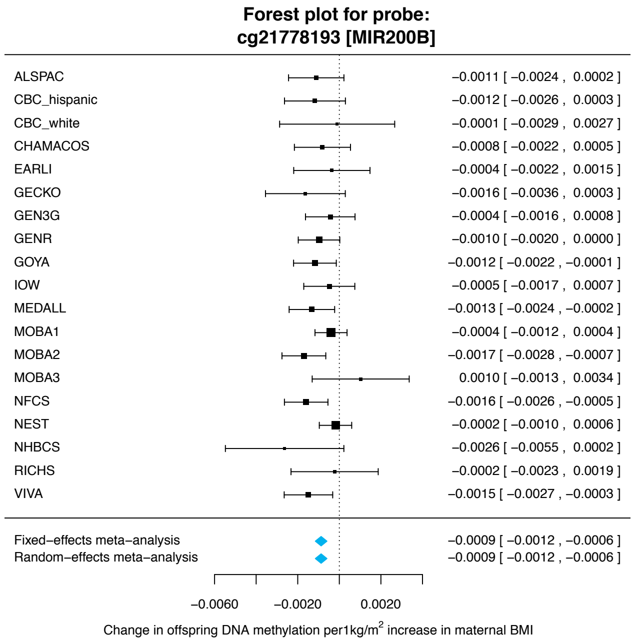
Forest plot for probe:
cg21778193 [MIR200B]
ALSPAC −0.0011 [−0.0024 , 0.0002]
CBC_hispanic −0.0012 [−0.0026 , 0.0003]
CBC_white −0.0001 [−0.0029 , 0.0027]
CHAMACOS −0.0008 [−0.0022 , 0.0005]
EARLI −0.0004 [−0.0022 , 0.0015]
GECKO −0.0016 [−0.0036 , 0.0003]
GEN3G −0.0004 [−0.0016 , 0.0008]
GENR −0.0010 [−0.0020 , 0.0000]
GOYA −0.0012 [−0.0022 , −0.0001]
IOW −0.0005 [−0.0017 , 0.0007]
MEDALL −0.0013 [−0.0024 , −0.0002]
MOBA1 −0.0004 [−0.0012 , 0.0004]
MOBA2 −0.0017 [−0.0028 , −0.0007]
MOBA3 0.0010 [−0.0013 , 0.0034]
NFCS −0.0016 [−0.0026 , −0.0005]
NEST −0.0002 [−0.0010 , 0.0006]
NHBCS −0.0026 [−0.0055 , 0.0002]
RICHS −0.0002 [−0.0023 , 0.0019]
VIVA −0.0015 [−0.0027 , −0.0003]
Fixed−effects meta−analysis −0.0009 [−0.0012 , −0.0006]
Random−effects meta−analysis −0.0009 [−0.0012 , −0.0006]
−0.0060
−0.0020
0.0020
Change in offspring DNA methylation per1kg/m2 increase in maternal BMI

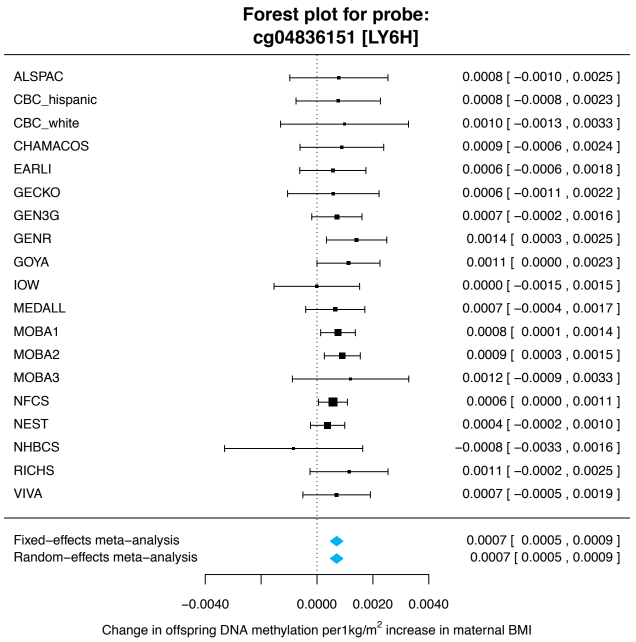
Forest plot for probe:
cg04836151 [LY6H]
ALSPAC 0.0008 [−0.0010 , 0.0025]
CBC_hispanic 0.0008 [−0.0008 , 0.0023]
CBC_white 0.0010 [−0.0013 , 0.0033]
CHAMACOS 0.0009 [−0.0006 , 0.0024]
EARLI 0.0006 [−0.0006 , 0.0018]
GECKO 0.0006 [−0.0011 , 0.0022]
GEN3G 0.0007 [−0.0002 , 0.0016]
GENR 0.0014 [0.0003 , 0.0025]
GOYA 0.0011 [0.0000 , 0.0023]
IOW 0.0000 [−0.0015 , 0.0015]
MEDALL 0.0007 [−0.0004 , 0.0017]
MOBA1 0.0008 [0.0001 , 0.0014]
MOBA2 0.0009 [0.0003 , 0.0015]
MOBA3 0.0012 [−0.0009 , 0.0033]
NFCS 0.0006 [0.0000 , 0.0011]
NEST 0.0004 [−0.0002 , 0.0010]
NHBCS −0.0008 [−0.0033 , 0.0016]
RICHS 0.0011 [−0.0002 , 0.0025]
VIVA 0.0007 [−0.0005 , 0.0019]
Fixed−effects meta−analysis 0.0007 [0.0005 , 0.0009]
Random−effects meta−analysis 0.0007 [0.0005 , 0.0009]
−0.0040
0.0000
0.0020
0.0040
Change in offspring DNA methylation per1kg/m2 increase in maternal BMI

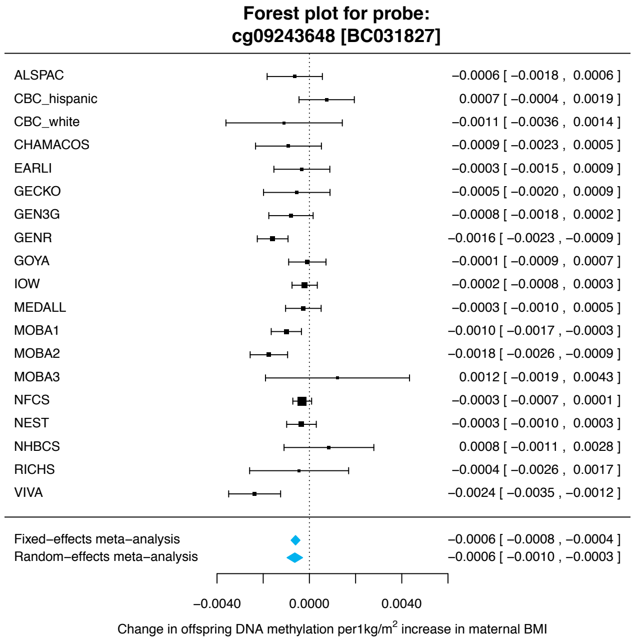
Forest plot for probe:
cg09243648 [BC031827]
ALSPAC −0.0006 [−0.0018 , 0.0006]
CBC_hispanic 0.0007 [−0.0004 , 0.0019]
CBC_white −0.0011 [−0.0036 , 0.0014]
CHAMACOS −0.0009 [−0.0023 , 0.0005]
EARLI −0.0003 [−0.0015 , 0.0009]
GECKO −0.0005 [−0.0020 , 0.0009]
GEN3G −0.0008 [−0.0018 , 0.0002]
GENR −0.0016 [−0.0023 , −0.0009]
GOYA −0.0001 [−0.0009 , 0.0007]
IOW −0.0002 [−0.0008 , 0.0003]
MEDALL −0.0003 [−0.0010 , 0.0005]
MOBA1 −0.0010 [−0.0017 , −0.0003]
MOBA2 −0.0018 [−0.0026 , −0.0009]
MOBA3 0.0012 [−0.0019 , 0.0043]
NFCS −0.0003 [−0.0007 , 0.0001]
NEST −0.0003 [−0.0010 , 0.0003]
NHBCS 0.0008 [−0.0011 , 0.0028]
RICHS −0.0004 [−0.0026 , 0.0017]
VIVA −0.0024 [−0.0035 , −0.0012]
Fixed−effects meta−analysis −0.0006 [−0.0008 , −0.0004]
Random−effects meta−analysis −0.0006 [−0.0010 , −0.0003]
−0.0040
0.0000
0.0040
Change in offspring DNA methylation per1kg/m2 increase in maternal BMI

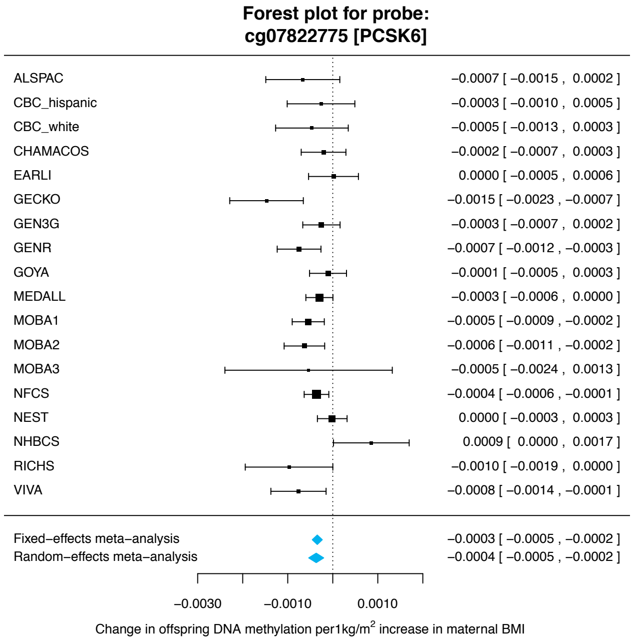
Forest plot for probe:
cg07822775 [PCSK6]
ALSPAC −0.0007 [−0.0015 , 0.0002]
CBC_hispanic −0.0003 [−0.0010 , 0.0005]
CBC_white −0.0005 [−0.0013 , 0.0003]
CHAMACOS −0.0002 [−0.0007 , 0.0003]
EARLI 0.0000 [−0.0005 , 0.0006]
GECKO −0.0015 [−0.0023 , −0.0007]
GEN3G −0.0003 [−0.0007 , 0.0002]
GENR −0.0007 [−0.0012 , −0.0003]
GOYA −0.0001 [−0.0005 , 0.0003]
MEDALL −0.0003 [−0.0006 , 0.0000]
MOBA1 −0.0005 [−0.0009 , −0.0002]
MOBA2 −0.0006 [−0.0011 , −0.0002]
MOBA3 −0.0005 [−0.0024 , 0.0013]
NFCS −0.0004 [−0.0006 , −0.0001]
NEST 0.0000 [−0.0003 , 0.0003]
NHBCS 0.0009 [0.0000 , 0.0017]
RICHS −0.0010 [−0.0019 , 0.0000]
VIVA −0.0008 [−0.0014 , −0.0001]
Fixed−effects meta−analysis −0.0003 [−0.0005 , −0.0002]
Random−effects meta−analysis −0.0004 [−0.0005 , −0.0002]
−0.0030
−0.0010
0.0010
Change in offspring DNA methylation per1kg/m2 increase in maternal BMI

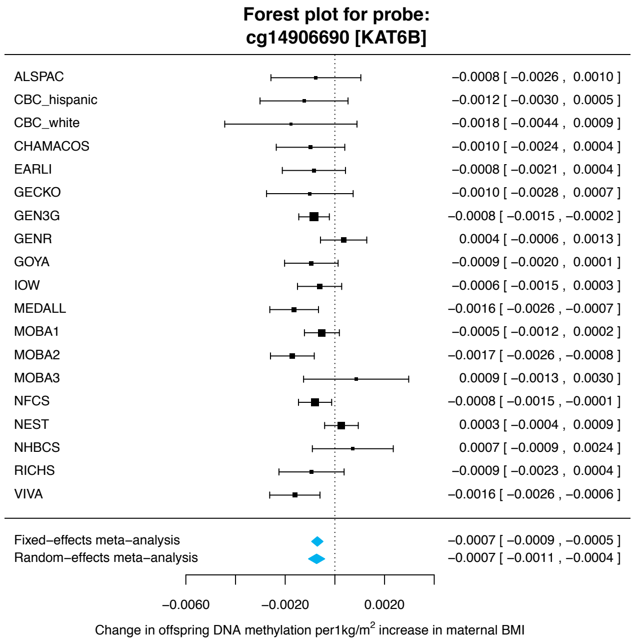
Forest plot for probe:
cg14906690 [KAT6B]
ALSPAC −0.0008 [−0.0026 , 0.0010]
CBC_hispanic −0.0012 [−0.0030 , 0.0005]
CBC_white −0.0018 [−0.0044 , 0.0009]
CHAMACOS −0.0010 [−0.0024 , 0.0004]
EARLI −0.0008 [−0.0021 , 0.0004]
GECKO −0.0010 [−0.0028 , 0.0007]
GEN3G −0.0008 [−0.0015 , −0.0002]
GENR 0.0004 [−0.0006 , 0.0013]
GOYA −0.0009 [−0.0020 , 0.0001]
IOW −0.0006 [−0.0015 , 0.0003]
MEDALL −0.0016 [−0.0026 , −0.0007]
MOBA1 −0.0005 [−0.0012 , 0.0002]
MOBA2 −0.0017 [−0.0026 , −0.0008]
MOBA3 0.0009 [−0.0013 , 0.0030]
NFCS −0.0008 [−0.0015 , −0.0001]
NEST 0.0003 [−0.0004 , 0.0009]
NHBCS 0.0007 [−0.0009 , 0.0024]
RICHS −0.0009 [−0.0023 , 0.0004]
VIVA −0.0016 [−0.0026 , −0.0006]
Fixed−effects meta−analysis −0.0007 [−0.0009 , −0.0005]
Random−effects meta−analysis −0.0007 [−0.0011 , −0.0004]
−0.0060
−0.0020
0.0020
Change in offspring DNA methylation per1kg/m^2 increase in maternal BMI

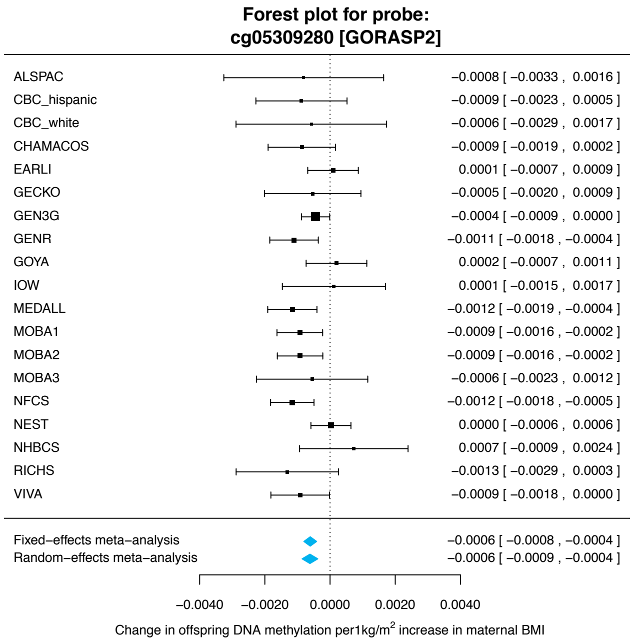
Forest plot for probe:
cg05309280 [GORASP2]
ALSPAC −0.0008 [−0.0033 , 0.0016]
CBC_hispanic −0.0009 [−0.0023 , 0.0005]
CBC_white −0.0006 [−0.0029 , 0.0017]
CHAMACOS −0.0009 [−0.0019 , 0.0002]
EARLI 0.0001 [−0.0007 , 0.0009]
GECKO −0.0005 [−0.0020 , 0.0009]
GEN3G −0.0004 [−0.0009 , 0.0000]
GENR −0.0011 [−0.0018 , −0.0004]
GOYA 0.0002 [−0.0007 , 0.0011]
IOW 0.0001 [−0.0015 , 0.0017]
MEDALL −0.0012 [−0.0019 , −0.0004]
MOBA1 −0.0009 [−0.0016 , −0.0002]
MOBA2 −0.0009 [−0.0016 , −0.0002]
MOBA3 −0.0006 [−0.0023 , 0.0012]
NFCS −0.0012 [−0.0018 , −0.0005]
NEST 0.0000 [−0.0006 , 0.0006]
NHBCS 0.0007 [−0.0009 , 0.0024]
RICHS −0.0013 [−0.0029 , 0.0003]
VIVA −0.0009 [−0.0018 , 0.0000]
Fixed−effects meta−analysis −0.0006 [−0.0008 , −0.0004]
Random−effects meta−analysis −0.0006 [−0.0009 , −0.0004]
−0.0040
−0.0020
0.0000
0.0020
0.0040
Change in offspring DNA methylation per1kg/m^2 increase in maternal BMI

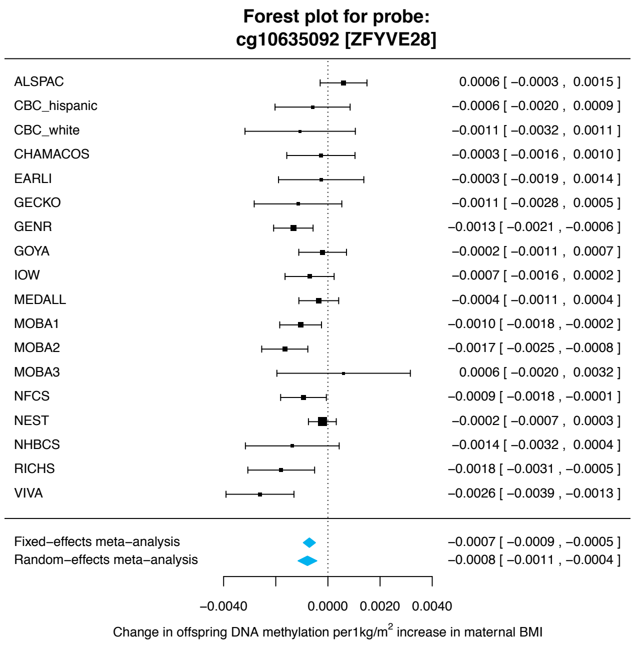
Forest plot for probe:
cg10635092 [ZFYVE28]
ALSPAC 0.0006 [−0.0003 , 0.0015]
CBC_hispanic −0.0006 [−0.0020 , 0.0009]
CBC_white −0.0011 [−0.0032 , 0.0011]
CHAMACOS −0.0003 [−0.0016 , 0.0010]
EARLI −0.0003 [−0.0019 , 0.0014]
GECKO −0.0011 [−0.0028 , 0.0005]
GENR −0.0013 [−0.0021 , −0.0006]
GOYA −0.0002 [−0.0011 , 0.0007]
IOW −0.0007 [−0.0016 , 0.0002]
MEDALL −0.0004 [−0.0011 , 0.0004]
MOBA1 −0.0010 [−0.0018 , −0.0002]
MOBA2 −0.0017 [−0.0025 , −0.0008]
MOBA3 0.0006 [−0.0020 , 0.0032]
NFCS −0.0009 [−0.0018 , −0.0001]
NEST −0.0002 [−0.0007 , 0.0003]
NHBCS −0.0014 [−0.0032 , 0.0004]
RICHS −0.0018 [−0.0031 , −0.0005]
VIVA −0.0026 [−0.0039 , −0.0013]
Fixed−effects meta−analysis −0.0007 [−0.0009 , −0.0005]
Random−effects meta−analysis −0.0008 [−0.0011 , −0.0004]
−0.0040
0.0000
0.0020
0.0040
Change in offspring DNA methylation per1kg/m^2 increase in maternal BMI

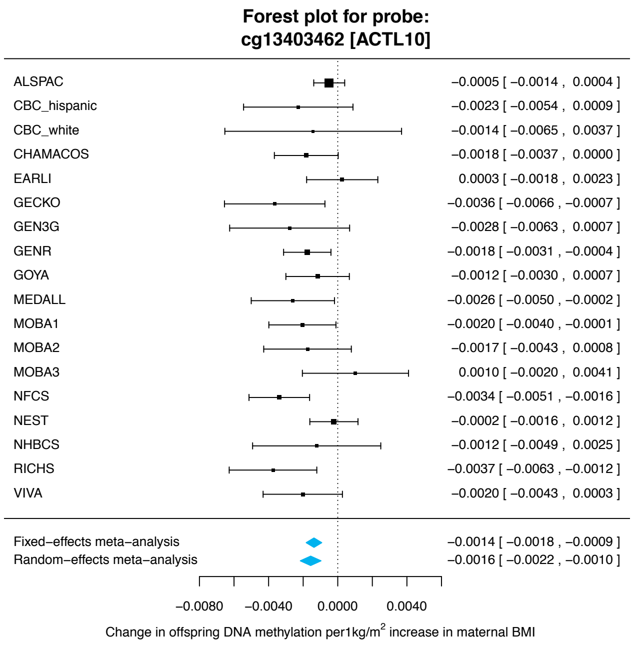
Forest plot for probe:
cg13403462 [ACTL10]
ALSPAC −0.0005 [−0.0014 , 0.0004]
CBC_hispanic −0.0023 [−0.0054 , 0.0009]
CBC_white −0.0014 [−0.0065 , 0.0037]
CHAMACOS −0.0018 [−0.0037 , 0.0000]
EARLI 0.0003 [−0.0018 , 0.0023]
GECKO −0.0036 [−0.0066 , −0.0007]
GEN3G −0.0028 [−0.0063 , 0.0007]
GENR −0.0018 [−0.0031 , −0.0004]
GOYA −0.0012 [−0.0030 , 0.0007]
MEDALL −0.0026 [−0.0050 , −0.0002]
MOBA1 −0.0020 [−0.0040 , −0.0001]
MOBA2 −0.0017 [−0.0043 , 0.0008]
MOBA3 0.0010 [−0.0020 , 0.0041]
NFCS −0.0034 [−0.0051 , −0.0016]
NEST −0.0002 [−0.0016 , 0.0012]
NHBCS −0.0012 [−0.0049 , 0.0025]
RICHS −0.0037 [−0.0063 , −0.0012]
VIVA −0.0020 [−0.0043 , 0.0003]
Fixed−effects meta−analysis −0.0014 [−0.0018 , −0.0009]
Random−effects meta−analysis −0.0016 [−0.0022 , −0.0010]
−0.0080
−0.0040
0.0000
0.0040
Change in offspring DNA methylation per1kg/m^2 increase in maternal BMI

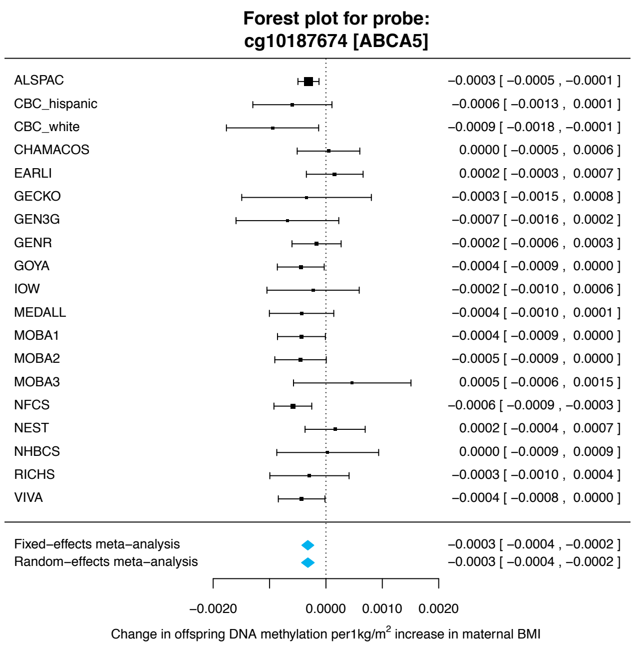
Forest plot for probe:
cg10187674 [ABCA5]
ALSPAC −0.0003 [−0.0005 , −0.0001]
CBC_hispanic −0.0006 [−0.0013 , 0.0001]
CBC_white −0.0009 [−0.0018 , −0.0001]
CHAMACOS 0.0000 [−0.0005 , 0.0006]
EARLI 0.0002 [−0.0003 , 0.0007]
GECKO −0.0003 [−0.0015 , 0.0008]
GEN3G −0.0007 [−0.0016 , 0.0002]
GENR −0.0002 [−0.0006 , 0.0003]
GOYA −0.0004 [−0.0009 , 0.0000]
IOW −0.0002 [−0.0010 , 0.0006]
MEDALL −0.0004 [−0.0010 , 0.0001]
MOBA1 −0.0004 [−0.0009 , 0.0000]
MOBA2 −0.0005 [−0.0009 , 0.0000]
MOBA3 0.0005 [−0.0006 , 0.0015]
NFCS −0.0006 [−0.0009 , −0.0003]
NEST 0.0002 [−0.0004 , 0.0007]
NHBCS 0.0000 [−0.0009 , 0.0009]
RICHS −0.0003 [−0.0010 , 0.0004]
VIVA −0.0004 [−0.0008 , 0.0000]
Fixed−effects meta−analysis −0.0003 [−0.0004 , −0.0002]
Random−effects meta−analysis −0.0003 [−0.0004 , −0.0002]
−0.0020
0.0000
0.0010
0.0020
Change in offspring DNA methylation per1kg/m^2 increase in maternal BMI

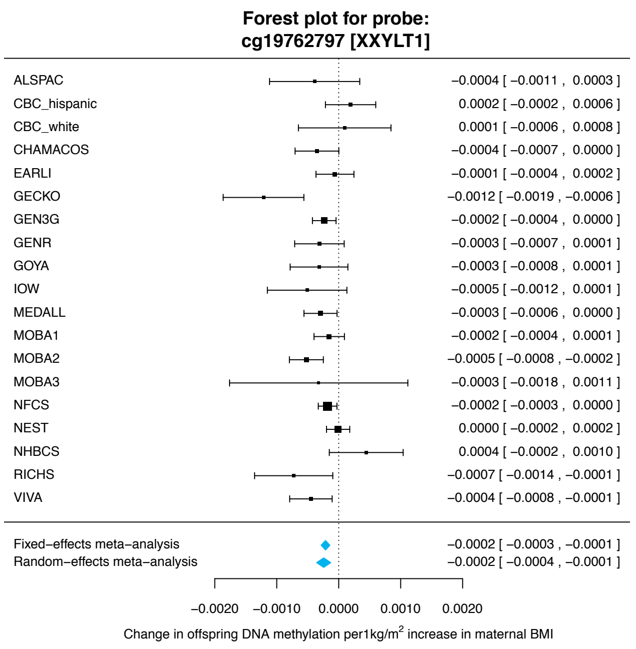
Forest plot for probe:
cg19762797 [XXYLT1]
ALSPAC −0.0004 [−0.0011 , 0.0003]
CBC_hispanic 0.0002 [−0.0002 , 0.0006]
CBC_white 0.0001 [−0.0006 , 0.0008]
CHAMACOS −0.0004 [−0.0007 , 0.0000]
EARLI −0.0001 [−0.0004 , 0.0002]
GECKO −0.0012 [−0.0019 , −0.0006]
GEN3G −0.0002 [−0.0004 , 0.0000]
GENR −0.0003 [−0.0007 , 0.0001]
GOYA −0.0003 [−0.0008 , 0.0001]
IOW −0.0005 [−0.0012 , 0.0001]
MEDALL −0.0003 [−0.0006 , 0.0000]
MOBA1 −0.0002 [−0.0004 , 0.0001]
MOBA2 −0.0005 [−0.0008 , −0.0002]
MOBA3 −0.0003 [−0.0018 , 0.0011]
NFCS −0.0002 [−0.0003 , 0.0000]
NEST 0.0000 [−0.0002 , 0.0002]
NHBCS 0.0004 [−0.0002 , 0.0010]
RICHS −0.0007 [−0.0014 , −0.0001]
VIVA −0.0004 [−0.0008 , −0.0001]
Fixed−effects meta−analysis −0.0002 [−0.0003 , −0.0001]
Random−effects meta−analysis −0.0002 [−0.0004 , −0.0001]
−0.0020
−0.0010
0.0000
0.0010
0.0020
Change in offspring DNA methylation per1kg/m^2 increase in maternal BMI

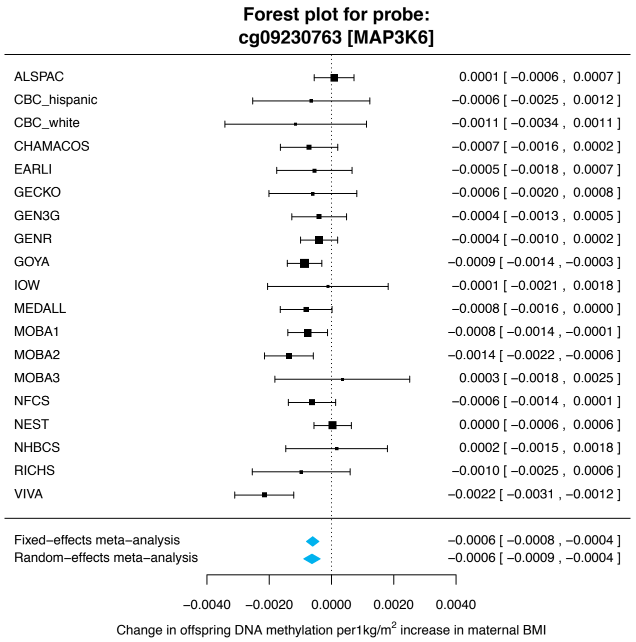
Forest plot for probe:
cg09230763 [MAP3K6]
ALSPAC 0.0001 [−0.0006 , 0.0007]
CBC_hispanic −0.0006 [−0.0025 , 0.0012]
CBC_white −0.0011 [−0.0034 , 0.0011]
CHAMACOS −0.0007 [−0.0016 , 0.0002]
EARLI −0.0005 [−0.0018 , 0.0007]
GECKO −0.0006 [−0.0020 , 0.0008]
GEN3G −0.0004 [−0.0013 , 0.0005]
GENR −0.0004 [−0.0010 , 0.0002]
GOYA −0.0009 [−0.0014 , −0.0003]
IOW −0.0001 [−0.0021 , 0.0018]
MEDALL −0.0008 [−0.0016 , 0.0000]
MOBA1 −0.0008 [−0.0014 , −0.0001]
MOBA2 −0.0014 [−0.0022 , −0.0006]
MOBA3 0.0003 [−0.0018 , 0.0025]
NFCS −0.0006 [−0.0014 , 0.0001]
NEST 0.0000 [−0.0006 , 0.0006]
NHBCS 0.0002 [−0.0015 , 0.0018]
RICHS −0.0010 [−0.0025 , 0.0006]
VIVA −0.0022 [−0.0031 , −0.0012]
Fixed−effects meta−analysis −0.0006 [−0.0008 , −0.0004]
Random−effects meta−analysis −0.0006 [−0.0009 , −0.0004]
−0.0040 −0.0020 0.0000 0.0020 0.0040
Change in offspring DNA methylation per1kg/m2 increase in maternal BMI

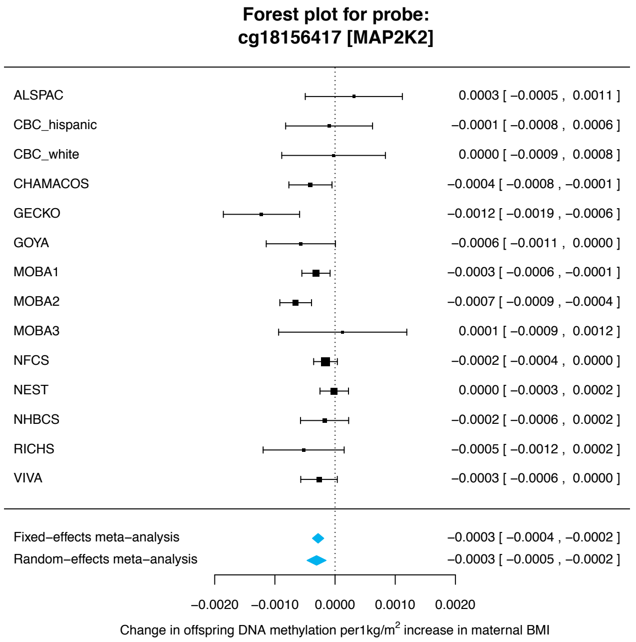
Forest plot for probe:
cg18156417 [MAP2K2]
ALSPAC 0.0003 [−0.0005 , 0.0011]
CBC_hispanic −0.0001 [−0.0008 , 0.0006]
CBC_white 0.0000 [−0.0009 , 0.0008]
CHAMACOS −0.0004 [−0.0008 , −0.0001]
GECKO −0.0012 [−0.0019 , −0.0006]
GOYA −0.0006 [−0.0011 , 0.0000]
MOBA1 −0.0003 [−0.0006 , −0.0001]
MOBA2 −0.0007 [−0.0009 , −0.0004]
MOBA3 0.0001 [−0.0009 , 0.0012]
NFCS −0.0002 [−0.0004 , 0.0000]
NEST 0.0000 [−0.0003 , 0.0002]
NHBCS −0.0002 [−0.0006 , 0.0002]
RICHS −0.0005 [−0.0012 , 0.0002]
VIVA −0.0003 [−0.0006 , 0.0000]
Fixed−effects meta−analysis −0.0003 [−0.0004 , −0.0002]
Random−effects meta−analysis −0.0003 [−0.0005 , −0.0002]
−0.0020 −0.0010 0.0000 0.0010 0.0020
Change in offspring DNA methylation per1kg/m2 increase in maternal BMI

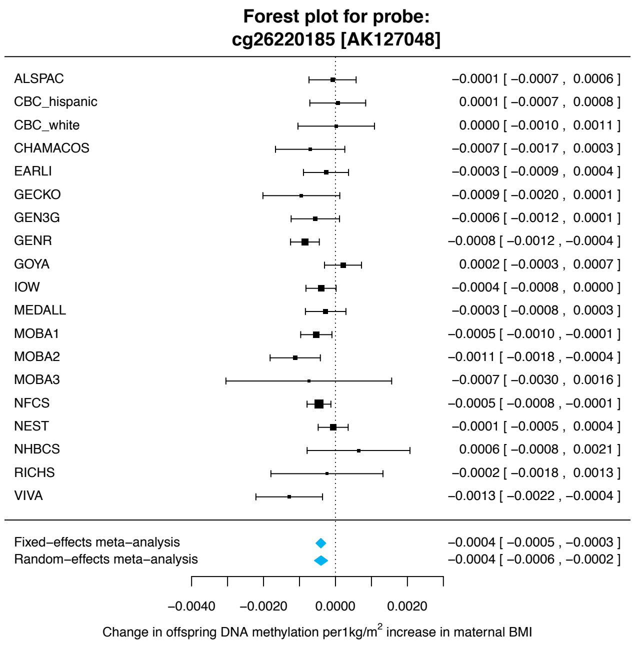
Forest plot for probe:
cg26220185 [AK127048]
ALSPAC −0.0001 [−0.0007 , 0.0006]
CBC_hispanic 0.0001 [−0.0007 , 0.0008]
CBC_white 0.0000 [−0.0010 , 0.0011]
CHAMACOS −0.0007 [−0.0017 , 0.0003]
EARLI −0.0003 [−0.0009 , 0.0004]
GECKO −0.0009 [−0.0020 , 0.0001]
GEN3G −0.0006 [−0.0012 , 0.0001]
GENR −0.0008 [−0.0012 , −0.0004]
GOYA 0.0002 [−0.0003 , 0.0007]
IOW −0.0004 [−0.0008 , 0.0000]
MEDALL −0.0003 [−0.0008 , 0.0003]
MOBA1 −0.0005 [−0.0010 , −0.0001]
MOBA2 −0.0011 [−0.0018 , −0.0004]
MOBA3 −0.0007 [−0.0030 , 0.0016]
NFCS −0.0005 [−0.0008 , −0.0001]
NEST −0.0001 [−0.0005 , 0.0004]
NHBCS 0.0006 [−0.0008 , 0.0021]
RICHS −0.0002 [−0.0018 , 0.0013]
VIVA −0.0013 [−0.0022 , −0.0004]
Fixed−effects meta−analysis −0.0004 [−0.0005 , −0.0003]
Random−effects meta−analysis −0.0004 [−0.0006 , −0.0002]
−0.0040 −0.0020 0.0000 0.0020
Change in offspring DNA methylation per1kg/m2 increase in maternal BMI

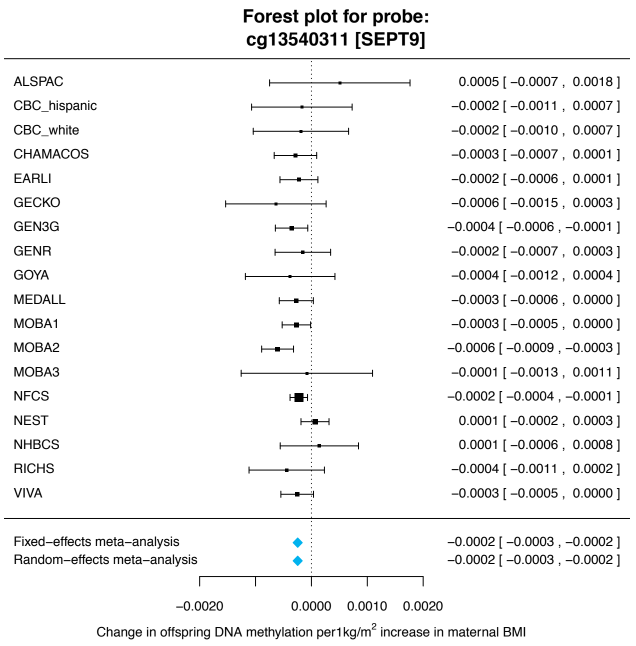
Forest plot for probe:
cg13540311 [SEPT9]
ALSPAC 0.0005 [−0.0007 , 0.0018]
CBC_hispanic −0.0002 [−0.0011 , 0.0007]
CBC_white −0.0002 [−0.0010 , 0.0007]
CHAMACOS −0.0003 [−0.0007 , 0.0001]
EARLI −0.0002 [−0.0006 , 0.0001]
GECKO −0.0006 [−0.0015 , 0.0003]
GEN3G −0.0004 [−0.0006 , −0.0001]
GENR −0.0002 [−0.0007 , 0.0003]
GOYA −0.0004 [−0.0012 , 0.0004]
MEDALL −0.0003 [−0.0006 , 0.0000]
MOBA1 −0.0003 [−0.0005 , 0.0000]
MOBA2 −0.0006 [−0.0009 , −0.0003]
MOBA3 −0.0001 [−0.0013 , 0.0011]
NFCS −0.0002 [−0.0004 , −0.0001]
NEST 0.0001 [−0.0002 , 0.0003]
NHBCS 0.0001 [−0.0006 , 0.0008]
RICHS −0.0004 [−0.0011 , 0.0002]
VIVA −0.0003 [−0.0005 , 0.0000]
Fixed−effects meta−analysis −0.0002 [−0.0003 , −0.0002]
Random−effects meta−analysis −0.0002 [−0.0003 , −0.0002]
−0.0020 0.0000 0.0010 0.0020
Change in offspring DNA methylation per1kg/m2 increase in maternal BMI

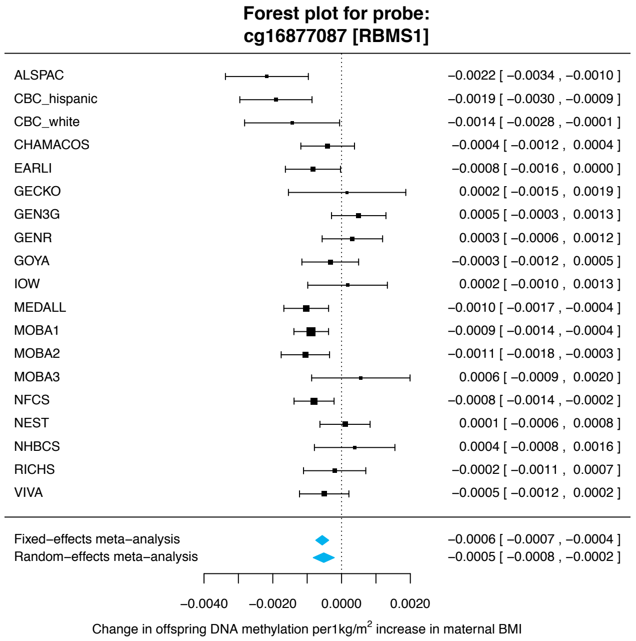
Forest plot for probe:
cg16877087 [RBMS1]
ALSPAC −0.0022 [−0.0034 , −0.0010]
CBC_hispanic −0.0019 [−0.0030 , −0.0009]
CBC_white −0.0014 [−0.0028 , −0.0001]
CHAMACOS −0.0004 [−0.0012 , 0.0004]
EARLI −0.0008 [−0.0016 , 0.0000]
GECKO 0.0002 [−0.0015 , 0.0019]
GEN3G 0.0005 [−0.0003 , 0.0013]
GENR 0.0003 [−0.0006 , 0.0012]
GOYA −0.0003 [−0.0012 , 0.0005]
IOW 0.0002 [−0.0010 , 0.0013]
MEDALL −0.0010 [−0.0017 , −0.0004]
MOBA1 −0.0009 [−0.0014 , −0.0004]
MOBA2 −0.0011 [−0.0018 , −0.0003]
MOBA3 0.0006 [−0.0009 , 0.0020]
NFCS −0.0008 [−0.0014 , −0.0002]
NEST 0.0001 [−0.0006 , 0.0008]
NHBCS 0.0004 [−0.0008 , 0.0016]
RICHS −0.0002 [−0.0011 , 0.0007]
VIVA −0.0005 [−0.0012 , 0.0002]
Fixed−effects meta−analysis −0.0006 [−0.0007 , −0.0004]
Random−effects meta−analysis −0.0005 [−0.0008 , −0.0002]
−0.0040 −0.0020 0.0000 0.0020
Change in offspring DNA methylation per1kg/m2 increase in maternal BMI

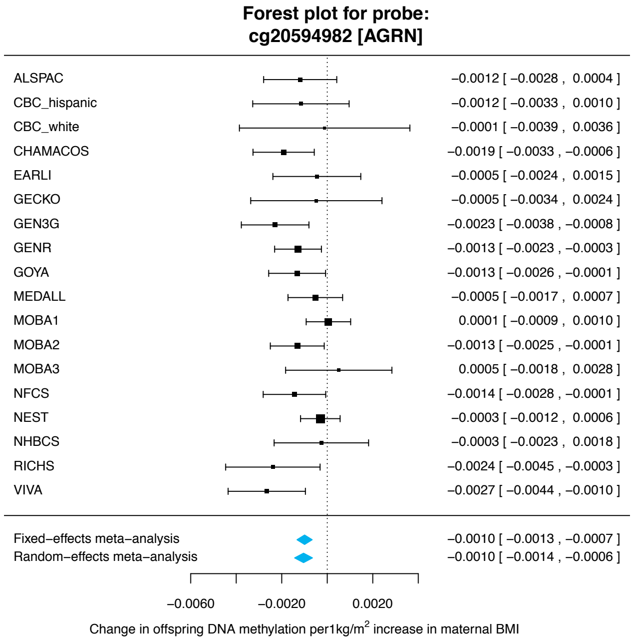
Forest plot for probe:
cg20594982 [AGRN]
ALSPAC −0.0012 [−0.0028 , 0.0004]
CBC_hispanic −0.0012 [−0.0033 , 0.0010]
CBC_white −0.0001 [−0.0039 , 0.0036]
CHAMACOS −0.0019 [−0.0033 , −0.0006]
EARLI −0.0005 [−0.0024 , 0.0015]
GECKO −0.0005 [−0.0034 , 0.0024]
GEN3G −0.0023 [−0.0038 , −0.0008]
GENR −0.0013 [−0.0023 , −0.0003]
GOYA −0.0013 [−0.0026 , −0.0001]
MEDALL −0.0005 [−0.0017 , 0.0007]
MOBA1 0.0001 [−0.0009 , 0.0010]
MOBA2 −0.0013 [−0.0025 , −0.0001]
MOBA3 0.0005 [−0.0018 , 0.0028]
NFCS −0.0014 [−0.0028 , −0.0001]
NEST −0.0003 [−0.0012 , 0.0006]
NHBCS −0.0003 [−0.0023 , 0.0018]
RICHS −0.0024 [−0.0045 , −0.0003]
VIVA −0.0027 [−0.0044 , −0.0010]
Fixed−effects meta−analysis −0.0010 [−0.0013 , −0.0007]
Random−effects meta−analysis −0.0010 [−0.0014 , −0.0006]
−0.0060 −0.0020 0.0020
Change in offspring DNA methylation per1kg/m2 increase in maternal BMI

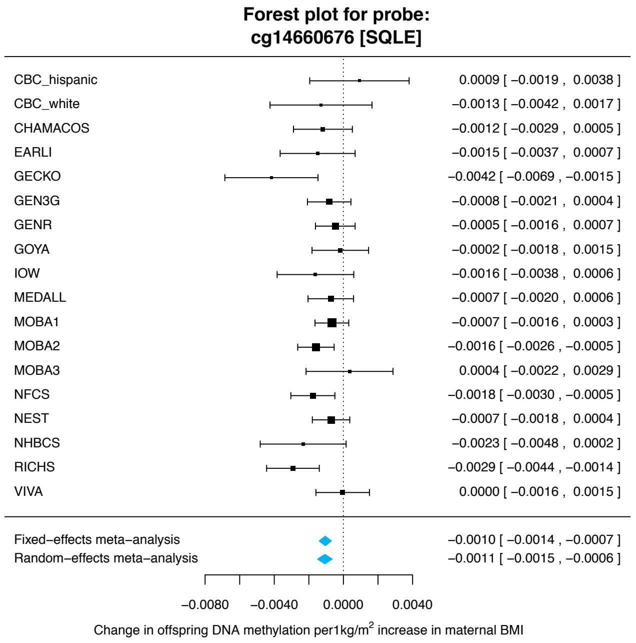
Forest plot for probe:
cg14660676 [SQLE]
CBC_hispanic 0.0009 [−0.0019 , 0.0038]
CBC_white −0.0013 [−0.0042 , 0.0017]
CHAMACOS −0.0012 [−0.0029 , 0.0005]
EARLI −0.0015 [−0.0037 , 0.0007]
GECKO −0.0042 [−0.0069 , −0.0015]
GEN3G −0.0008 [−0.0021 , 0.0004]
GENR −0.0005 [−0.0016 , 0.0007]
GOYA −0.0002 [−0.0018 , 0.0015]
IOW −0.0016 [−0.0038 , 0.0006]
MEDALL −0.0007 [−0.0020 , 0.0006]
MOBA1 −0.0007 [−0.0016 , 0.0003]
MOBA2 −0.0016 [−0.0026 , −0.0005]
MOBA3 0.0004 [−0.0022 , 0.0029]
NFCS −0.0018 [−0.0030 , −0.0005]
NEST −0.0007 [−0.0018 , 0.0004]
NHBCS −0.0023 [−0.0048 , 0.0002]
RICHS −0.0029 [−0.0044 , −0.0014]
VIVA 0.0000 [−0.0016 , 0.0015]
Fixed−effects meta−analysis −0.0010 [−0.0014 , −0.0007]
Random−effects meta−analysis −0.0011 [−0.0015 , −0.0006]
−0.0080 −0.0040 0.0000 0.0040
Change in offspring DNA methylation per1kg/m2 increase in maternal BMI

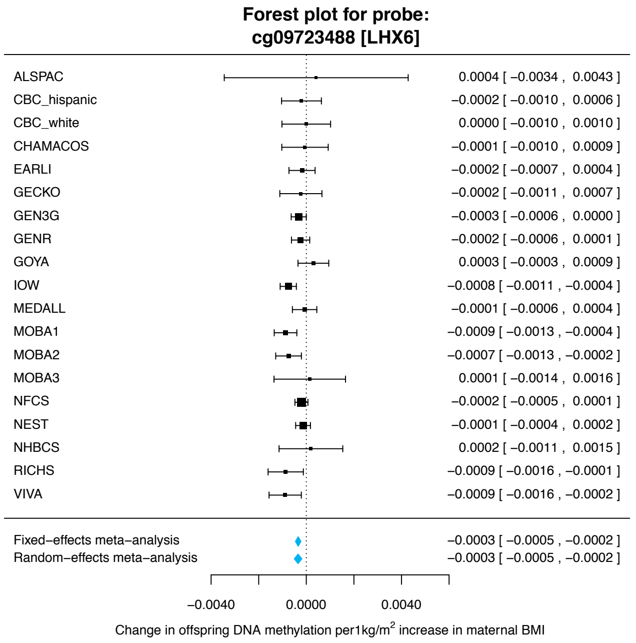
Forest plot for probe:
cg09723488 [LHX6]
ALSPAC 0.0004 [−0.0034 , 0.0043]
CBC_hispanic −0.0002 [−0.0010 , 0.0006]
CBC_white 0.0000 [−0.0010 , 0.0010]
CHAMACOS −0.0001 [−0.0010 , 0.0009]
EARLI −0.0002 [−0.0007 , 0.0004]
GECKO −0.0002 [−0.0011 , 0.0007]
GEN3G −0.0003 [−0.0006 , 0.0000]
GENR −0.0002 [−0.0006 , 0.0001]
GOYA 0.0003 [−0.0003 , 0.0009]
IOW −0.0008 [−0.0011 , −0.0004]
MEDALL −0.0001 [−0.0006 , 0.0004]
MOBA1 −0.0009 [−0.0013 , −0.0004]
MOBA2 −0.0007 [−0.0013 , −0.0002]
MOBA3 0.0001 [−0.0014 , 0.0016]
NFCS −0.0002 [−0.0005 , 0.0001]
NEST −0.0001 [−0.0004 , 0.0002]
NHBCS 0.0002 [−0.0011 , 0.0015]
RICHS −0.0009 [−0.0016 , −0.0001]
VIVA −0.0009 [−0.0016 , −0.0002]
Fixed−effects meta−analysis −0.0003 [−0.0005 , −0.0002]
Random−effects meta−analysis −0.0003 [−0.0005 , −0.0002]
−0.0040 0.0000 0.0040
Change in offspring DNA methylation per1kg/m2 increase in maternal BMI

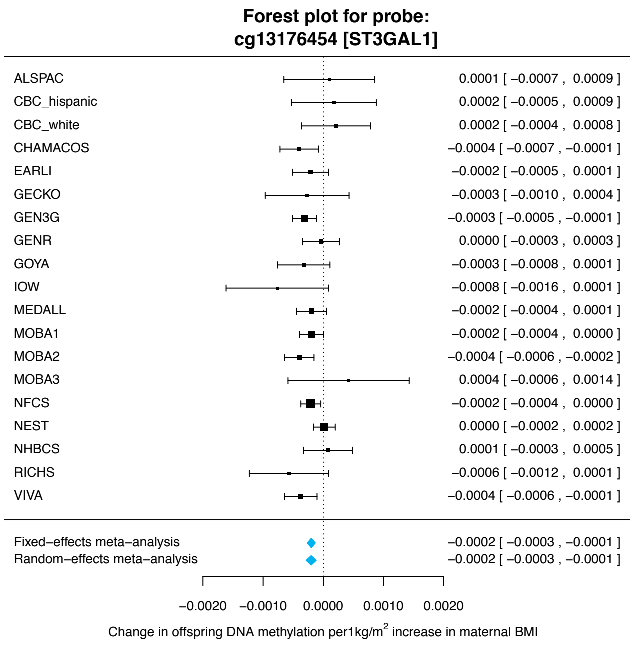
Forest plot for probe:
cg13176454 [ST3GAL1]
ALSPAC 0.0001 [−0.0007 , 0.0009]
CBC_hispanic 0.0002 [−0.0005 , 0.0009]
CBC_white 0.0002 [−0.0004 , 0.0008]
CHAMACOS −0.0004 [−0.0007 , −0.0001]
EARLI −0.0002 [−0.0005 , 0.0001]
GECKO −0.0003 [−0.0010 , 0.0004]
GEN3G −0.0003 [−0.0005 , −0.0001]
GENR 0.0000 [−0.0003 , 0.0003]
GOYA −0.0003 [−0.0008 , 0.0001]
IOW −0.0008 [−0.0016 , 0.0001]
MEDALL −0.0002 [−0.0004 , 0.0001]
MOBA1 −0.0002 [−0.0004 , 0.0000]
MOBA2 −0.0004 [−0.0006 , −0.0002]
MOBA3 0.0004 [−0.0006 , 0.0014]
NFCS −0.0002 [−0.0004 , 0.0000]
NEST 0.0000 [−0.0002 , 0.0002]
NHBCS 0.0001 [−0.0003 , 0.0005]
RICHS −0.0006 [−0.0012 , 0.0001]
VIVA −0.0004 [−0.0006 , −0.0001]
Fixed−effects meta−analysis −0.0002 [−0.0003 , −0.0001]
Random−effects meta−analysis −0.0002 [−0.0003 , −0.0001]
−0.0020 −0.0010 0.0000 0.0010 0.0020
Change in offspring DNA methylation per1kg/m2 increase in maternal BMI

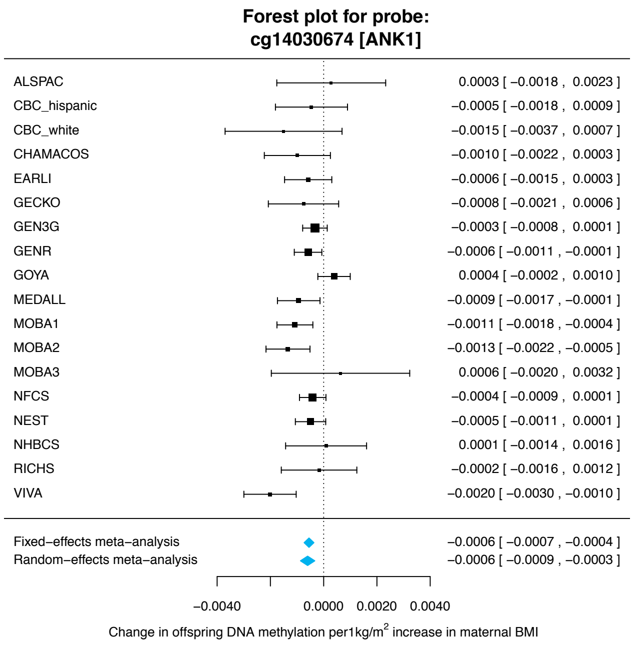
Forest plot for probe:
cg14030674 [ANK1]
ALSPAC 0.0003 [−0.0018 , 0.0023]
CBC_hispanic −0.0005 [−0.0018 , 0.0009]
CBC_white −0.0015 [−0.0037 , 0.0007]
CHAMACOS −0.0010 [−0.0022 , 0.0003]
EARLI −0.0006 [−0.0015 , 0.0003]
GECKO −0.0008 [−0.0021 , 0.0006]
GEN3G −0.0003 [−0.0008 , 0.0001]
GENR −0.0006 [−0.0011 , −0.0001]
GOYA 0.0004 [−0.0002 , 0.0010]
MEDALL −0.0009 [−0.0017 , −0.0001]
MOBA1 −0.0011 [−0.0018 , −0.0004]
MOBA2 −0.0013 [−0.0022 , −0.0005]
MOBA3 0.0006 [−0.0020 , 0.0032]
NFCS −0.0004 [−0.0009 , 0.0001]
NEST −0.0005 [−0.0011 , 0.0001]
NHBCS 0.0001 [−0.0014 , 0.0016]
RICHS −0.0002 [−0.0016 , 0.0012]
VIVA −0.0020 [−0.0030 , −0.0010]
Fixed−effects meta−analysis −0.0006 [−0.0007 , −0.0004]
Random−effects meta−analysis −0.0006 [−0.0009 , −0.0003]
−0.0040 0.0000 0.0020 0.0040
Change in offspring DNA methylation per1kg/m2 increase in maternal BMI

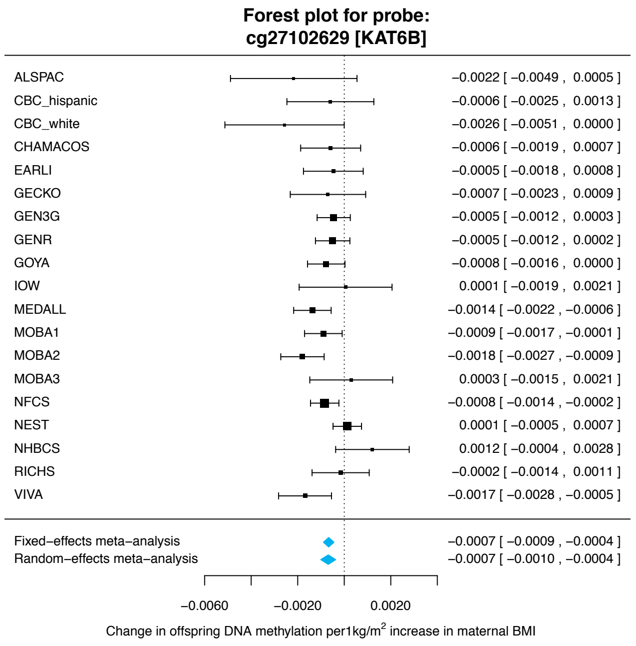
Forest plot for probe:
cg27102629 [KAT6B]
ALSPAC −0.0022 [−0.0049 , 0.0005]
CBC_hispanic −0.0006 [−0.0025 , 0.0013]
CBC_white −0.0026 [−0.0051 , 0.0000]
CHAMACOS −0.0006 [−0.0019 , 0.0007]
EARLI −0.0005 [−0.0018 , 0.0008]
GECKO −0.0007 [−0.0023 , 0.0009]
GEN3G −0.0005 [−0.0012 , 0.0003]
GENR −0.0005 [−0.0012 , 0.0002]
GOYA −0.0008 [−0.0016 , 0.0000]
IOW 0.0001 [−0.0019 , 0.0021]
MEDALL −0.0014 [−0.0022 , −0.0006]
MOBA1 −0.0009 [−0.0017 , −0.0001]
MOBA2 −0.0018 [−0.0027 , −0.0009]
MOBA3 0.0003 [−0.0015 , 0.0021]
NFCS −0.0008 [−0.0014 , −0.0002]
NEST 0.0001 [−0.0005 , 0.0007]
NHBCS 0.0012 [−0.0004 , 0.0028]
RICHS −0.0002 [−0.0014 , 0.0011]
VIVA −0.0017 [−0.0028 , −0.0005]
Fixed−effects meta−analysis −0.0007 [−0.0009 , −0.0004]
Random−effects meta−analysis −0.0007 [−0.0010 , −0.0004]
−0.0060 −0.0020 0.0020
Change in offspring DNA methylation per1kg/m2 increase in maternal BMI

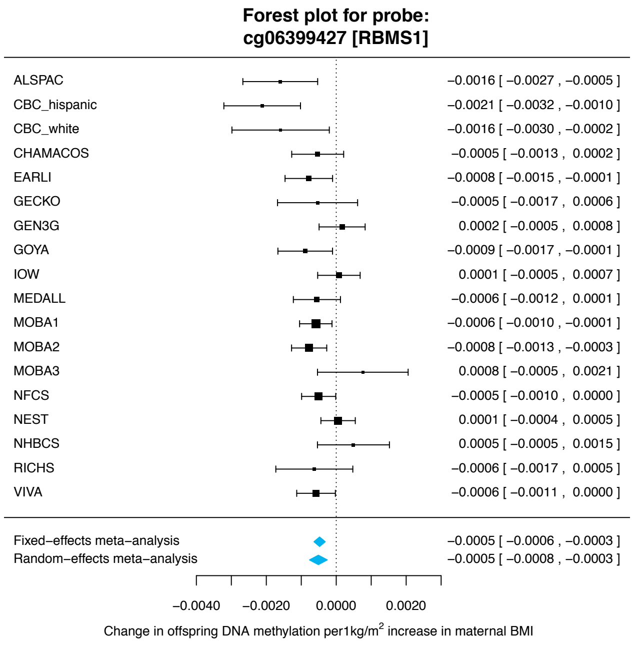
Forest plot for probe:
cg06399427 [RBMS1]
ALSPAC −0.0016 [−0.0027 , −0.0005]
CBC_hispanic −0.0021 [−0.0032 , −0.0010]
CBC_white −0.0016 [−0.0030 , −0.0002]
CHAMACOS −0.0005 [−0.0013 , 0.0002]
EARLI −0.0008 [−0.0015 , −0.0001]
GECKO −0.0005 [−0.0017 , 0.0006]
GEN3G 0.0002 [−0.0005 , 0.0008]
GOYA −0.0009 [−0.0017 , −0.0001]
IOW 0.0001 [−0.0005 , 0.0007]
MEDALL −0.0006 [−0.0012 , 0.0001]
MOBA1 −0.0006 [−0.0010 , −0.0001]
MOBA2 −0.0008 [−0.0013 , −0.0003]
MOBA3 0.0008 [−0.0005 , 0.0021]
NFCS −0.0005 [−0.0010 , 0.0000]
NEST 0.0001 [−0.0004 , 0.0005]
NHBCS 0.0005 [−0.0005 , 0.0015]
RICHS −0.0006 [−0.0017 , 0.0005]
VIVA −0.0006 [−0.0011 , 0.0000]
Fixed−effects meta−analysis −0.0005 [−0.0006 , −0.0003]
Random−effects meta−analysis −0.0005 [−0.0008 , −0.0003]
−0.0040 −0.0020 0.0000 0.0020
Change in offspring DNA methylation per1kg/m2 increase in maternal BMI

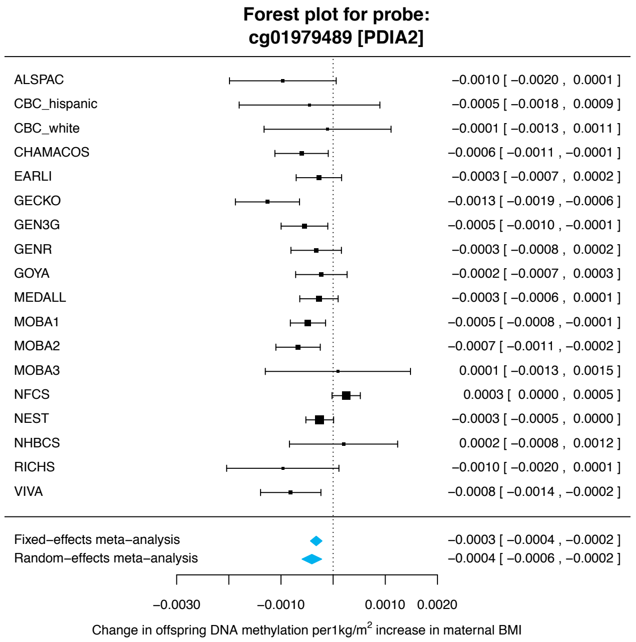

Forest plot for probe:
cg01979489 [PDIA2]
ALSPAC −0.0010 [−0.0020 , 0.0001]
CBC_hispanic −0.0005 [−0.0018 , 0.0009]
CBC_white −0.0001 [−0.0013 , 0.0011]
CHAMACOS −0.0006 [−0.0011 , −0.0001]
EARLI −0.0003 [−0.0007 , 0.0002]
GECKO −0.0013 [−0.0019 , −0.0006]
GEN3G −0.0005 [−0.0010 , −0.0001]
GENR −0.0003 [−0.0008 , 0.0002]
GOYA −0.0002 [−0.0007 , 0.0003]
MEDALL −0.0003 [−0.0006 , 0.0001]
MOBA1 −0.0005 [−0.0008 , −0.0001]
MOBA2 −0.0007 [−0.0011 , −0.0002]
MOBA3 0.0001 [−0.0013 , 0.0015]
NFCS 0.0003 [0.0000 , 0.0005]
NEST −0.0003 [−0.0005 , 0.0000]
NHBCS 0.0002 [−0.0008 , 0.0012]
RICHS −0.0010 [−0.0020 , 0.0001]
VIVA −0.0008 [−0.0014 , −0.0002]
Fixed−effects meta−analysis −0.0003 [−0.0004 , −0.0002]
Random−effects meta−analysis −0.0004 [−0.0006 , −0.0002]
−0.0030 −0.0010 0.0010 0.0020
Change in offspring DNA methylation per1kg/m2 increase in maternal BMI

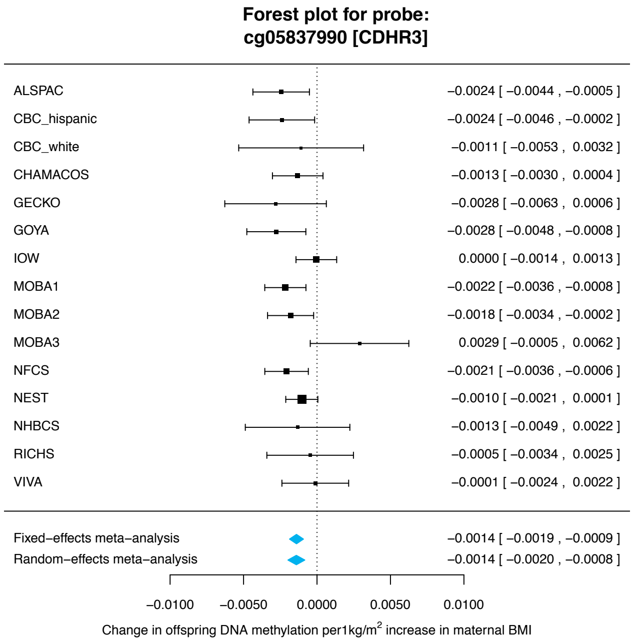

Forest plot for probe:
cg05837990 [CDHR3]
ALSPAC −0.0024 [−0.0044 , −0.0005]
CBC_hispanic −0.0024 [−0.0046 , −0.0002]
CBC_white −0.0011 [−0.0053 , 0.0032]
CHAMACOS −0.0013 [−0.0030 , 0.0004]
GECKO −0.0028 [−0.0063 , 0.0006]
GOYA −0.0028 [−0.0048 , −0.0008]
IOW 0.0000 [−0.0014 , 0.0013]
MOBA1 −0.0022 [−0.0036 , −0.0008]
MOBA2 −0.0018 [−0.0034 , −0.0002]
MOBA3 0.0029 [−0.0005 , 0.0062]
NFCS −0.0021 [−0.0036 , −0.0006]
NEST −0.0010 [−0.0021 , 0.0001]
NHBCS −0.0013 [−0.0049 , 0.0022]
RICHS −0.0005 [−0.0034 , 0.0025]
VIVA −0.0001 [−0.0024 , 0.0022]
Fixed−effects meta−analysis −0.0014 [−0.0019 , −0.0009]
Random−effects meta−analysis −0.0014 [−0.0020 , −0.0008]
−0.0100 −0.0050 0.0000 0.0050 0.0100
Change in offspring DNA methylation per1kg/m2 increase in maternal BMI

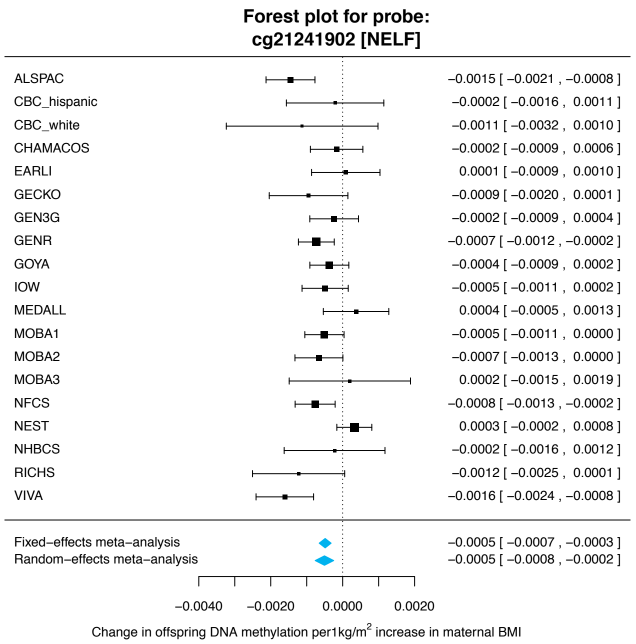

Forest plot for probe:
cg21241902 [NELF]
ALSPAC −0.0015 [−0.0021 , −0.0008]
CBC_hispanic −0.0002 [−0.0016 , 0.0011]
CBC_white −0.0011 [−0.0032 , 0.0010]
CHAMACOS −0.0002 [−0.0009 , 0.0006]
EARLI 0.0001 [−0.0009 , 0.0010]
GECKO −0.0009 [−0.0020 , 0.0001]
GEN3G −0.0002 [−0.0009 , 0.0004]
GENR −0.0007 [−0.0012 , −0.0002]
GOYA −0.0004 [−0.0009 , 0.0002]
IOW −0.0005 [−0.0011 , 0.0002]
MEDALL 0.0004 [−0.0005 , 0.0013]
MOBA1 −0.0005 [−0.0011 , 0.0000]
MOBA2 −0.0007 [−0.0013 , 0.0000]
MOBA3 0.0002 [−0.0015 , 0.0019]
NFCS −0.0008 [−0.0013 , −0.0002]
NEST 0.0003 [−0.0002 , 0.0008]
NHBCS −0.0002 [−0.0016 , 0.0012]
RICHS −0.0012 [−0.0025 , 0.0001]
VIVA −0.0016 [−0.0024 , −0.0008]
Fixed−effects meta−analysis −0.0005 [−0.0007 , −0.0003]
Random−effects meta−analysis −0.0005 [−0.0008 , −0.0002]
−0.0040 −0.0020 0.0000 0.0020
Change in offspring DNA methylation per1kg/m2 increase in maternal BMI

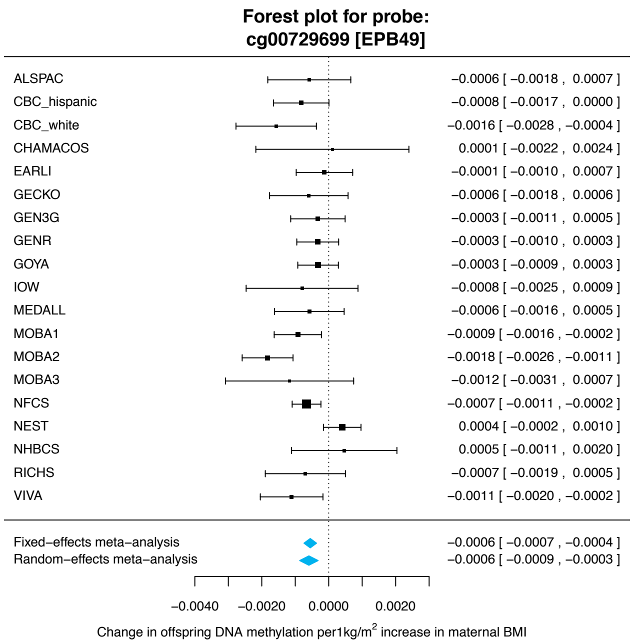

Forest plot for probe:
cg00729699 [EPB49]
ALSPAC −0.0006 [−0.0018 , 0.0007]
CBC_hispanic −0.0008 [−0.0017 , 0.0000]
CBC_white −0.0016 [−0.0028 , −0.0004]
CHAMACOS 0.0001 [−0.0022 , 0.0024]
EARLI −0.0001 [−0.0010 , 0.0007]
GECKO −0.0006 [−0.0018 , 0.0006]
GEN3G −0.0003 [−0.0011 , 0.0005]
GENR −0.0003 [−0.0010 , 0.0003]
GOYA −0.0003 [−0.0009 , 0.0003]
IOW −0.0008 [−0.0025 , 0.0009]
MEDALL −0.0006 [−0.0016 , 0.0005]
MOBA1 −0.0009 [−0.0016 , −0.0002]
MOBA2 −0.0018 [−0.0026 , −0.0011]
MOBA3 −0.0012 [−0.0031 , 0.0007]
NFCS −0.0007 [−0.0011 , −0.0002]
NEST 0.0004 [−0.0002 , 0.0010]
NHBCS 0.0005 [−0.0011 , 0.0020]
RICHS −0.0007 [−0.0019 , 0.0005]
VIVA −0.0011 [−0.0020 , −0.0002]
Fixed−effects meta−analysis −0.0006 [−0.0007 , −0.0004]
Random−effects meta−analysis −0.0006 [−0.0009 , −0.0003]
−0.0040 −0.0020 0.0000 0.0020
Change in offspring DNA methylation per1kg/m2 increase in maternal BMI

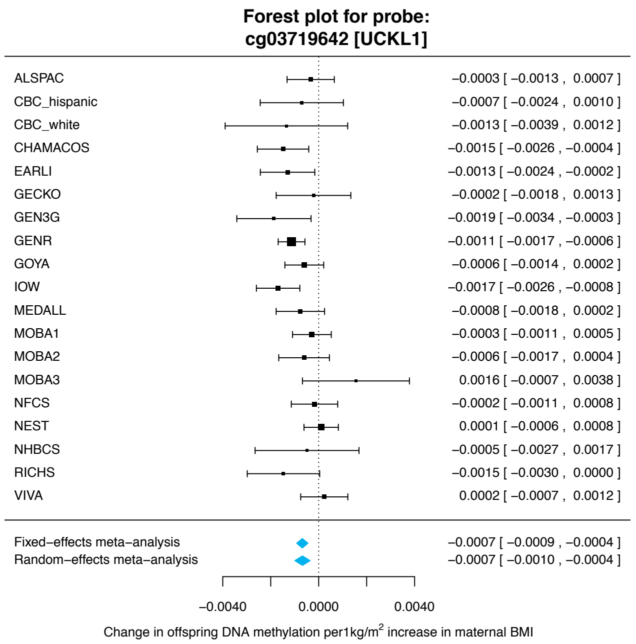

Forest plot for probe:
cg03719642 [UCKL1]
ALSPAC −0.0003 [−0.0013 , 0.0007]
CBC_hispanic −0.0007 [−0.0024 , 0.0010]
CBC_white −0.0013 [−0.0039 , 0.0012]
CHAMACOS −0.0015 [−0.0026 , −0.0004]
EARLI −0.0013 [−0.0024 , −0.0002]
GECKO −0.0002 [−0.0018 , 0.0013]
GEN3G −0.0019 [−0.0034 , −0.0003]
GENR −0.0011 [−0.0017 , −0.0006]
GOYA −0.0006 [−0.0014 , 0.0002]
IOW −0.0017 [−0.0026 , −0.0008]
MEDALL −0.0008 [−0.0018 , 0.0002]
MOBA1 −0.0003 [−0.0011 , 0.0005]
MOBA2 −0.0006 [−0.0017 , 0.0004]
MOBA3 0.0016 [−0.0007 , 0.0038]
NFCS −0.0002 [−0.0011 , 0.0008]
NEST 0.0001 [−0.0006 , 0.0008]
NHBCS −0.0005 [−0.0027 , 0.0017]
RICHS −0.0015 [−0.0030 , 0.0000]
VIVA 0.0002 [−0.0007 , 0.0012]
Fixed−effects meta−analysis −0.0007 [−0.0009 , −0.0004]
Random−effects meta−analysis −0.0007 [−0.0010 , −0.0004]
−0.0040 0.0000 0.0040
Change in offspring DNA methylation per1kg/m2 increase in maternal BMI

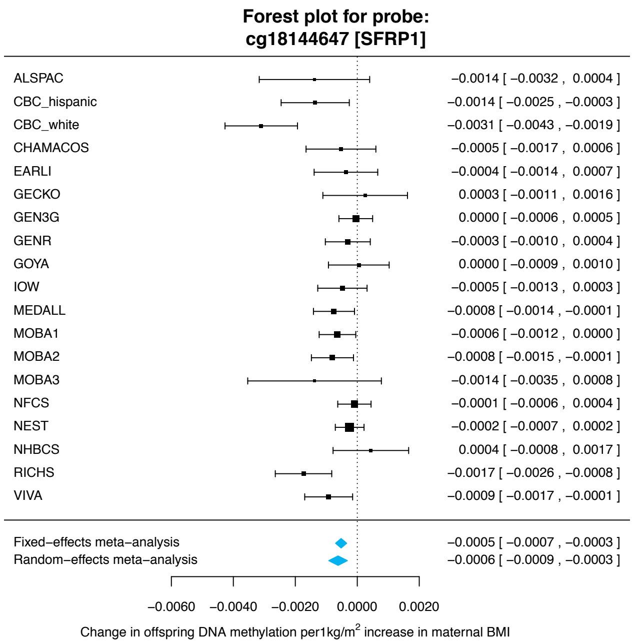

Forest plot for probe:
cg18144647 [SFRP1]
ALSPAC −0.0014 [−0.0032 , 0.0004]
CBC_hispanic −0.0014 [−0.0025 , −0.0003]
CBC_white −0.0031 [−0.0043 , −0.0019]
CHAMACOS −0.0005 [−0.0017 , 0.0006]
EARLI −0.0004 [−0.0014 , 0.0007]
GECKO 0.0003 [−0.0011 , 0.0016]
GEN3G 0.0000 [−0.0006 , 0.0005]
GENR −0.0003 [−0.0010 , 0.0004]
GOYA 0.0000 [−0.0009 , 0.0010]
IOW −0.0005 [−0.0013 , 0.0003]
MEDALL −0.0008 [−0.0014 , −0.0001]
MOBA1 −0.0006 [−0.0012 , 0.0000]
MOBA2 −0.0008 [−0.0015 , −0.0001]
MOBA3 −0.0014 [−0.0035 , 0.0008]
NFCS −0.0001 [−0.0006 , 0.0004]
NEST −0.0002 [−0.0007 , 0.0002]
NHBCS 0.0004 [−0.0008 , 0.0017]
RICHS −0.0017 [−0.0026 , −0.0008]
VIVA −0.0009 [−0.0017 , −0.0001]
Fixed−effects meta−analysis −0.0005 [−0.0007 , −0.0003]
Random−effects meta−analysis −0.0006 [−0.0009 , −0.0003]
−0.0060 −0.0040 −0.0020 0.0000 0.0020
Change in offspring DNA methylation per1kg/m2 increase in maternal BMI

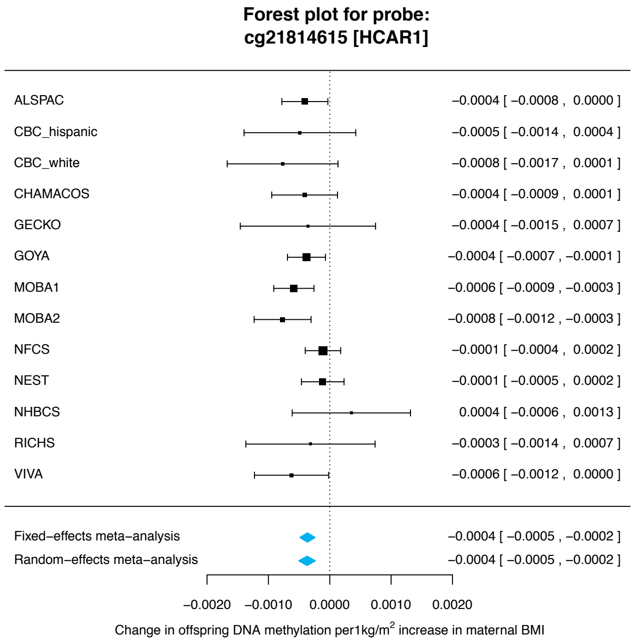
Forest plot for probe:
cg21814615 [HCAR1]
ALSPAC −0.0004 [−0.0008 , 0.0000]
CBC_hispanic −0.0005 [−0.0014 , 0.0004]
CBC_white −0.0008 [−0.0017 , 0.0001]
CHAMACOS −0.0004 [−0.0009 , 0.0001]
GECKO −0.0004 [−0.0015 , 0.0007]
GOYA −0.0004 [−0.0007 , −0.0001]
MOBA1 −0.0006 [−0.0009 , −0.0003]
MOBA2 −0.0008 [−0.0012 , −0.0003]
NFCS −0.0001 [−0.0004 , 0.0002]
NEST −0.0001 [−0.0005 , 0.0002]
NHBCS 0.0004 [−0.0006 , 0.0013]
RICHS −0.0003 [−0.0014 , 0.0007]
VIVA −0.0006 [−0.0012 , 0.0000]
Fixed−effects meta−analysis −0.0004 [−0.0005 , −0.0002]
Random−effects meta−analysis −0.0004 [−0.0005 , −0.0002]
−0.0020 −0.0010 0.0000 0.0010 0.0020
Change in offspring DNA methylation per1kg/m2 increase in maternal BMI

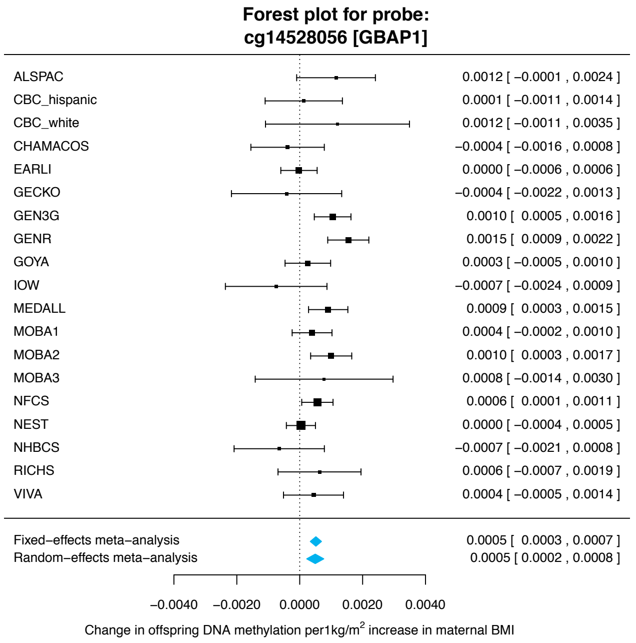
Forest plot for probe:
cg14528056 [GBAP1]
ALSPAC 0.0012 [−0.0001 , 0.0024]
CBC_hispanic 0.0001 [−0.0011 , 0.0014]
CBC_white 0.0012 [−0.0011 , 0.0035]
CHAMACOS −0.0004 [−0.0016 , 0.0008]
EARLI 0.0000 [−0.0006 , 0.0006]
GECKO −0.0004 [−0.0022 , 0.0013]
GEN3G 0.0010 [0.0005 , 0.0016]
GENR 0.0015 [0.0009 , 0.0022]
GOYA 0.0003 [−0.0005 , 0.0010]
IOW −0.0007 [−0.0024 , 0.0009]
MEDALL 0.0009 [0.0003 , 0.0015]
MOBA1 0.0004 [−0.0002 , 0.0010]
MOBA2 0.0010 [0.0003 , 0.0017]
MOBA3 0.0008 [−0.0014 , 0.0030]
NFCS 0.0006 [0.0001 , 0.0011]
NEST 0.0000 [−0.0004 , 0.0005]
NHBCS −0.0007 [−0.0021 , 0.0008]
RICHS 0.0006 [−0.0007 , 0.0019]
VIVA 0.0004 [−0.0005 , 0.0014]
Fixed−effects meta−analysis 0.0005 [0.0003 , 0.0007]
Random−effects meta−analysis 0.0005 [0.0002 , 0.0008]
−0.0040 −0.0020 0.0000 0.0020 0.0040
Change in offspring DNA methylation per1kg/m2 increase in maternal BMI

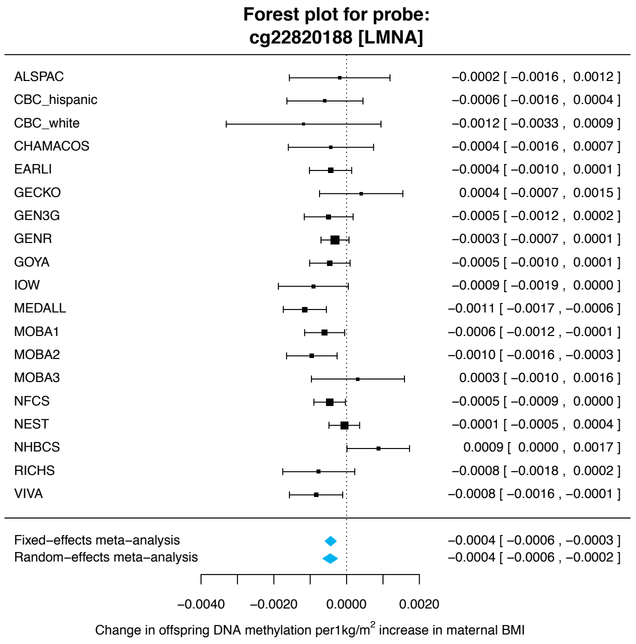
Forest plot for probe:
cg22820188 [LMNA]
ALSPAC −0.0002 [−0.0016 , 0.0012]
CBC_hispanic −0.0006 [−0.0016 , 0.0004]
CBC_white −0.0012 [−0.0033 , 0.0009]
CHAMACOS −0.0004 [−0.0016 , 0.0007]
EARLI −0.0004 [−0.0010 , 0.0001]
GECKO 0.0004 [−0.0007 , 0.0015]
GEN3G −0.0005 [−0.0012 , 0.0002]
GENR −0.0003 [−0.0007 , 0.0001]
GOYA −0.0005 [−0.0010 , 0.0001]
IOW −0.0009 [−0.0019 , 0.0000]
MEDALL −0.0011 [−0.0017 , −0.0006]
MOBA1 −0.0006 [−0.0012 , −0.0001]
MOBA2 −0.0010 [−0.0016 , −0.0003]
MOBA3 0.0003 [−0.0010 , 0.0016]
NFCS −0.0005 [−0.0009 , 0.0000]
NEST −0.0001 [−0.0005 , 0.0004]
NHBCS 0.0009 [0.0000 , 0.0017]
RICHS −0.0008 [−0.0018 , 0.0002]
VIVA −0.0008 [−0.0016 , −0.0001]
Fixed−effects meta−analysis −0.0004 [−0.0006 , −0.0003]
Random−effects meta−analysis −0.0004 [−0.0006 , −0.0002]
−0.0040 −0.0020 0.0000 0.0020
Change in offspring DNA methylation per1kg/m2 increase in maternal BMI

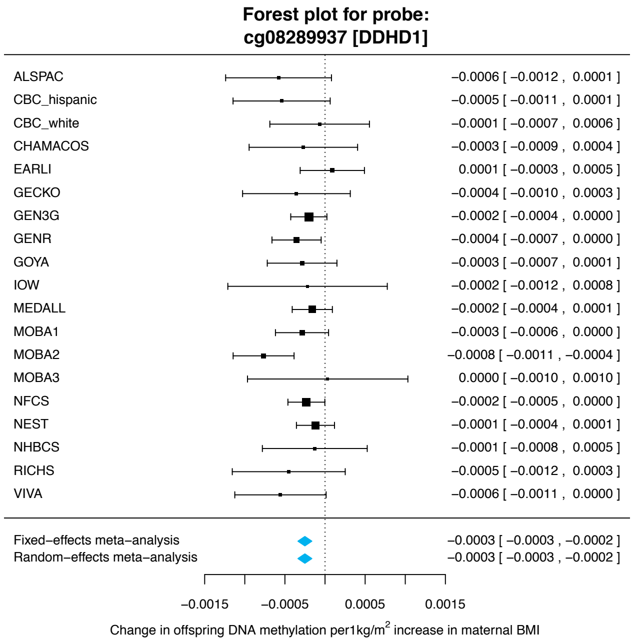
Forest plot for probe:
cg08289937 [DDHD1]
ALSPAC −0.0006 [−0.0012 , 0.0001]
CBC_hispanic −0.0005 [−0.0011 , 0.0001]
CBC_white −0.0001 [−0.0007 , 0.0006]
CHAMACOS −0.0003 [−0.0009 , 0.0004]
EARLI 0.0001 [−0.0003 , 0.0005]
GECKO −0.0004 [−0.0010 , 0.0003]
GEN3G −0.0002 [−0.0004 , 0.0000]
GENR −0.0004 [−0.0007 , 0.0000]
GOYA −0.0003 [−0.0007 , 0.0001]
IOW −0.0002 [−0.0012 , 0.0008]
MEDALL −0.0002 [−0.0004 , 0.0001]
MOBA1 −0.0003 [−0.0006 , 0.0000]
MOBA2 −0.0008 [−0.0011 , −0.0004]
MOBA3 0.0000 [−0.0010 , 0.0010]
NFCS −0.0002 [−0.0005 , 0.0000]
NEST −0.0001 [−0.0004 , 0.0001]
NHBCS −0.0001 [−0.0008 , 0.0005]
RICHS −0.0005 [−0.0012 , 0.0003]
VIVA −0.0006 [−0.0011 , 0.0000]
Fixed−effects meta−analysis −0.0003 [−0.0003 , −0.0002]
Random−effects meta−analysis −0.0003 [−0.0003 , −0.0002]
−0.0015 −0.0005 0.0005 0.0015
Change in offspring DNA methylation per1kg/m2 increase in maternal BMI

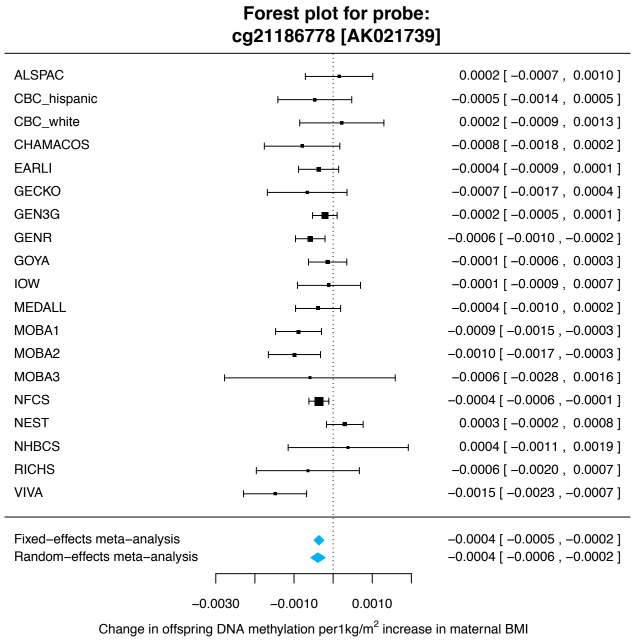
Forest plot for probe:
cg21186778 [AK021739]
ALSPAC 0.0002 [−0.0007 , 0.0010]
CBC_hispanic −0.0005 [−0.0014 , 0.0005]
CBC_white 0.0002 [−0.0009 , 0.0013]
CHAMACOS −0.0008 [−0.0018 , 0.0002]
EARLI −0.0004 [−0.0009 , 0.0001]
GECKO −0.0007 [−0.0017 , 0.0004]
GEN3G −0.0002 [−0.0005 , 0.0001]
GENR −0.0006 [−0.0010 , −0.0002]
GOYA −0.0001 [−0.0006 , 0.0003]
IOW −0.0001 [−0.0009 , 0.0007]
MEDALL −0.0004 [−0.0010 , 0.0002]
MOBA1 −0.0009 [−0.0015 , −0.0003]
MOBA2 −0.0010 [−0.0017 , −0.0003]
MOBA3 −0.0006 [−0.0028 , 0.0016]
NFCS −0.0004 [−0.0006 , −0.0001]
NEST 0.0003 [−0.0002 , 0.0008]
NHBCS 0.0004 [−0.0011 , 0.0019]
RICHS −0.0006 [−0.0020 , 0.0007]
VIVA −0.0015 [−0.0023 , −0.0007]
Fixed−effects meta−analysis −0.0004 [−0.0005 , −0.0002]
Random−effects meta−analysis −0.0004 [−0.0006 , −0.0002]
−0.0030 −0.0010 0.0010
Change in offspring DNA methylation per1kg/m2 increase in maternal BMI

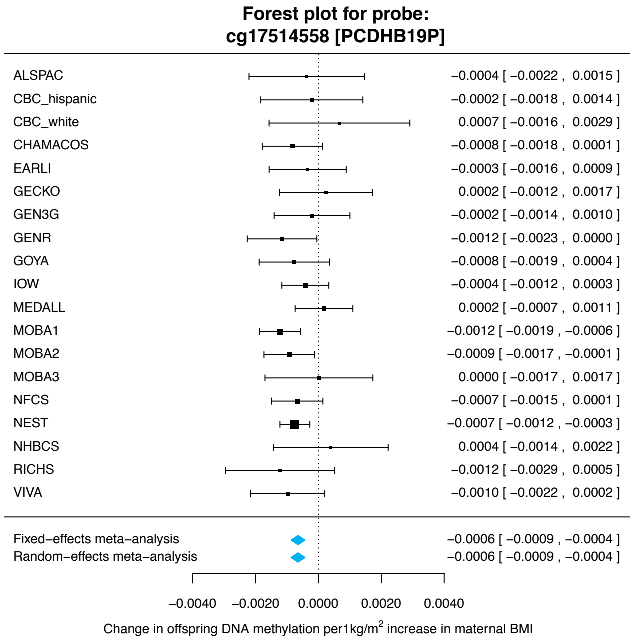
Forest plot for probe:
cg17514558 [PCDHB19P]
ALSPAC −0.0004 [−0.0022 , 0.0015]
CBC_hispanic −0.0002 [−0.0018 , 0.0014]
CBC_white 0.0007 [−0.0016 , 0.0029]
CHAMACOS −0.0008 [−0.0018 , 0.0001]
EARLI −0.0003 [−0.0016 , 0.0009]
GECKO 0.0002 [−0.0012 , 0.0017]
GEN3G −0.0002 [−0.0014 , 0.0010]
GENR −0.0012 [−0.0023 , 0.0000]
GOYA −0.0008 [−0.0019 , 0.0004]
IOW −0.0004 [−0.0012 , 0.0003]
MEDALL 0.0002 [−0.0007 , 0.0011]
MOBA1 −0.0012 [−0.0019 , −0.0006]
MOBA2 −0.0009 [−0.0017 , −0.0001]
MOBA3 0.0000 [−0.0017 , 0.0017]
NFCS −0.0007 [−0.0015 , 0.0001]
NEST −0.0007 [−0.0012 , −0.0003]
NHBCS 0.0004 [−0.0014 , 0.0022]
RICHS −0.0012 [−0.0029 , 0.0005]
VIVA −0.0010 [−0.0022 , 0.0002]
Fixed−effects meta−analysis −0.0006 [−0.0009 , −0.0004]
Random−effects meta−analysis −0.0006 [−0.0009 , −0.0004]
−0.0040 −0.0020 0.0000 0.0020 0.0040
Change in offspring DNA methylation per1kg/m2 increase in maternal BMI

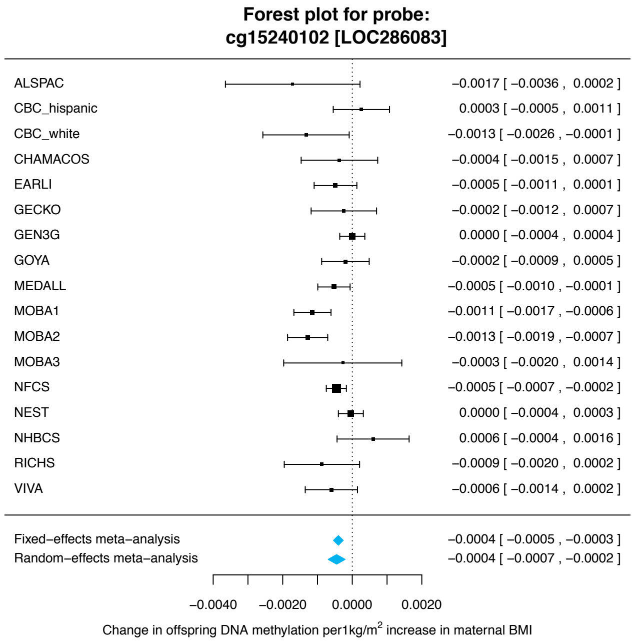
Forest plot for probe:
cg15240102 [LOC286083]
ALSPAC −0.0017 [−0.0036 , 0.0002]
CBC_hispanic 0.0003 [−0.0005 , 0.0011]
CBC_white −0.0013 [−0.0026 , −0.0001]
CHAMACOS −0.0004 [−0.0015 , 0.0007]
EARLI −0.0005 [−0.0011 , 0.0001]
GECKO −0.0002 [−0.0012 , 0.0007]
GEN3G 0.0000 [−0.0004 , 0.0004]
GOYA −0.0002 [−0.0009 , 0.0005]
MEDALL −0.0005 [−0.0010 , −0.0001]
MOBA1 −0.0011 [−0.0017 , −0.0006]
MOBA2 −0.0013 [−0.0019 , −0.0007]
MOBA3 −0.0003 [−0.0020 , 0.0014]
NFCS −0.0005 [−0.0007 , −0.0002]
NEST 0.0000 [−0.0004 , 0.0003]
NHBCS 0.0006 [−0.0004 , 0.0016]
RICHS −0.0009 [−0.0020 , 0.0002]
VIVA −0.0006 [−0.0014 , 0.0002]
Fixed−effects meta−analysis −0.0004 [−0.0005 , −0.0003]
Random−effects meta−analysis −0.0004 [−0.0007 , −0.0002]
−0.0040 −0.0020 0.0000 0.0020
Change in offspring DNA methylation per1kg/m2 increase in maternal BMI

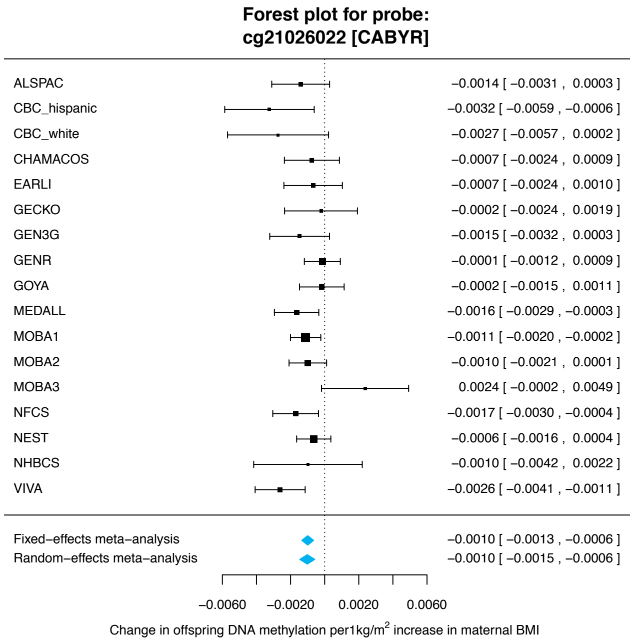
Forest plot for probe:
cg21026022 [CABYR]
ALSPAC −0.0014 [−0.0031 , 0.0003]
CBC_hispanic −0.0032 [−0.0059 , −0.0006]
CBC_white −0.0027 [−0.0057 , 0.0002]
CHAMACOS −0.0007 [−0.0024 , 0.0009]
EARLI −0.0007 [−0.0024 , 0.0010]
GECKO −0.0002 [−0.0024 , 0.0019]
GEN3G −0.0015 [−0.0032 , 0.0003]
GENR −0.0001 [−0.0012 , 0.0009]
GOYA −0.0002 [−0.0015 , 0.0011]
MEDALL −0.0016 [−0.0029 , −0.0003]
MOBA1 −0.0011 [−0.0020 , −0.0002]
MOBA2 −0.0010 [−0.0021 , 0.0001]
MOBA3 0.0024 [−0.0002 , 0.0049]
NFCS −0.0017 [−0.0030 , −0.0004]
NEST −0.0006 [−0.0016 , 0.0004]
NHBCS −0.0010 [−0.0042 , 0.0022]
VIVA −0.0026 [−0.0041 , −0.0011]
Fixed−effects meta−analysis −0.0010 [−0.0013 , −0.0006]
Random−effects meta−analysis −0.0010 [−0.0015 , −0.0006]
−0.0060 −0.0020 0.0020 0.0060
Change in offspring DNA methylation per1kg/m2 increase in maternal BMI

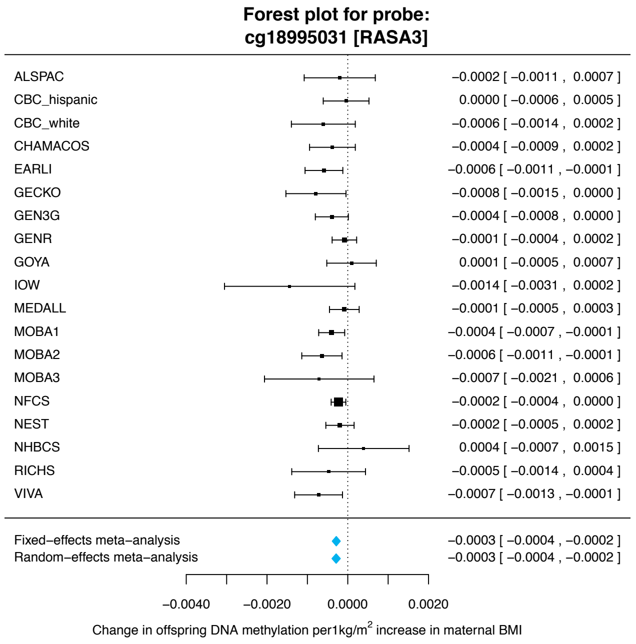
Forest plot for probe:
cg18995031 [RASA3]
ALSPAC −0.0002 [−0.0011 , 0.0007]
CBC_hispanic 0.0000 [−0.0006 , 0.0005]
CBC_white −0.0006 [−0.0014 , 0.0002]
CHAMACOS −0.0004 [−0.0009 , 0.0002]
EARLI −0.0006 [−0.0011 , −0.0001]
GECKO −0.0008 [−0.0015 , 0.0000]
GEN3G −0.0004 [−0.0008 , 0.0000]
GENR −0.0001 [−0.0004 , 0.0002]
GOYA 0.0001 [−0.0005 , 0.0007]
IOW −0.0014 [−0.0031 , 0.0002]
MEDALL −0.0001 [−0.0005 , 0.0003]
MOBA1 −0.0004 [−0.0007 , −0.0001]
MOBA2 −0.0006 [−0.0011 , −0.0001]
MOBA3 −0.0007 [−0.0021 , 0.0006]
NFCS −0.0002 [−0.0004 , 0.0000]
NEST −0.0002 [−0.0005 , 0.0002]
NHBCS 0.0004 [−0.0007 , 0.0015]
RICHS −0.0005 [−0.0014 , 0.0004]
VIVA −0.0007 [−0.0013 , −0.0001]
Fixed−effects meta−analysis −0.0003 [−0.0004 , −0.0002]
Random−effects meta−analysis −0.0003 [−0.0004 , −0.0002]
−0.0040 −0.0020 0.0000 0.0020
Change in offspring DNA methylation per1kg/m2 increase in maternal BMI

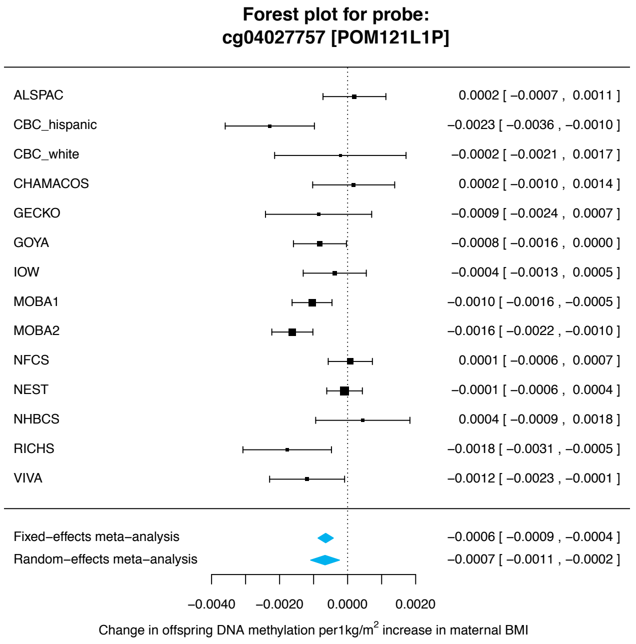
Forest plot for probe:
cg04027757 [POM121L1P]
ALSPAC 0.0002 [−0.0007 , 0.0011]
CBC_hispanic −0.0023 [−0.0036 , −0.0010]
CBC_white −0.0002 [−0.0021 , 0.0017]
CHAMACOS 0.0002 [−0.0010 , 0.0014]
GECKO −0.0009 [−0.0024 , 0.0007]
GOYA −0.0008 [−0.0016 , 0.0000]
IOW −0.0004 [−0.0013 , 0.0005]
MOBA1 −0.0010 [−0.0016 , −0.0005]
MOBA2 −0.0016 [−0.0022 , −0.0010]
NFCS 0.0001 [−0.0006 , 0.0007]
NEST −0.0001 [−0.0006 , 0.0004]
NHBCS 0.0004 [−0.0009 , 0.0018]
RICHS −0.0018 [−0.0031 , −0.0005]
VIVA −0.0012 [−0.0023 , −0.0001]
Fixed−effects meta−analysis −0.0006 [−0.0009 , −0.0004]
Random−effects meta−analysis −0.0007 [−0.0011 , −0.0002]
−0.0040 −0.0020 0.0000 0.0020
Change in offspring DNA methylation per1kg/m2 increase in maternal BMI

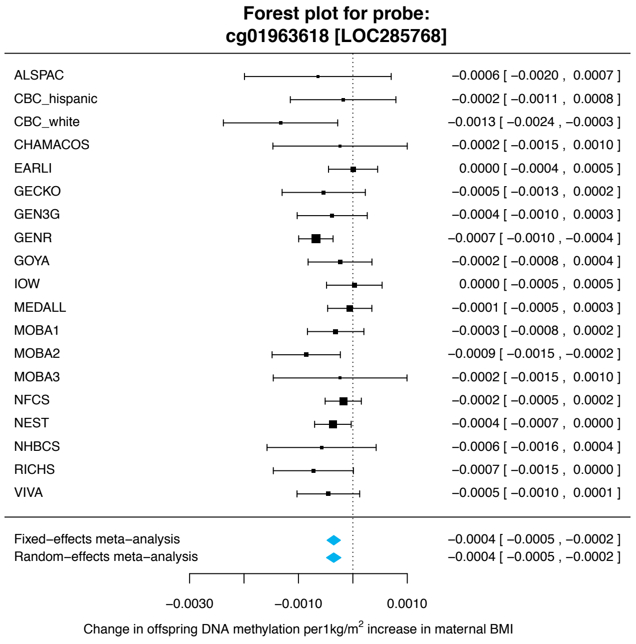
Forest plot for probe:
cg01963618 [LOC285768]
ALSPAC −0.0006 [−0.0020 , 0.0007]
CBC_hispanic −0.0002 [−0.0011 , 0.0008]
CBC_white −0.0013 [−0.0024 , −0.0003]
CHAMACOS −0.0002 [−0.0015 , 0.0010]
EARLI 0.0000 [−0.0004 , 0.0005]
GECKO −0.0005 [−0.0013 , 0.0002]
GEN3G −0.0004 [−0.0010 , 0.0003]
GENR −0.0007 [−0.0010 , −0.0004]
GOYA −0.0002 [−0.0008 , 0.0004]
IOW 0.0000 [−0.0005 , 0.0005]
MEDALL −0.0001 [−0.0005 , 0.0003]
MOBA1 −0.0003 [−0.0008 , 0.0002]
MOBA2 −0.0009 [−0.0015 , −0.0002]
MOBA3 −0.0002 [−0.0015 , 0.0010]
NFCS −0.0002 [−0.0005 , 0.0002]
NEST −0.0004 [−0.0007 , 0.0000]
NHBCS −0.0006 [−0.0016 , 0.0004]
RICHS −0.0007 [−0.0015 , 0.0000]
VIVA −0.0005 [−0.0010 , 0.0001]
Fixed−effects meta−analysis −0.0004 [−0.0005 , −0.0002]
Random−effects meta−analysis −0.0004 [−0.0005 , −0.0002]
−0.0030 −0.0010 0.0010
Change in offspring DNA methylation per1kg/m2 increase in maternal BMI

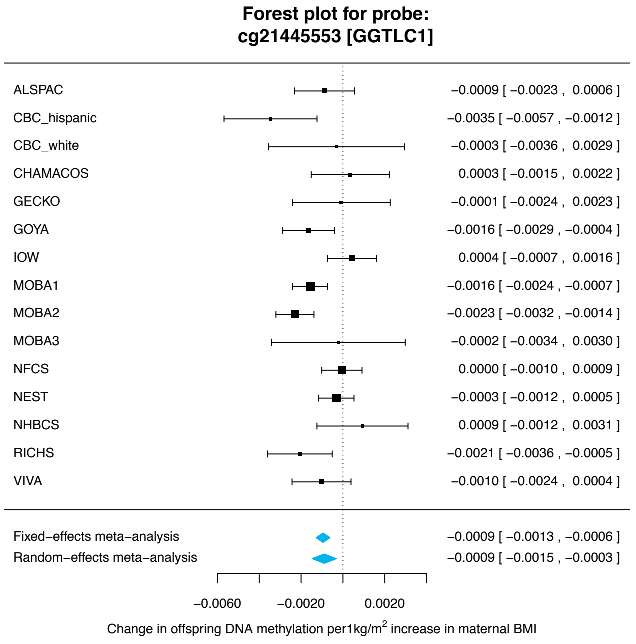
Forest plot for probe:
cg21445553 [GGTLC1]
ALSPAC −0.0009 [−0.0023 , 0.0006]
CBC_hispanic −0.0035 [−0.0057 , −0.0012]
CBC_white −0.0003 [−0.0036 , 0.0029]
CHAMACOS 0.0003 [−0.0015 , 0.0022]
GECKO −0.0001 [−0.0024 , 0.0023]
GOYA −0.0016 [−0.0029 , −0.0004]
IOW 0.0004 [−0.0007 , 0.0016]
MOBA1 −0.0016 [−0.0024 , −0.0007]
MOBA2 −0.0023 [−0.0032 , −0.0014]
MOBA3 −0.0002 [−0.0034 , 0.0030]
NFCS 0.0000 [−0.0010 , 0.0009]
NEST −0.0003 [−0.0012 , 0.0005]
NHBCS 0.0009 [−0.0012 , 0.0031]
RICHS −0.0021 [−0.0036 , −0.0005]
VIVA −0.0010 [−0.0024 , 0.0004]
Fixed−effects meta−analysis −0.0009 [−0.0013 , −0.0006]
Random−effects meta−analysis −0.0009 [−0.0015 , −0.0003]
−0.0060 −0.0020 0.0020
Change in offspring DNA methylation per1kg/m2 increase in maternal BMI

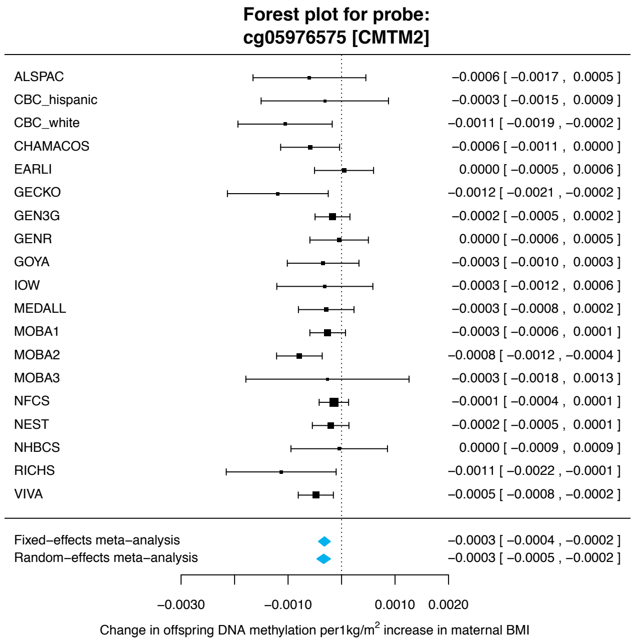
Forest plot for probe:
cg05976575 [CMTM2]
ALSPAC −0.0006 [−0.0017 , 0.0005]
CBC_hispanic −0.0003 [−0.0015 , 0.0009]
CBC_white −0.0011 [−0.0019 , −0.0002]
CHAMACOS −0.0006 [−0.0011 , 0.0000]
EARLI 0.0000 [−0.0005 , 0.0006]
GECKO −0.0012 [−0.0021 , −0.0002]
GEN3G −0.0002 [−0.0005 , 0.0002]
GENR 0.0000 [−0.0006 , 0.0005]
GOYA −0.0003 [−0.0010 , 0.0003]
IOW −0.0003 [−0.0012 , 0.0006]
MEDALL −0.0003 [−0.0008 , 0.0002]
MOBA1 −0.0003 [−0.0006 , 0.0001]
MOBA2 −0.0008 [−0.0012 , −0.0004]
MOBA3 −0.0003 [−0.0018 , 0.0013]
NFCS −0.0001 [−0.0004 , 0.0001]
NEST −0.0002 [−0.0005 , 0.0001]
NHBCS 0.0000 [−0.0009 , 0.0009]
RICHS −0.0011 [−0.0022 , −0.0001]
VIVA −0.0005 [−0.0008 , −0.0002]
Fixed−effects meta−analysis −0.0003 [−0.0004 , −0.0002]
Random−effects meta−analysis −0.0003 [−0.0005 , −0.0002]
−0.0030
−0.0010
0.0010
0.0020
Change in offspring DNA methylation per1kg/m2 increase in maternal BMI

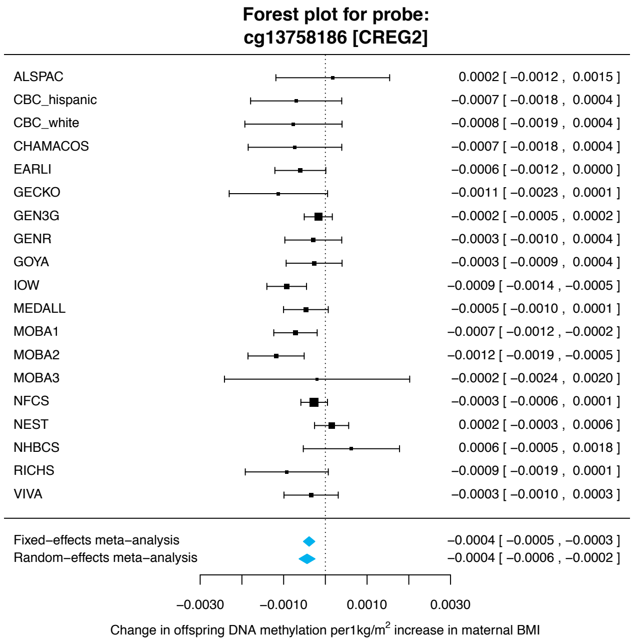
Forest plot for probe:
cg13758186 [CREG2]
ALSPAC 0.0002 [−0.0012 , 0.0015]
CBC_hispanic −0.0007 [−0.0018 , 0.0004]
CBC_white −0.0008 [−0.0019 , 0.0004]
CHAMACOS −0.0007 [−0.0018 , 0.0004]
EARLI −0.0006 [−0.0012 , 0.0000]
GECKO −0.0011 [−0.0023 , 0.0001]
GEN3G −0.0002 [−0.0005 , 0.0002]
GENR −0.0003 [−0.0010 , 0.0004]
GOYA −0.0003 [−0.0009 , 0.0004]
IOW −0.0009 [−0.0014 , −0.0005]
MEDALL −0.0005 [−0.0010 , 0.0001]
MOBA1 −0.0007 [−0.0012 , −0.0002]
MOBA2 −0.0012 [−0.0019 , −0.0005]
MOBA3 −0.0002 [−0.0024 , 0.0020]
NFCS −0.0003 [−0.0006 , 0.0001]
NEST 0.0002 [−0.0003 , 0.0006]
NHBCS 0.0006 [−0.0005 , 0.0018]
RICHS −0.0009 [−0.0019 , 0.0001]
VIVA −0.0003 [−0.0010 , 0.0003]
Fixed−effects meta−analysis −0.0004 [−0.0005 , −0.0003]
Random−effects meta−analysis −0.0004 [−0.0006 , −0.0002]
−0.0030
−0.0010
0.0010
0.0030
Change in offspring DNA methylation per1kg/m2 increase in maternal BMI

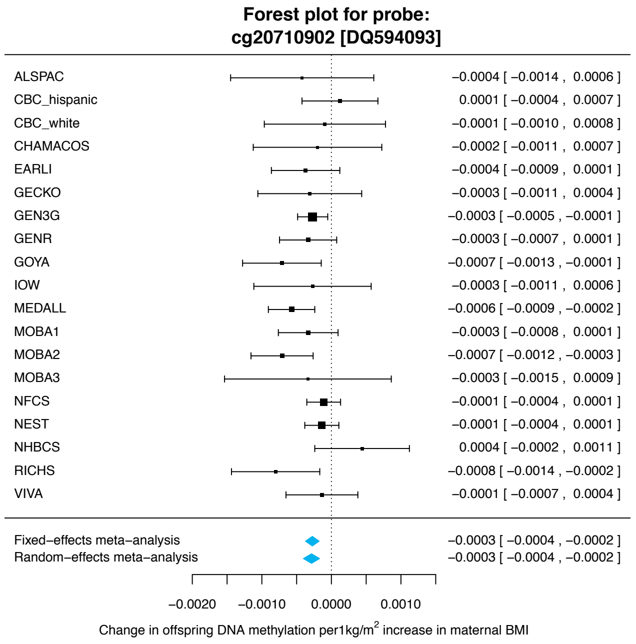
Forest plot for probe:
cg20710902 [DQ594093]
ALSPAC −0.0004 [−0.0014 , 0.0006]
CBC_hispanic 0.0001 [−0.0004 , 0.0007]
CBC_white −0.0001 [−0.0010 , 0.0008]
CHAMACOS −0.0002 [−0.0011 , 0.0007]
EARLI −0.0004 [−0.0009 , 0.0001]
GECKO −0.0003 [−0.0011 , 0.0004]
GEN3G −0.0003 [−0.0005 , −0.0001]
GENR −0.0003 [−0.0007 , 0.0001]
GOYA −0.0007 [−0.0013 , −0.0001]
IOW −0.0003 [−0.0011 , 0.0006]
MEDALL −0.0006 [−0.0009 , −0.0002]
MOBA1 −0.0003 [−0.0008 , 0.0001]
MOBA2 −0.0007 [−0.0012 , −0.0003]
MOBA3 −0.0003 [−0.0015 , 0.0009]
NFCS −0.0001 [−0.0004 , 0.0001]
NEST −0.0001 [−0.0004 , 0.0001]
NHBCS 0.0004 [−0.0002 , 0.0011]
RICHS −0.0008 [−0.0014 , −0.0002]
VIVA −0.0001 [−0.0007 , 0.0004]
Fixed−effects meta−analysis −0.0003 [−0.0004 , −0.0002]
Random−effects meta−analysis −0.0003 [−0.0004 , −0.0002]
−0.0020
−0.0010
0.0000
0.0010
Change in offspring DNA methylation per1kg/m2 increase in maternal BMI

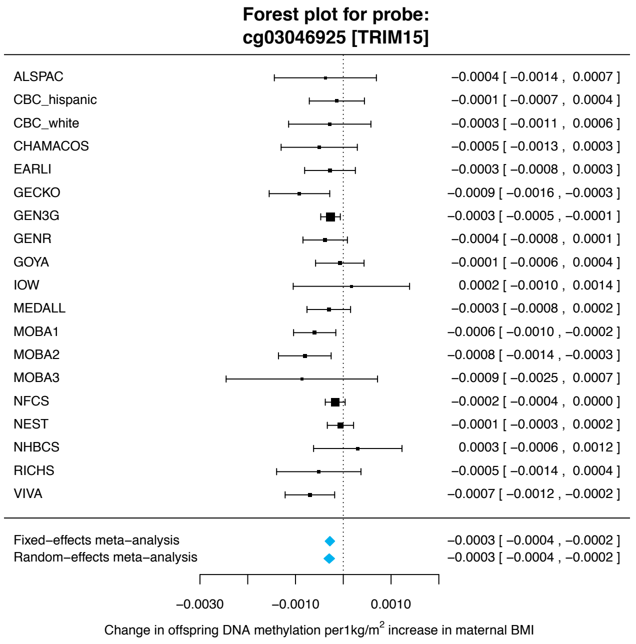
Forest plot for probe:
cg03046925 [TRIM15]
ALSPAC −0.0004 [−0.0014 , 0.0007]
CBC_hispanic −0.0001 [−0.0007 , 0.0004]
CBC_white −0.0003 [−0.0011 , 0.0006]
CHAMACOS −0.0005 [−0.0013 , 0.0003]
EARLI −0.0003 [−0.0008 , 0.0003]
GECKO −0.0009 [−0.0016 , −0.0003]
GEN3G −0.0003 [−0.0005 , −0.0001]
GENR −0.0004 [−0.0008 , 0.0001]
GOYA −0.0001 [−0.0006 , 0.0004]
IOW 0.0002 [−0.0010 , 0.0014]
MEDALL −0.0003 [−0.0008 , 0.0002]
MOBA1 −0.0006 [−0.0010 , −0.0002]
MOBA2 −0.0008 [−0.0014 , −0.0003]
MOBA3 −0.0009 [−0.0025 , 0.0007]
NFCS −0.0002 [−0.0004 , 0.0000]
NEST −0.0001 [−0.0003 , 0.0002]
NHBCS 0.0003 [−0.0006 , 0.0012]
RICHS −0.0005 [−0.0014 , 0.0004]
VIVA −0.0007 [−0.0012 , −0.0002]
Fixed−effects meta−analysis −0.0003 [−0.0004 , −0.0002]
Random−effects meta−analysis −0.0003 [−0.0004 , −0.0002]
−0.0030
−0.0010
0.0010
Change in offspring DNA methylation per1kg/m2 increase in maternal BMI

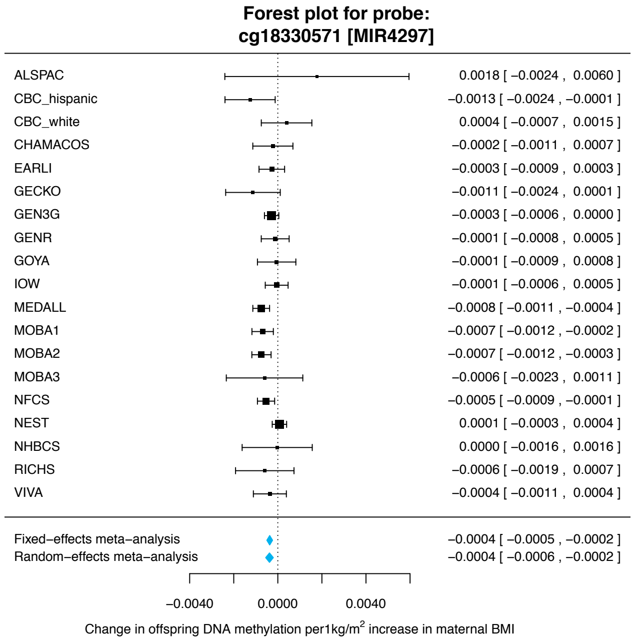
Forest plot for probe:
cg18330571 [MIR4297]
ALSPAC 0.0018 [−0.0024 , 0.0060]
CBC_hispanic −0.0013 [−0.0024 , −0.0001]
CBC_white 0.0004 [−0.0007 , 0.0015]
CHAMACOS −0.0002 [−0.0011 , 0.0007]
EARLI −0.0003 [−0.0009 , 0.0003]
GECKO −0.0011 [−0.0024 , 0.0001]
GEN3G −0.0003 [−0.0006 , 0.0000]
GENR −0.0001 [−0.0008 , 0.0005]
GOYA −0.0001 [−0.0009 , 0.0008]
IOW −0.0001 [−0.0006 , 0.0005]
MEDALL −0.0008 [−0.0011 , −0.0004]
MOBA1 −0.0007 [−0.0012 , −0.0002]
MOBA2 −0.0007 [−0.0012 , −0.0003]
MOBA3 −0.0006 [−0.0023 , 0.0011]
NFCS −0.0005 [−0.0009 , −0.0001]
NEST 0.0001 [−0.0003 , 0.0004]
NHBCS 0.0000 [−0.0016 , 0.0016]
RICHS −0.0006 [−0.0019 , 0.0007]
VIVA −0.0004 [−0.0011 , 0.0004]
Fixed−effects meta−analysis −0.0004 [−0.0005 , −0.0002]
Random−effects meta−analysis −0.0004 [−0.0006 , −0.0002]
−0.0040
0.0000
0.0040
Change in offspring DNA methylation per1kg/m2 increase in maternal BMI

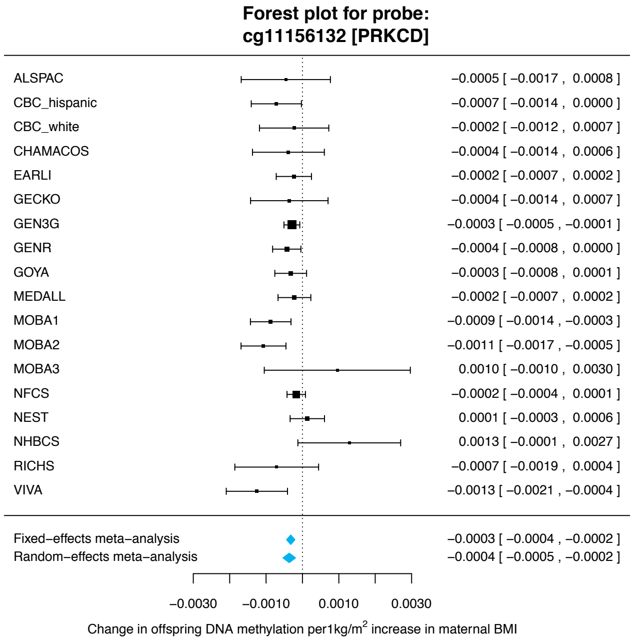
Forest plot for probe:
cg11156132 [PRKCD]
ALSPAC −0.0005 [−0.0017 , 0.0008]
CBC_hispanic −0.0007 [−0.0014 , 0.0000]
CBC_white −0.0002 [−0.0012 , 0.0007]
CHAMACOS −0.0004 [−0.0014 , 0.0006]
EARLI −0.0002 [−0.0007 , 0.0002]
GECKO −0.0004 [−0.0014 , 0.0007]
GEN3G −0.0003 [−0.0005 , −0.0001]
GENR −0.0004 [−0.0008 , 0.0000]
GOYA −0.0003 [−0.0008 , 0.0001]
MEDALL −0.0002 [−0.0007 , 0.0002]
MOBA1 −0.0009 [−0.0014 , −0.0003]
MOBA2 −0.0011 [−0.0017 , −0.0005]
MOBA3 0.0010 [−0.0010 , 0.0030]
NFCS −0.0002 [−0.0004 , 0.0001]
NEST 0.0001 [−0.0003 , 0.0006]
NHBCS 0.0013 [−0.0001 , 0.0027]
RICHS −0.0007 [−0.0019 , 0.0004]
VIVA −0.0013 [−0.0021 , −0.0004]
Fixed−effects meta−analysis −0.0003 [−0.0004 , −0.0002]
Random−effects meta−analysis −0.0004 [−0.0005 , −0.0002]
−0.0030
−0.0010
0.0010
0.0030
Change in offspring DNA methylation per1kg/m2 increase in maternal BMI

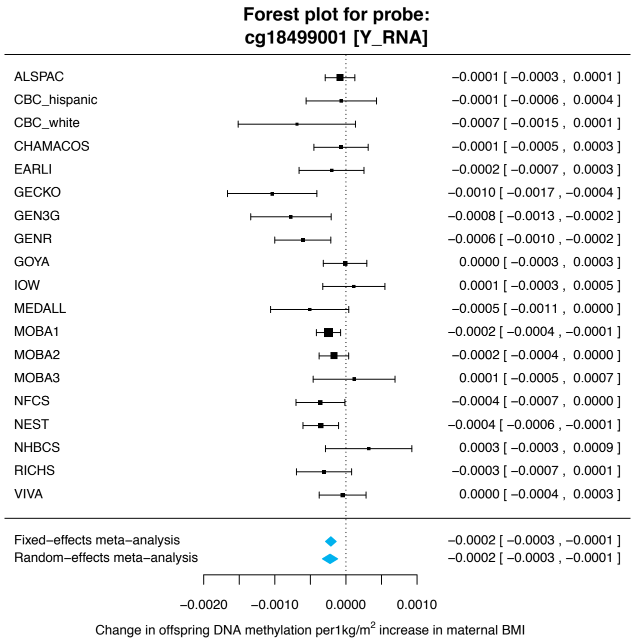
Forest plot for probe:
cg18499001 [Y_RNA]
ALSPAC −0.0001 [−0.0003 , 0.0001]
CBC_hispanic −0.0001 [−0.0006 , 0.0004]
CBC_white −0.0007 [−0.0015 , 0.0001]
CHAMACOS −0.0001 [−0.0005 , 0.0003]
EARLI −0.0002 [−0.0007 , 0.0003]
GECKO −0.0010 [−0.0017 , −0.0004]
GEN3G −0.0008 [−0.0013 , −0.0002]
GENR −0.0006 [−0.0010 , −0.0002]
GOYA 0.0000 [−0.0003 , 0.0003]
IOW 0.0001 [−0.0003 , 0.0005]
MEDALL −0.0005 [−0.0011 , 0.0000]
MOBA1 −0.0002 [−0.0004 , −0.0001]
MOBA2 −0.0002 [−0.0004 , 0.0000]
MOBA3 0.0001 [−0.0005 , 0.0007]
NFCS −0.0004 [−0.0007 , 0.0000]
NEST −0.0004 [−0.0006 , −0.0001]
NHBCS 0.0003 [−0.0003 , 0.0009]
RICHS −0.0003 [−0.0007 , 0.0001]
VIVA 0.0000 [−0.0004 , 0.0003]
Fixed−effects meta−analysis −0.0002 [−0.0003 , −0.0001]
Random−effects meta−analysis −0.0002 [−0.0003 , −0.0001]
−0.0020 −0.0010 0.0000 0.0010
Change in offspring DNA methylation per1kg/m2 increase in maternal BMI

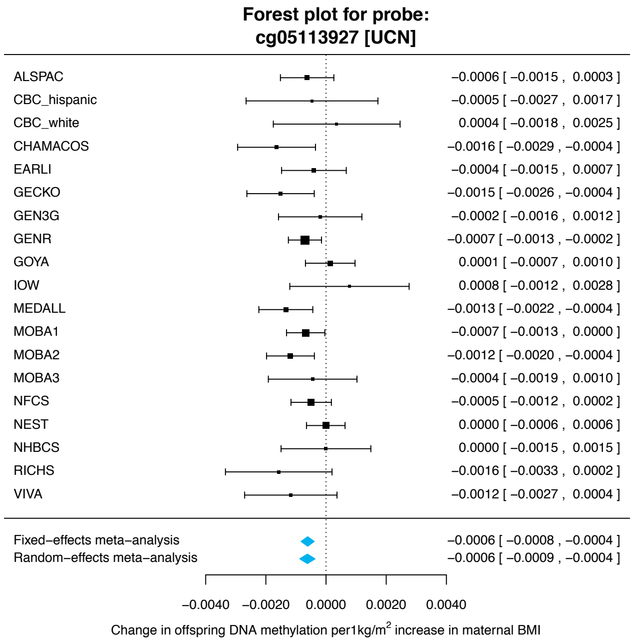
Forest plot for probe:
cg05113927 [UCN]
ALSPAC −0.0006 [−0.0015 , 0.0003]
CBC_hispanic −0.0005 [−0.0027 , 0.0017]
CBC_white 0.0004 [−0.0018 , 0.0025]
CHAMACOS −0.0016 [−0.0029 , −0.0004]
EARLI −0.0004 [−0.0015 , 0.0007]
GECKO −0.0015 [−0.0026 , −0.0004]
GEN3G −0.0002 [−0.0016 , 0.0012]
GENR −0.0007 [−0.0013 , −0.0002]
GOYA 0.0001 [−0.0007 , 0.0010]
IOW 0.0008 [−0.0012 , 0.0028]
MEDALL −0.0013 [−0.0022 , −0.0004]
MOBA1 −0.0007 [−0.0013 , 0.0000]
MOBA2 −0.0012 [−0.0020 , −0.0004]
MOBA3 −0.0004 [−0.0019 , 0.0010]
NFCS −0.0005 [−0.0012 , 0.0002]
NEST 0.0000 [−0.0006 , 0.0006]
NHBCS 0.0000 [−0.0015 , 0.0015]
RICHS −0.0016 [−0.0033 , 0.0002]
VIVA −0.0012 [−0.0027 , 0.0004]
Fixed−effects meta−analysis −0.0006 [−0.0008 , −0.0004]
Random−effects meta−analysis −0.0006 [−0.0009 , −0.0004]
−0.0040 −0.0020 0.0000 0.0020 0.0040
Change in offspring DNA methylation per1kg/m2 increase in maternal BMI

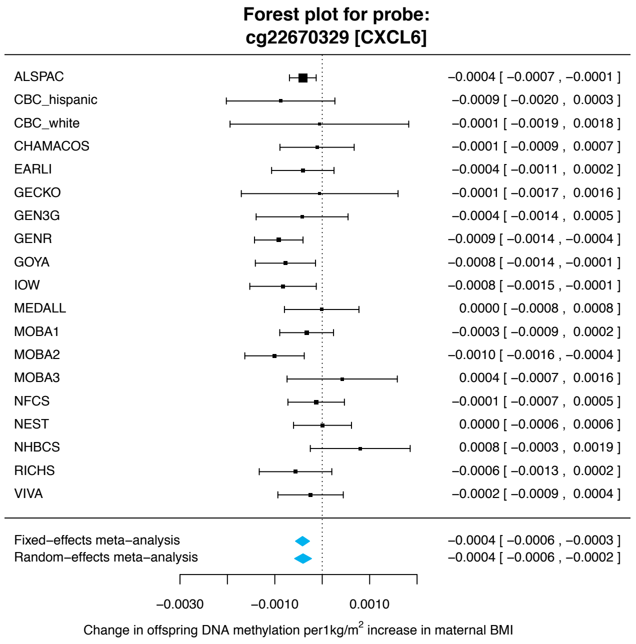
Forest plot for probe:
cg22670329 [CXCL6]
ALSPAC −0.0004 [−0.0007 , −0.0001]
CBC_hispanic −0.0009 [−0.0020 , 0.0003]
CBC_white −0.0001 [−0.0019 , 0.0018]
CHAMACOS −0.0001 [−0.0009 , 0.0007]
EARLI −0.0004 [−0.0011 , 0.0002]
GECKO −0.0001 [−0.0017 , 0.0016]
GEN3G −0.0004 [−0.0014 , 0.0005]
GENR −0.0009 [−0.0014 , −0.0004]
GOYA −0.0008 [−0.0014 , −0.0001]
IOW −0.0008 [−0.0015 , −0.0001]
MEDALL 0.0000 [−0.0008 , 0.0008]
MOBA1 −0.0003 [−0.0009 , 0.0002]
MOBA2 −0.0010 [−0.0016 , −0.0004]
MOBA3 0.0004 [−0.0007 , 0.0016]
NFCS −0.0001 [−0.0007 , 0.0005]
NEST 0.0000 [−0.0006 , 0.0006]
NHBCS 0.0008 [−0.0003 , 0.0019]
RICHS −0.0006 [−0.0013 , 0.0002]
VIVA −0.0002 [−0.0009 , 0.0004]
Fixed−effects meta−analysis −0.0004 [−0.0006 , −0.0003]
Random−effects meta−analysis −0.0004 [−0.0006 , −0.0002]
−0.0030 −0.0010 0.0010
Change in offspring DNA methylation per1kg/m2 increase in maternal BMI

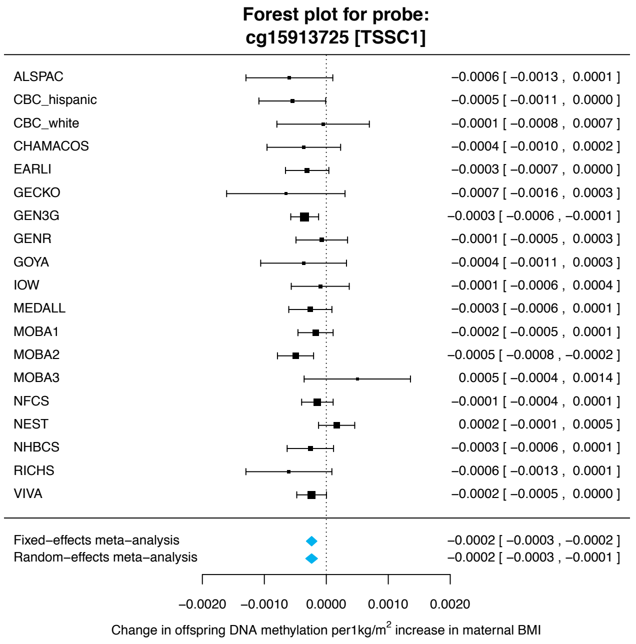
Forest plot for probe:
cg15913725 [TSSC1]
ALSPAC −0.0006 [−0.0013 , 0.0001]
CBC_hispanic −0.0005 [−0.0011 , 0.0000]
CBC_white −0.0001 [−0.0008 , 0.0007]
CHAMACOS −0.0004 [−0.0010 , 0.0002]
EARLI −0.0003 [−0.0007 , 0.0000]
GECKO −0.0007 [−0.0016 , 0.0003]
GEN3G −0.0003 [−0.0006 , −0.0001]
GENR −0.0001 [−0.0005 , 0.0003]
GOYA −0.0004 [−0.0011 , 0.0003]
IOW −0.0001 [−0.0006 , 0.0004]
MEDALL −0.0003 [−0.0006 , 0.0001]
MOBA1 −0.0002 [−0.0005 , 0.0001]
MOBA2 −0.0005 [−0.0008 , −0.0002]
MOBA3 0.0005 [−0.0004 , 0.0014]
NFCS −0.0001 [−0.0004 , 0.0001]
NEST 0.0002 [−0.0001 , 0.0005]
NHBCS −0.0003 [−0.0006 , 0.0001]
RICHS −0.0006 [−0.0013 , 0.0001]
VIVA −0.0002 [−0.0005 , 0.0000]
Fixed−effects meta−analysis −0.0002 [−0.0003 , −0.0002]
Random−effects meta−analysis −0.0002 [−0.0003 , −0.0001]
−0.0020 −0.0010 0.0000 0.0010 0.0020
Change in offspring DNA methylation per1kg/m2 increase in maternal BMI

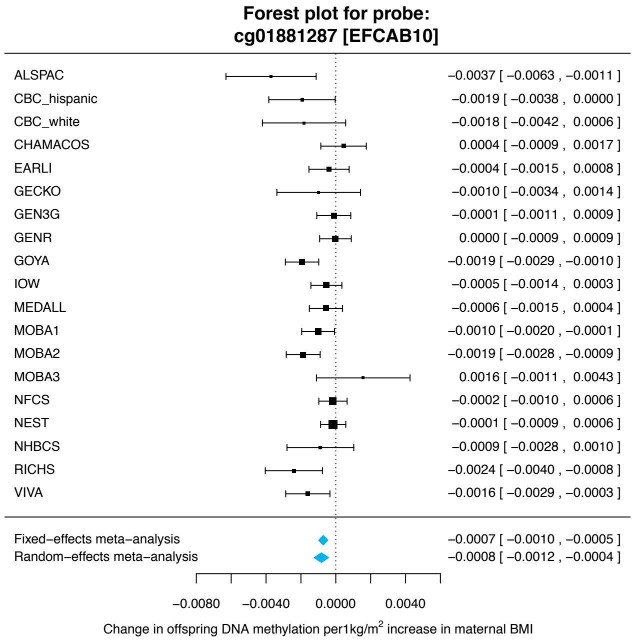
Forest plot for probe:
cg01881287 [EFCAB10]
ALSPAC −0.0037 [−0.0063 , −0.0011]
CBC_hispanic −0.0019 [−0.0038 , 0.0000]
CBC_white −0.0018 [−0.0042 , 0.0006]
CHAMACOS 0.0004 [−0.0009 , 0.0017]
EARLI −0.0004 [−0.0015 , 0.0008]
GECKO −0.0010 [−0.0034 , 0.0014]
GEN3G −0.0001 [−0.0011 , 0.0009]
GENR 0.0000 [−0.0009 , 0.0009]
GOYA −0.0019 [−0.0029 , −0.0010]
IOW −0.0005 [−0.0014 , 0.0003]
MEDALL −0.0006 [−0.0015 , 0.0004]
MOBA1 −0.0010 [−0.0020 , −0.0001]
MOBA2 −0.0019 [−0.0028 , −0.0009]
MOBA3 0.0016 [−0.0011 , 0.0043]
NFCS −0.0002 [−0.0010 , 0.0006]
NEST −0.0001 [−0.0009 , 0.0006]
NHBCS −0.0009 [−0.0028 , 0.0010]
RICHS −0.0024 [−0.0040 , −0.0008]
VIVA −0.0016 [−0.0029 , −0.0003]
Fixed−effects meta−analysis −0.0007 [−0.0010 , −0.0005]
Random−effects meta−analysis −0.0008 [−0.0012 , −0.0004]
−0.0080 −0.0040 0.0000 0.0040
Change in offspring DNA methylation per1kg/m2 increase in maternal BMI

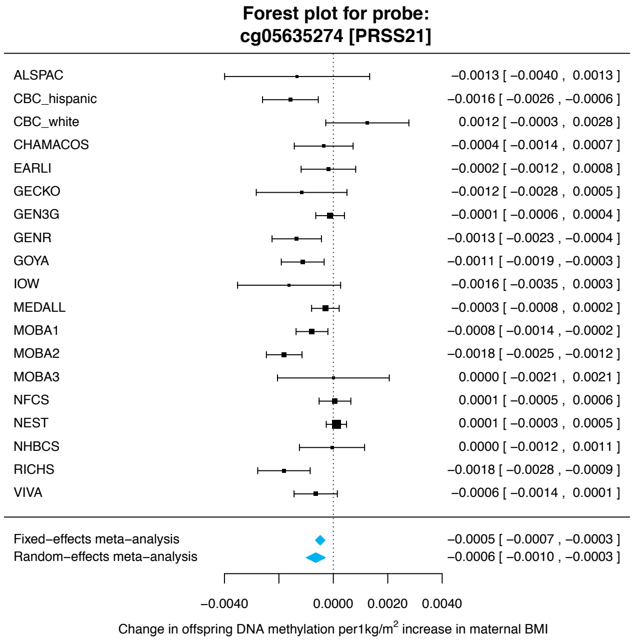
Forest plot for probe:
cg05635274 [PRSS21]
ALSPAC −0.0013 [−0.0040 , 0.0013]
CBC_hispanic −0.0016 [−0.0026 , −0.0006]
CBC_white 0.0012 [−0.0003 , 0.0028]
CHAMACOS −0.0004 [−0.0014 , 0.0007]
EARLI −0.0002 [−0.0012 , 0.0008]
GECKO −0.0012 [−0.0028 , 0.0005]
GEN3G −0.0001 [−0.0006 , 0.0004]
GENR −0.0013 [−0.0023 , −0.0004]
GOYA −0.0011 [−0.0019 , −0.0003]
IOW −0.0016 [−0.0035 , 0.0003]
MEDALL −0.0003 [−0.0008 , 0.0002]
MOBA1 −0.0008 [−0.0014 , −0.0002]
MOBA2 −0.0018 [−0.0025 , −0.0012]
MOBA3 0.0000 [−0.0021 , 0.0021]
NFCS 0.0001 [−0.0005 , 0.0006]
NEST 0.0001 [−0.0003 , 0.0005]
NHBCS 0.0000 [−0.0012 , 0.0011]
RICHS −0.0018 [−0.0028 , −0.0009]
VIVA −0.0006 [−0.0014 , 0.0001]
Fixed−effects meta−analysis −0.0005 [−0.0007 , −0.0003]
Random−effects meta−analysis −0.0006 [−0.0010 , −0.0003]
−0.0040 0.0000 0.0020 0.0040
Change in offspring DNA methylation per1kg/m2 increase in maternal BMI

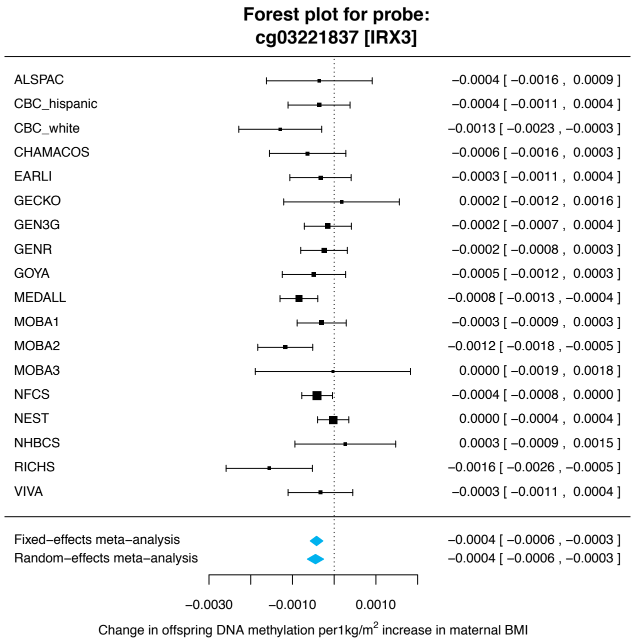
Forest plot for probe:
cg03221837 [IRX3]
ALSPAC −0.0004 [−0.0016 , 0.0009]
CBC_hispanic −0.0004 [−0.0011 , 0.0004]
CBC_white −0.0013 [−0.0023 , −0.0003]
CHAMACOS −0.0006 [−0.0016 , 0.0003]
EARLI −0.0003 [−0.0011 , 0.0004]
GECKO 0.0002 [−0.0012 , 0.0016]
GEN3G −0.0002 [−0.0007 , 0.0004]
GENR −0.0002 [−0.0008 , 0.0003]
GOYA −0.0005 [−0.0012 , 0.0003]
MEDALL −0.0008 [−0.0013 , −0.0004]
MOBA1 −0.0003 [−0.0009 , 0.0003]
MOBA2 −0.0012 [−0.0018 , −0.0005]
MOBA3 0.0000 [−0.0019 , 0.0018]
NFCS −0.0004 [−0.0008 , 0.0000]
NEST 0.0000 [−0.0004 , 0.0004]
NHBCS 0.0003 [−0.0009 , 0.0015]
RICHS −0.0016 [−0.0026 , −0.0005]
VIVA −0.0003 [−0.0011 , 0.0004]
Fixed−effects meta−analysis −0.0004 [−0.0006 , −0.0003]
Random−effects meta−analysis −0.0004 [−0.0006 , −0.0003]
−0.0030 −0.0010 0.0010
Change in offspring DNA methylation per1kg/m2 increase in maternal BMI

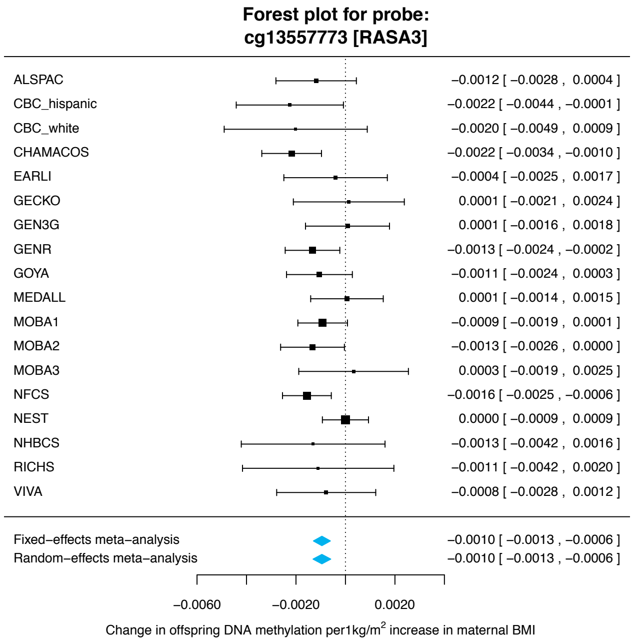
Forest plot for probe:
cg13557773 [RASA3]
ALSPAC −0.0012 [−0.0028 , 0.0004]
CBC_hispanic −0.0022 [−0.0044 , −0.0001]
CBC_white −0.0020 [−0.0049 , 0.0009]
CHAMACOS −0.0022 [−0.0034 , −0.0010]
EARLI −0.0004 [−0.0025 , 0.0017]
GECKO 0.0001 [−0.0021 , 0.0024]
GEN3G 0.0001 [−0.0016 , 0.0018]
GENR −0.0013 [−0.0024 , −0.0002]
GOYA −0.0011 [−0.0024 , 0.0003]
MEDALL 0.0001 [−0.0014 , 0.0015]
MOBA1 −0.0009 [−0.0019 , 0.0001]
MOBA2 −0.0013 [−0.0026 , 0.0000]
MOBA3 0.0003 [−0.0019 , 0.0025]
NFCS −0.0016 [−0.0025 , −0.0006]
NEST 0.0000 [−0.0009 , 0.0009]
NHBCS −0.0013 [−0.0042 , 0.0016]
RICHS −0.0011 [−0.0042 , 0.0020]
VIVA −0.0008 [−0.0028 , 0.0012]
Fixed−effects meta−analysis −0.0010 [−0.0013 , −0.0006]
Random−effects meta−analysis −0.0010 [−0.0013 , −0.0006]
−0.0060 −0.0020 0.0020
Change in offspring DNA methylation per1kg/m2 increase in maternal BMI

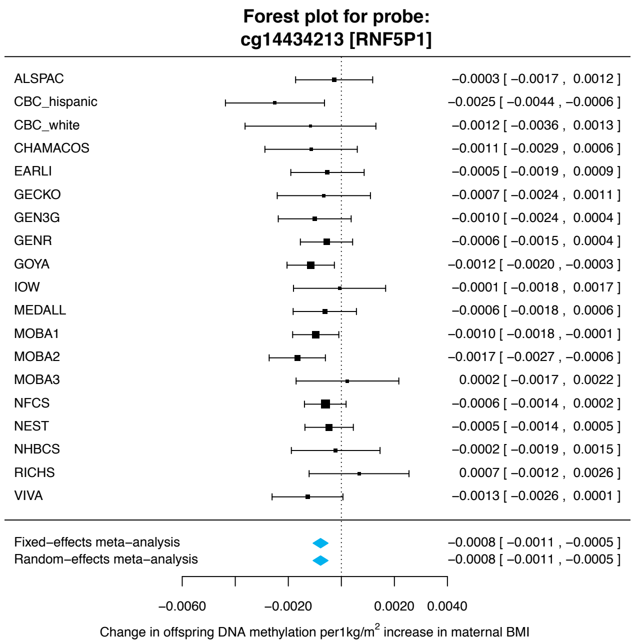
Forest plot for probe:
cg14434213 [RNF5P1]
ALSPAC −0.0003 [−0.0017 , 0.0012]
CBC_hispanic −0.0025 [−0.0044 , −0.0006]
CBC_white −0.0012 [−0.0036 , 0.0013]
CHAMACOS −0.0011 [−0.0029 , 0.0006]
EARLI −0.0005 [−0.0019 , 0.0009]
GECKO −0.0007 [−0.0024 , 0.0011]
GEN3G −0.0010 [−0.0024 , 0.0004]
GENR −0.0006 [−0.0015 , 0.0004]
GOYA −0.0012 [−0.0020 , −0.0003]
IOW −0.0001 [−0.0018 , 0.0017]
MEDALL −0.0006 [−0.0018 , 0.0006]
MOBA1 −0.0010 [−0.0018 , −0.0001]
MOBA2 −0.0017 [−0.0027 , −0.0006]
MOBA3 0.0002 [−0.0017 , 0.0022]
NFCS −0.0006 [−0.0014 , 0.0002]
NEST −0.0005 [−0.0014 , 0.0005]
NHBCS −0.0002 [−0.0019 , 0.0015]
RICHS 0.0007 [−0.0012 , 0.0026]
VIVA −0.0013 [−0.0026 , 0.0001]
Fixed−effects meta−analysis −0.0008 [−0.0011 , −0.0005]
Random−effects meta−analysis −0.0008 [−0.0011 , −0.0005]
−0.0060 −0.0020 0.0020 0.0040
Change in offspring DNA methylation per1kg/m2 increase in maternal BMI

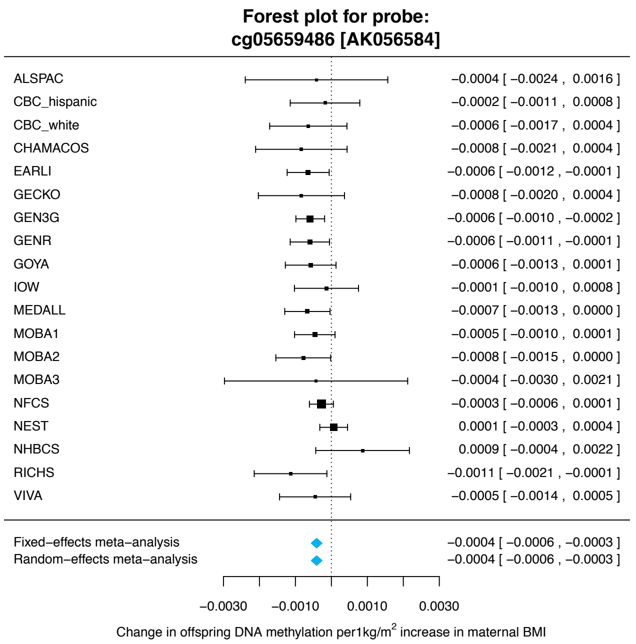
Forest plot for probe:
cg05659486 [AK056584]
ALSPAC −0.0004 [−0.0024 , 0.0016]
CBC_hispanic −0.0002 [−0.0011 , 0.0008]
CBC_white −0.0006 [−0.0017 , 0.0004]
CHAMACOS −0.0008 [−0.0021 , 0.0004]
EARLI −0.0006 [−0.0012 , −0.0001]
GECKO −0.0008 [−0.0020 , 0.0004]
GEN3G −0.0006 [−0.0010 , −0.0002]
GENR −0.0006 [−0.0011 , −0.0001]
GOYA −0.0006 [−0.0013 , 0.0001]
IOW −0.0001 [−0.0010 , 0.0008]
MEDALL −0.0007 [−0.0013 , 0.0000]
MOBA1 −0.0005 [−0.0010 , 0.0001]
MOBA2 −0.0008 [−0.0015 , 0.0000]
MOBA3 −0.0004 [−0.0030 , 0.0021]
NFCS −0.0003 [−0.0006 , 0.0001]
NEST 0.0001 [−0.0003 , 0.0004]
NHBCS 0.0009 [−0.0004 , 0.0022]
RICHS −0.0011 [−0.0021 , −0.0001]
VIVA −0.0005 [−0.0014 , 0.0005]
Fixed−effects meta−analysis −0.0004 [−0.0006 , −0.0003]
Random−effects meta−analysis −0.0004 [−0.0006 , −0.0003]
−0.0030 −0.0010 0.0010 0.0030
Change in offspring DNA methylation per1kg/m2 increase in maternal BMI

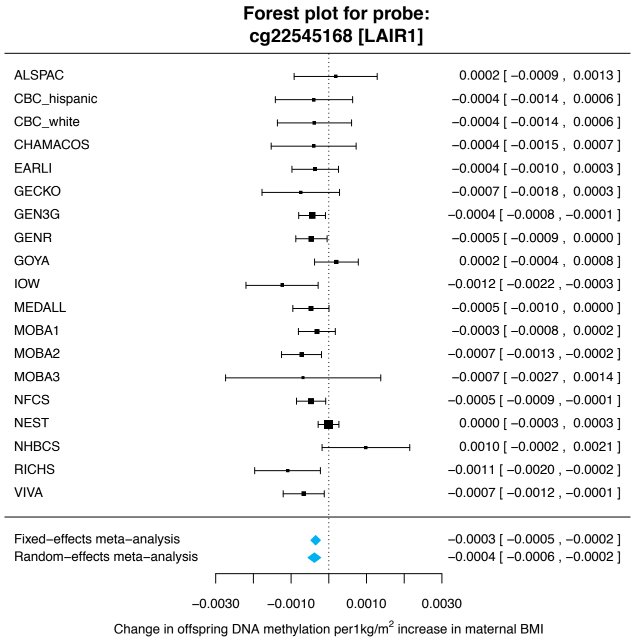
Forest plot for probe:
cg22545168 [LAIR1]
ALSPAC 0.0002 [−0.0009 , 0.0013]
CBC_hispanic −0.0004 [−0.0014 , 0.0006]
CBC_white −0.0004 [−0.0014 , 0.0006]
CHAMACOS −0.0004 [−0.0015 , 0.0007]
EARLI −0.0004 [−0.0010 , 0.0003]
GECKO −0.0007 [−0.0018 , 0.0003]
GEN3G −0.0004 [−0.0008 , −0.0001]
GENR −0.0005 [−0.0009 , 0.0000]
GOYA 0.0002 [−0.0004 , 0.0008]
IOW −0.0012 [−0.0022 , −0.0003]
MEDALL −0.0005 [−0.0010 , 0.0000]
MOBA1 −0.0003 [−0.0008 , 0.0002]
MOBA2 −0.0007 [−0.0013 , −0.0002]
MOBA3 −0.0007 [−0.0027 , 0.0014]
NFCS −0.0005 [−0.0009 , −0.0001]
NEST 0.0000 [−0.0003 , 0.0003]
NHBCS 0.0010 [−0.0002 , 0.0021]
RICHS −0.0011 [−0.0020 , −0.0002]
VIVA −0.0007 [−0.0012 , −0.0001]
Fixed−effects meta−analysis −0.0003 [−0.0005 , −0.0002]
Random−effects meta−analysis −0.0004 [−0.0006 , −0.0002]
−0.0030 −0.0010 0.0010 0.0030
Change in offspring DNA methylation per1kg/m2 increase in maternal BMI

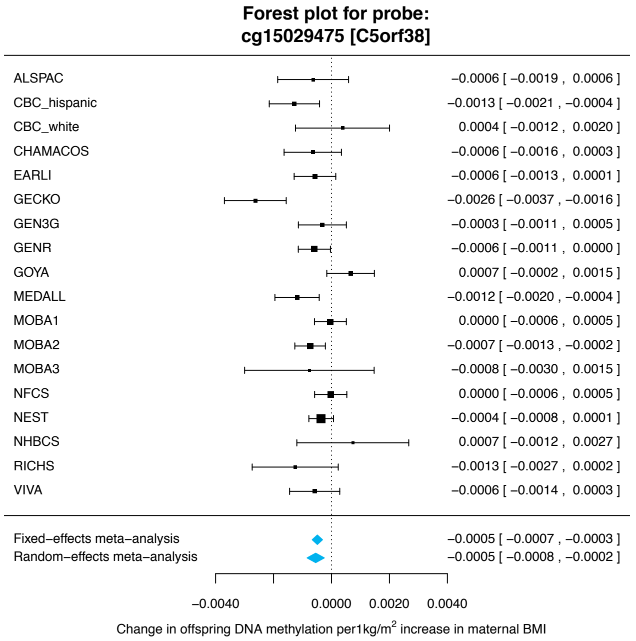
Forest plot for probe:
cg15029475 [C5orf38]
ALSPAC −0.0006 [−0.0019 , 0.0006]
CBC_hispanic −0.0013 [−0.0021 , −0.0004]
CBC_white 0.0004 [−0.0012 , 0.0020]
CHAMACOS −0.0006 [−0.0016 , 0.0003]
EARLI −0.0006 [−0.0013 , 0.0001]
GECKO −0.0026 [−0.0037 , −0.0016]
GEN3G −0.0003 [−0.0011 , 0.0005]
GENR −0.0006 [−0.0011 , 0.0000]
GOYA 0.0007 [−0.0002 , 0.0015]
MEDALL −0.0012 [−0.0020 , −0.0004]
MOBA1 0.0000 [−0.0006 , 0.0005]
MOBA2 −0.0007 [−0.0013 , −0.0002]
MOBA3 −0.0008 [−0.0030 , 0.0015]
NFCS 0.0000 [−0.0006 , 0.0005]
NEST −0.0004 [−0.0008 , 0.0001]
NHBCS 0.0007 [−0.0012 , 0.0027]
RICHS −0.0013 [−0.0027 , 0.0002]
VIVA −0.0006 [−0.0014 , 0.0003]
Fixed−effects meta−analysis −0.0005 [−0.0007 , −0.0003]
Random−effects meta−analysis −0.0005 [−0.0008 , −0.0002]
−0.0040 0.0000 0.0020 0.0040
Change in offspring DNA methylation per1kg/m2 increase in maternal BMI

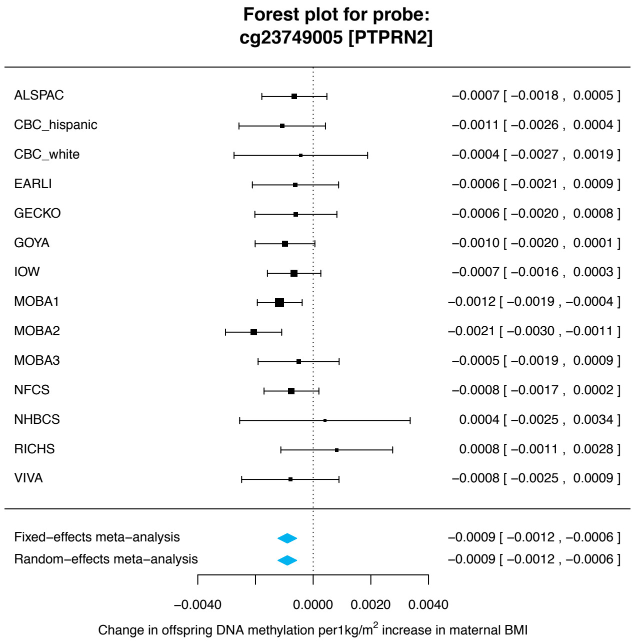
Forest plot for probe:
cg23749005 [PTPRN2]
ALSPAC −0.0007 [−0.0018 , 0.0005]
CBC_hispanic −0.0011 [−0.0026 , 0.0004]
CBC_white −0.0004 [−0.0027 , 0.0019]
EARLI −0.0006 [−0.0021 , 0.0009]
GECKO −0.0006 [−0.0020 , 0.0008]
GOYA −0.0010 [−0.0020 , 0.0001]
IOW −0.0007 [−0.0016 , 0.0003]
MOBA1 −0.0012 [−0.0019 , −0.0004]
MOBA2 −0.0021 [−0.0030 , −0.0011]
MOBA3 −0.0005 [−0.0019 , 0.0009]
NFCS −0.0008 [−0.0017 , 0.0002]
NHBCS 0.0004 [−0.0025 , 0.0034]
RICHS 0.0008 [−0.0011 , 0.0028]
VIVA −0.0008 [−0.0025 , 0.0009]
Fixed−effects meta−analysis −0.0009 [−0.0012 , −0.0006]
Random−effects meta−analysis −0.0009 [−0.0012 , −0.0006]
−0.0040
0.0000
0.0020
0.0040
Change in offspring DNA methylation per1kg/m2 increase in maternal BMI

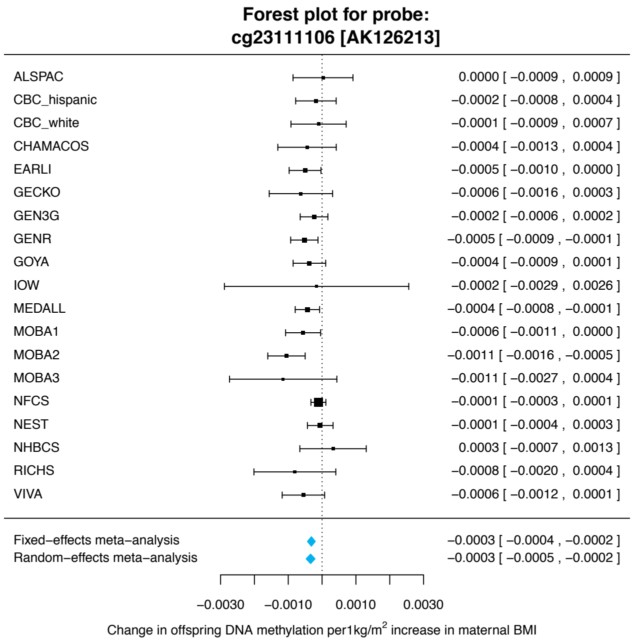
Forest plot for probe:
cg23111106 [AK126213]
ALSPAC 0.0000 [−0.0009 , 0.0009]
CBC_hispanic −0.0002 [−0.0008 , 0.0004]
CBC_white −0.0001 [−0.0009 , 0.0007]
CHAMACOS −0.0004 [−0.0013 , 0.0004]
EARLI −0.0005 [−0.0010 , 0.0000]
GECKO −0.0006 [−0.0016 , 0.0003]
GEN3G −0.0002 [−0.0006 , 0.0002]
GENR −0.0005 [−0.0009 , −0.0001]
GOYA −0.0004 [−0.0009 , 0.0001]
IOW −0.0002 [−0.0029 , 0.0026]
MEDALL −0.0004 [−0.0008 , −0.0001]
MOBA1 −0.0006 [−0.0011 , 0.0000]
MOBA2 −0.0011 [−0.0016 , −0.0005]
MOBA3 −0.0011 [−0.0027 , 0.0004]
NFCS −0.0001 [−0.0003 , 0.0001]
NEST −0.0001 [−0.0004 , 0.0003]
NHBCS 0.0003 [−0.0007 , 0.0013]
RICHS −0.0008 [−0.0020 , 0.0004]
VIVA −0.0006 [−0.0012 , 0.0001]
Fixed−effects meta−analysis −0.0003 [−0.0004 , −0.0002]
Random−effects meta−analysis −0.0003 [−0.0005 , −0.0002]
−0.0030
−0.0010
0.0010
0.0030
Change in offspring DNA methylation per1kg/m2 increase in maternal BMI

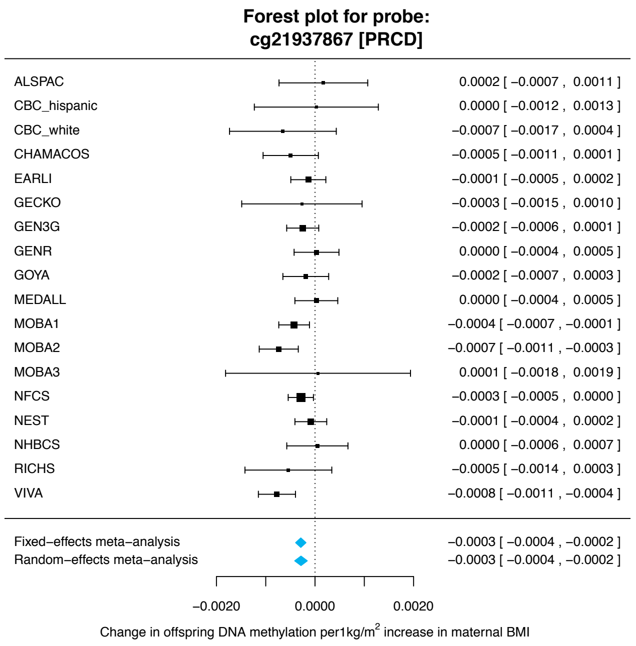
Forest plot for probe:
cg21937867 [PRCD]
ALSPAC 0.0002 [−0.0007 , 0.0011]
CBC_hispanic 0.0000 [−0.0012 , 0.0013]
CBC_white −0.0007 [−0.0017 , 0.0004]
CHAMACOS −0.0005 [−0.0011 , 0.0001]
EARLI −0.0001 [−0.0005 , 0.0002]
GECKO −0.0003 [−0.0015 , 0.0010]
GEN3G −0.0002 [−0.0006 , 0.0001]
GENR 0.0000 [−0.0004 , 0.0005]
GOYA −0.0002 [−0.0007 , 0.0003]
MEDALL 0.0000 [−0.0004 , 0.0005]
MOBA1 −0.0004 [−0.0007 , −0.0001]
MOBA2 −0.0007 [−0.0011 , −0.0003]
MOBA3 0.0001 [−0.0018 , 0.0019]
NFCS −0.0003 [−0.0005 , 0.0000]
NEST −0.0001 [−0.0004 , 0.0002]
NHBCS 0.0000 [−0.0006 , 0.0007]
RICHS −0.0005 [−0.0014 , 0.0003]
VIVA −0.0008 [−0.0011 , −0.0004]
Fixed−effects meta−analysis −0.0003 [−0.0004 , −0.0002]
Random−effects meta−analysis −0.0003 [−0.0004 , −0.0002]
−0.0020
0.0000
0.0020
Change in offspring DNA methylation per1kg/m2 increase in maternal BMI

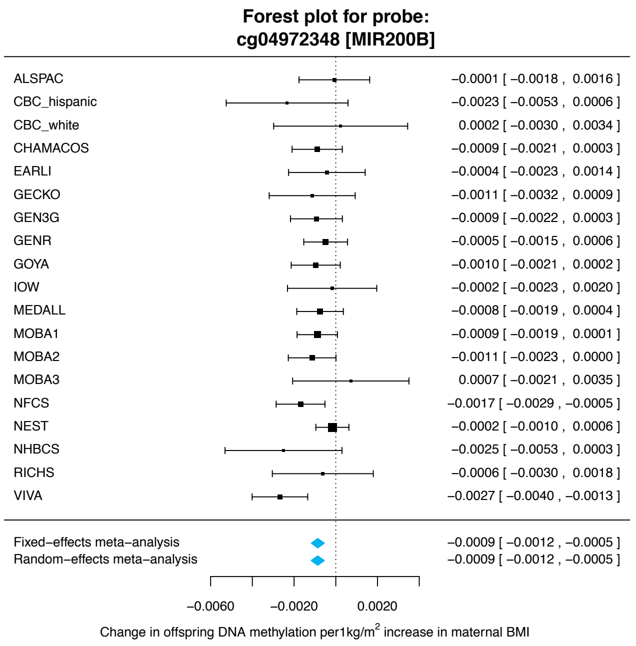
Forest plot for probe:
cg04972348 [MIR200B]
ALSPAC −0.0001 [−0.0018 , 0.0016]
CBC_hispanic −0.0023 [−0.0053 , 0.0006]
CBC_white 0.0002 [−0.0030 , 0.0034]
CHAMACOS −0.0009 [−0.0021 , 0.0003]
EARLI −0.0004 [−0.0023 , 0.0014]
GECKO −0.0011 [−0.0032 , 0.0009]
GEN3G −0.0009 [−0.0022 , 0.0003]
GENR −0.0005 [−0.0015 , 0.0006]
GOYA −0.0010 [−0.0021 , 0.0002]
IOW −0.0002 [−0.0023 , 0.0020]
MEDALL −0.0008 [−0.0019 , 0.0004]
MOBA1 −0.0009 [−0.0019 , 0.0001]
MOBA2 −0.0011 [−0.0023 , 0.0000]
MOBA3 0.0007 [−0.0021 , 0.0035]
NFCS −0.0017 [−0.0029 , −0.0005]
NEST −0.0002 [−0.0010 , 0.0006]
NHBCS −0.0025 [−0.0053 , 0.0003]
RICHS −0.0006 [−0.0030 , 0.0018]
VIVA −0.0027 [−0.0040 , −0.0013]
Fixed−effects meta−analysis −0.0009 [−0.0012 , −0.0005]
Random−effects meta−analysis −0.0009 [−0.0012 , −0.0005]
−0.0060
−0.0020
0.0020
Change in offspring DNA methylation per1kg/m2 increase in maternal BMI

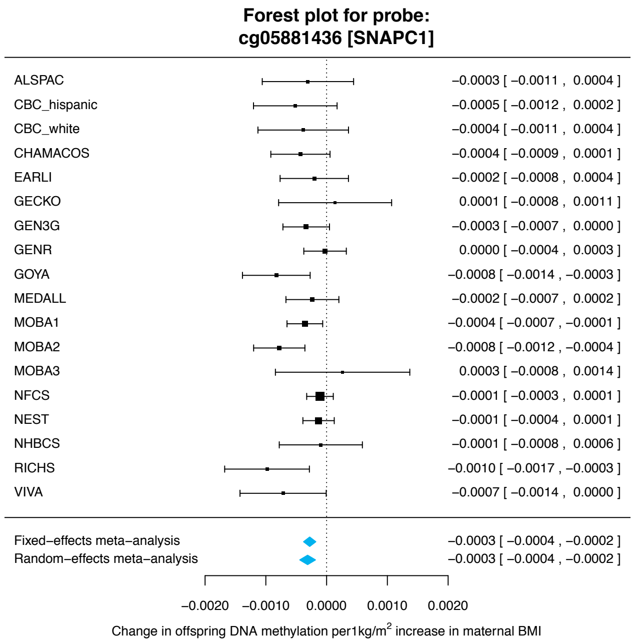
Forest plot for probe:
cg05881436 [SNAPC1]
ALSPAC −0.0003 [−0.0011 , 0.0004]
CBC_hispanic −0.0005 [−0.0012 , 0.0002]
CBC_white −0.0004 [−0.0011 , 0.0004]
CHAMACOS −0.0004 [−0.0009 , 0.0001]
EARLI −0.0002 [−0.0008 , 0.0004]
GECKO 0.0001 [−0.0008 , 0.0011]
GEN3G −0.0003 [−0.0007 , 0.0000]
GENR 0.0000 [−0.0004 , 0.0003]
GOYA −0.0008 [−0.0014 , −0.0003]
MEDALL −0.0002 [−0.0007 , 0.0002]
MOBA1 −0.0004 [−0.0007 , −0.0001]
MOBA2 −0.0008 [−0.0012 , −0.0004]
MOBA3 0.0003 [−0.0008 , 0.0014]
NFCS −0.0001 [−0.0003 , 0.0001]
NEST −0.0001 [−0.0004 , 0.0001]
NHBCS −0.0001 [−0.0008 , 0.0006]
RICHS −0.0010 [−0.0017 , −0.0003]
VIVA −0.0007 [−0.0014 , 0.0000]
Fixed−effects meta−analysis −0.0003 [−0.0004 , −0.0002]
Random−effects meta−analysis −0.0003 [−0.0004 , −0.0002]
−0.0020
−0.0010
0.0000
0.0010
0.0020
Change in offspring DNA methylation per1kg/m2 increase in maternal BMI

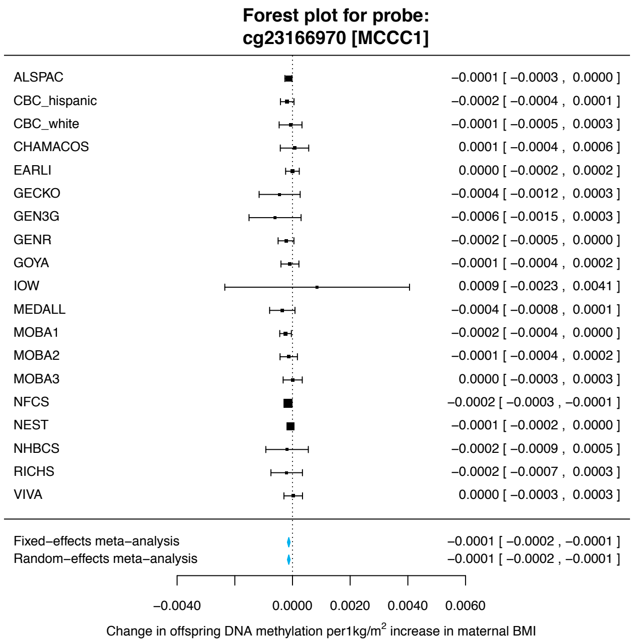
Forest plot for probe:
cg23166970 [MCCC1]
ALSPAC −0.0001 [−0.0003 , 0.0000]
CBC_hispanic −0.0002 [−0.0004 , 0.0001]
CBC_white −0.0001 [−0.0005 , 0.0003]
CHAMACOS 0.0001 [−0.0004 , 0.0006]
EARLI 0.0000 [−0.0002 , 0.0002]
GECKO −0.0004 [−0.0012 , 0.0003]
GEN3G −0.0006 [−0.0015 , 0.0003]
GENR −0.0002 [−0.0005 , 0.0000]
GOYA −0.0001 [−0.0004 , 0.0002]
IOW 0.0009 [−0.0023 , 0.0041]
MEDALL −0.0004 [−0.0008 , 0.0001]
MOBA1 −0.0002 [−0.0004 , 0.0000]
MOBA2 −0.0001 [−0.0004 , 0.0002]
MOBA3 0.0000 [−0.0003 , 0.0003]
NFCS −0.0002 [−0.0003 , −0.0001]
NEST −0.0001 [−0.0002 , 0.0000]
NHBCS −0.0002 [−0.0009 , 0.0005]
RICHS −0.0002 [−0.0007 , 0.0003]
VIVA 0.0000 [−0.0003 , 0.0003]
Fixed−effects meta−analysis −0.0001 [−0.0002 , −0.0001]
Random−effects meta−analysis −0.0001 [−0.0002 , −0.0001]
−0.0040
0.0000
0.0020
0.0040
0.0060
Change in offspring DNA methylation per1kg/m2 increase in maternal BMI

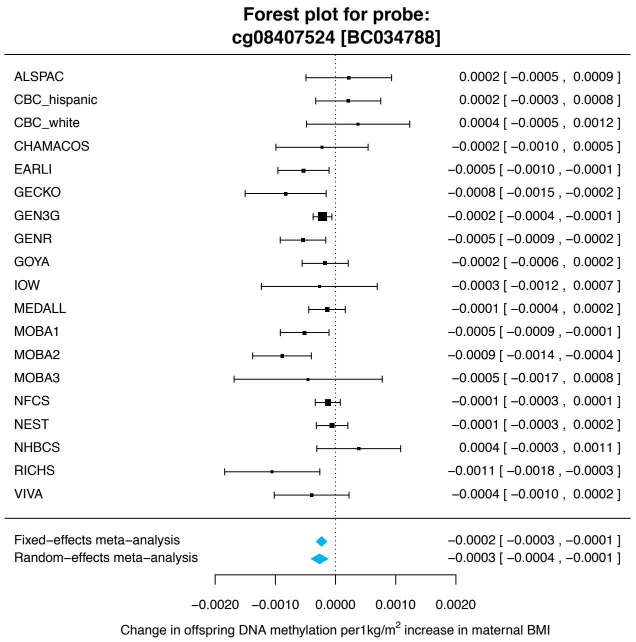
Forest plot for probe:
cg08407524 [BC034788]
ALSPAC 0.0002 [−0.0005 , 0.0009]
CBC_hispanic 0.0002 [−0.0003 , 0.0008]
CBC_white 0.0004 [−0.0005 , 0.0012]
CHAMACOS −0.0002 [−0.0010 , 0.0005]
EARLI −0.0005 [−0.0010 , −0.0001]
GECKO −0.0008 [−0.0015 , −0.0002]
GEN3G −0.0002 [−0.0004 , −0.0001]
GENR −0.0005 [−0.0009 , −0.0002]
GOYA −0.0002 [−0.0006 , 0.0002]
IOW −0.0003 [−0.0012 , 0.0007]
MEDALL −0.0001 [−0.0004 , 0.0002]
MOBA1 −0.0005 [−0.0009 , −0.0001]
MOBA2 −0.0009 [−0.0014 , −0.0004]
MOBA3 −0.0005 [−0.0017 , 0.0008]
NFCS −0.0001 [−0.0003 , 0.0001]
NEST −0.0001 [−0.0003 , 0.0002]
NHBCS 0.0004 [−0.0003 , 0.0011]
RICHS −0.0011 [−0.0018 , −0.0003]
VIVA −0.0004 [−0.0010 , 0.0002]
Fixed−effects meta−analysis −0.0002 [−0.0003 , −0.0001]
Random−effects meta−analysis −0.0003 [−0.0004 , −0.0001]
−0.0020 −0.0010 0.0000 0.0010 0.0020
Change in offspring DNA methylation per1kg/m2 increase in maternal BMI

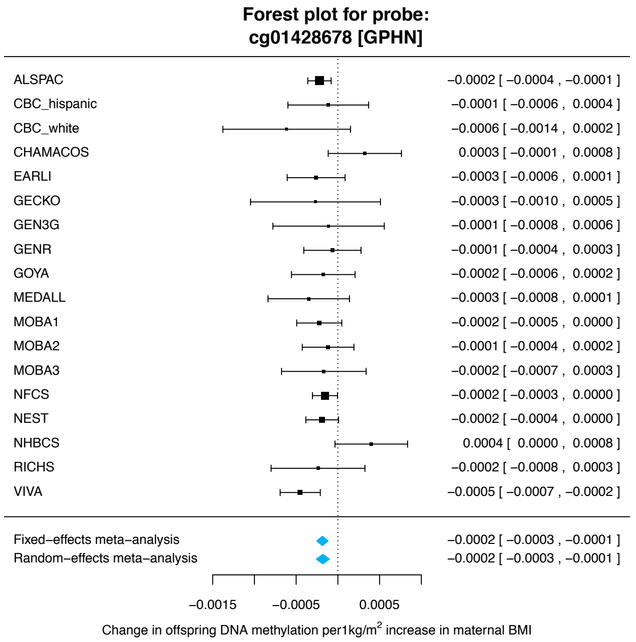
Forest plot for probe:
cg01428678 [GPHN]
ALSPAC −0.0002 [−0.0004 , −0.0001]
CBC_hispanic −0.0001 [−0.0006 , 0.0004]
CBC_white −0.0006 [−0.0014 , 0.0002]
CHAMACOS 0.0003 [−0.0001 , 0.0008]
EARLI −0.0003 [−0.0006 , 0.0001]
GECKO −0.0003 [−0.0010 , 0.0005]
GEN3G −0.0001 [−0.0008 , 0.0006]
GENR −0.0001 [−0.0004 , 0.0003]
GOYA −0.0002 [−0.0006 , 0.0002]
MEDALL −0.0003 [−0.0008 , 0.0001]
MOBA1 −0.0002 [−0.0005 , 0.0000]
MOBA2 −0.0001 [−0.0004 , 0.0002]
MOBA3 −0.0002 [−0.0007 , 0.0003]
NFCS −0.0002 [−0.0003 , 0.0000]
NEST −0.0002 [−0.0004 , 0.0000]
NHBCS 0.0004 [0.0000 , 0.0008]
RICHS −0.0002 [−0.0008 , 0.0003]
VIVA −0.0005 [−0.0007 , −0.0002]
Fixed−effects meta−analysis −0.0002 [−0.0003 , −0.0001]
Random−effects meta−analysis −0.0002 [−0.0003 , −0.0001]
−0.0015 −0.0005 0.0005
Change in offspring DNA methylation per1kg/m2 increase in maternal BMI

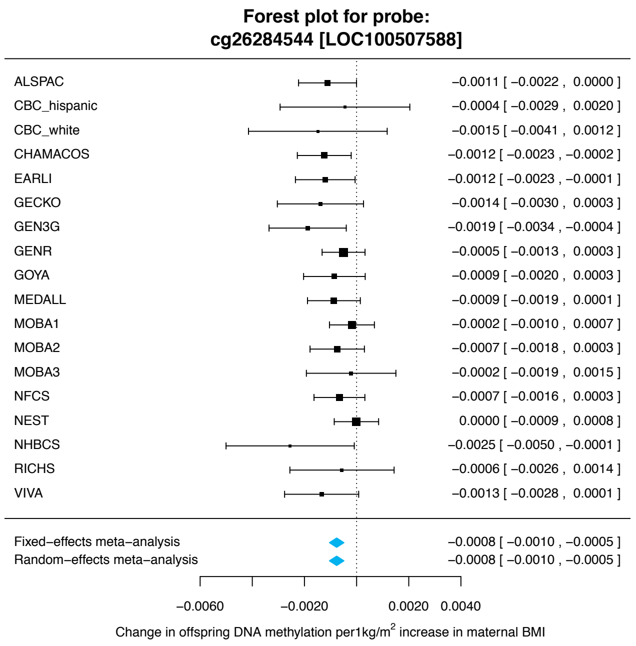
Forest plot for probe:
cg26284544 [LOC100507588]
ALSPAC −0.0011 [−0.0022 , 0.0000]
CBC_hispanic −0.0004 [−0.0029 , 0.0020]
CBC_white −0.0015 [−0.0041 , 0.0012]
CHAMACOS −0.0012 [−0.0023 , −0.0002]
EARLI −0.0012 [−0.0023 , −0.0001]
GECKO −0.0014 [−0.0030 , 0.0003]
GEN3G −0.0019 [−0.0034 , −0.0004]
GENR −0.0005 [−0.0013 , 0.0003]
GOYA −0.0009 [−0.0020 , 0.0003]
MEDALL −0.0009 [−0.0019 , 0.0001]
MOBA1 −0.0002 [−0.0010 , 0.0007]
MOBA2 −0.0007 [−0.0018 , 0.0003]
MOBA3 −0.0002 [−0.0019 , 0.0015]
NFCS −0.0007 [−0.0016 , 0.0003]
NEST 0.0000 [−0.0009 , 0.0008]
NHBCS −0.0025 [−0.0050 , −0.0001]
RICHS −0.0006 [−0.0026 , 0.0014]
VIVA −0.0013 [−0.0028 , 0.0001]
Fixed−effects meta−analysis −0.0008 [−0.0010 , −0.0005]
Random−effects meta−analysis −0.0008 [−0.0010 , −0.0005]
−0.0060 −0.0020 0.0020 0.0040
Change in offspring DNA methylation per1kg/m2 increase in maternal BMI

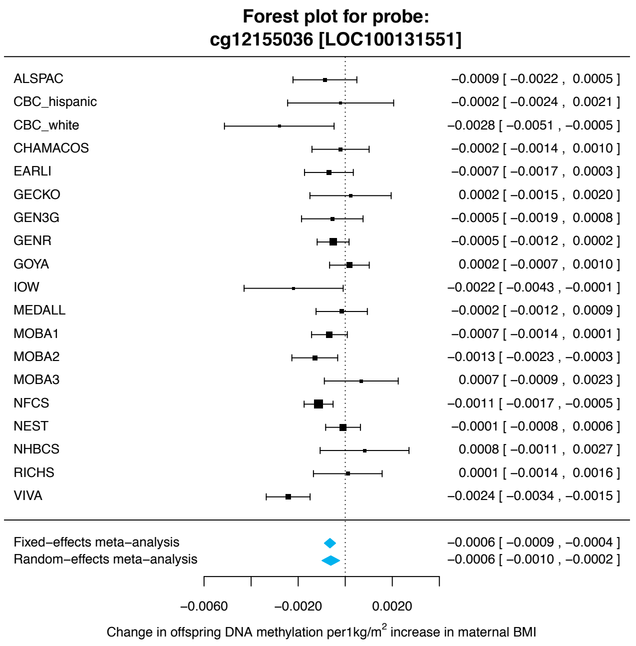
Forest plot for probe:
cg12155036 [LOC100131551]
ALSPAC −0.0009 [−0.0022 , 0.0005]
CBC_hispanic −0.0002 [−0.0024 , 0.0021]
CBC_white −0.0028 [−0.0051 , −0.0005]
CHAMACOS −0.0002 [−0.0014 , 0.0010]
EARLI −0.0007 [−0.0017 , 0.0003]
GECKO 0.0002 [−0.0015 , 0.0020]
GEN3G −0.0005 [−0.0019 , 0.0008]
GENR −0.0005 [−0.0012 , 0.0002]
GOYA 0.0002 [−0.0007 , 0.0010]
IOW −0.0022 [−0.0043 , −0.0001]
MEDALL −0.0002 [−0.0012 , 0.0009]
MOBA1 −0.0007 [−0.0014 , 0.0001]
MOBA2 −0.0013 [−0.0023 , −0.0003]
MOBA3 0.0007 [−0.0009 , 0.0023]
NFCS −0.0011 [−0.0017 , −0.0005]
NEST −0.0001 [−0.0008 , 0.0006]
NHBCS 0.0008 [−0.0011 , 0.0027]
RICHS 0.0001 [−0.0014 , 0.0016]
VIVA −0.0024 [−0.0034 , −0.0015]
Fixed−effects meta−analysis −0.0006 [−0.0009 , −0.0004]
Random−effects meta−analysis −0.0006 [−0.0010 , −0.0002]
−0.0060 −0.0020 0.0020
Change in offspring DNA methylation per1kg/m2 increase in maternal BMI

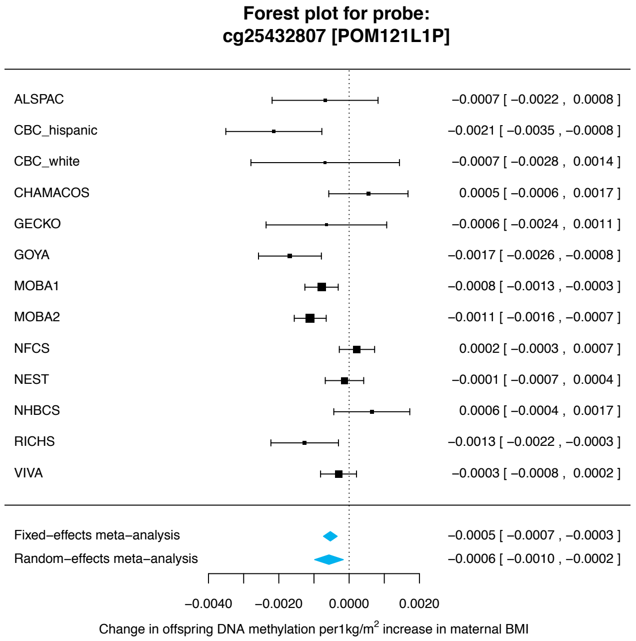
Forest plot for probe:
cg25432807 [POM121L1P]
ALSPAC −0.0007 [−0.0022 , 0.0008]
CBC_hispanic −0.0021 [−0.0035 , −0.0008]
CBC_white −0.0007 [−0.0028 , 0.0014]
CHAMACOS 0.0005 [−0.0006 , 0.0017]
GECKO −0.0006 [−0.0024 , 0.0011]
GOYA −0.0017 [−0.0026 , −0.0008]
MOBA1 −0.0008 [−0.0013 , −0.0003]
MOBA2 −0.0011 [−0.0016 , −0.0007]
NFCS 0.0002 [−0.0003 , 0.0007]
NEST −0.0001 [−0.0007 , 0.0004]
NHBCS 0.0006 [−0.0004 , 0.0017]
RICHS −0.0013 [−0.0022 , −0.0003]
VIVA −0.0003 [−0.0008 , 0.0002]
Fixed−effects meta−analysis −0.0005 [−0.0007 , −0.0003]
Random−effects meta−analysis −0.0006 [−0.0010 , −0.0002]
−0.0040 −0.0020 0.0000 0.0020
Change in offspring DNA methylation per1kg/m2 increase in maternal BMI

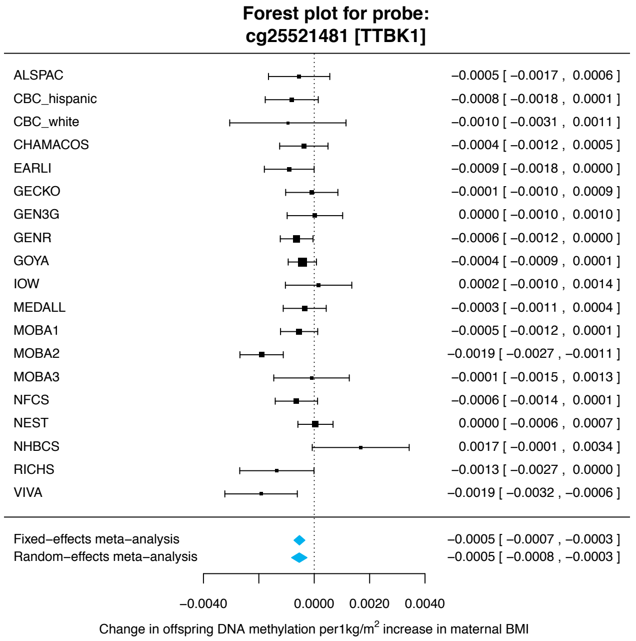
Forest plot for probe:
cg25521481 [TTBK1]
ALSPAC −0.0005 [−0.0017 , 0.0006]
CBC_hispanic −0.0008 [−0.0018 , 0.0001]
CBC_white −0.0010 [−0.0031 , 0.0011]
CHAMACOS −0.0004 [−0.0012 , 0.0005]
EARLI −0.0009 [−0.0018 , 0.0000]
GECKO −0.0001 [−0.0010 , 0.0009]
GEN3G 0.0000 [−0.0010 , 0.0010]
GENR −0.0006 [−0.0012 , 0.0000]
GOYA −0.0004 [−0.0009 , 0.0001]
IOW 0.0002 [−0.0010 , 0.0014]
MEDALL −0.0003 [−0.0011 , 0.0004]
MOBA1 −0.0005 [−0.0012 , 0.0001]
MOBA2 −0.0019 [−0.0027 , −0.0011]
MOBA3 −0.0001 [−0.0015 , 0.0013]
NFCS −0.0006 [−0.0014 , 0.0001]
NEST 0.0000 [−0.0006 , 0.0007]
NHBCS 0.0017 [−0.0001 , 0.0034]
RICHS −0.0013 [−0.0027 , 0.0000]
VIVA −0.0019 [−0.0032 , −0.0006]
Fixed−effects meta−analysis −0.0005 [−0.0007 , −0.0003]
Random−effects meta−analysis −0.0005 [−0.0008 , −0.0003]
−0.0040 0.0000 0.0020 0.0040
Change in offspring DNA methylation per1kg/m2 increase in maternal BMI

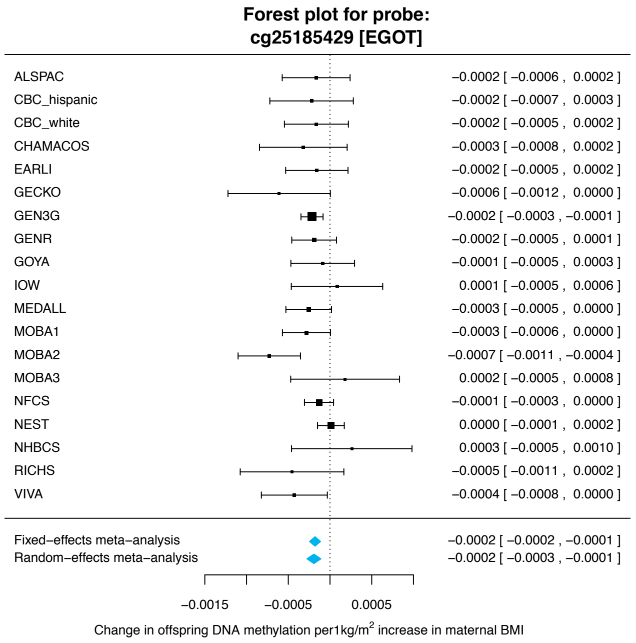
Forest plot for probe:
cg25185429 [EGOT]
ALSPAC −0.0002 [−0.0006 , 0.0002]
CBC_hispanic −0.0002 [−0.0007 , 0.0003]
CBC_white −0.0002 [−0.0005 , 0.0002]
CHAMACOS −0.0003 [−0.0008 , 0.0002]
EARLI −0.0002 [−0.0005 , 0.0002]
GECKO −0.0006 [−0.0012 , 0.0000]
GEN3G −0.0002 [−0.0003 , −0.0001]
GENR −0.0002 [−0.0005 , 0.0001]
GOYA −0.0001 [−0.0005 , 0.0003]
IOW 0.0001 [−0.0005 , 0.0006]
MEDALL −0.0003 [−0.0005 , 0.0000]
MOBA1 −0.0003 [−0.0006 , 0.0000]
MOBA2 −0.0007 [−0.0011 , −0.0004]
MOBA3 0.0002 [−0.0005 , 0.0008]
NFCS −0.0001 [−0.0003 , 0.0000]
NEST 0.0000 [−0.0001 , 0.0002]
NHBCS 0.0003 [−0.0005 , 0.0010]
RICHS −0.0005 [−0.0011 , 0.0002]
VIVA −0.0004 [−0.0008 , 0.0000]
Fixed−effects meta−analysis −0.0002 [−0.0002 , −0.0001]
Random−effects meta−analysis −0.0002 [−0.0003 , −0.0001]
−0.0015 −0.0005 0.0005
Change in offspring DNA methylation per1kg/m2 increase in maternal BMI

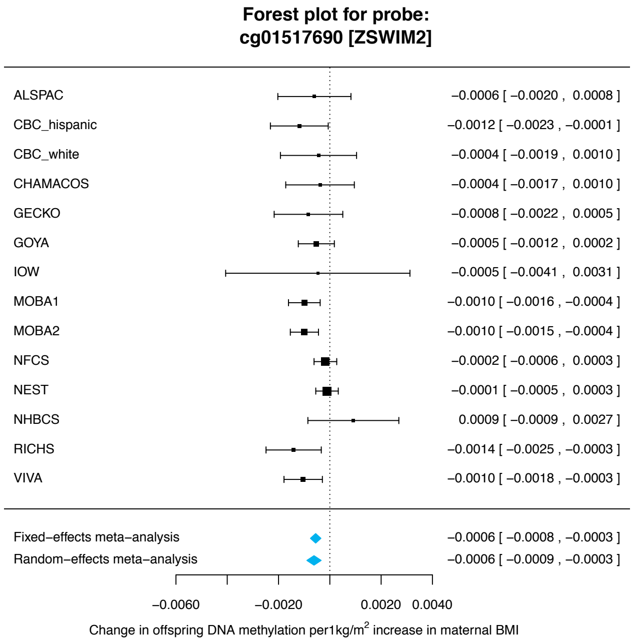
Forest plot for probe:
cg01517690 [ZSWIM2]
ALSPAC −0.0006 [−0.0020 , 0.0008]
CBC_hispanic −0.0012 [−0.0023 , −0.0001]
CBC_white −0.0004 [−0.0019 , 0.0010]
CHAMACOS −0.0004 [−0.0017 , 0.0010]
GECKO −0.0008 [−0.0022 , 0.0005]
GOYA −0.0005 [−0.0012 , 0.0002]
IOW −0.0005 [−0.0041 , 0.0031]
MOBA1 −0.0010 [−0.0016 , −0.0004]
MOBA2 −0.0010 [−0.0015 , −0.0004]
NFCS −0.0002 [−0.0006 , 0.0003]
NEST −0.0001 [−0.0005 , 0.0003]
NHBCS 0.0009 [−0.0009 , 0.0027]
RICHS −0.0014 [−0.0025 , −0.0003]
VIVA −0.0010 [−0.0018 , −0.0003]
Fixed−effects meta−analysis −0.0006 [−0.0008 , −0.0003]
Random−effects meta−analysis −0.0006 [−0.0009 , −0.0003]
−0.0060 −0.0020 0.0020 0.0040
Change in offspring DNA methylation per1kg/m2 increase in maternal BMI

Leave-one-out plots for the cell-adjusted continuous BMI model

Plots show –log10 P-values for meta-analyses with each cohort (x-axis) omitted. The red, full horizontal line indicates the –log10 P-value for the meta-analysis with all cohorts included. The black, dashed horizontal line indicates the Bonferroni cut off for genome-wide significance ($1.06 * 10^{-7}$).

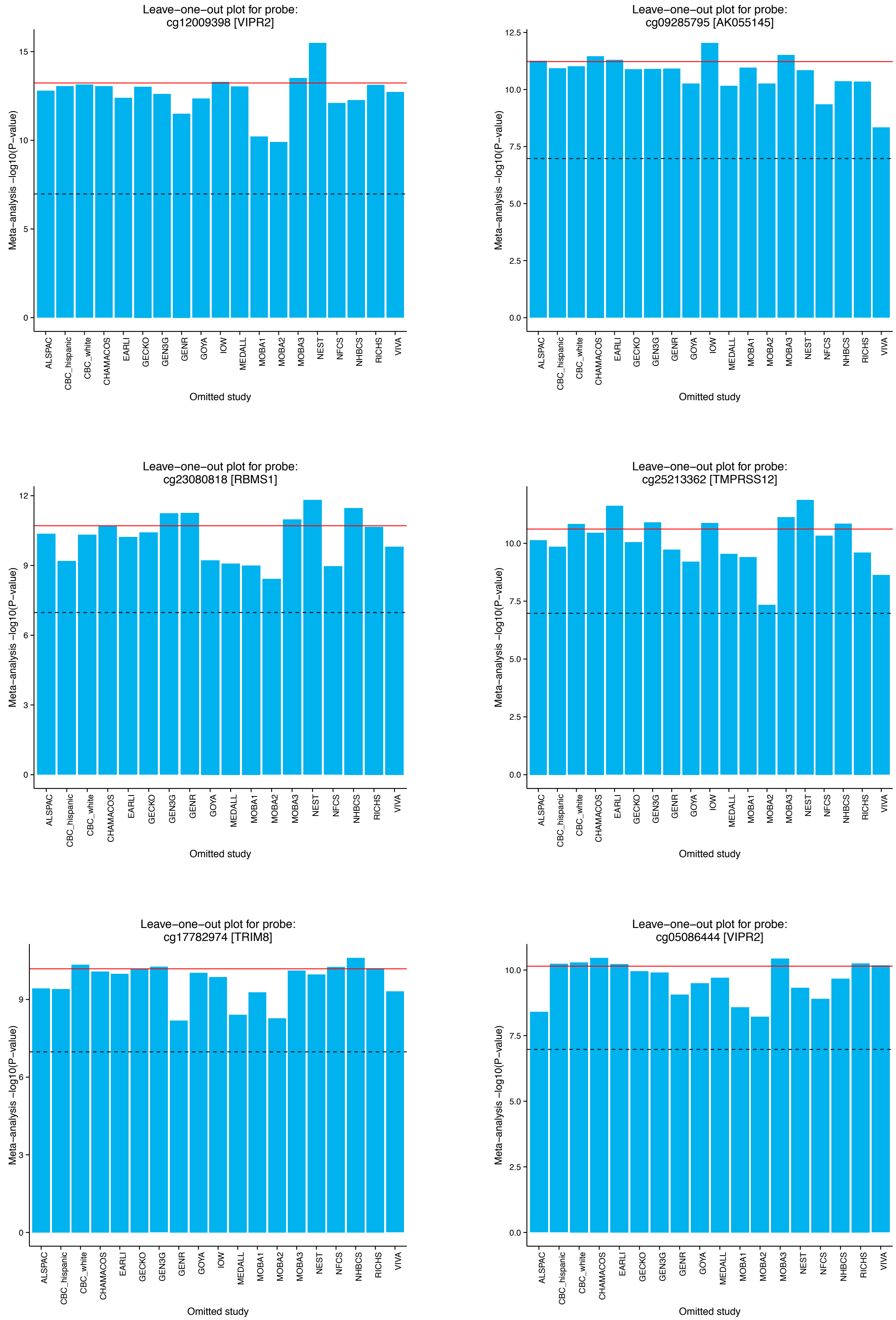
Leave−one−out plot for probe:
cg12009398 [VIPR2]
Meta−analysis −log10(P−value)
0
5
10
15
ALSPAC
CBC_hispanic
CBC_white
CHAMACOS
EARLI
GECKO
GEN3G
GENR
GOYA
IOW
MEDALL
MOBA1
MOBA2
MOBA3
NEST
NFCS
NHBCS
RICHS
VIVA
Omitted study
Leave−one−out plot for probe:
cg09285795 [AK055145]
Meta−analysis −log10(P−value)
0.0
2.5
5.0
7.5
10.0
12.5
ALSPAC
CBC_hispanic
CBC_white
CHAMACOS
EARLI
GECKO
GEN3G
GENR
GOYA
IOW
MEDALL
MOBA1
MOBA2
MOBA3
NEST
NFCS
NHBCS
RICHS
VIVA
Omitted study
Leave−one−out plot for probe:
cg23080818 [RBMS1]
Meta−analysis −log10(P−value)
0
3
6
9
12
ALSPAC
CBC_hispanic
CBC_white
CHAMACOS
EARLI
GECKO
GEN3G
GENR
GOYA
MEDALL
MOBA1
MOBA2
MOBA3
NEST
NFCS
NHBCS
RICHS
VIVA
Omitted study
Leave−one−out plot for probe:
cg25213362 [TMPRSS12]
Meta−analysis −log10(P−value)
0.0
2.5
5.0
7.5
10.0
ALSPAC
CBC_hispanic
CBC_white
CHAMACOS
EARLI
GECKO
GEN3G
GENR
GOYA
IOW
MEDALL
MOBA1
MOBA2
MOBA3
NEST
NFCS
NHBCS
RICHS
VIVA
Omitted study
Leave−one−out plot for probe:
cg17782974 [TRIM8]
Meta−analysis −log10(P−value)
0
3
6
9
ALSPAC
CBC_hispanic
CBC_white
CHAMACOS
EARLI
GECKO
GEN3G
GENR
GOYA
IOW
MEDALL
MOBA1
MOBA2
MOBA3
NEST
NFCS
NHBCS
RICHS
VIVA
Omitted study
Leave−one−out plot for probe:
cg05086444 [VIPR2]
Meta−analysis −log10(P−value)
0.0
2.5
5.0
7.5
10.0
ALSPAC
CBC_hispanic
CBC_white
CHAMACOS
EARLI
GECKO
GEN3G
GENR
GOYA
MEDALL
MOBA1
MOBA2
MOBA3
NEST
NFCS
NHBCS
RICHS
VIVA
Omitted study

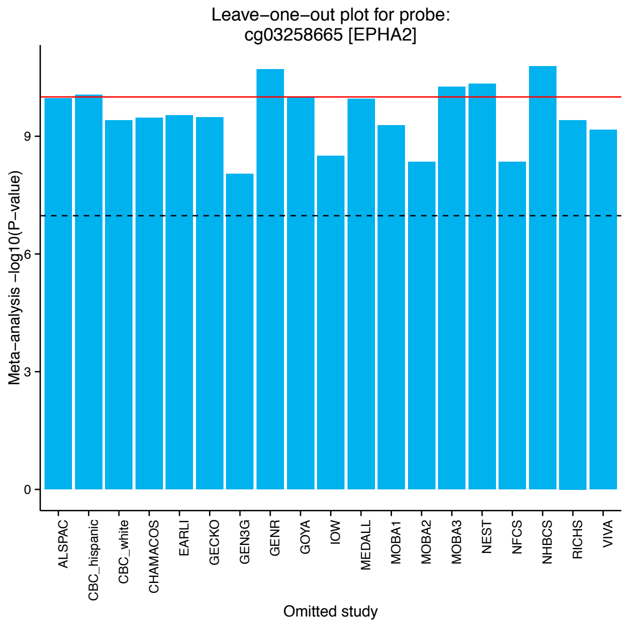
Leave−one−out plot for probe:
cg03258665 [EPHA2]
Meta−analysis −log10(P−value)
0
3
6
9
ALSPAC
CBC_hispanic
CBC_white
CHAMACOS
EARLI
GECKO
GEN3G
GENR
GOYA
IOW
MEDALL
MOBA1
MOBA2
MOBA3
NEST
NFCS
NHBCS
RICHS
VIVA
Omitted study

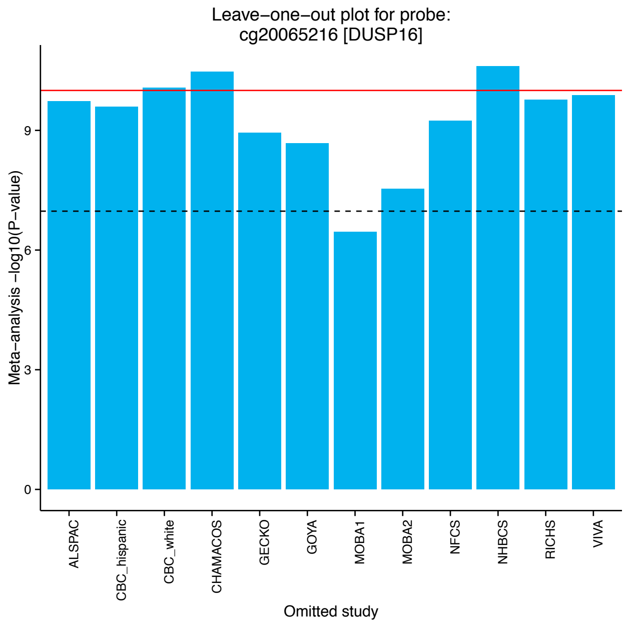
Leave−one−out plot for probe:
cg20065216 [DUSP16]
Meta−analysis −log10(P−value)
0
3
6
9
ALSPAC
CBC_hispanic
CBC_white
CHAMACOS
GECKO
GOYA
MOBA1
MOBA2
NFCS
NHBCS
RICHS
VIVA
Omitted study

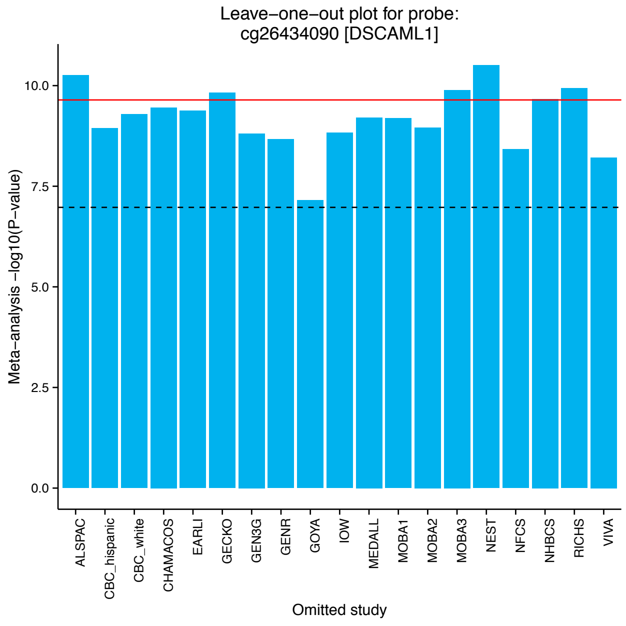
Leave−one−out plot for probe:
cg26434090 [DSCAML1]
Meta−analysis −log10(P−value)
0.0
2.5
5.0
7.5
10.0
ALSPAC
CBC_hispanic
CBC_white
CHAMACOS
EARLI
GECKO
GEN3G
GENR
GOYA
IOW
MEDALL
MOBA1
MOBA2
MOBA3
NEST
NFCS
NHBCS
RICHS
VIVA
Omitted study

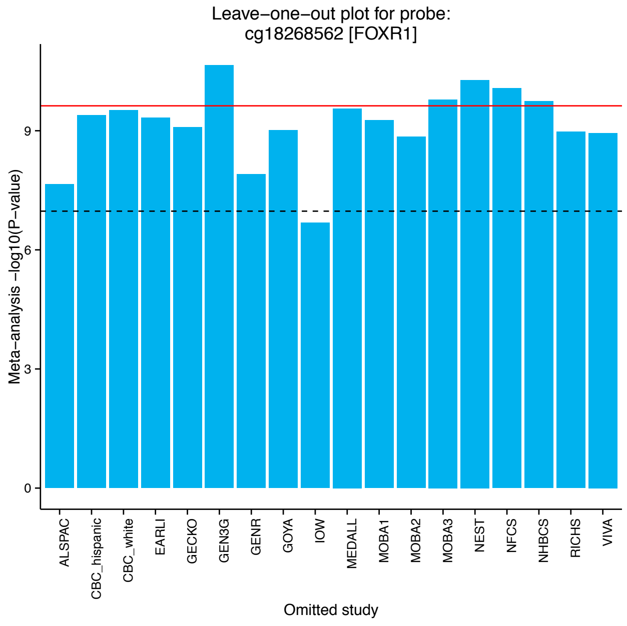
Leave−one−out plot for probe:
cg18268562 [FOXR1]
Meta−analysis −log10(P−value)
0
3
6
9
ALSPAC
CBC_hispanic
CBC_white
EARLI
GECKO
GEN3G
GENR
GOYA
IOW
MEDALL
MOBA1
MOBA2
MOBA3
NEST
NFCS
NHBCS
RICHS
VIVA
Omitted study

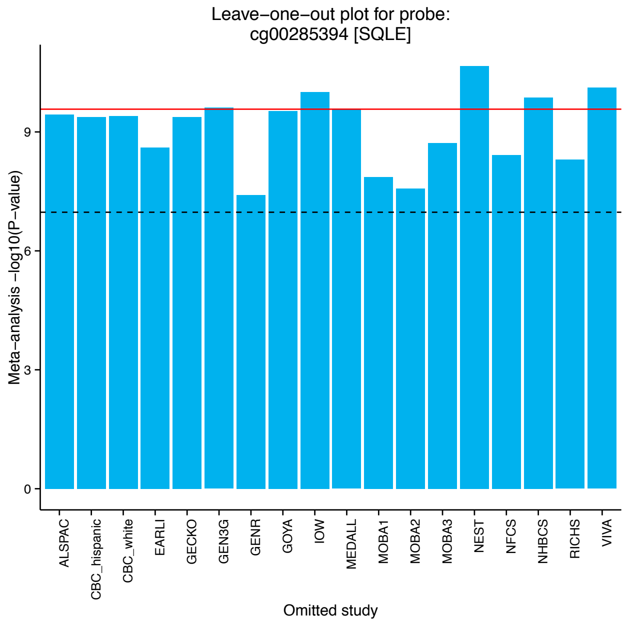
Leave−one−out plot for probe:
cg00285394 [SQLE]
Meta−analysis −log10(P−value)
0
3
6
9
ALSPAC
CBC_hispanic
CBC_white
EARLI
GECKO
GEN3G
GENR
GOYA
IOW
MEDALL
MOBA1
MOBA2
MOBA3
NEST
NFCS
NHBCS
RICHS
VIVA
Omitted study

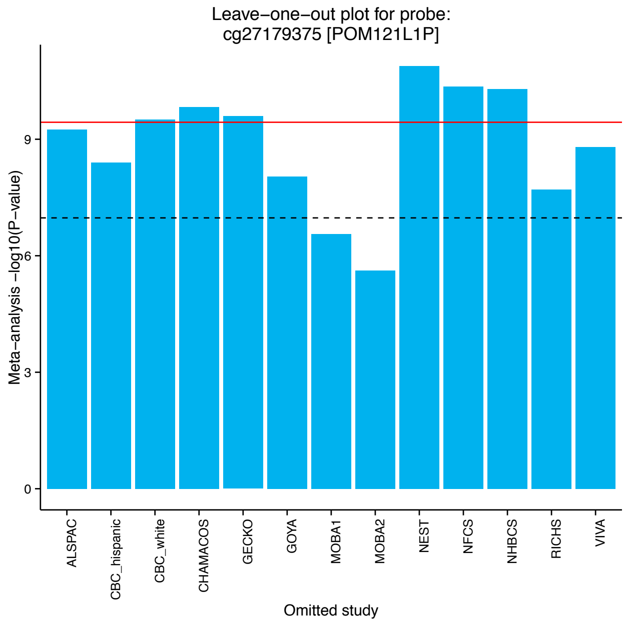
Leave−one−out plot for probe:
cg27179375 [POM121L1P]
Meta−analysis −log10(P−value)
0
3
6
9
ALSPAC
CBC_hispanic
CBC_white
CHAMACOS
GECKO
GOYA
MOBA1
MOBA2
NEST
NFCS
NHBCS
RICHS
VIVA
Omitted study

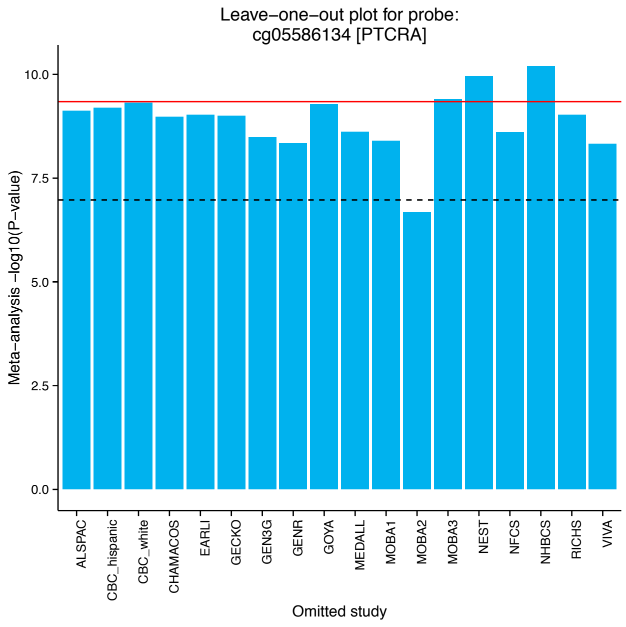
Leave−one−out plot for probe:
cg05586134 [PTCRA]
Meta−analysis −log10(P−value)
0.0
2.5
5.0
7.5
10.0
ALSPAC
CBC_hispanic
CBC_white
CHAMACOS
EARLI
GECKO
GEN3G
GENR
GOYA
MEDALL
MOBA1
MOBA2
MOBA3
NEST
NFCS
NHBCS
RICHS
VIVA
Omitted study

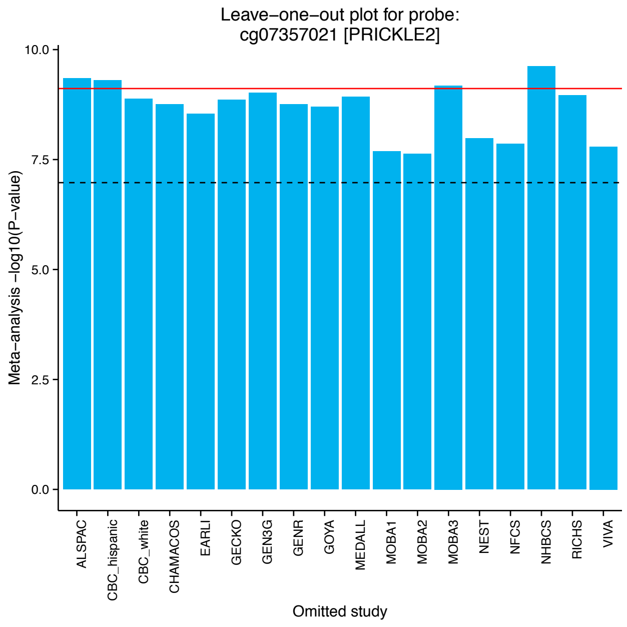
Leave−one−out plot for probe:
cg07357021 [PRICKLE2]
Meta−analysis −log10(P−value)
0.0
2.5
5.0
7.5
10.0
ALSPAC
CBC_hispanic
CBC_white
CHAMACOS
EARLI
GECKO
GEN3G
GENR
GOYA
MEDALL
MOBA1
MOBA2
MOBA3
NEST
NFCS
NHBCS
RICHS
VIVA
Omitted study

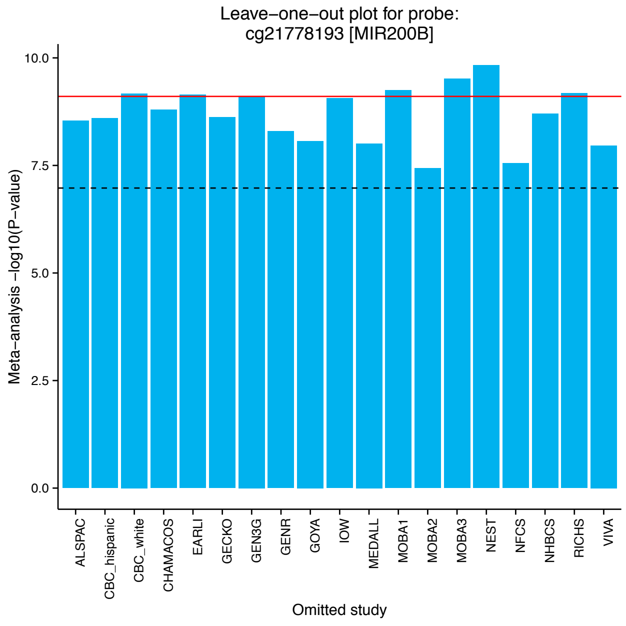
Leave−one−out plot for probe:
cg21778193 [MIR200B]
Meta−analysis −log10(P−value)
0.0
2.5
5.0
7.5
10.0
ALSPAC
CBC_hispanic
CBC_white
CHAMACOS
EARLI
GECKO
GEN3G
GENR
GOYA
IOW
MEDALL
MOBA1
MOBA2
MOBA3
NEST
NFCS
NHBCS
RICHS
VIVA
Omitted study

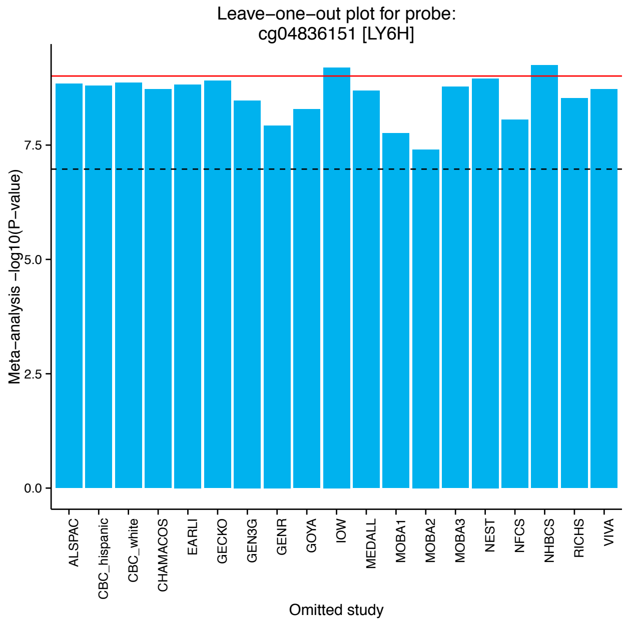
Leave−one−out plot for probe:
cg04836151 [LY6H]
Meta−analysis −log10(P−value)
0.0
2.5
5.0
7.5
ALSPAC
CBC_hispanic
CBC_white
CHAMACOS
EARLI
GECKO
GEN3G
GENR
GOYA
IOW
MEDALL
MOBA1
MOBA2
MOBA3
NEST
NFCS
NHBCS
RICHS
VIVA
Omitted study

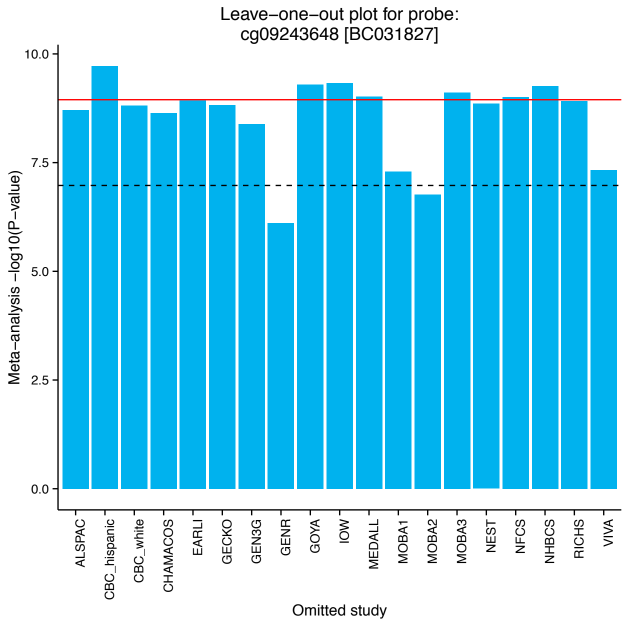
Leave−one−out plot for probe:
cg09243648 [BC031827]
Meta−analysis −log10(P−value)
0.0
2.5
5.0
7.5
10.0
ALSPAC
CBC_hispanic
CBC_white
CHAMACOS
EARLI
GECKO
GEN3G
GENR
GOYA
IOW
MEDALL
MOBA1
MOBA2
MOBA3
NEST
NFCS
NHBCS
RICHS
VIVA
Omitted study

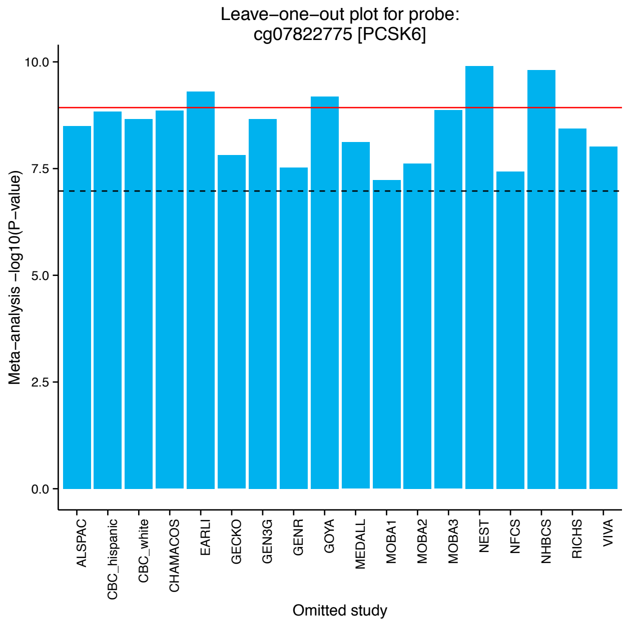
Leave−one−out plot for probe:
cg07822775 [PCSK6]
Meta−analysis −log10(P−value)
0.0
2.5
5.0
7.5
10.0
ALSPAC
CBC_hispanic
CBC_white
CHAMACOS
EARLI
GECKO
GEN3G
GENR
GOYA
MEDALL
MOBA1
MOBA2
MOBA3
NEST
NFCS
NHBCS
RICHS
VIVA
Omitted study

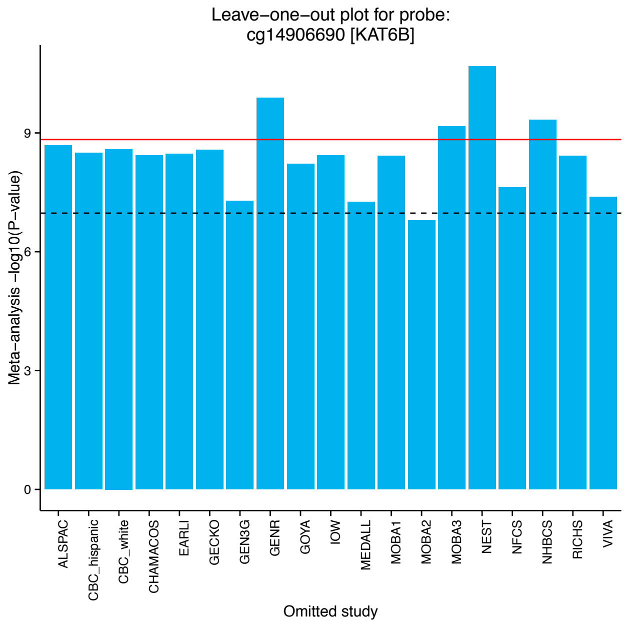
Leave−one−out plot for probe:
cg14906690 [KAT6B]
Meta−analysis −log10(P−value)
0
3
6
9
ALSPAC
CBC_hispanic
CBC_white
CHAMACOS
EARLI
GECKO
GEN3G
GENR
GOYA
IOW
MEDALL
MOBA1
MOBA2
MOBA3
NEST
NFCS
NHBCS
RICHS
VIVA
Omitted study

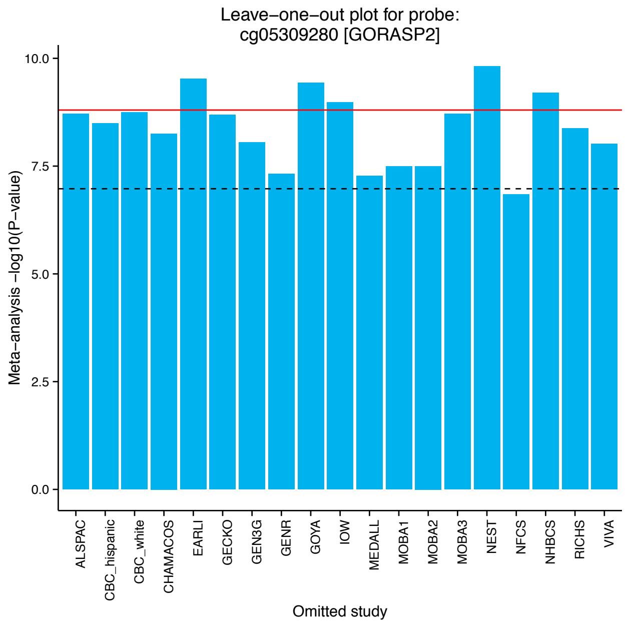
Leave−one−out plot for probe:
cg05309280 [GORASP2]
Meta−analysis −log10(P−value)
0.0
2.5
5.0
7.5
10.0
ALSPAC
CBC_hispanic
CBC_white
CHAMACOS
EARLI
GECKO
GEN3G
GENR
GOYA
IOW
MEDALL
MOBA1
MOBA2
MOBA3
NEST
NFCS
NHBCS
RICHS
VIVA
Omitted study

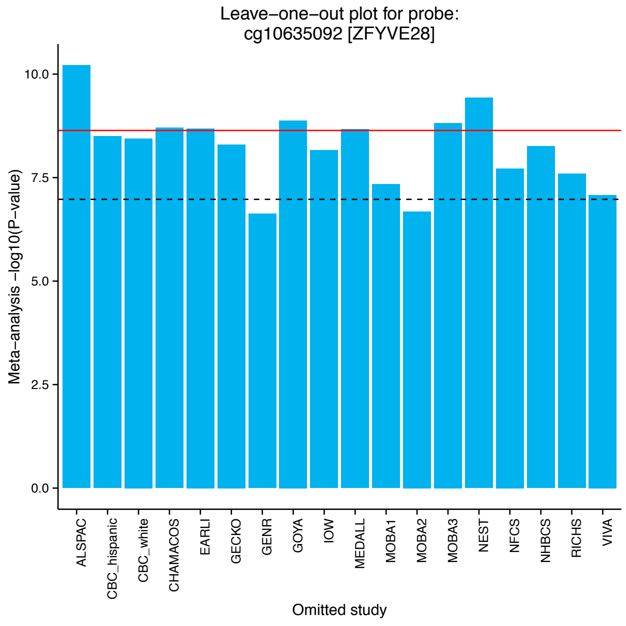
Leave−one−out plot for probe:
cg10635092 [ZFYVE28]
Meta−analysis −log10(P−value)
0.0
2.5
5.0
7.5
10.0
ALSPAC
CBC_hispanic
CBC_white
CHAMACOS
EARLI
GECKO
GENR
GOYA
IOW
MEDALL
MOBA1
MOBA2
MOBA3
NEST
NFCS
NHBCS
RICHS
VIVA
Omitted study

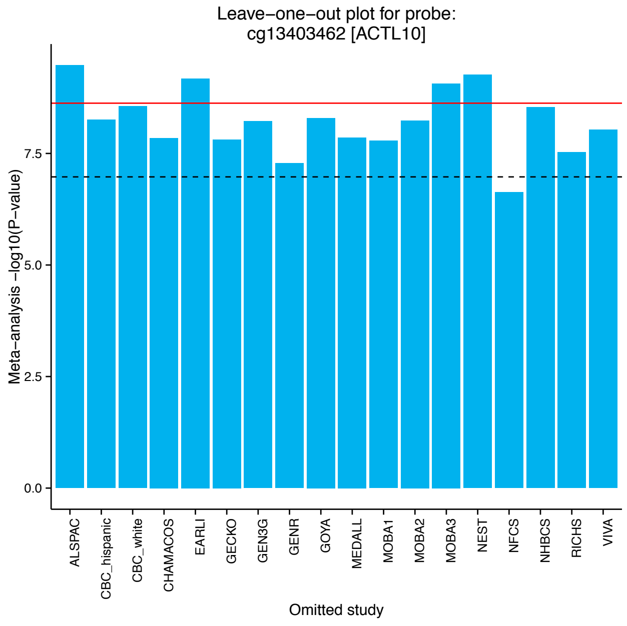
Leave−one−out plot for probe:
cg13403462 [ACTL10]
Meta−analysis −log10(P−value)
0.0
2.5
5.0
7.5
ALSPAC
CBC_hispanic
CBC_white
CHAMACOS
EARLI
GECKO
GEN3G
GENR
GOYA
MEDALL
MOBA1
MOBA2
MOBA3
NEST
NFCS
NHBCS
RICHS
VIVA
Omitted study

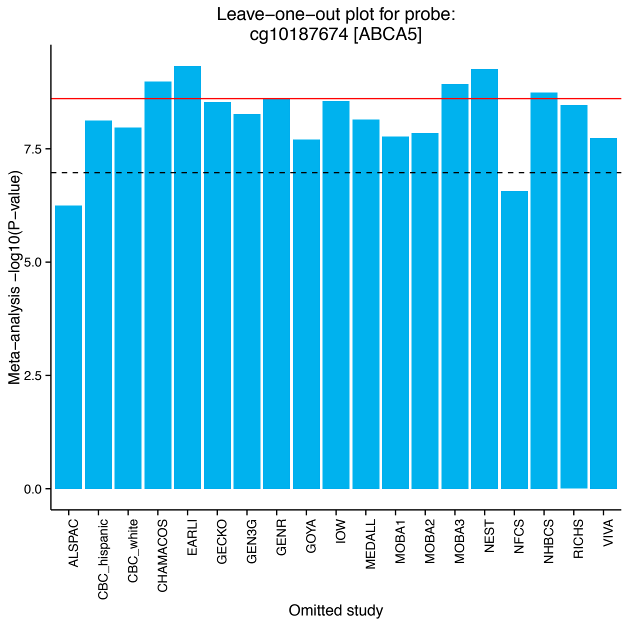
Leave−one−out plot for probe:
cg10187674 [ABCA5]
Meta−analysis −log10(P−value)
0.0
2.5
5.0
7.5
ALSPAC
CBC_hispanic
CBC_white
CHAMACOS
EARLI
GECKO
GEN3G
GENR
GOYA
IOW
MEDALL
MOBA1
MOBA2
MOBA3
NEST
NFCS
NHBCS
RICHS
VIVA
Omitted study

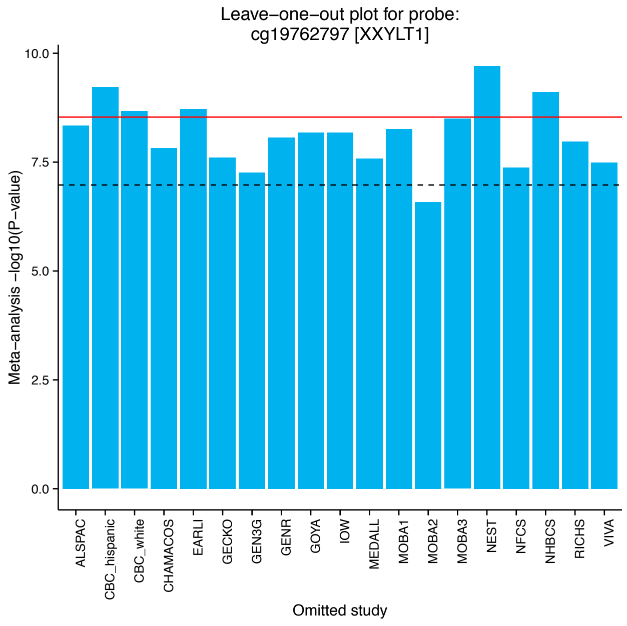
Leave−one−out plot for probe:
cg19762797 [XXYLT1]
Meta−analysis −log10(P−value)
0.0
2.5
5.0
7.5
10.0
ALSPAC
CBC_hispanic
CBC_white
CHAMACOS
EARLI
GECKO
GEN3G
GENR
GOYA
IOW
MEDALL
MOBA1
MOBA2
MOBA3
NEST
NFCS
NHBCS
RICHS
VIVA
Omitted study

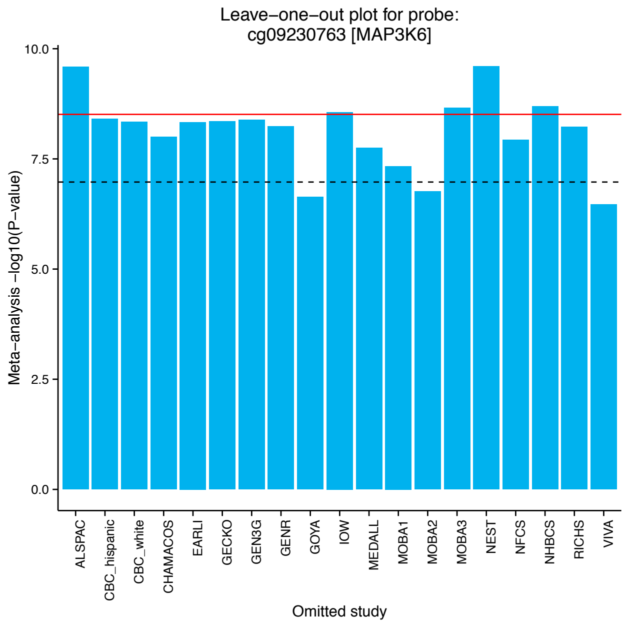
Leave−one−out plot for probe:
cg09230763 [MAP3K6]
Meta−analysis −log10(P−value)
10.0
7.5
5.0
2.5
0.0
ALSPAC
CBC_hispanic
CBC_white
CHAMACOS
EARLI
GECKO
GEN3G
GENR
GOYA
IOW
MEDALL
MOBA1
MOBA2
MOBA3
NEST
NFCS
NHBCS
RICHS
VIVA
Omitted study

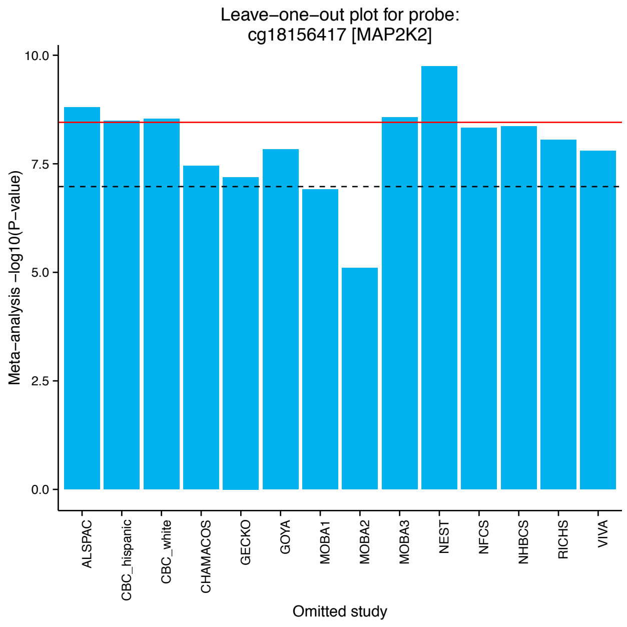
Leave−one−out plot for probe:
cg18156417 [MAP2K2]
Meta−analysis −log10(P−value)
10.0
7.5
5.0
2.5
0.0
ALSPAC
CBC_hispanic
CBC_white
CHAMACOS
GECKO
GOYA
MOBA1
MOBA2
MOBA3
NEST
NFCS
NHBCS
RICHS
VIVA
Omitted study

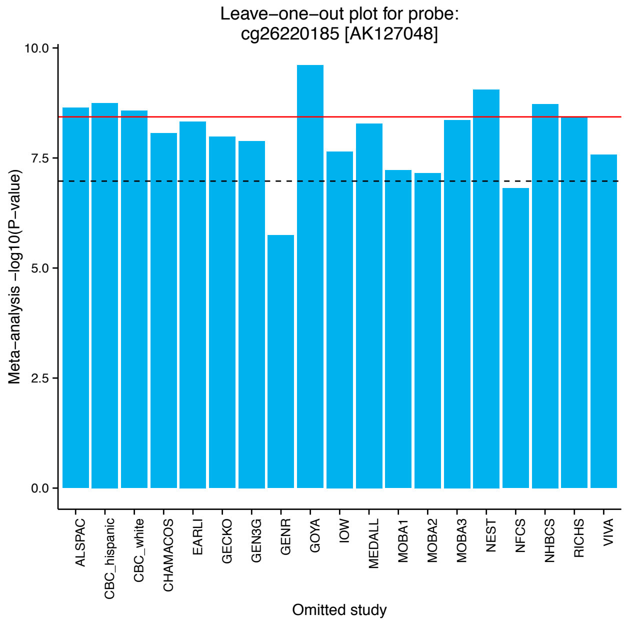
Leave−one−out plot for probe:
cg26220185 [AK127048]
Meta−analysis −log10(P−value)
10.0
7.5
5.0
2.5
0.0
ALSPAC
CBC_hispanic
CBC_white
CHAMACOS
EARLI
GECKO
GEN3G
GENR
GOYA
IOW
MEDALL
MOBA1
MOBA2
MOBA3
NEST
NFCS
NHBCS
RICHS
VIVA
Omitted study

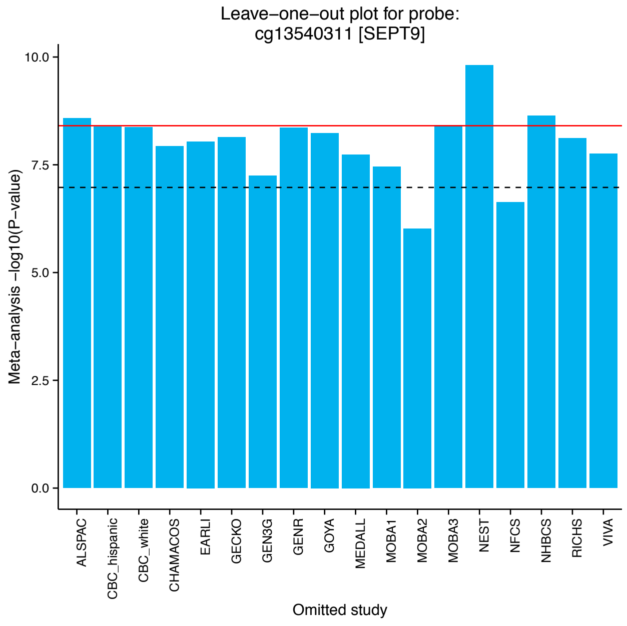
Leave−one−out plot for probe:
cg13540311 [SEPT9]
Meta−analysis −log10(P−value)
10.0
7.5
5.0
2.5
0.0
ALSPAC
CBC_hispanic
CBC_white
CHAMACOS
EARLI
GECKO
GEN3G
GENR
GOYA
MEDALL
MOBA1
MOBA2
MOBA3
NEST
NFCS
NHBCS
RICHS
VIVA
Omitted study

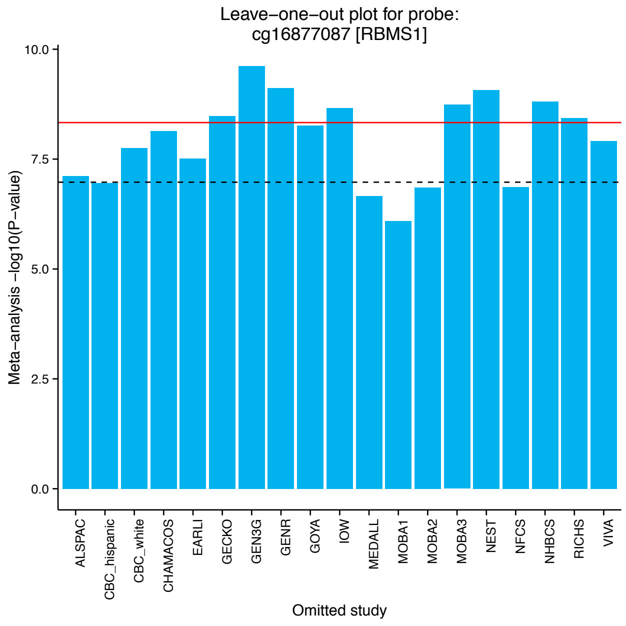
Leave−one−out plot for probe:
cg16877087 [RBMS1]
Meta−analysis −log10(P−value)
10.0
7.5
5.0
2.5
0.0
ALSPAC
CBC_hispanic
CBC_white
CHAMACOS
EARLI
GECKO
GEN3G
GENR
GOYA
IOW
MEDALL
MOBA1
MOBA2
MOBA3
NEST
NFCS
NHBCS
RICHS
VIVA
Omitted study

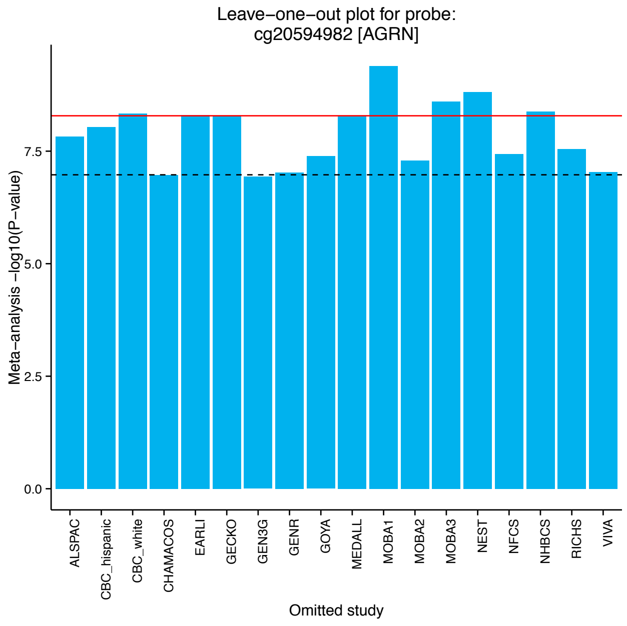
Leave−one−out plot for probe:
cg20594982 [AGRN]
Meta−analysis −log10(P−value)
7.5
5.0
2.5
0.0
ALSPAC
CBC_hispanic
CBC_white
CHAMACOS
EARLI
GECKO
GEN3G
GENR
GOYA
MEDALL
MOBA1
MOBA2
MOBA3
NEST
NFCS
NHBCS
RICHS
VIVA
Omitted study

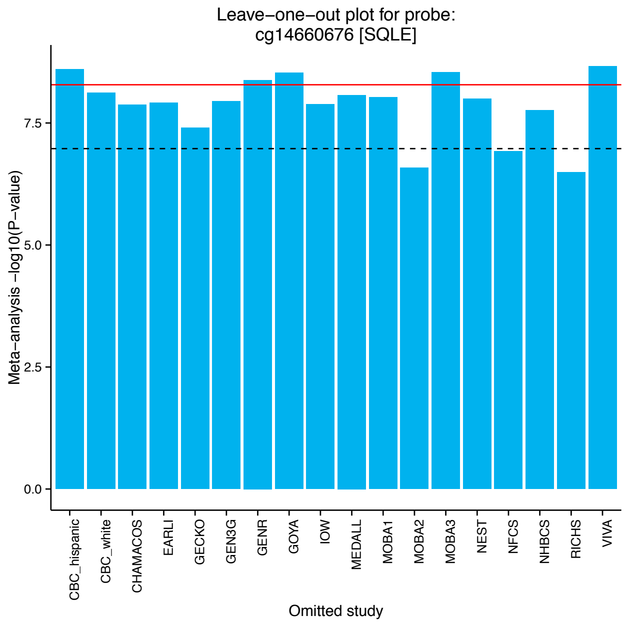
Leave−one−out plot for probe:
cg14660676 [SQLE]
Meta−analysis −log10(P−value)
0.0
2.5
5.0
7.5
CBC_hispanic
CBC_white
CHAMACOS
EARLI
GECKO
GEN3G
GENR
GOYA
IOW
MEDALL
MOBA1
MOBA2
MOBA3
NEST
NFCS
NHBCS
RICHS
VIVA
Omitted study

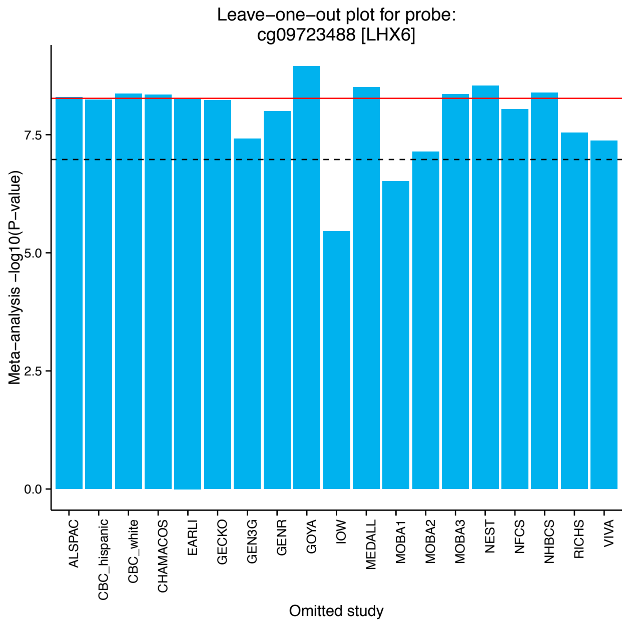
Leave−one−out plot for probe:
cg09723488 [LHX6]
Meta−analysis −log10(P−value)
0.0
2.5
5.0
7.5
ALSPAC
CBC_hispanic
CBC_white
CHAMACOS
EARLI
GECKO
GEN3G
GENR
GOYA
IOW
MEDALL
MOBA1
MOBA2
MOBA3
NEST
NFCS
NHBCS
RICHS
VIVA
Omitted study

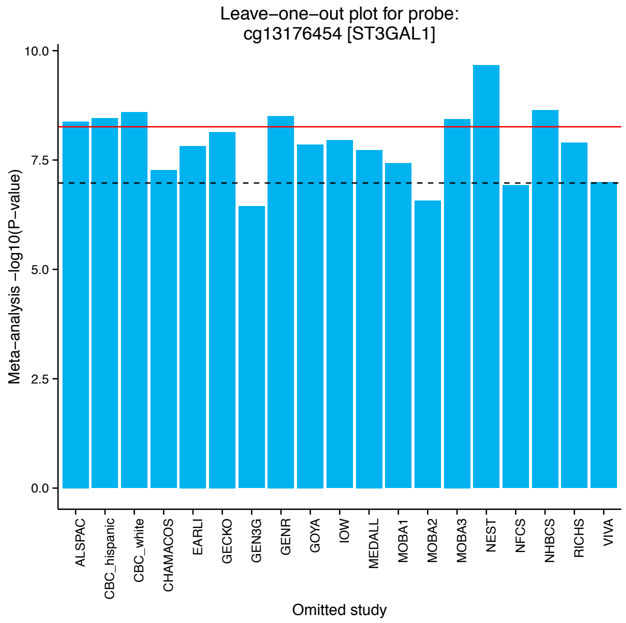
Leave−one−out plot for probe:
cg13176454 [ST3GAL1]
Meta−analysis −log10(P−value)
0.0
2.5
5.0
7.5
10.0
ALSPAC
CBC_hispanic
CBC_white
CHAMACOS
EARLI
GECKO
GEN3G
GENR
GOYA
IOW
MEDALL
MOBA1
MOBA2
MOBA3
NEST
NFCS
NHBCS
RICHS
VIVA
Omitted study

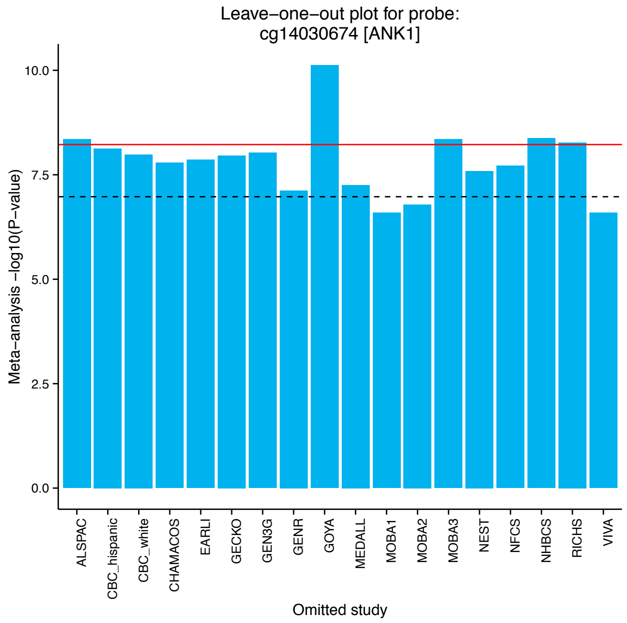
Leave−one−out plot for probe:
cg14030674 [ANK1]
Meta−analysis −log10(P−value)
0.0
2.5
5.0
7.5
10.0
ALSPAC
CBC_hispanic
CBC_white
CHAMACOS
EARLI
GECKO
GEN3G
GENR
GOYA
MEDALL
MOBA1
MOBA2
MOBA3
NEST
NFCS
NHBCS
RICHS
VIVA
Omitted study

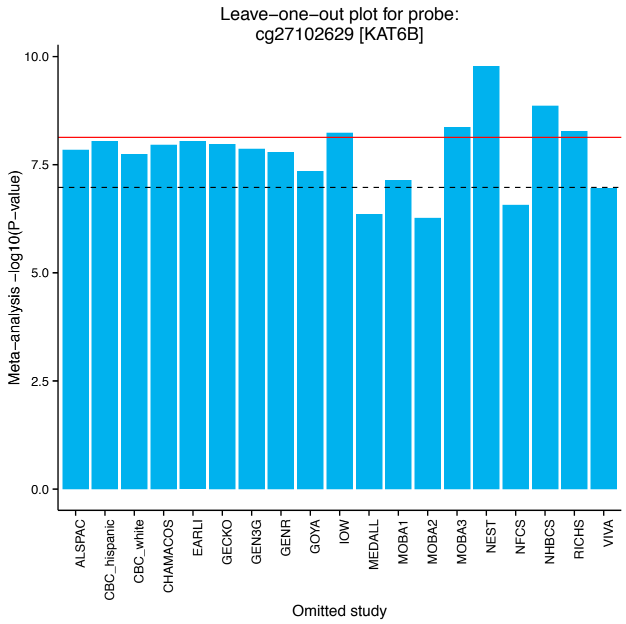
Leave−one−out plot for probe:
cg27102629 [KAT6B]
Meta−analysis −log10(P−value)
0.0
2.5
5.0
7.5
10.0
ALSPAC
CBC_hispanic
CBC_white
CHAMACOS
EARLI
GECKO
GEN3G
GENR
GOYA
IOW
MEDALL
MOBA1
MOBA2
MOBA3
NEST
NFCS
NHBCS
RICHS
VIVA
Omitted study

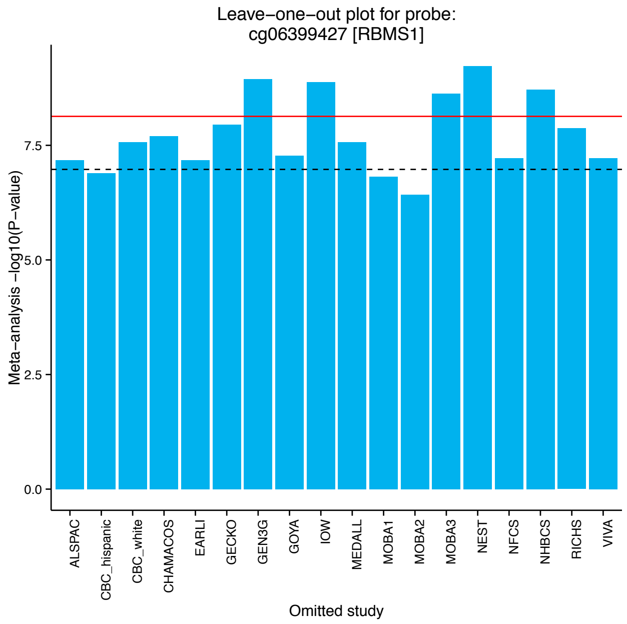
Leave−one−out plot for probe:
cg06399427 [RBMS1]
Meta−analysis −log10(P−value)
0.0
2.5
5.0
7.5
ALSPAC
CBC_hispanic
CBC_white
CHAMACOS
EARLI
GECKO
GEN3G
GOYA
IOW
MEDALL
MOBA1
MOBA2
MOBA3
NEST
NFCS
NHBCS
RICHS
VIVA
Omitted study

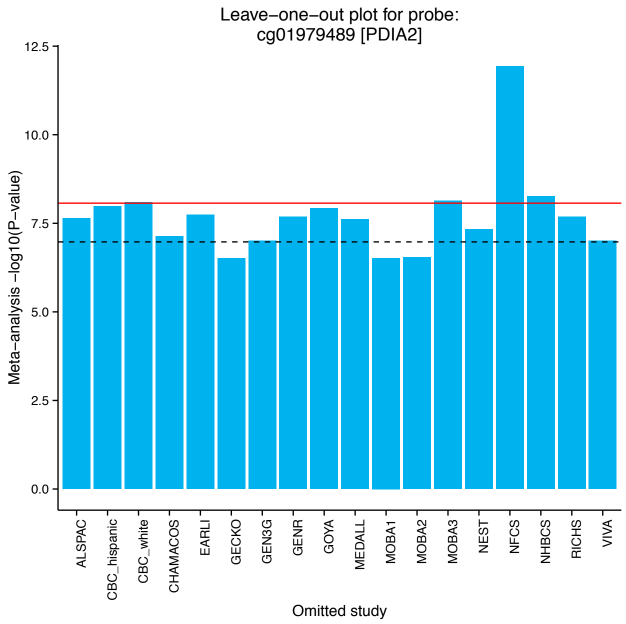
Leave−one−out plot for probe:
cg01979489 [PDIA2]
Meta−analysis −log10(P−value)
0.0
2.5
5.0
7.5
10.0
12.5
ALSPAC
CBC_hispanic
CBC_white
CHAMACOS
EARLI
GECKO
GEN3G
GENR
GOYA
MEDALL
MOBA1
MOBA2
MOBA3
NEST
NFCS
NHBCS
RICHS
VIVA
Omitted study

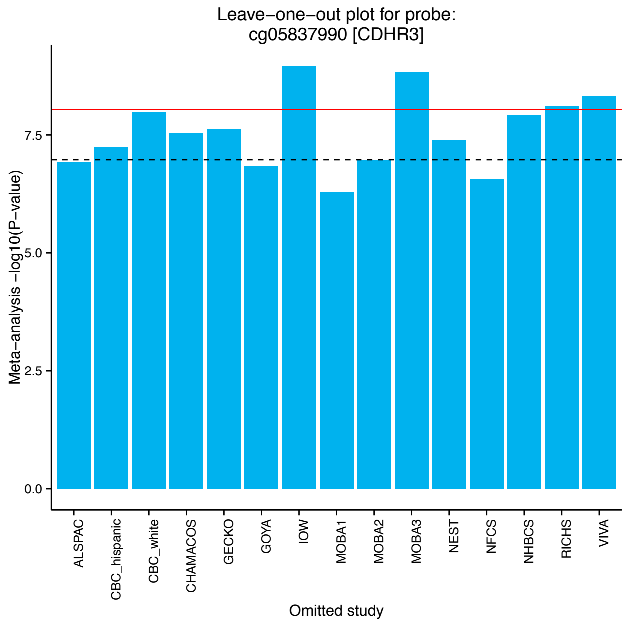
Leave−one−out plot for probe:
cg05837990 [CDHR3]
Meta−analysis −log10(P−value)
0.0
2.5
5.0
7.5
ALSPAC
CBC_hispanic
CBC_white
CHAMACOS
GECKO
GOYA
IOW
MOBA1
MOBA2
MOBA3
NEST
NFCS
NHBCS
RICHS
VIVA
Omitted study

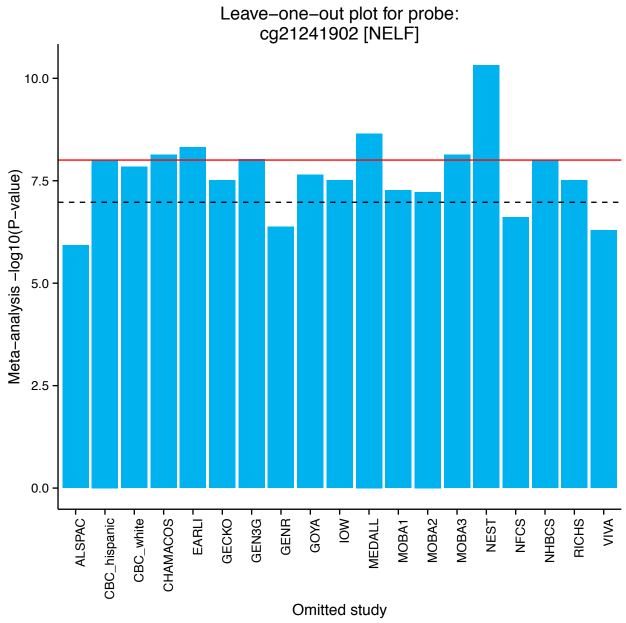
Leave−one−out plot for probe:
cg21241902 [NELF]
Meta−analysis −log10(P−value)
0.0
2.5
5.0
7.5
10.0
ALSPAC
CBC_hispanic
CBC_white
CHAMACOS
EARLI
GECKO
GEN3G
GENR
GOYA
IOW
MEDALL
MOBA1
MOBA2
MOBA3
NEST
NFCS
NHBCS
RICHS
VIVA
Omitted study

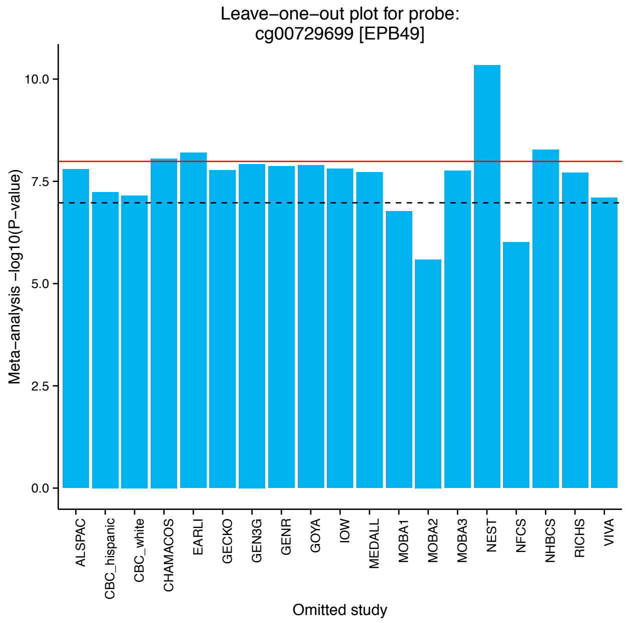
Leave−one−out plot for probe:
cg00729699 [EPB49]
Meta−analysis −log10(P−value)
0.0
2.5
5.0
7.5
10.0
ALSPAC
CBC_hispanic
CBC_white
CHAMACOS
EARLI
GECKO
GEN3G
GENR
GOYA
IOW
MEDALL
MOBA1
MOBA2
MOBA3
NEST
NFCS
NHBCS
RICHS
VIVA
Omitted study

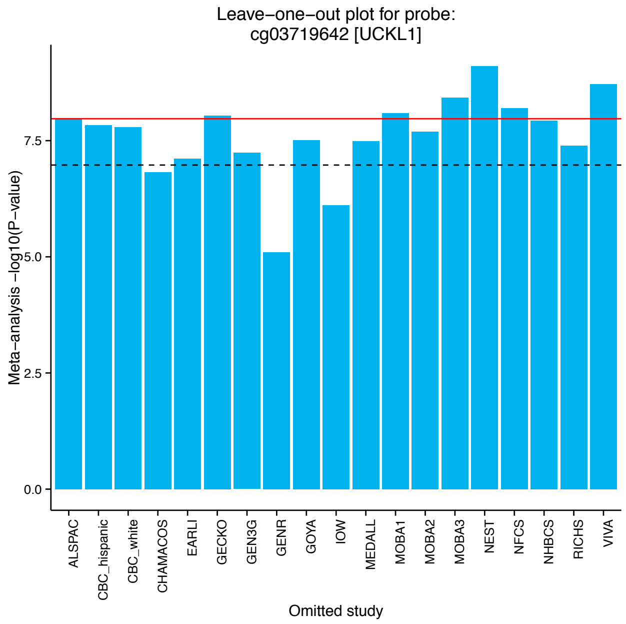
Leave−one−out plot for probe:
cg03719642 [UCKL1]
Meta−analysis −log10(P−value)
0.0
2.5
5.0
7.5
ALSPAC
CBC_hispanic
CBC_white
CHAMACOS
EARLI
GECKO
GEN3G
GENR
GOYA
IOW
MEDALL
MOBA1
MOBA2
MOBA3
NEST
NFCS
NHBCS
RICHS
VIVA
Omitted study

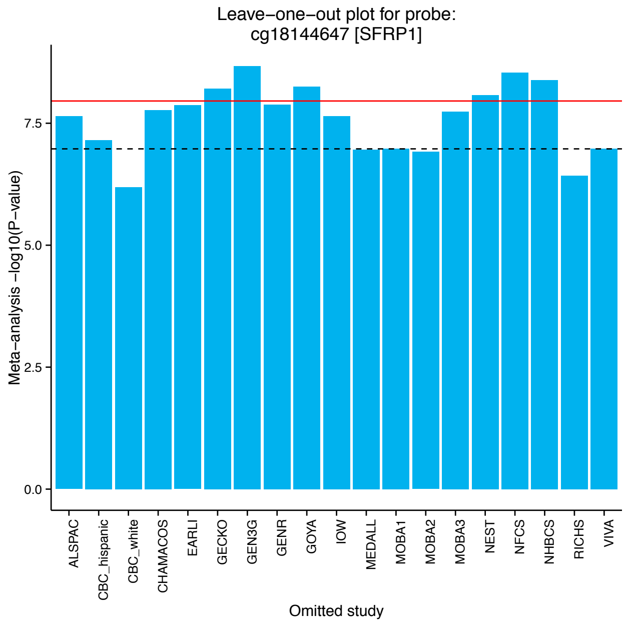
Leave−one−out plot for probe:
cg18144647 [SFRP1]
Meta−analysis −log10(P−value)
0.0
2.5
5.0
7.5
ALSPAC
CBC_hispanic
CBC_white
CHAMACOS
EARLI
GECKO
GEN3G
GENR
GOYA
IOW
MEDALL
MOBA1
MOBA2
MOBA3
NEST
NFCS
NHBCS
RICHS
VIVA
Omitted study

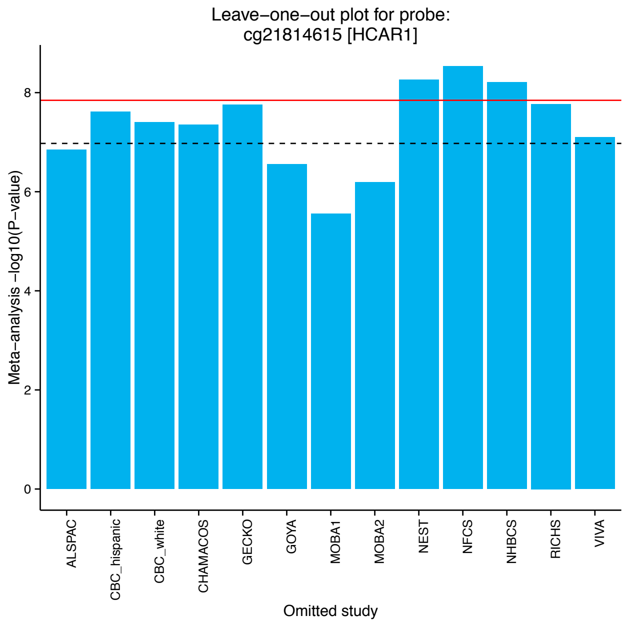
Leave−one−out plot for probe:
cg21814615 [HCAR1]
Meta−analysis −log10(P−value)
0
2
4
6
8
ALSPAC
CBC_hispanic
CBC_white
CHAMACOS
GECKO
GOYA
MOBA1
MOBA2
NEST
NFCS
NHBCS
RICHS
VIVA
Omitted study

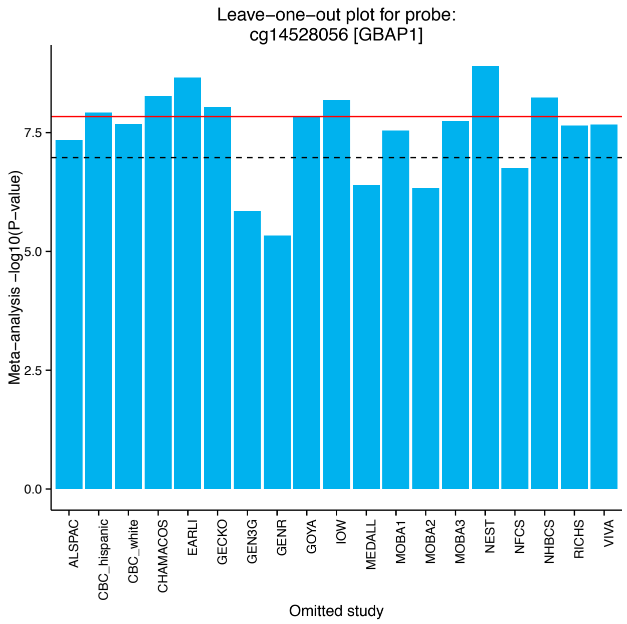
Leave−one−out plot for probe:
cg14528056 [GBAP1]
Meta−analysis −log10(P−value)
0.0
2.5
5.0
7.5
ALSPAC
CBC_hispanic
CBC_white
CHAMACOS
EARLI
GECKO
GEN3G
GENR
GOYA
IOW
MEDALL
MOBA1
MOBA2
MOBA3
NEST
NFCS
NHBCS
RICHS
VIVA
Omitted study

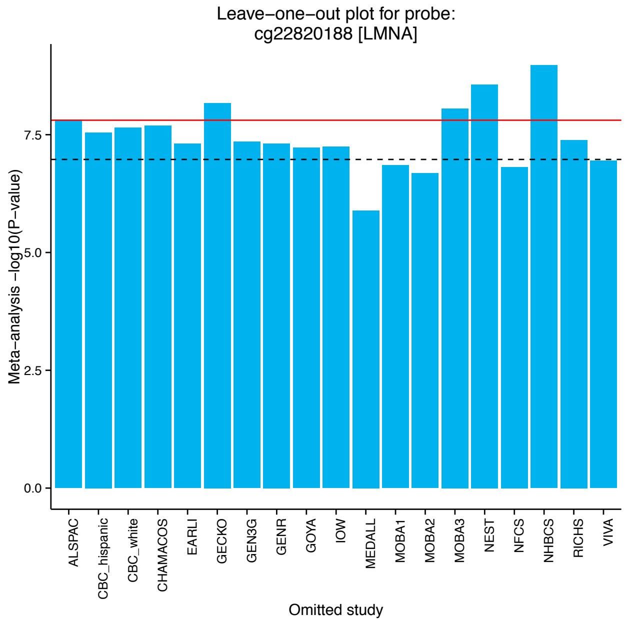
Leave−one−out plot for probe:
cg22820188 [LMNA]
Meta−analysis −log10(P−value)
0.0
2.5
5.0
7.5
ALSPAC
CBC_hispanic
CBC_white
CHAMACOS
EARLI
GECKO
GEN3G
GENR
GOYA
IOW
MEDALL
MOBA1
MOBA2
MOBA3
NEST
NFCS
NHBCS
RICHS
VIVA
Omitted study

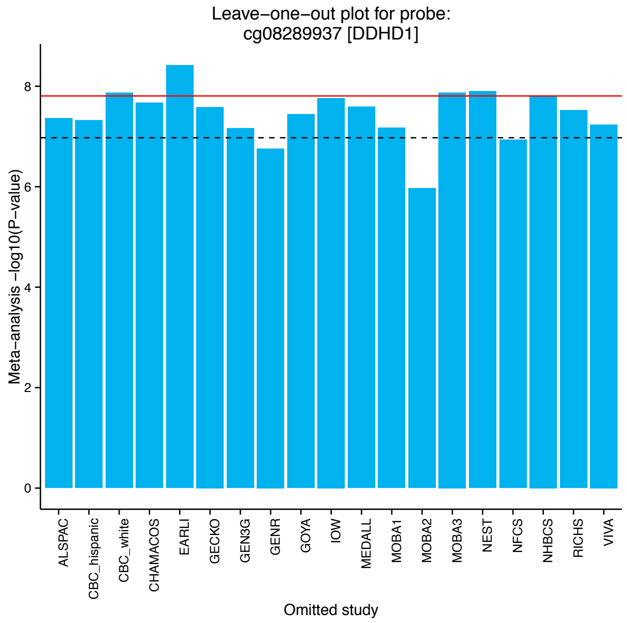
Leave−one−out plot for probe:
cg08289937 [DDHD1]
Meta−analysis −log10(P−value)
0
2
4
6
8
ALSPAC
CBC_hispanic
CBC_white
CHAMACOS
EARLI
GECKO
GEN3G
GENR
GOYA
IOW
MEDALL
MOBA1
MOBA2
MOBA3
NEST
NFCS
NHBCS
RICHS
VIVA
Omitted study

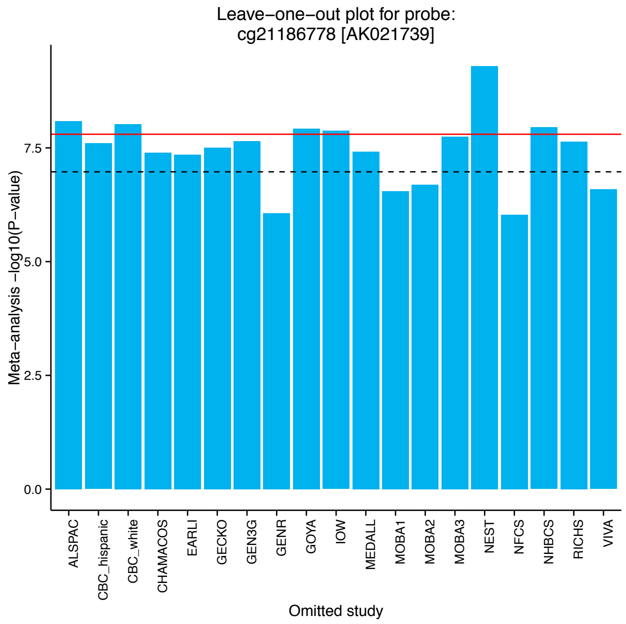
Leave−one−out plot for probe:
cg21186778 [AK021739]
Meta−analysis −log10(P−value)
0.0
2.5
5.0
7.5
ALSPAC
CBC_hispanic
CBC_white
CHAMACOS
EARLI
GECKO
GEN3G
GENR
GOYA
IOW
MEDALL
MOBA1
MOBA2
MOBA3
NEST
NFCS
NHBCS
RICHS
VIVA
Omitted study

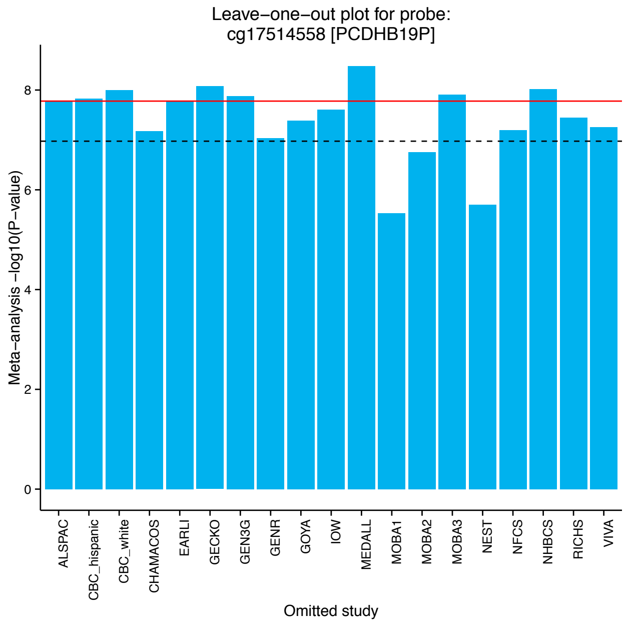
Leave−one−out plot for probe:
cg17514558 [PCDHB19P]
Meta−analysis −log10(P−value)
0
2
4
6
8
ALSPAC
CBC_hispanic
CBC_white
CHAMACOS
EARLI
GECKO
GEN3G
GENR
GOYA
IOW
MEDALL
MOBA1
MOBA2
MOBA3
NEST
NFCS
NHBCS
RICHS
VIVA
Omitted study

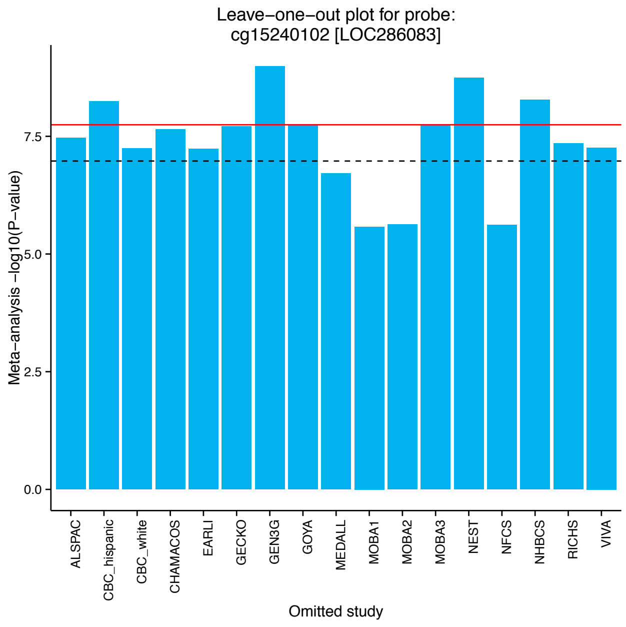
Leave−one−out plot for probe:
cg15240102 [LOC286083]
Meta−analysis −log10(P−value)
0.0
2.5
5.0
7.5
ALSPAC
CBC_hispanic
CBC_white
CHAMACOS
EARLI
GECKO
GEN3G
GOYA
MEDALL
MOBA1
MOBA2
MOBA3
NEST
NFCS
NHBCS
RICHS
VIVA
Omitted study

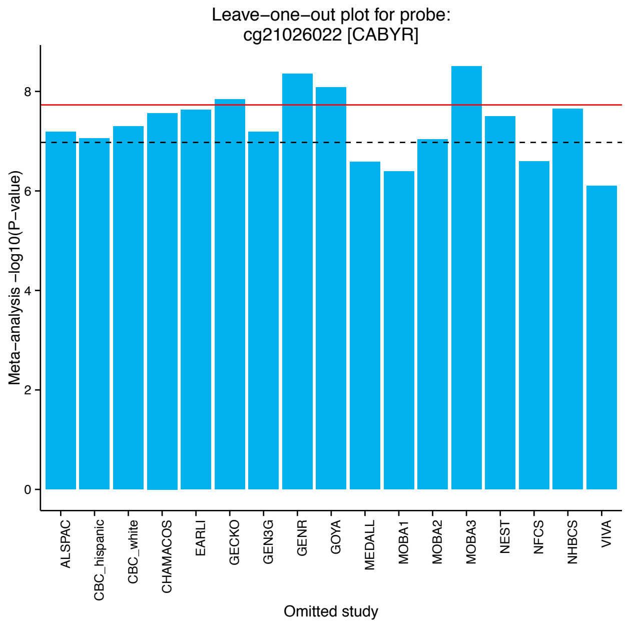
Leave−one−out plot for probe:
cg21026022 [CABYR]
Meta−analysis −log10(P−value)
0
2
4
6
8
ALSPAC
CBC_hispanic
CBC_white
CHAMACOS
EARLI
GECKO
GEN3G
GENR
GOYA
MEDALL
MOBA1
MOBA2
MOBA3
NEST
NFCS
NHBCS
VIVA
Omitted study

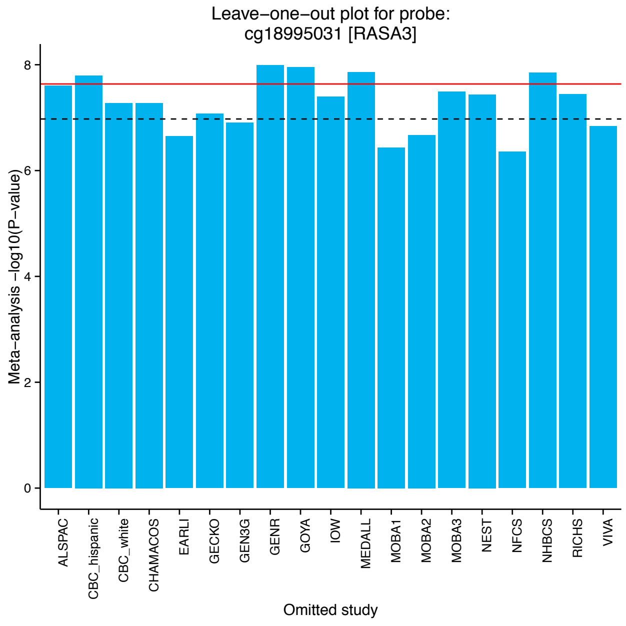
Leave−one−out plot for probe:
cg18995031 [RASA3]
Meta−analysis −log10(P−value)
0
2
4
6
8
ALSPAC
CBC_hispanic
CBC_white
CHAMACOS
EARLI
GECKO
GEN3G
GENR
GOYA
IOW
MEDALL
MOBA1
MOBA2
MOBA3
NEST
NFCS
NHBCS
RICHS
VIVA
Omitted study

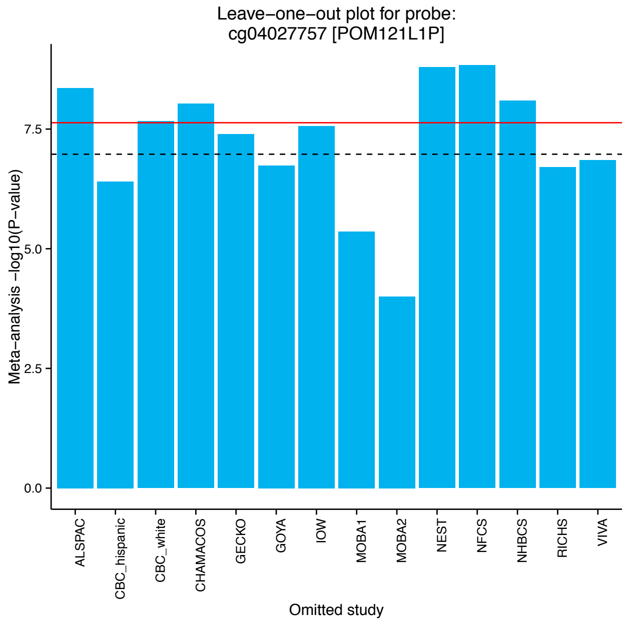
Leave−one−out plot for probe:
cg04027757 [POM121L1P]
Meta−analysis −log10(P−value)
0.0
2.5
5.0
7.5
ALSPAC
CBC_hispanic
CBC_white
CHAMACOS
GECKO
GOYA
IOW
MOBA1
MOBA2
NEST
NFCS
NHBCS
RICHS
VIVA
Omitted study

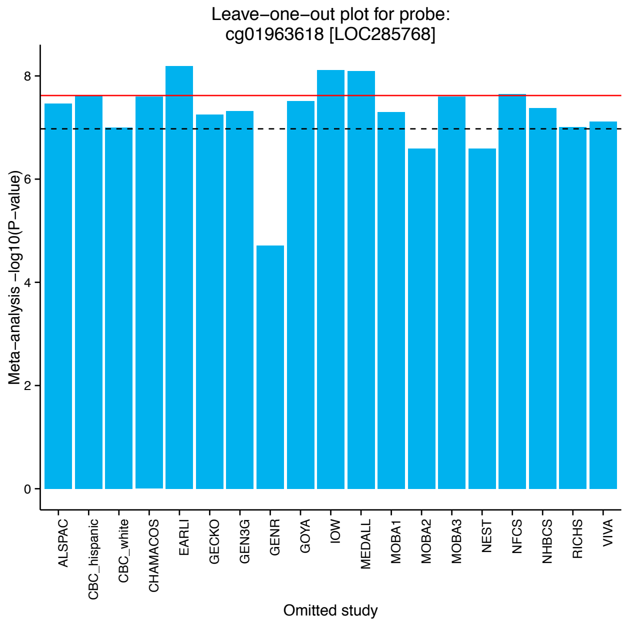
Leave−one−out plot for probe:
cg01963618 [LOC285768]
Meta−analysis −log10(P−value)
0
2
4
6
8
ALSPAC
CBC_hispanic
CBC_white
CHAMACOS
EARLI
GECKO
GEN3G
GENR
GOYA
IOW
MEDALL
MOBA1
MOBA2
MOBA3
NEST
NFCS
NHBCS
RICHS
VIVA
Omitted study

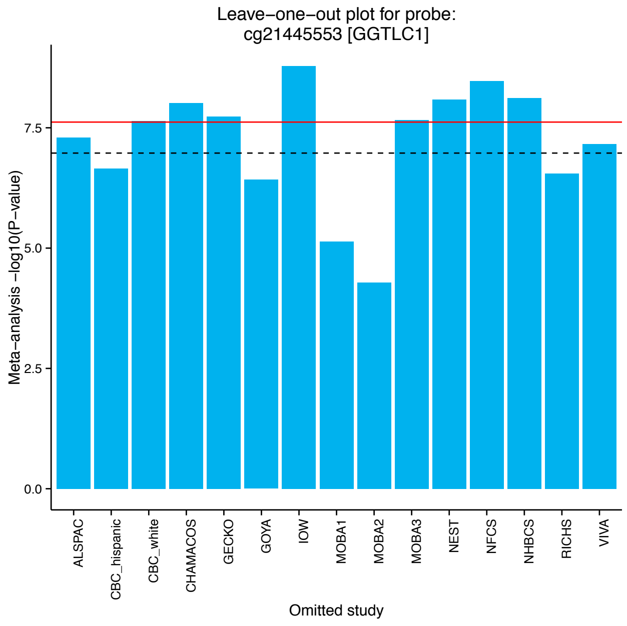
Leave−one−out plot for probe:
cg21445553 [GGTLC1]
Meta−analysis −log10(P−value)
0.0
2.5
5.0
7.5
ALSPAC
CBC_hispanic
CBC_white
CHAMACOS
GECKO
GOYA
IOW
MOBA1
MOBA2
MOBA3
NEST
NFCS
NHBCS
RICHS
VIVA
Omitted study

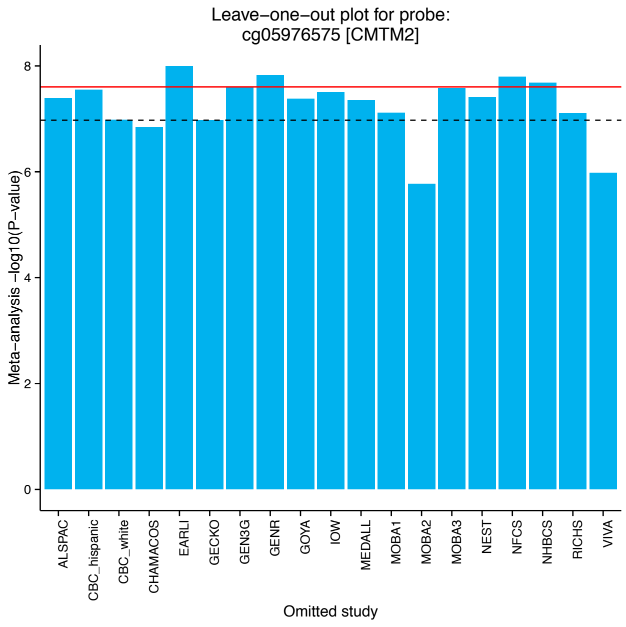
Leave−one−out plot for probe:
cg05976575 [CMTM2]
Meta−analysis −log10(P−value)
0
2
4
6
8
ALSPAC
CBC_hispanic
CBC_white
CHAMACOS
EARLI
GECKO
GEN3G
GENR
GOYA
IOW
MEDALL
MOBA1
MOBA2
MOBA3
NEST
NFCS
NHBCS
RICHS
VIVA
Omitted study

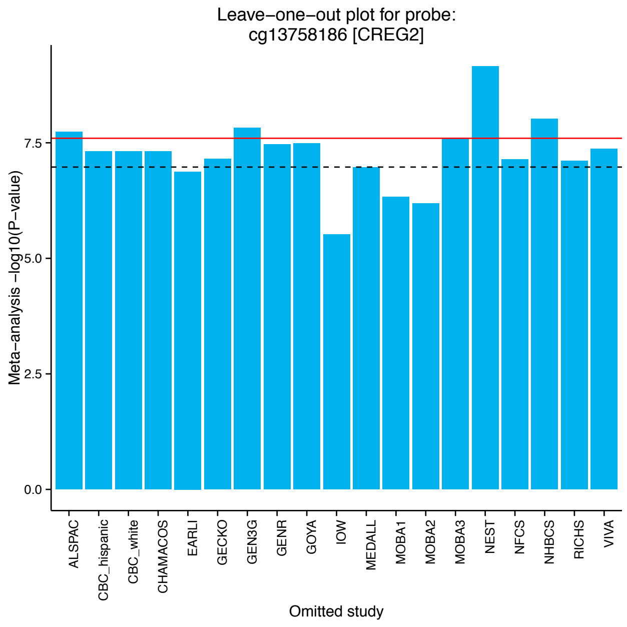
Leave−one−out plot for probe:
cg13758186 [CREG2]
Meta−analysis −log10(P−value)
0.0
2.5
5.0
7.5
ALSPAC
CBC_hispanic
CBC_white
CHAMACOS
EARLI
GECKO
GEN3G
GENR
GOYA
IOW
MEDALL
MOBA1
MOBA2
MOBA3
NEST
NFCS
NHBCS
RICHS
VIVA
Omitted study

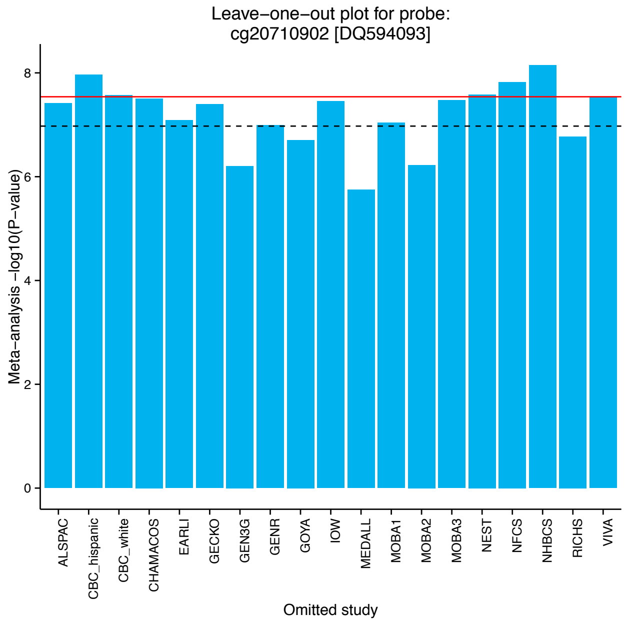
Leave−one−out plot for probe:
cg20710902 [DQ594093]
Meta−analysis −log10(P−value)
0
2
4
6
8
ALSPAC
CBC_hispanic
CBC_white
CHAMACOS
EARLI
GECKO
GEN3G
GENR
GOYA
IOW
MEDALL
MOBA1
MOBA2
MOBA3
NEST
NFCS
NHBCS
RICHS
VIVA
Omitted study

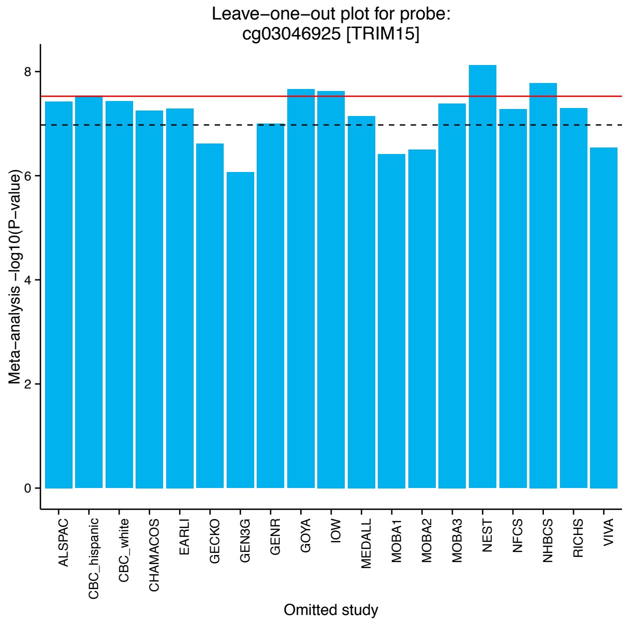
Leave−one−out plot for probe:
cg03046925 [TRIM15]
Meta−analysis −log10(P−value)
0
2
4
6
8
ALSPAC
CBC_hispanic
CBC_white
CHAMACOS
EARLI
GECKO
GEN3G
GENR
GOYA
IOW
MEDALL
MOBA1
MOBA2
MOBA3
NEST
NFCS
NHBCS
RICHS
VIVA
Omitted study

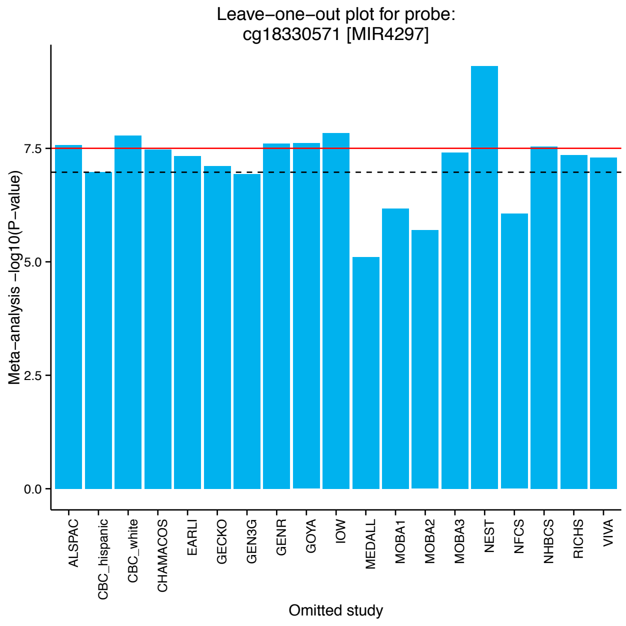
Leave−one−out plot for probe:
cg18330571 [MIR4297]
Meta−analysis −log10(P−value)
0.0
2.5
5.0
7.5
ALSPAC
CBC_hispanic
CBC_white
CHAMACOS
EARLI
GECKO
GEN3G
GENR
GOYA
IOW
MEDALL
MOBA1
MOBA2
MOBA3
NEST
NFCS
NHBCS
RICHS
VIVA
Omitted study

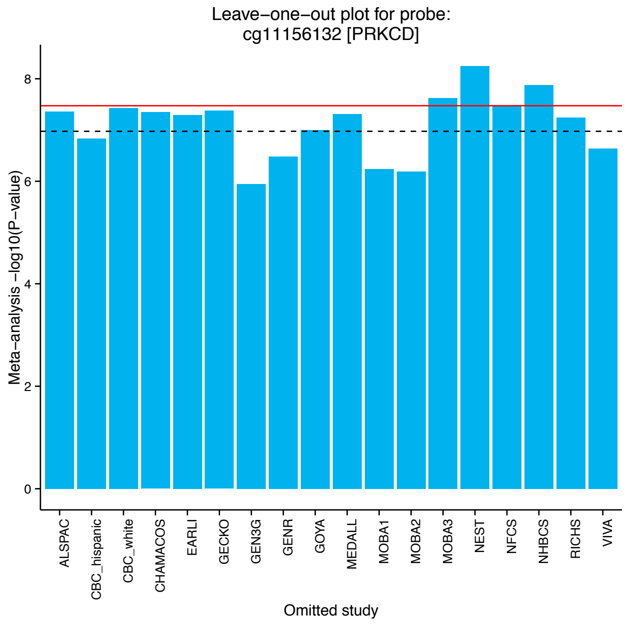
Leave−one−out plot for probe:
cg11156132 [PRKCD]
Meta−analysis −log10(P−value)
0
2
4
6
8
ALSPAC
CBC_hispanic
CBC_white
CHAMACOS
EARLI
GECKO
GEN3G
GENR
GOYA
MEDALL
MOBA1
MOBA2
MOBA3
NEST
NFCS
NHBCS
RICHS
VIVA
Omitted study

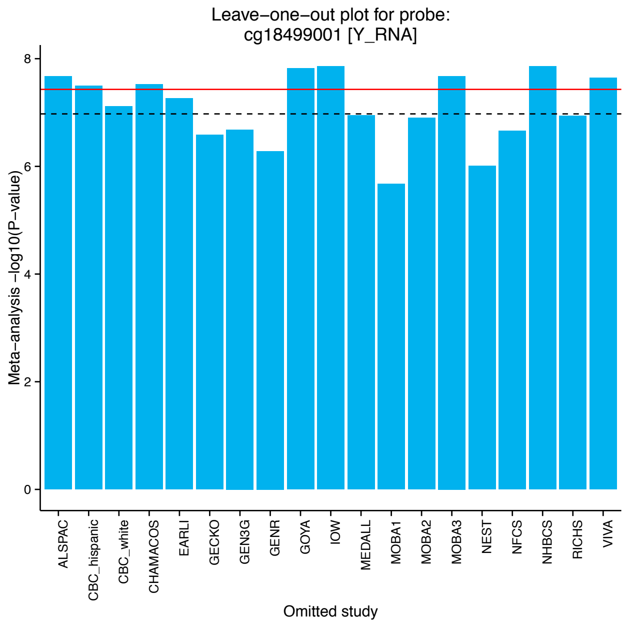
Leave−one−out plot for probe:
cg18499001 [Y_RNA]
Meta−analysis −log10(P−value)
0
2
4
6
8
ALSPAC
CBC_hispanic
CBC_white
CHAMACOS
EARLI
GECKO
GEN3G
GENR
GOYA
IOW
MEDALL
MOBA1
MOBA2
MOBA3
NEST
NFCS
NHBCS
RICHS
VIVA
Omitted study

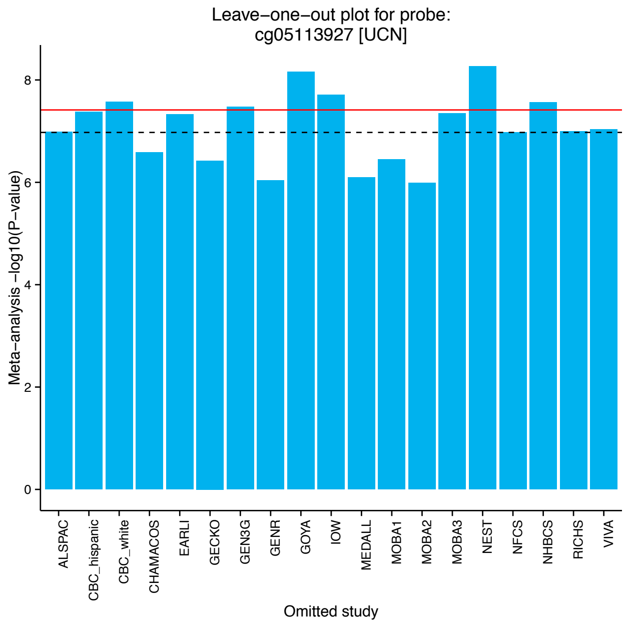
Leave−one−out plot for probe:
cg05113927 [UCN]
Meta−analysis −log10(P−value)
0
2
4
6
8
ALSPAC
CBC_hispanic
CBC_white
CHAMACOS
EARLI
GECKO
GEN3G
GENR
GOYA
IOW
MEDALL
MOBA1
MOBA2
MOBA3
NEST
NFCS
NHBCS
RICHS
VIVA
Omitted study

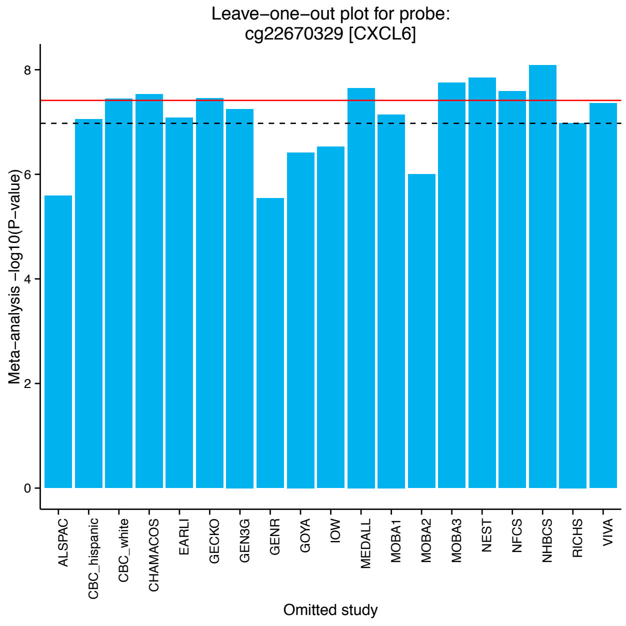
Leave−one−out plot for probe:
cg22670329 [CXCL6]
Meta−analysis −log10(P−value)
0
2
4
6
8
ALSPAC
CBC_hispanic
CBC_white
CHAMACOS
EARLI
GECKO
GEN3G
GENR
GOYA
IOW
MEDALL
MOBA1
MOBA2
MOBA3
NEST
NFCS
NHBCS
RICHS
VIVA
Omitted study

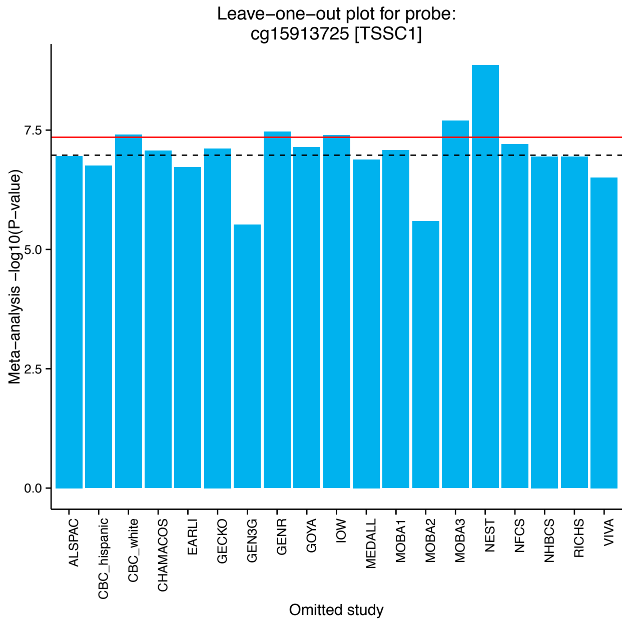
Leave−one−out plot for probe:
cg15913725 [TSSC1]
Meta−analysis −log10(P−value)
0.0
2.5
5.0
7.5
ALSPAC
CBC_hispanic
CBC_white
CHAMACOS
EARLI
GECKO
GEN3G
GENR
GOYA
IOW
MEDALL
MOBA1
MOBA2
MOBA3
NEST
NFCS
NHBCS
RICHS
VIVA
Omitted study

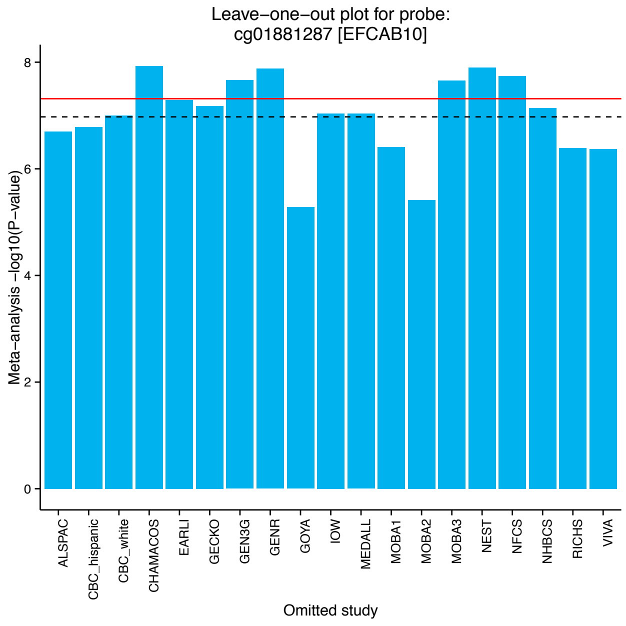
Leave−one−out plot for probe:
cg01881287 [EFCAB10]
Meta−analysis −log10(P−value)
0
2
4
6
8
ALSPAC
CBC_hispanic
CBC_white
CHAMACOS
EARLI
GECKO
GEN3G
GENR
GOYA
IOW
MEDALL
MOBA1
MOBA2
MOBA3
NEST
NFCS
NHBCS
RICHS
VIVA
Omitted study

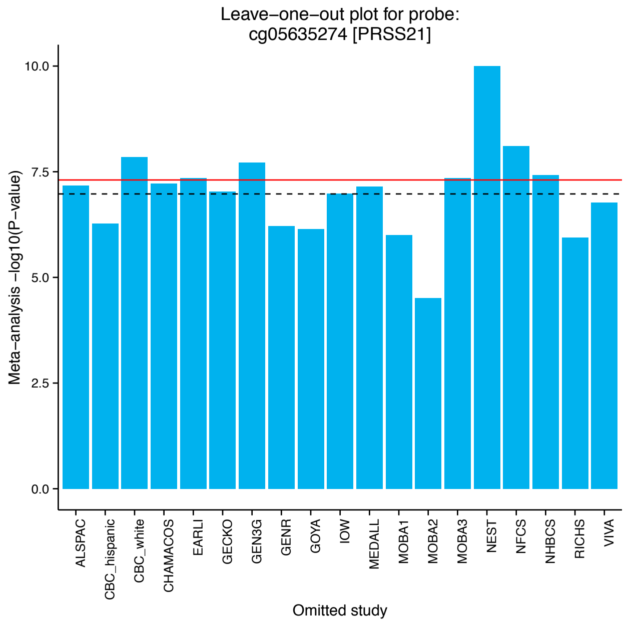
Leave−one−out plot for probe:
cg05635274 [PRSS21]
Meta−analysis −log10(P−value)
0.0
2.5
5.0
7.5
10.0
ALSPAC
CBC_hispanic
CBC_white
CHAMACOS
EARLI
GECKO
GEN3G
GENR
GOYA
IOW
MEDALL
MOBA1
MOBA2
MOBA3
NEST
NFCS
NHBCS
RICHS
VIVA
Omitted study

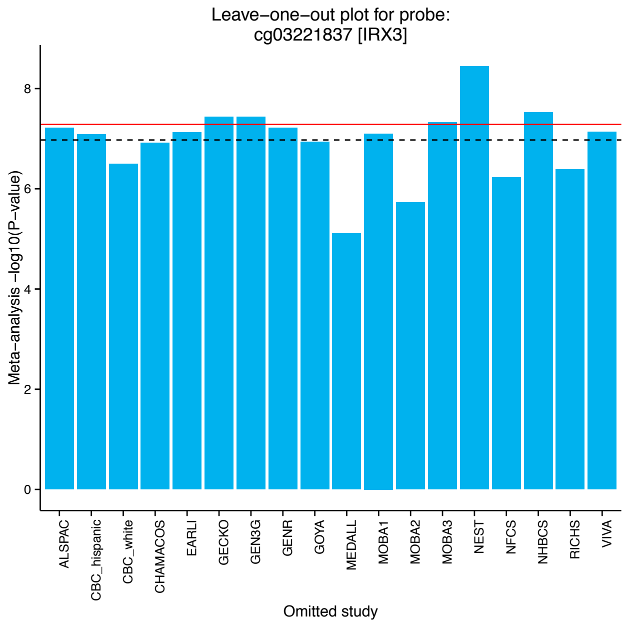
Leave−one−out plot for probe:
cg03221837 [IRX3]
Meta−analysis −log10(P−value)
0
2
4
6
8
ALSPAC
CBC_hispanic
CBC_white
CHAMACOS
EARLI
GECKO
GEN3G
GENR
GOYA
MEDALL
MOBA1
MOBA2
MOBA3
NEST
NFCS
NHBCS
RICHS
VIVA
Omitted study

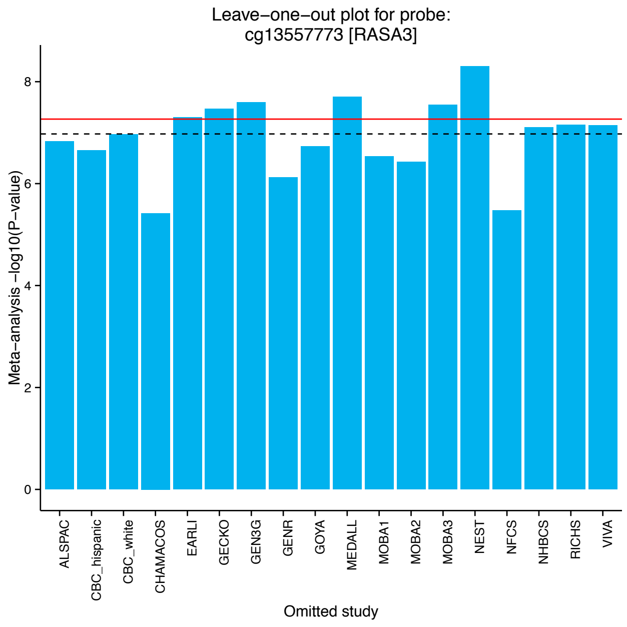
Leave−one−out plot for probe:
cg13557773 [RASA3]
Meta−analysis −log10(P−value)
0
2
4
6
8
ALSPAC
CBC_hispanic
CBC_white
CHAMACOS
EARLI
GECKO
GEN3G
GENR
GOYA
MEDALL
MOBA1
MOBA2
MOBA3
NEST
NFCS
NHBCS
RICHS
VIVA
Omitted study

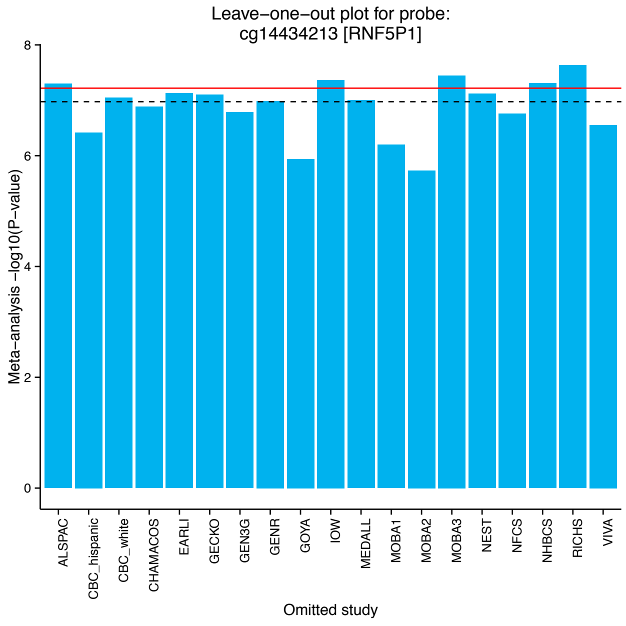
Leave−one−out plot for probe:
cg14434213 [RNF5P1]
Meta−analysis −log10(P−value)
0
2
4
6
8
ALSPAC
CBC_hispanic
CBC_white
CHAMACOS
EARLI
GECKO
GEN3G
GENR
GOYA
IOW
MEDALL
MOBA1
MOBA2
MOBA3
NEST
NFCS
NHBCS
RICHS
VIVA
Omitted study

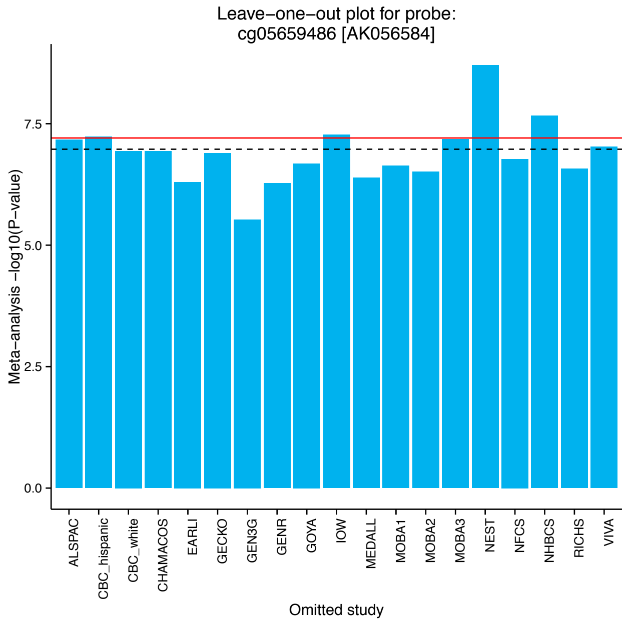
Leave−one−out plot for probe:
cg05659486 [AK056584]
Meta−analysis −log10(P−value)
0.0
2.5
5.0
7.5
ALSPAC
CBC_hispanic
CBC_white
CHAMACOS
EARLI
GECKO
GEN3G
GENR
GOYA
IOW
MEDALL
MOBA1
MOBA2
MOBA3
NEST
NFCS
NHBCS
RICHS
VIVA
Omitted study

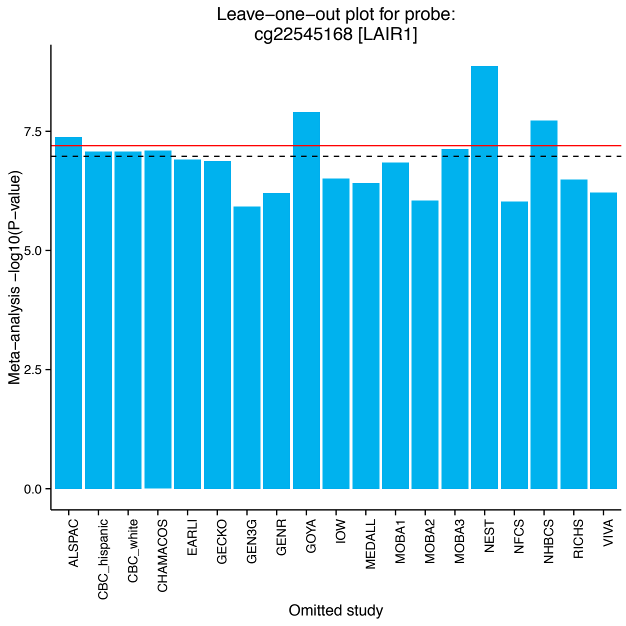
Leave−one−out plot for probe:
cg22545168 [LAIR1]
Meta−analysis −log10(P−value)
0.0
2.5
5.0
7.5
ALSPAC
CBC_hispanic
CBC_white
CHAMACOS
EARLI
GECKO
GEN3G
GENR
GOYA
IOW
MEDALL
MOBA1
MOBA2
MOBA3
NEST
NFCS
NHBCS
RICHS
VIVA
Omitted study

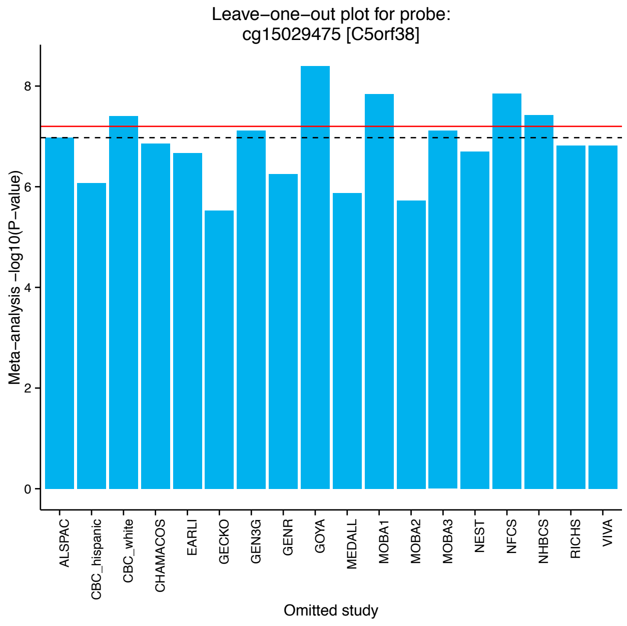
Leave−one−out plot for probe:
cg15029475 [C5orf38]
Meta−analysis −log10(P−value)
0
2
4
6
8
ALSPAC
CBC_hispanic
CBC_white
CHAMACOS
EARLI
GECKO
GEN3G
GENR
GOYA
MEDALL
MOBA1
MOBA2
MOBA3
NEST
NFCS
NHBCS
RICHS
VIVA
Omitted study

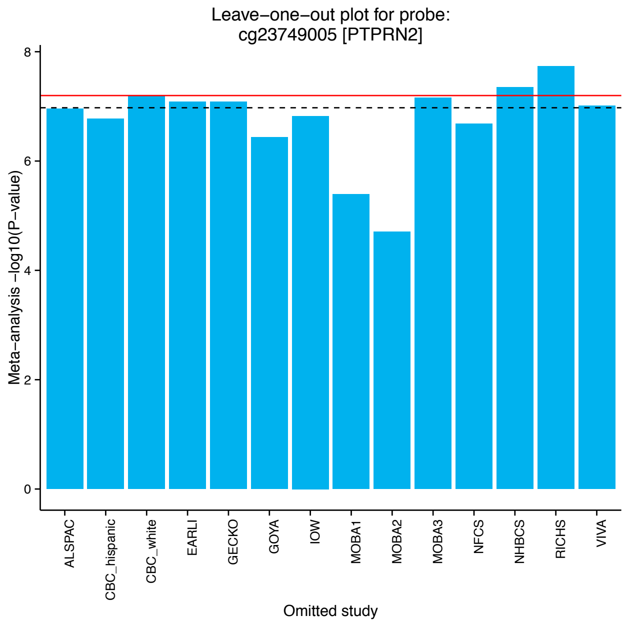
Leave−one−out plot for probe:
cg23749005 [PTPRN2]
Meta−analysis −log10(P−value)
0
2
4
6
8
ALSPAC
CBC_hispanic
CBC_white
EARLI
GECKO
GOYA
IOW
MOBA1
MOBA2
MOBA3
NFCS
NHBCS
RICHS
VIVA
Omitted study

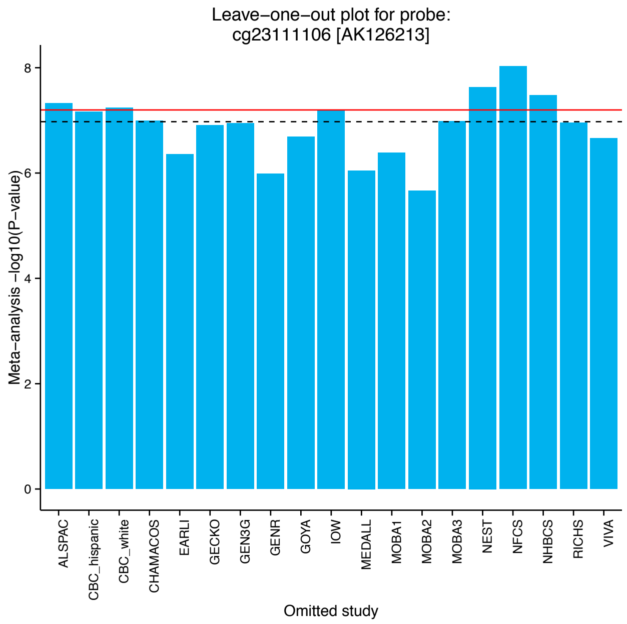
Leave−one−out plot for probe:
cg23111106 [AK126213]
Meta−analysis −log10(P−value)
0
2
4
6
8
ALSPAC
CBC_hispanic
CBC_white
CHAMACOS
EARLI
GECKO
GEN3G
GENR
GOYA
IOW
MEDALL
MOBA1
MOBA2
MOBA3
NEST
NFCS
NHBCS
RICHS
VIVA
Omitted study

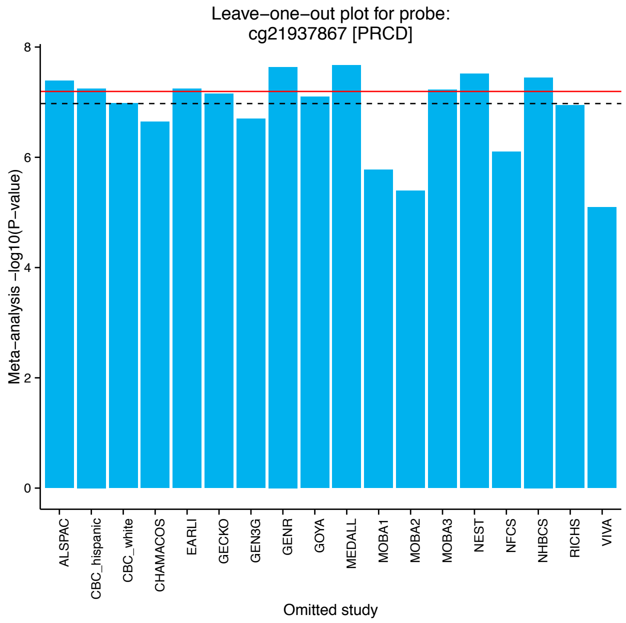
Leave−one−out plot for probe:
cg21937867 [PRCD]
Meta−analysis −log10(P−value)
0
2
4
6
8
ALSPAC
CBC_hispanic
CBC_white
CHAMACOS
EARLI
GECKO
GEN3G
GENR
GOYA
MEDALL
MOBA1
MOBA2
MOBA3
NEST
NFCS
NHBCS
RICHS
VIVA
Omitted study

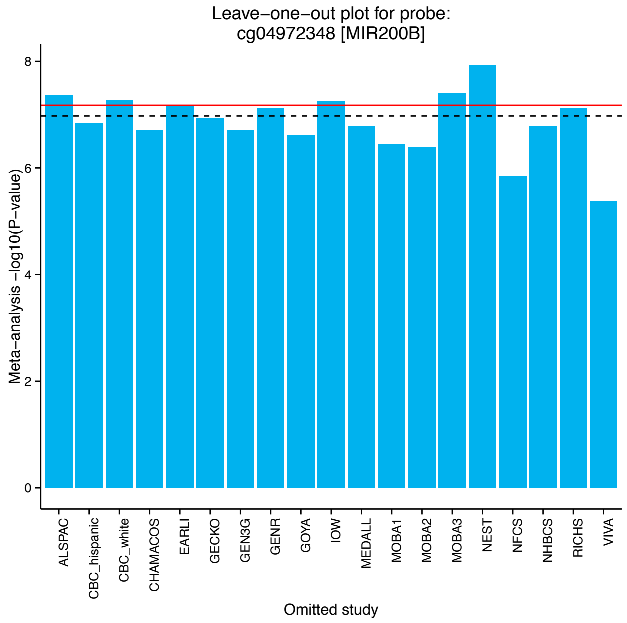
Leave−one−out plot for probe:
cg04972348 [MIR200B]
Meta−analysis −log10(P−value)
0
2
4
6
8
ALSPAC
CBC_hispanic
CBC_white
CHAMACOS
EARLI
GECKO
GEN3G
GENR
GOYA
IOW
MEDALL
MOBA1
MOBA2
MOBA3
NEST
NFCS
NHBCS
RICHS
VIVA
Omitted study

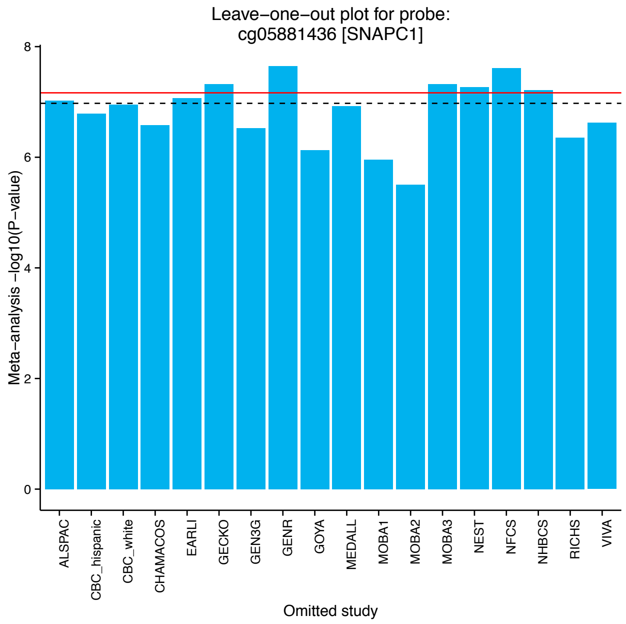
Leave−one−out plot for probe:
cg05881436 [SNAPC1]
Meta−analysis −log10(P−value)
0
2
4
6
8
ALSPAC
CBC_hispanic
CBC_white
CHAMACOS
EARLI
GECKO
GEN3G
GENR
GOYA
MEDALL
MOBA1
MOBA2
MOBA3
NEST
NFCS
NHBCS
RICHS
VIVA
Omitted study

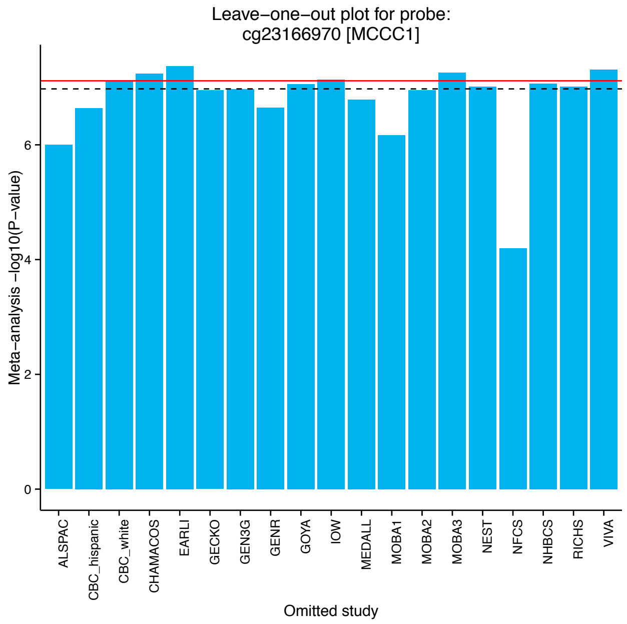
Leave−one−out plot for probe:
cg23166970 [MCCC1]
Meta−analysis −log10(P−value)
0
2
4
6
ALSPAC
CBC_hispanic
CBC_white
CHAMACOS
EARLI
GECKO
GEN3G
GENR
GOYA
IOW
MEDALL
MOBA1
MOBA2
MOBA3
NEST
NFCS
NHBCS
RICHS
VIVA
Omitted study

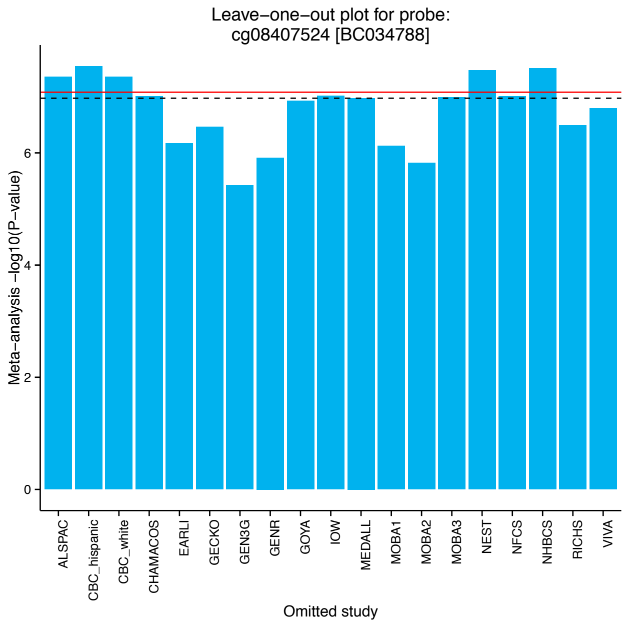
Leave−one−out plot for probe:
cg08407524 [BC034788]
Meta−analysis −log10(P−value)
0
2
4
6
ALSPAC
CBC_hispanic
CBC_white
CHAMACOS
EARLI
GECKO
GEN3G
GENR
GOYA
IOW
MEDALL
MOBA1
MOBA2
MOBA3
NEST
NFCS
NHBCS
RICHS
VIVA
Omitted study

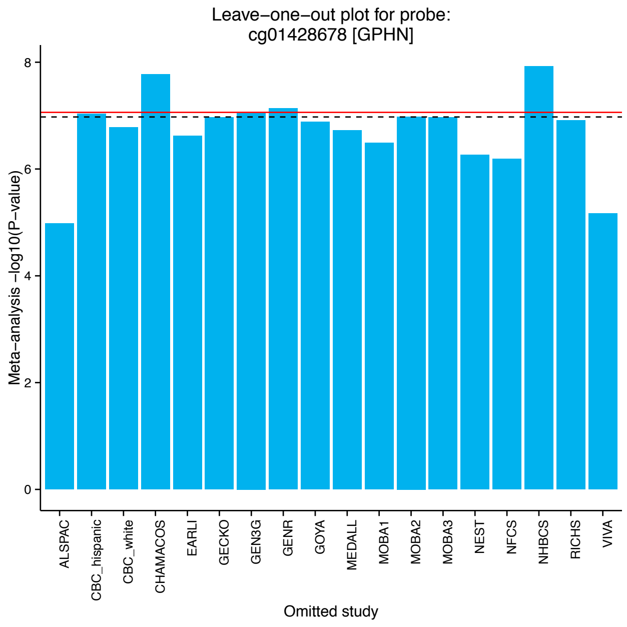
Leave−one−out plot for probe:
cg01428678 [GPHN]
Meta−analysis −log10(P−value)
0
2
4
6
8
ALSPAC
CBC_hispanic
CBC_white
CHAMACOS
EARLI
GECKO
GEN3G
GENR
GOYA
MEDALL
MOBA1
MOBA2
MOBA3
NEST
NFCS
NHBCS
RICHS
VIVA
Omitted study

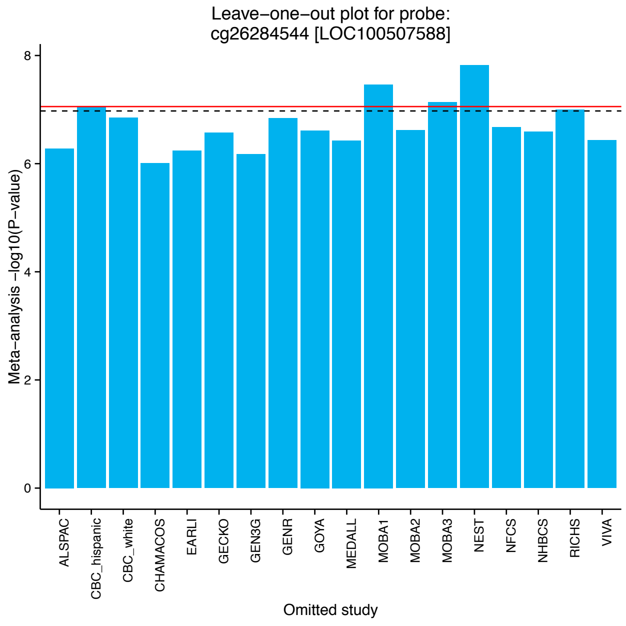
Leave−one−out plot for probe:
cg26284544 [LOC100507588]
Meta−analysis −log10(P−value)
0
2
4
6
8
ALSPAC
CBC_hispanic
CBC_white
CHAMACOS
EARLI
GECKO
GEN3G
GENR
GOYA
MEDALL
MOBA1
MOBA2
MOBA3
NEST
NFCS
NHBCS
RICHS
VIVA
Omitted study

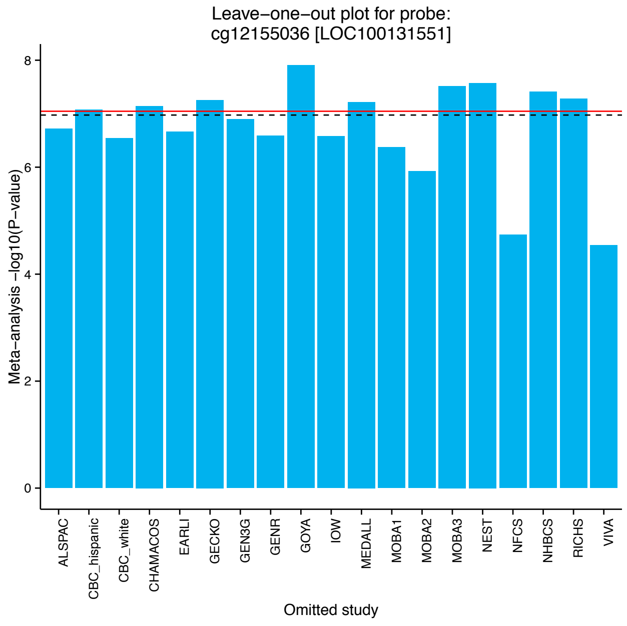
Leave−one−out plot for probe:
cg12155036 [LOC100131551]
Meta−analysis −log10(P−value)
0
2
4
6
8
ALSPAC
CBC_hispanic
CBC_white
CHAMACOS
EARLI
GECKO
GEN3G
GENR
GOYA
IOW
MEDALL
MOBA1
MOBA2
MOBA3
NEST
NFCS
NHBCS
RICHS
VIVA
Omitted study

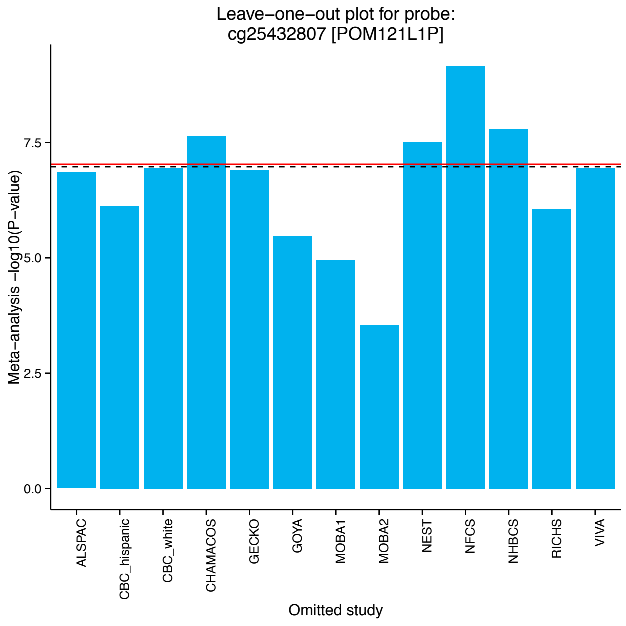
Leave−one−out plot for probe:
cg25432807 [POM121L1P]
Meta−analysis −log10(P−value)
0.0
2.5
5.0
7.5
ALSPAC
CBC_hispanic
CBC_white
CHAMACOS
GECKO
GOYA
MOBA1
MOBA2
NEST
NFCS
NHBCS
RICHS
VIVA
Omitted study

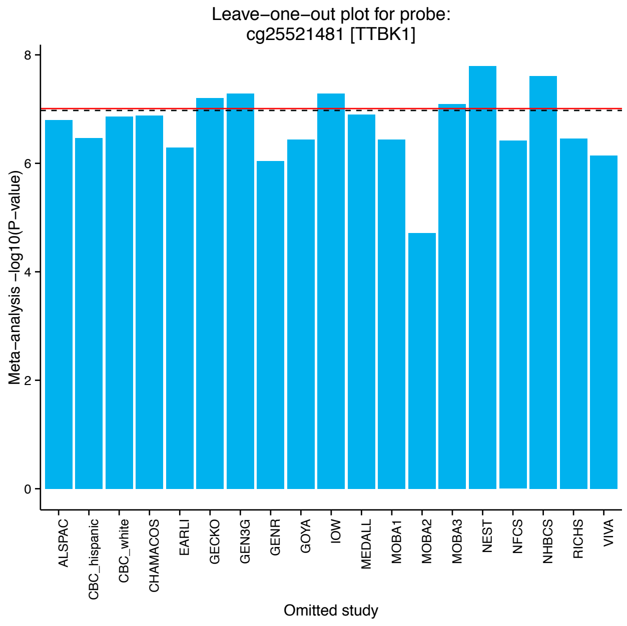
Leave−one−out plot for probe:
cg25521481 [TTBK1]
Meta−analysis −log10(P−value)
0
2
4
6
8
ALSPAC
CBC_hispanic
CBC_white
CHAMACOS
EARLI
GECKO
GEN3G
GENR
GOYA
IOW
MEDALL
MOBA1
MOBA2
MOBA3
NEST
NFCS
NHBCS
RICHS
VIVA
Omitted study

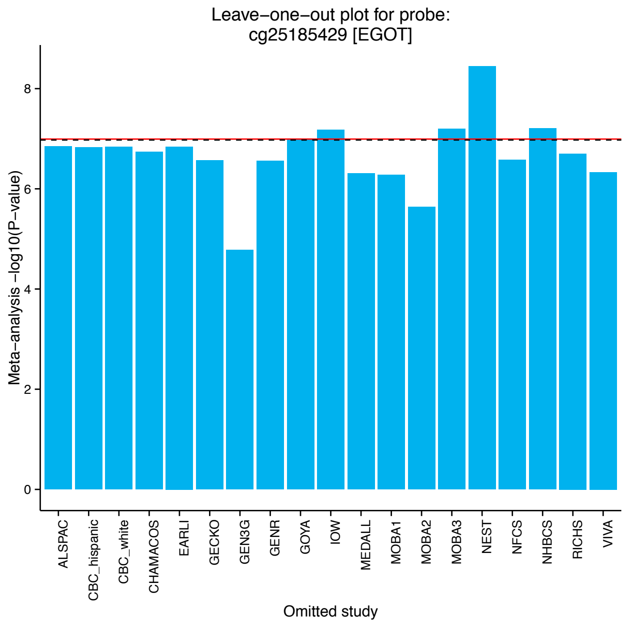
Leave−one−out plot for probe:
cg25185429 [EGOT]
Meta−analysis −log10(P−value)
0
2
4
6
8
ALSPAC
CBC_hispanic
CBC_white
CHAMACOS
EARLI
GECKO
GEN3G
GENR
GOYA
IOW
MEDALL
MOBA1
MOBA2
MOBA3
NEST
NFCS
NHBCS
RICHS
VIVA
Omitted study

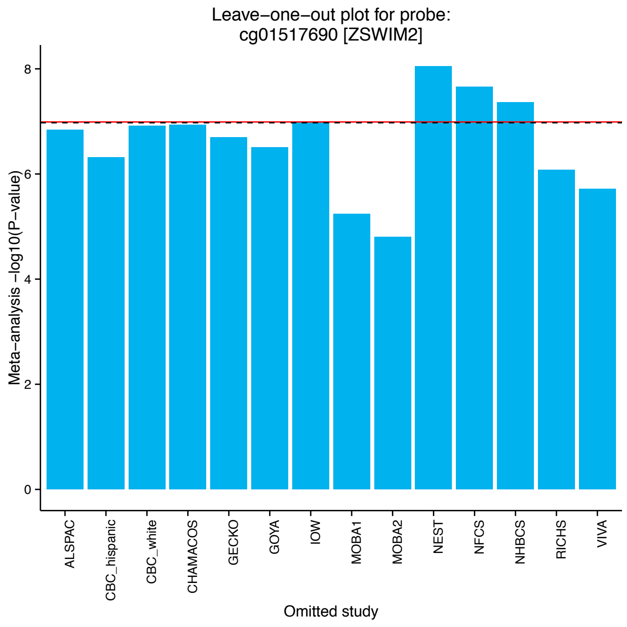
Leave−one−out plot for probe:
cg01517690 [ZSWIM2]
Meta−analysis −log10(P−value)
0
2
4
6
8
ALSPAC
CBC_hispanic
CBC_white
CHAMACOS
GECKO
GOYA
IOW
MOBA1
MOBA2
NEST
NFCS
NHBCS
RICHS
VIVA
Omitted study

Density plots: ALSPAC

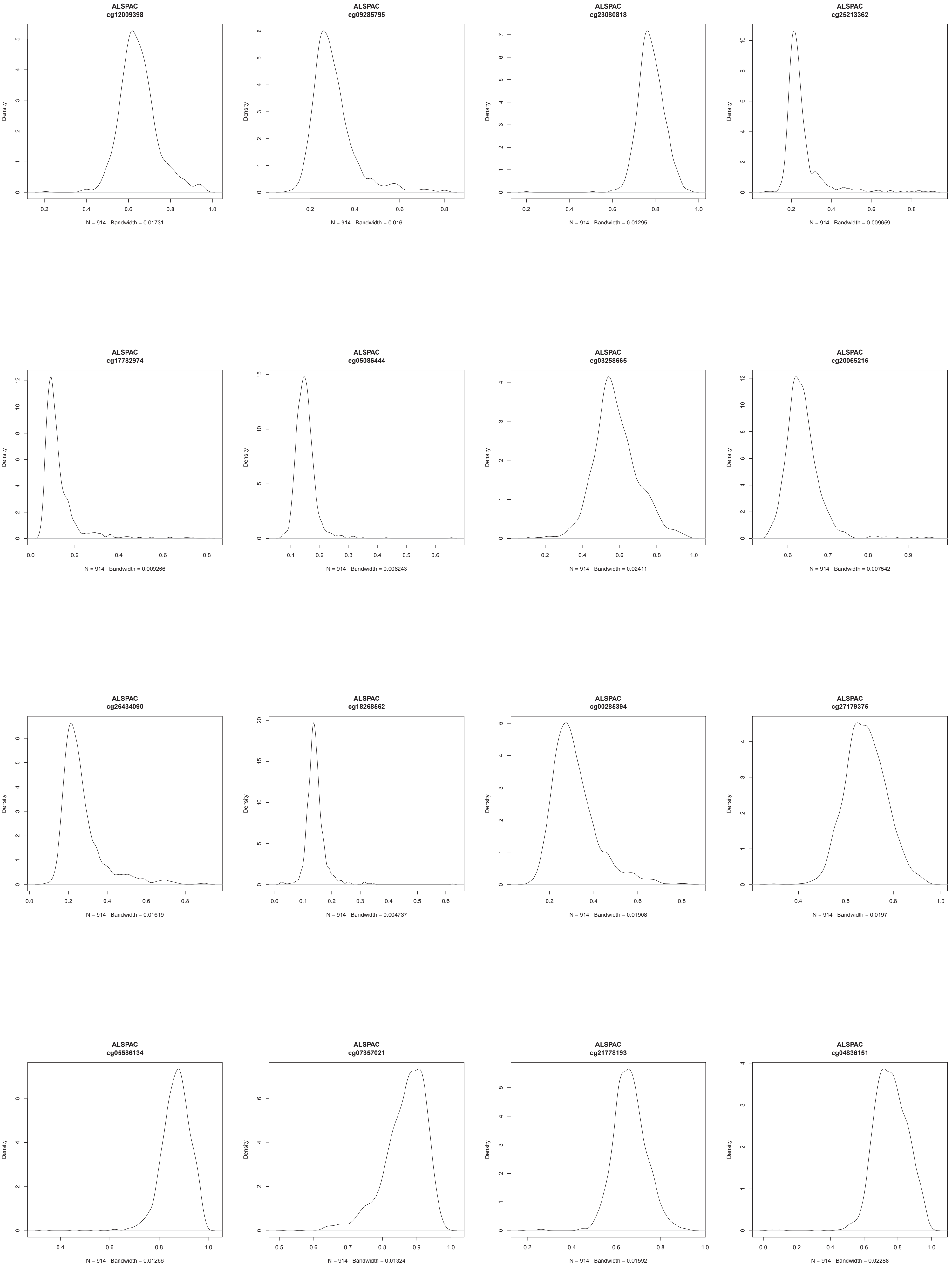
ALSPAC
cg12009398
N = 914 Bandwidth = 0.01731
ALSPAC
cg09285795
N = 914 Bandwidth = 0.016
ALSPAC
cg23080818
N = 914 Bandwidth = 0.01295
ALSPAC
cg25213362
N = 914 Bandwidth = 0.009659
ALSPAC
cg17782974
N = 914 Bandwidth = 0.009266
ALSPAC
cg05086444
N = 914 Bandwidth = 0.006243
ALSPAC
cg03258665
N = 914 Bandwidth = 0.02411
ALSPAC
cg20065216
N = 914 Bandwidth = 0.007542
ALSPAC
cg26434090
N = 914 Bandwidth = 0.01619
ALSPAC
cg18268562
N = 914 Bandwidth = 0.004737
ALSPAC
cg00285394
N = 914 Bandwidth = 0.01908
ALSPAC
cg27179375
N = 914 Bandwidth = 0.0197
ALSPAC
cg05586134
N = 914 Bandwidth = 0.01266
ALSPAC
cg07357021
N = 914 Bandwidth = 0.01324
ALSPAC
cg21778193
N = 914 Bandwidth = 0.01592
ALSPAC
cg04836151
N = 914 Bandwidth = 0.02288
Density

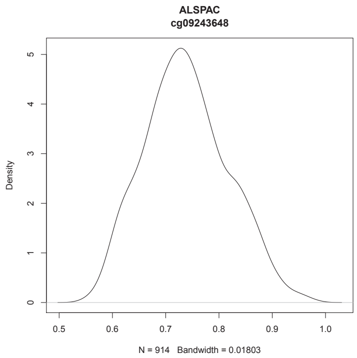
ALSPAC
cg09243648
Density
N = 914 Bandwidth = 0.01803

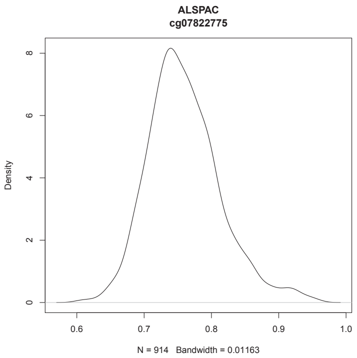
ALSPAC
cg07822775
Density
N = 914 Bandwidth = 0.01163

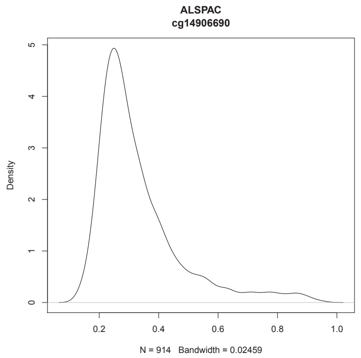
ALSPAC
cg14906690
Density
N = 914 Bandwidth = 0.02459

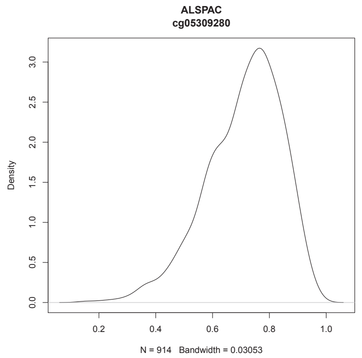
ALSPAC
cg05309280
Density
N = 914 Bandwidth = 0.03053

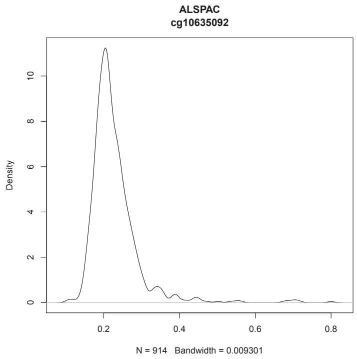
ALSPAC
cg10635092
Density
N = 914 Bandwidth = 0.009301

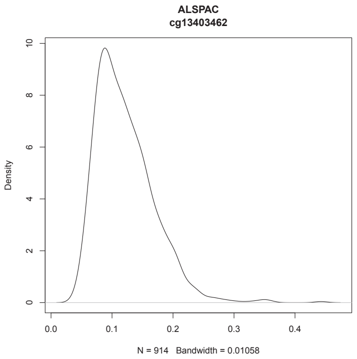
ALSPAC
cg13403462
Density
N = 914 Bandwidth = 0.01058

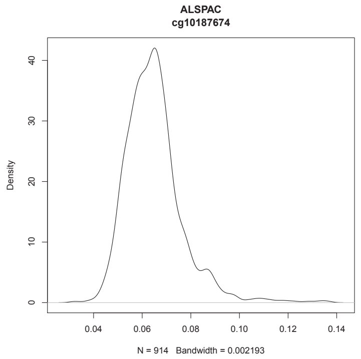
ALSPAC
cg10187674
Density
N = 914 Bandwidth = 0.002193

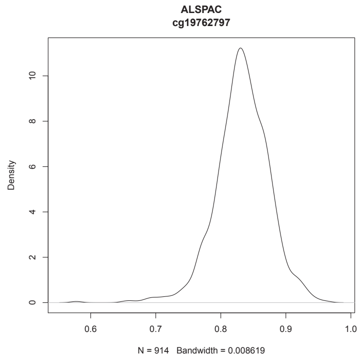
ALSPAC
cg19762797
Density
N = 914 Bandwidth = 0.008619

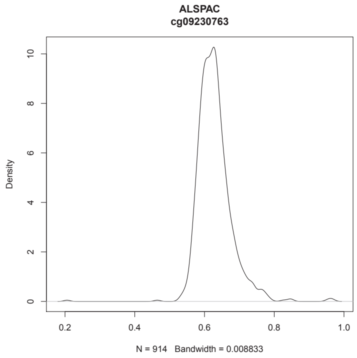
ALSPAC
cg09230763
Density
N = 914 Bandwidth = 0.008833

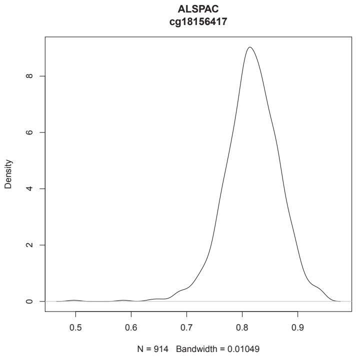
ALSPAC
cg18156417
Density
N = 914 Bandwidth = 0.01049

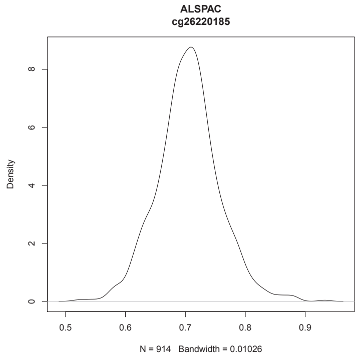
ALSPAC
cg26220185
Density
N = 914 Bandwidth = 0.01026

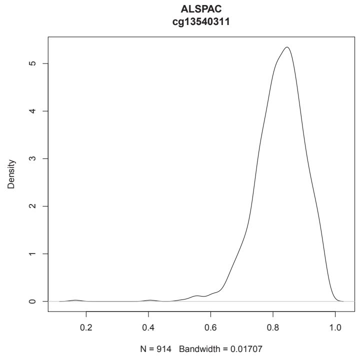
ALSPAC
cg13540311
Density
N = 914 Bandwidth = 0.01707

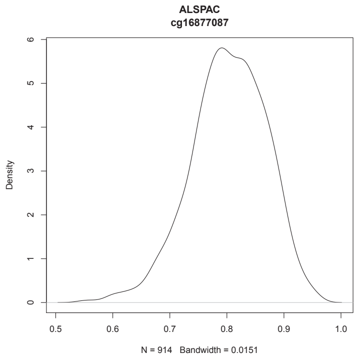
ALSPAC
cg16877087
Density
N = 914 Bandwidth = 0.0151

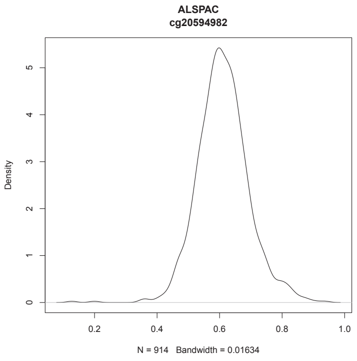
ALSPAC
cg20594982
Density
N = 914 Bandwidth = 0.01634

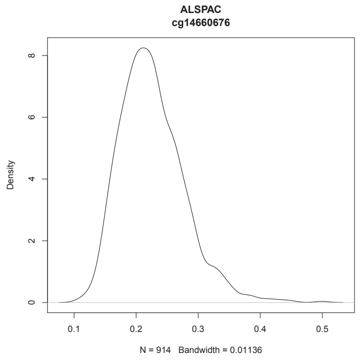
ALSPAC
cg14660676
Density
N = 914 Bandwidth = 0.01136

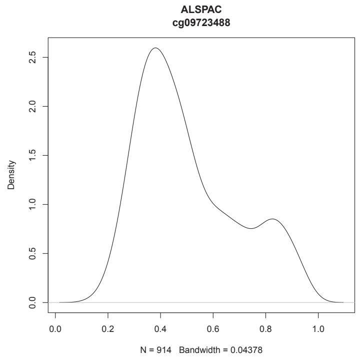
ALSPAC
cg09723488
Density
N = 914 Bandwidth = 0.04378

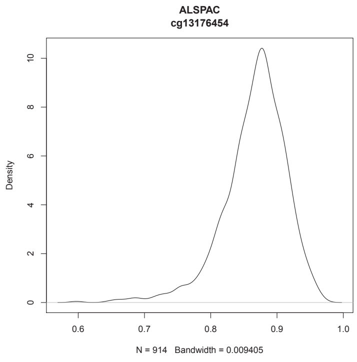
ALSPAC
cg13176454
Density
N = 914   Bandwidth = 0.009405

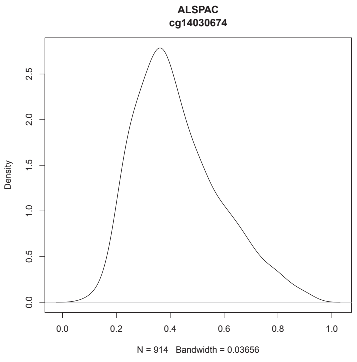
ALSPAC
cg14030674
Density
N = 914   Bandwidth = 0.03656

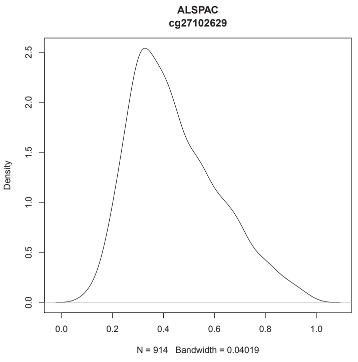
ALSPAC
cg27102629
Density
N = 914   Bandwidth = 0.04019

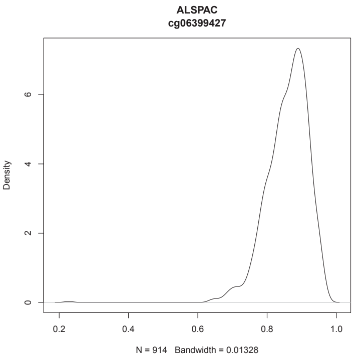
ALSPAC
cg06399427
Density
N = 914   Bandwidth = 0.01328

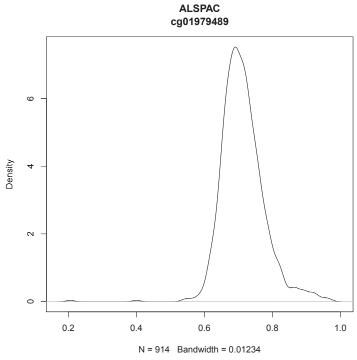
ALSPAC
cg01979489
Density
N = 914   Bandwidth = 0.01234

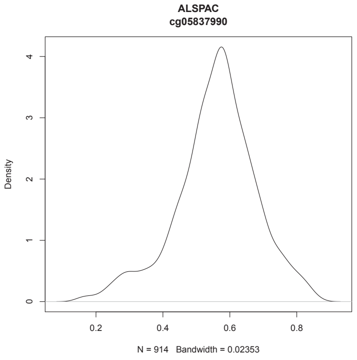
ALSPAC
cg05837990
Density
N = 914   Bandwidth = 0.02353

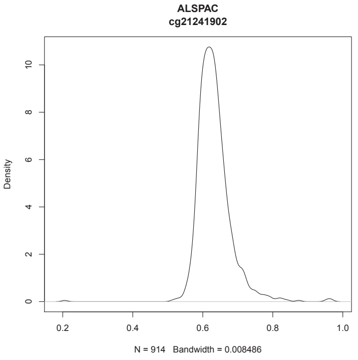
ALSPAC
cg21241902
Density
N = 914   Bandwidth = 0.008486

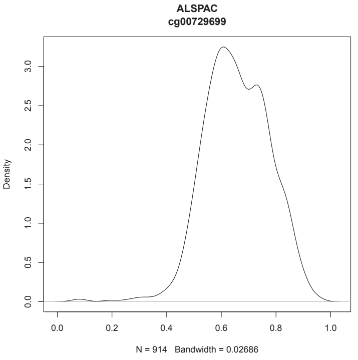
ALSPAC
cg00729699
Density
N = 914   Bandwidth = 0.02686

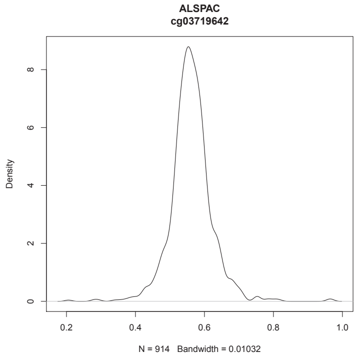
ALSPAC
cg03719642
Density
N = 914   Bandwidth = 0.01032

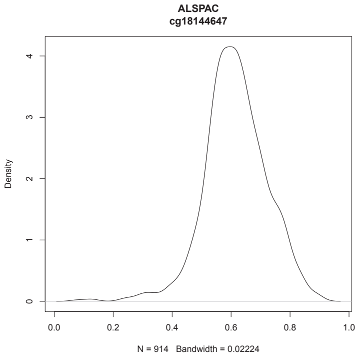
ALSPAC
cg18144647
Density
N = 914   Bandwidth = 0.02224

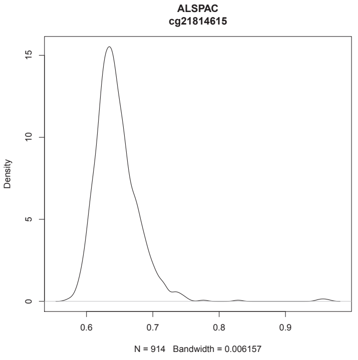
ALSPAC
cg21814615
Density
N = 914   Bandwidth = 0.006157

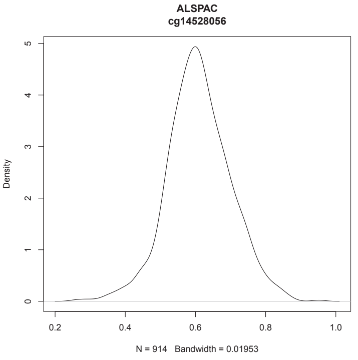
ALSPAC
cg14528056
Density
N = 914   Bandwidth = 0.01953

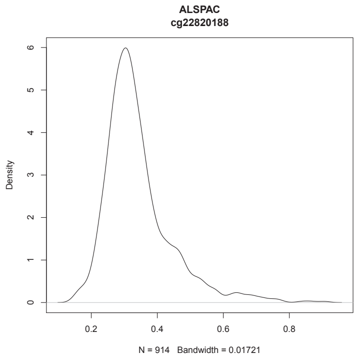
ALSPAC
cg22820188
Density
N = 914   Bandwidth = 0.01721

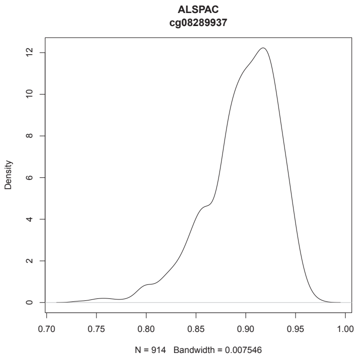
ALSPAC
cg08289937
Density
N = 914   Bandwidth = 0.007546

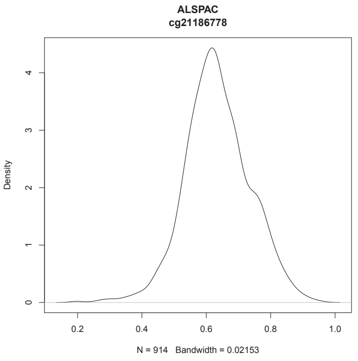
ALSPAC
cg21186778
Density
N = 914   Bandwidth = 0.02153

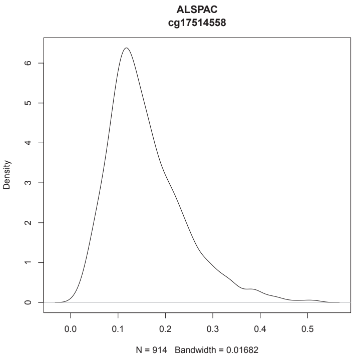
ALSPAC
cg17514558
Density
N = 914   Bandwidth = 0.01682

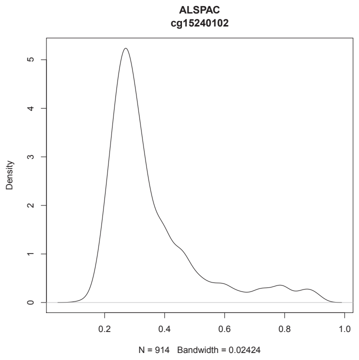
ALSPAC
cg15240102
Density
N = 914 Bandwidth = 0.02424

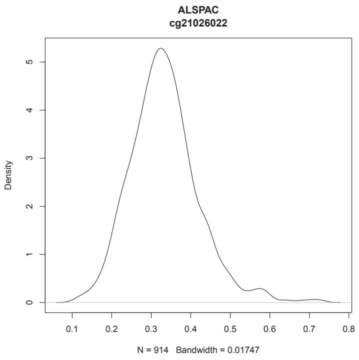
ALSPAC
cg21026022
Density
N = 914 Bandwidth = 0.01747

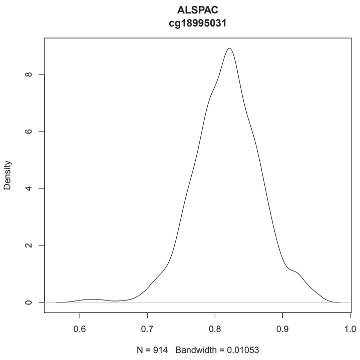
ALSPAC
cg18995031
Density
N = 914 Bandwidth = 0.01053

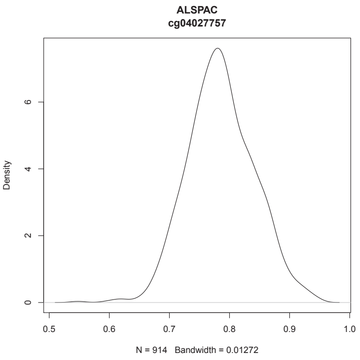
ALSPAC
cg04027757
Density
N = 914 Bandwidth = 0.01272

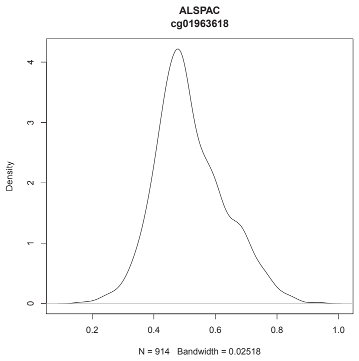
ALSPAC
cg01963618
Density
N = 914 Bandwidth = 0.02518

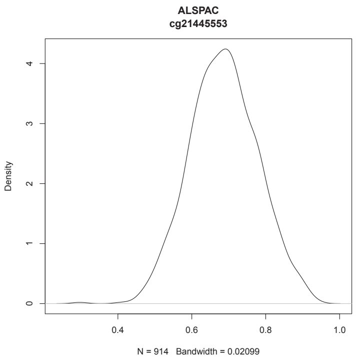
ALSPAC
cg21445553
Density
N = 914 Bandwidth = 0.02099

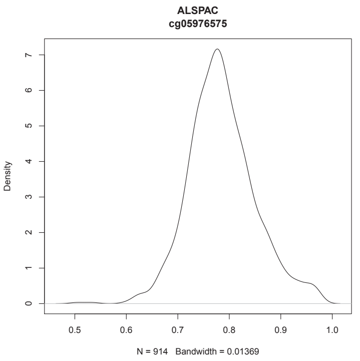
ALSPAC
cg05976575
Density
N = 914 Bandwidth = 0.01369

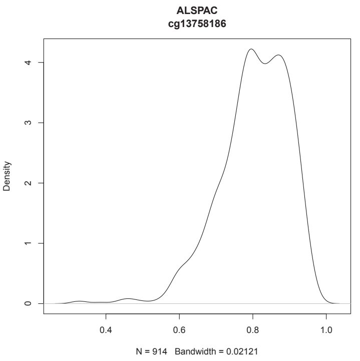
ALSPAC
cg13758186
Density
N = 914 Bandwidth = 0.02121

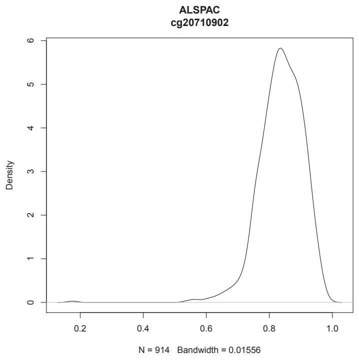
ALSPAC
cg20710902
Density
N = 914 Bandwidth = 0.01556

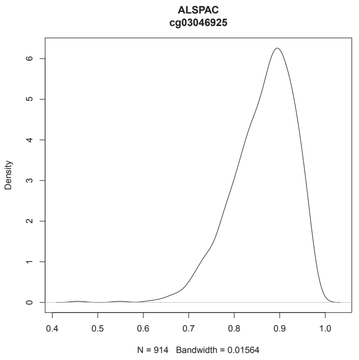
ALSPAC
cg03046925
Density
N = 914 Bandwidth = 0.01564

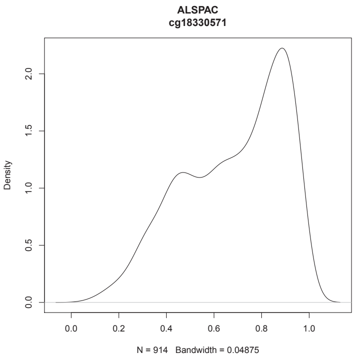
ALSPAC
cg18330571
Density
N = 914 Bandwidth = 0.04875

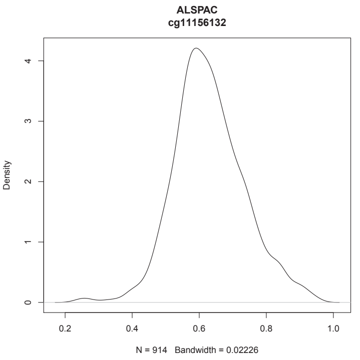
ALSPAC
cg11156132
Density
N = 914 Bandwidth = 0.02226

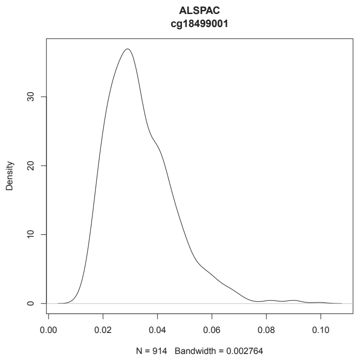
ALSPAC
cg18499001
Density
N = 914 Bandwidth = 0.002764

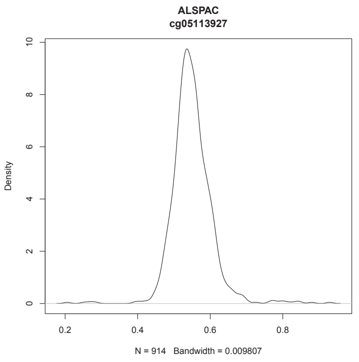
ALSPAC
cg05113927
Density
N = 914 Bandwidth = 0.009807

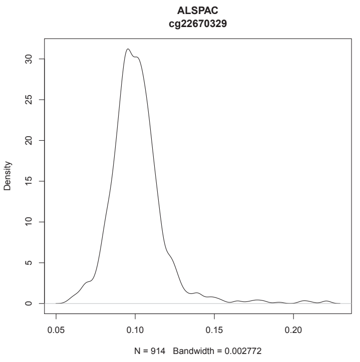
ALSPAC
cg22670329
Density
N = 914 Bandwidth = 0.002772

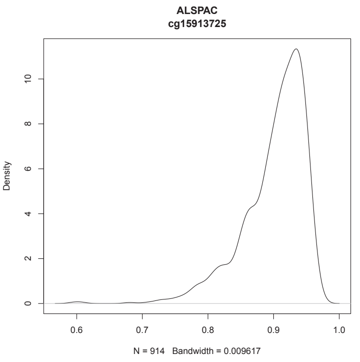
ALSPAC
cg15913725
Density
N = 914 Bandwidth = 0.009617

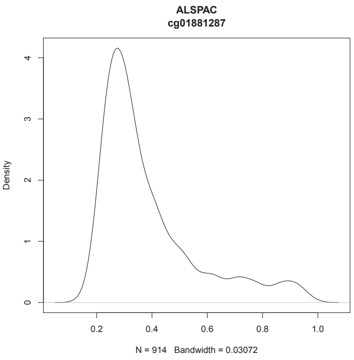
ALSPAC
cg01881287
Density
N = 914 Bandwidth = 0.03072

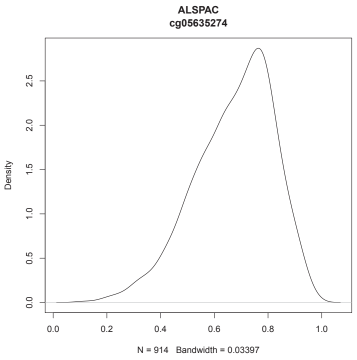
ALSPAC
cg05635274
Density
N = 914 Bandwidth = 0.03397

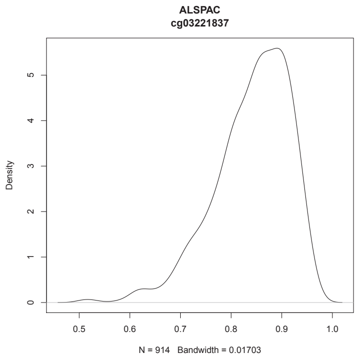
ALSPAC
cg03221837
Density
N = 914 Bandwidth = 0.01703

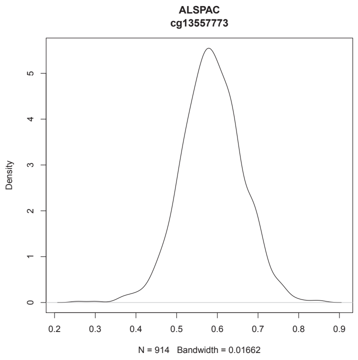
ALSPAC
cg13557773
Density
N = 914 Bandwidth = 0.01662

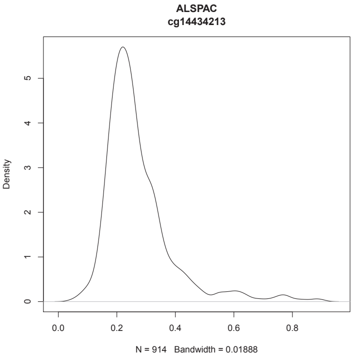
ALSPAC
cg14434213
Density
N = 914 Bandwidth = 0.01888

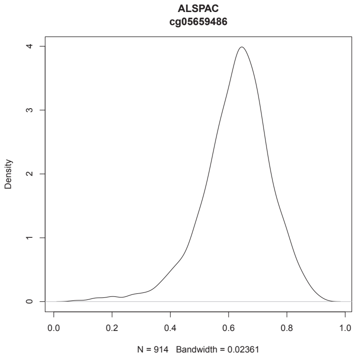
ALSPAC
cg05659486
Density
N = 914 Bandwidth = 0.02361

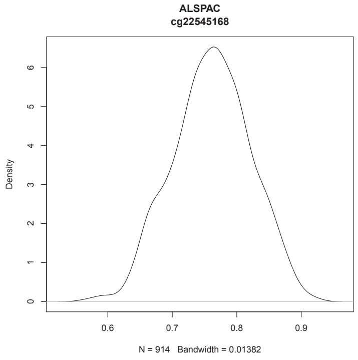
ALSPAC
cg22545168
Density
N = 914 Bandwidth = 0.01382

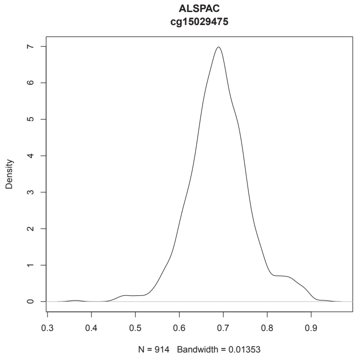
ALSPAC
cg15029475
Density
N = 914 Bandwidth = 0.01353

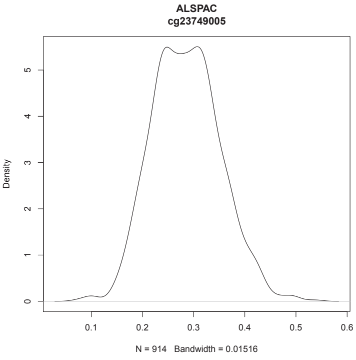
ALSPAC
cg23749005
Density
N = 914 Bandwidth = 0.01516

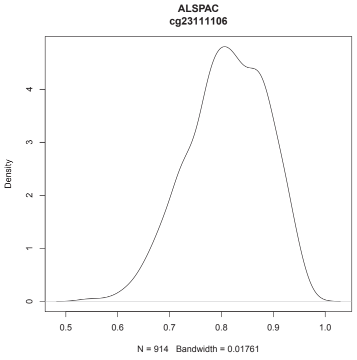
ALSPAC
cg23111106
Density
N = 914 Bandwidth = 0.01761

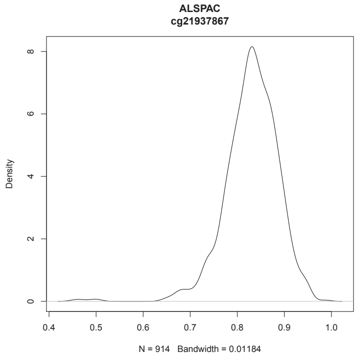
ALSPAC
cg21937867
Density
N = 914 Bandwidth = 0.01184

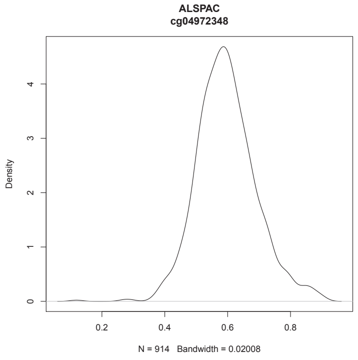
ALSPAC
cg04972348
Density
N = 914 Bandwidth = 0.02008

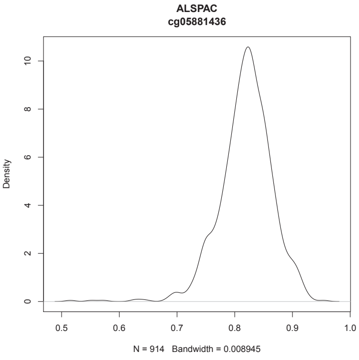
ALSPAC
cg05881436
Density
N = 914 Bandwidth = 0.008945

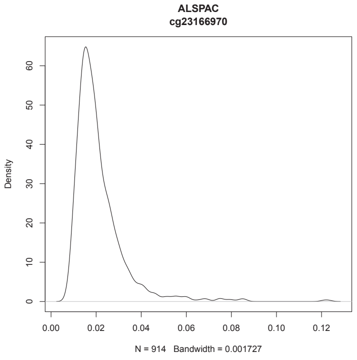
ALSPAC
cg23166970
Density
N = 914 Bandwidth = 0.001727

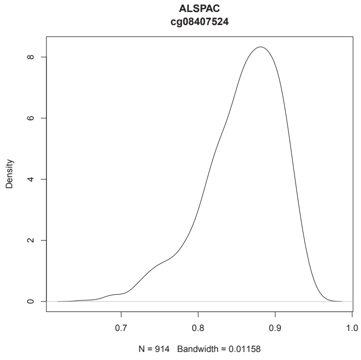
ALSPAC
cg08407524
Density
N = 914 Bandwidth = 0.01158

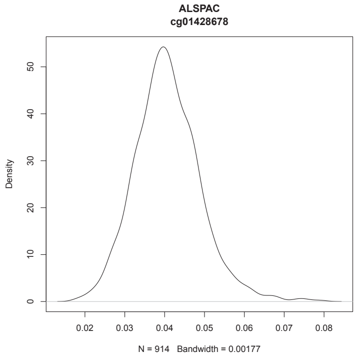
ALSPAC
cg01428678
Density
N = 914 Bandwidth = 0.00177

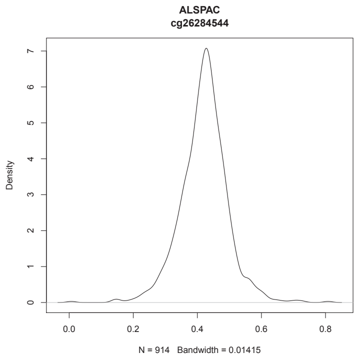
ALSPAC
cg26284544
Density
N = 914   Bandwidth = 0.01415

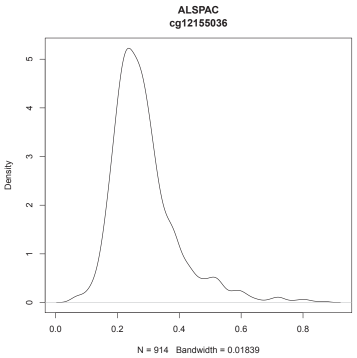
ALSPAC
cg12155036
Density
N = 914   Bandwidth = 0.01839

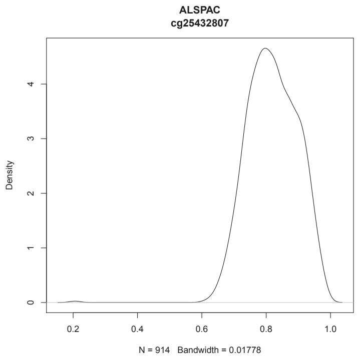
ALSPAC
cg25432807
Density
N = 914   Bandwidth = 0.01778

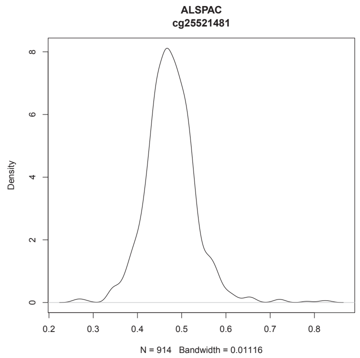
ALSPAC
cg25521481
Density
N = 914   Bandwidth = 0.01116

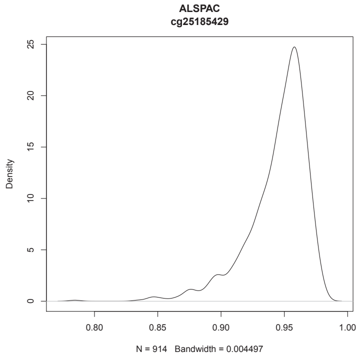
ALSPAC
cg25185429
Density
N = 914   Bandwidth = 0.004497

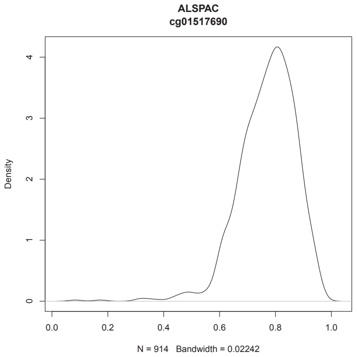
ALSPAC
cg01517690
Density
N = 914   Bandwidth = 0.02242

Density plots: GOYA

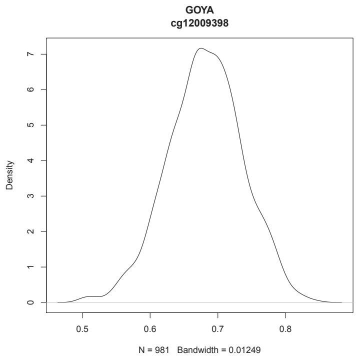
GOYA
cg12009398
Density
N = 981 Bandwidth = 0.01249

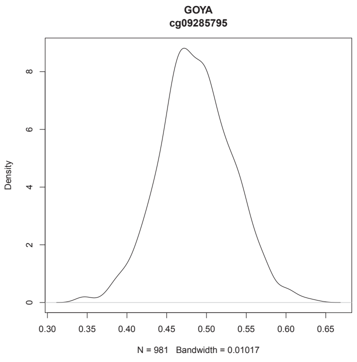
GOYA
cg09285795
Density
N = 981 Bandwidth = 0.01017

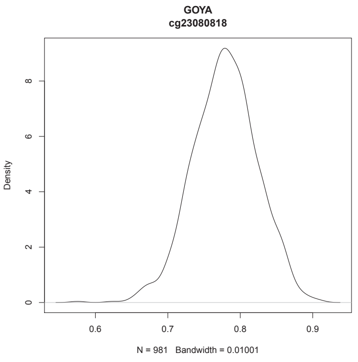
GOYA
cg23080818
Density
N = 981 Bandwidth = 0.01001

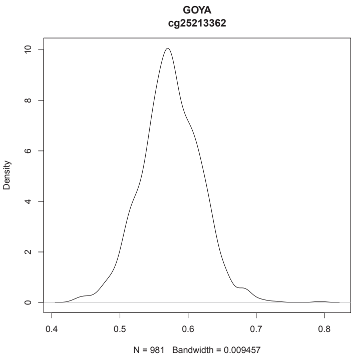
GOYA
cg25213362
Density
N = 981 Bandwidth = 0.009457

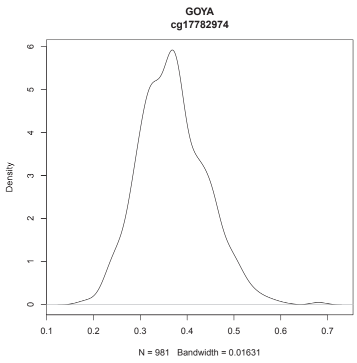
GOYA
cg17782974
Density
N = 981 Bandwidth = 0.01631

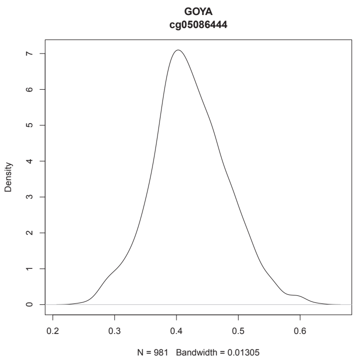
GOYA
cg05086444
Density
N = 981 Bandwidth = 0.01305

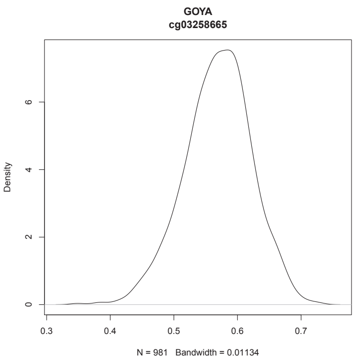
GOYA
cg03258665
Density
N = 981 Bandwidth = 0.01134

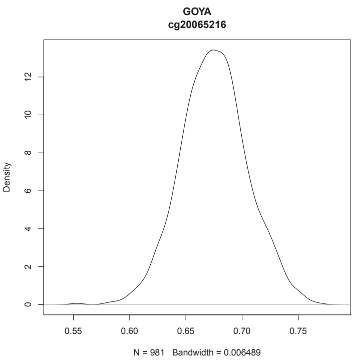
GOYA
cg20065216
Density
N = 981 Bandwidth = 0.006489

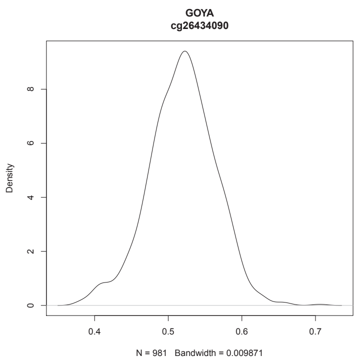
GOYA
cg26434090
Density
N = 981 Bandwidth = 0.009871

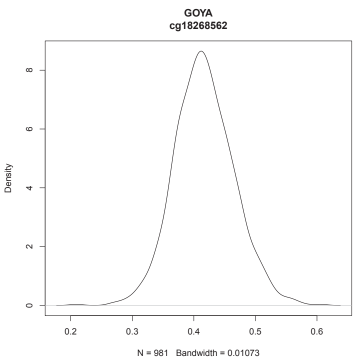
GOYA
cg18268562
Density
N = 981 Bandwidth = 0.01073

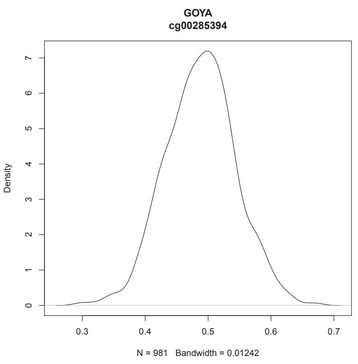
GOYA
cg00285394
Density
N = 981 Bandwidth = 0.01242

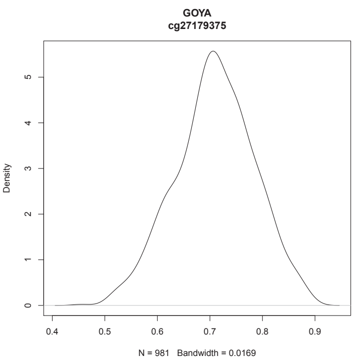
GOYA
cg27179375
Density
N = 981 Bandwidth = 0.0169

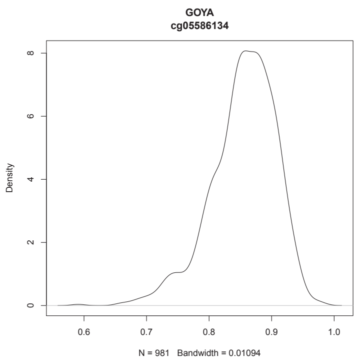
GOYA
cg05586134
Density
N = 981 Bandwidth = 0.01094

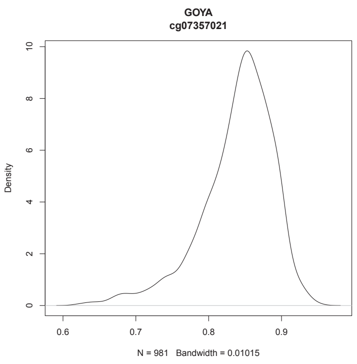
GOYA
cg07357021
Density
N = 981 Bandwidth = 0.01015

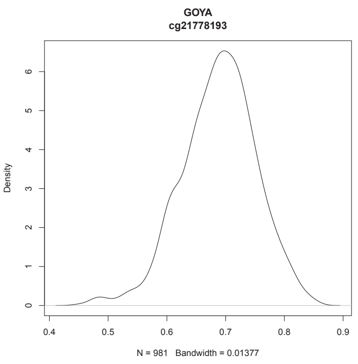
GOYA
cg21778193
Density
N = 981 Bandwidth = 0.01377

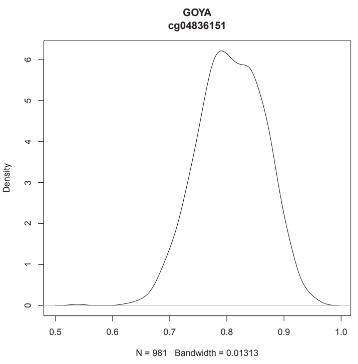
GOYA
cg04836151
Density
N = 981 Bandwidth = 0.01313

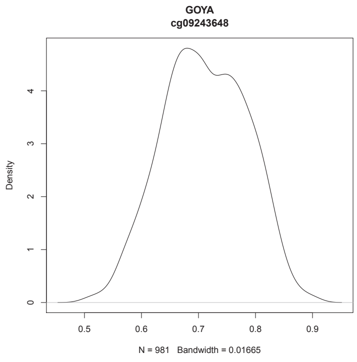
GOYA
cg09243648
N = 981 Bandwidth = 0.01665

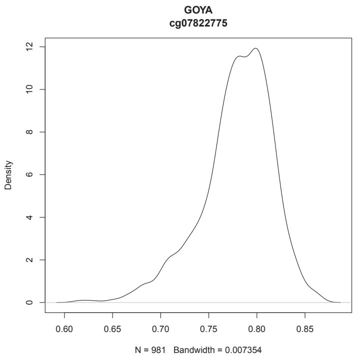
GOYA
cg07822775
N = 981 Bandwidth = 0.007354

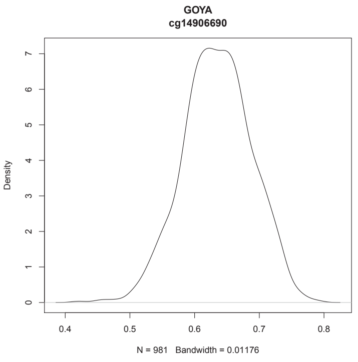
GOYA
cg14906690
N = 981 Bandwidth = 0.01176

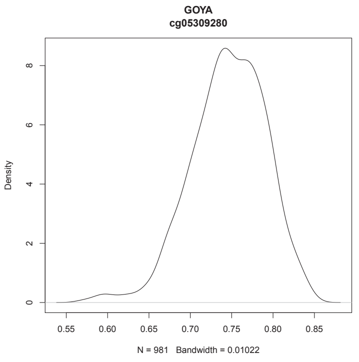
GOYA
cg05309280
N = 981 Bandwidth = 0.01022

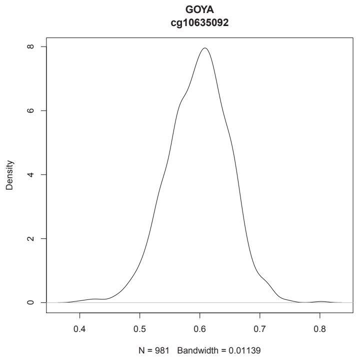
GOYA
cg10635092
N = 981 Bandwidth = 0.01139

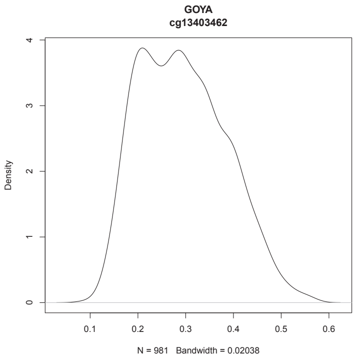
GOYA
cg13403462
N = 981 Bandwidth = 0.02038

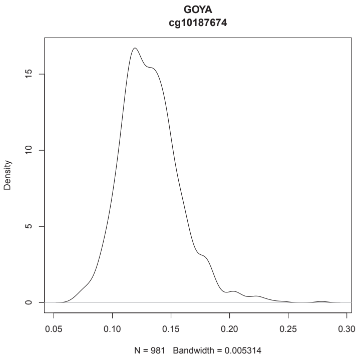
GOYA
cg10187674
N = 981 Bandwidth = 0.005314

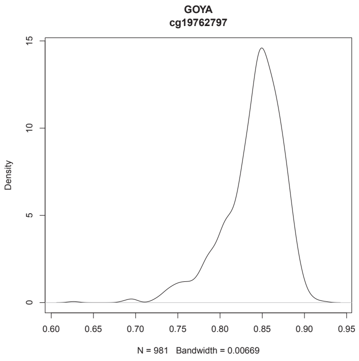
GOYA
cg19762797
N = 981 Bandwidth = 0.00669

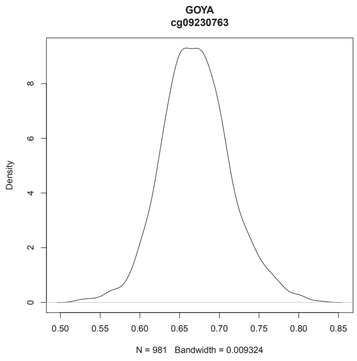
GOYA
cg09230763
N = 981 Bandwidth = 0.009324

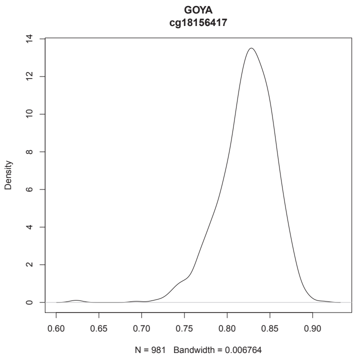
GOYA
cg18156417
N = 981 Bandwidth = 0.006764

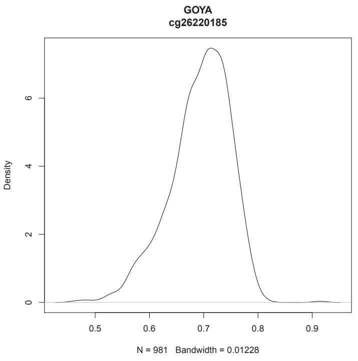
GOYA
cg26220185
N = 981 Bandwidth = 0.01228

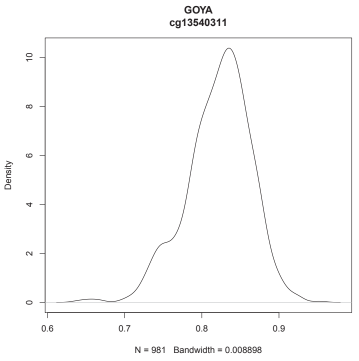
GOYA
cg13540311
N = 981 Bandwidth = 0.008898

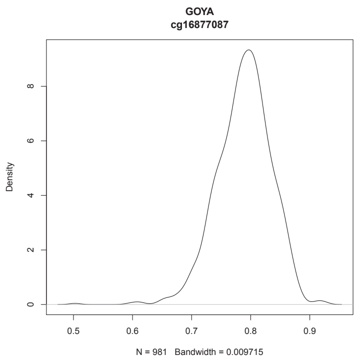
GOYA
cg16877087
N = 981 Bandwidth = 0.009715

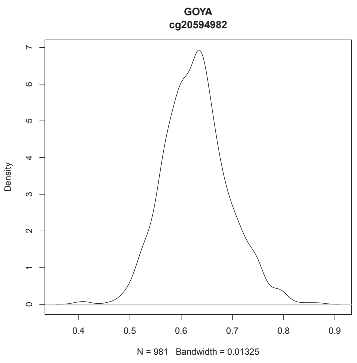
GOYA
cg20594982
N = 981 Bandwidth = 0.01325

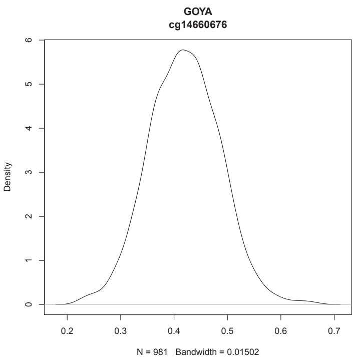
GOYA
cg14660676
N = 981 Bandwidth = 0.01502

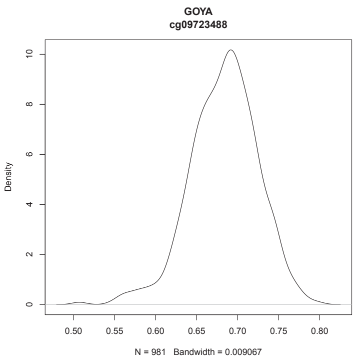
GOYA
cg09723488
N = 981 Bandwidth = 0.009067

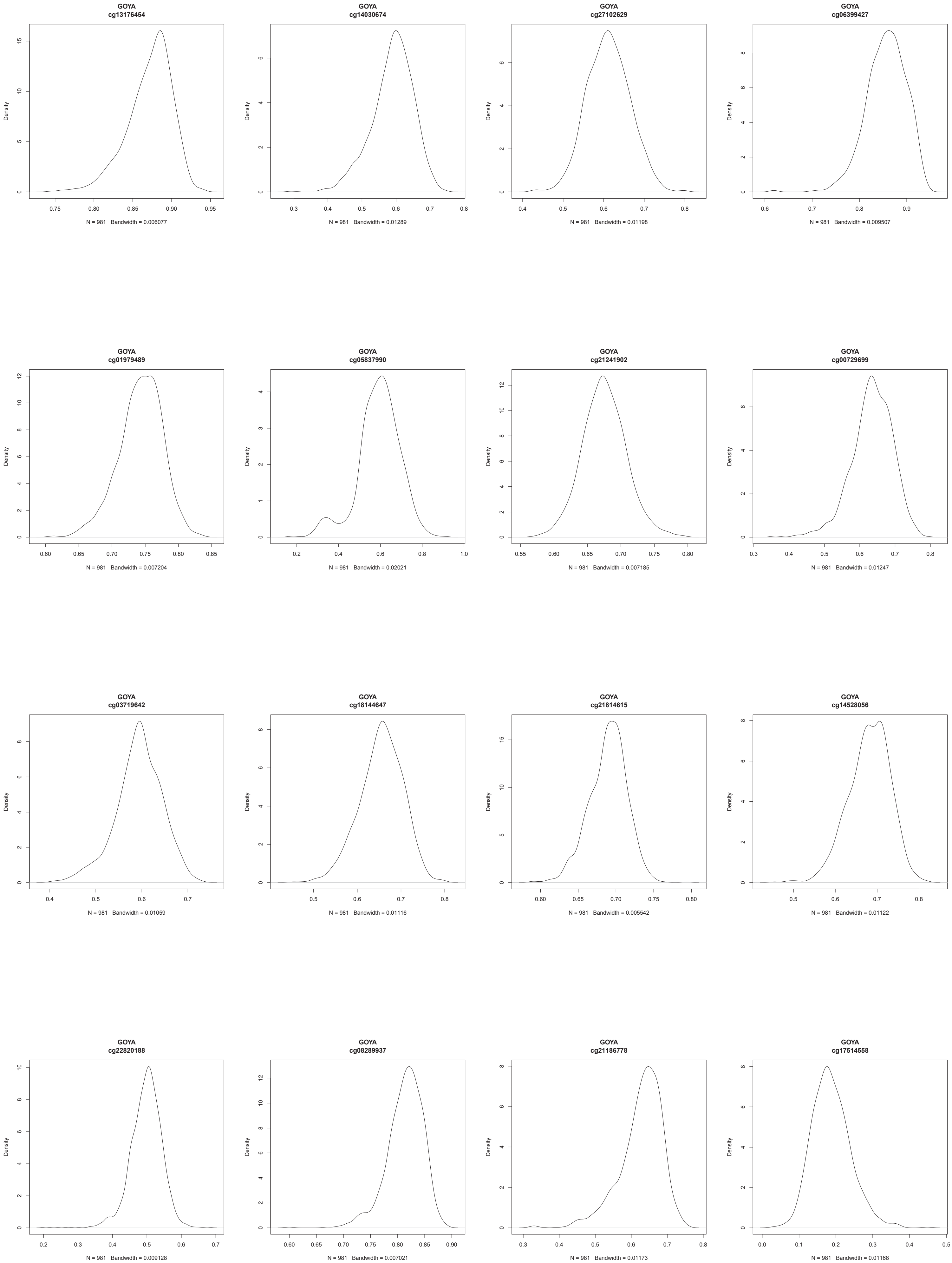
GOYA
cg13176454
N = 981 Bandwidth = 0.006077
GOYA
cg14030674
N = 981 Bandwidth = 0.01289
GOYA
cg27102629
N = 981 Bandwidth = 0.01198
GOYA
cg06399427
N = 981 Bandwidth = 0.009507
GOYA
cg01979489
N = 981 Bandwidth = 0.007204
GOYA
cg05837990
N = 981 Bandwidth = 0.02021
GOYA
cg21241902
N = 981 Bandwidth = 0.007185
GOYA
cg00729699
N = 981 Bandwidth = 0.01247
GOYA
cg03719642
N = 981 Bandwidth = 0.01059
GOYA
cg18144647
N = 981 Bandwidth = 0.01116
GOYA
cg21814615
N = 981 Bandwidth = 0.005542
GOYA
cg14528056
N = 981 Bandwidth = 0.01122
GOYA
cg22820188
N = 981 Bandwidth = 0.009128
GOYA
cg08289937
N = 981 Bandwidth = 0.007021
GOYA
cg21186778
N = 981 Bandwidth = 0.01173
GOYA
cg17514558
N = 981 Bandwidth = 0.01168
Density

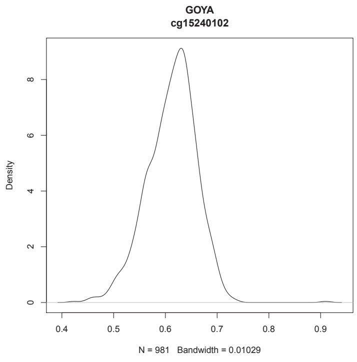
GOYA
cg15240102
Density
N = 981 Bandwidth = 0.01029
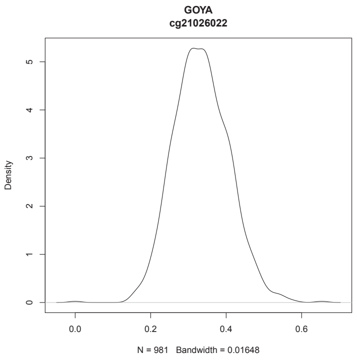
GOYA
cg21026022
Density
N = 981 Bandwidth = 0.01648
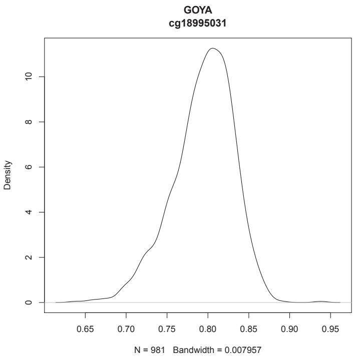
GOYA
cg18995031
Density
N = 981 Bandwidth = 0.007957
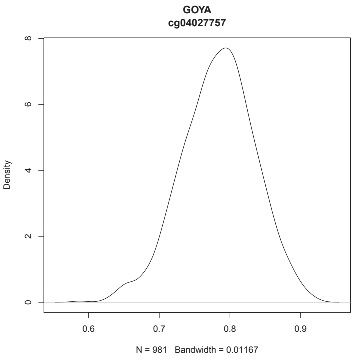
GOYA
cg04027757
Density
N = 981 Bandwidth = 0.01167
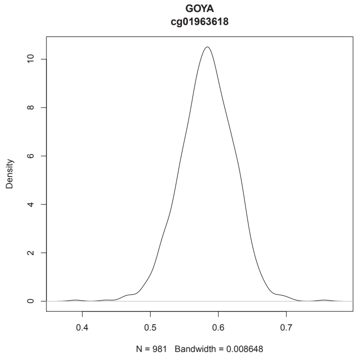
GOYA
cg01963618
Density
N = 981 Bandwidth = 0.008648
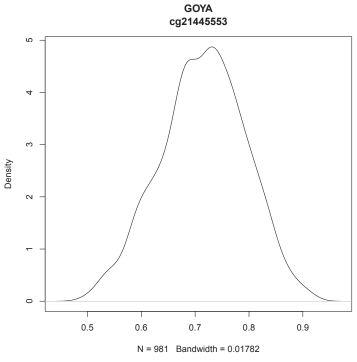
GOYA
cg21445553
Density
N = 981 Bandwidth = 0.01782
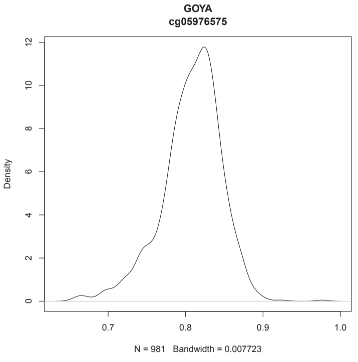
GOYA
cg05976575
Density
N = 981 Bandwidth = 0.007723
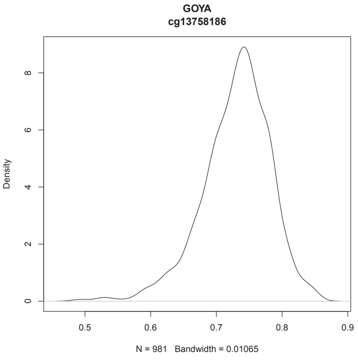
GOYA
cg13758186
Density
N = 981 Bandwidth = 0.01065
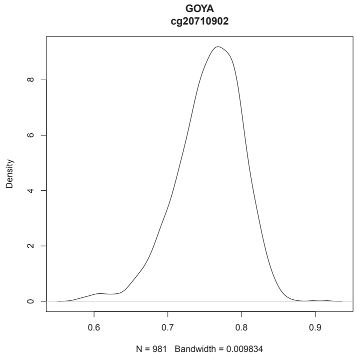
GOYA
cg20710902
Density
N = 981 Bandwidth = 0.009834
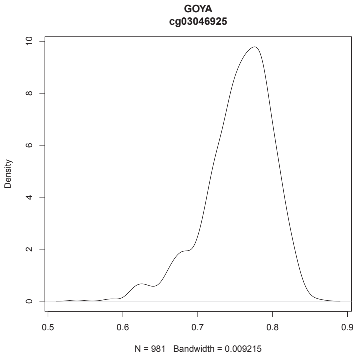
GOYA
cg03046925
Density
N = 981 Bandwidth = 0.009215
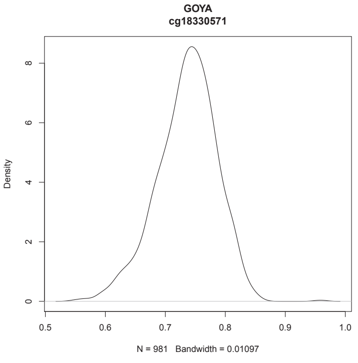
GOYA
cg18330571
Density
N = 981 Bandwidth = 0.01097
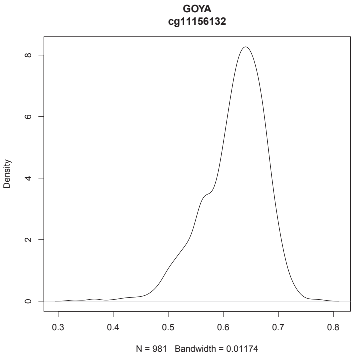
GOYA
cg11156132
Density
N = 981 Bandwidth = 0.01174
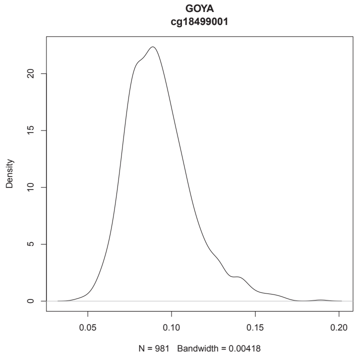
GOYA
cg18499001
Density
N = 981 Bandwidth = 0.00418
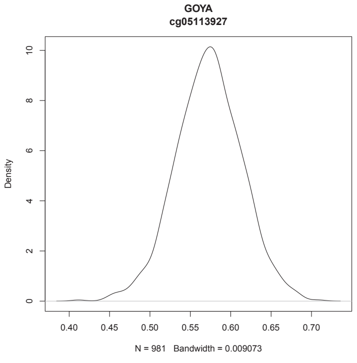
GOYA
cg05113927
Density
N = 981 Bandwidth = 0.009073
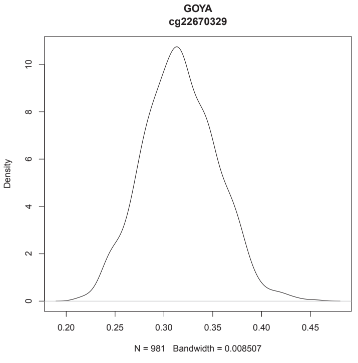
GOYA
cg22670329
Density
N = 981 Bandwidth = 0.008507
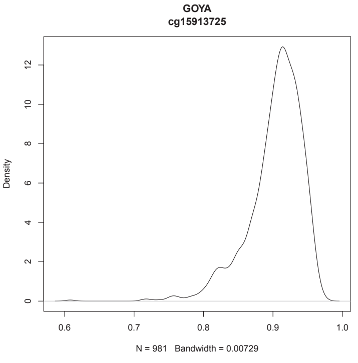
GOYA
cg15913725
Density
N = 981 Bandwidth = 0.00729

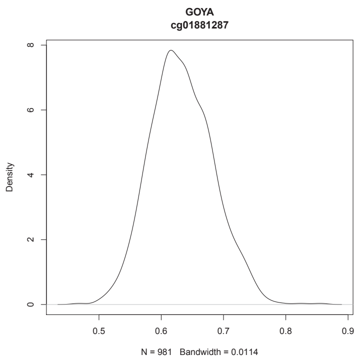
GOYA
cg01881287
Density
N = 981 Bandwidth = 0.0114

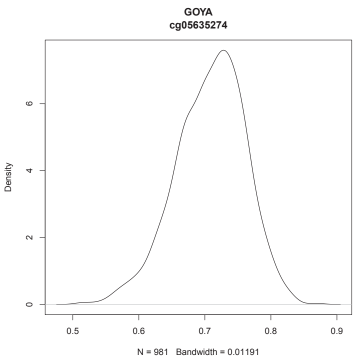
GOYA
cg05635274
Density
N = 981 Bandwidth = 0.01191

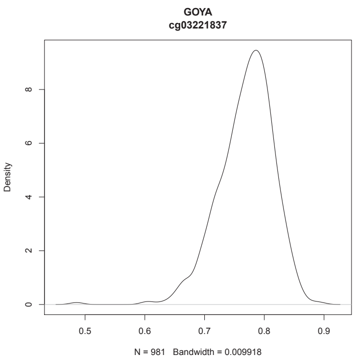
GOYA
cg03221837
Density
N = 981 Bandwidth = 0.009918

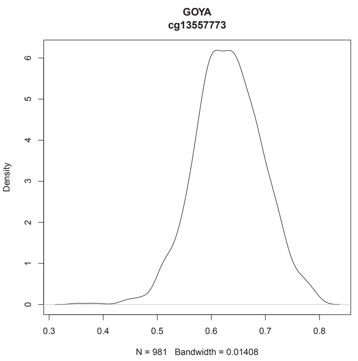
GOYA
cg13557773
Density
N = 981 Bandwidth = 0.01408

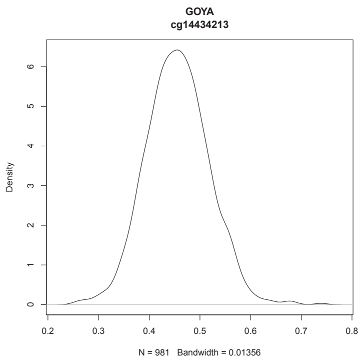
GOYA
cg14434213
Density
N = 981 Bandwidth = 0.01356

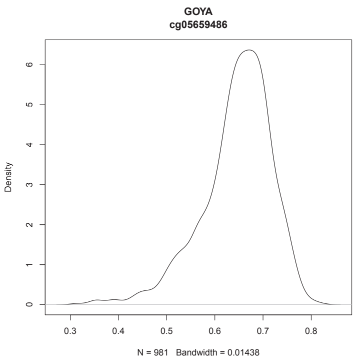
GOYA
cg05659486
Density
N = 981 Bandwidth = 0.01438

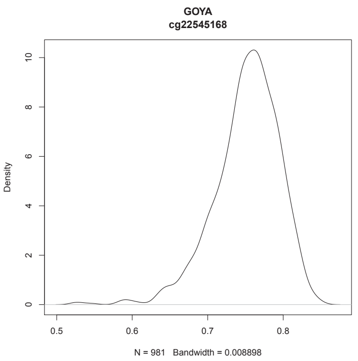
GOYA
cg22545168
Density
N = 981 Bandwidth = 0.008898

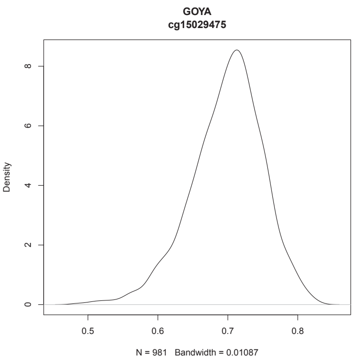
GOYA
cg15029475
Density
N = 981 Bandwidth = 0.01087

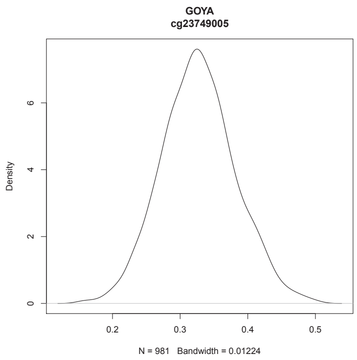
GOYA
cg23749005
Density
N = 981 Bandwidth = 0.01224

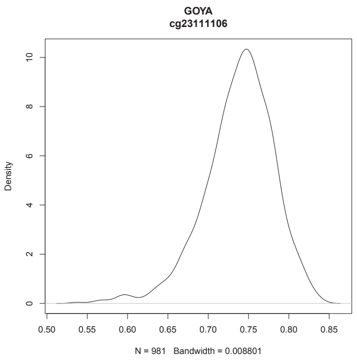
GOYA
cg23111106
Density
N = 981 Bandwidth = 0.008801

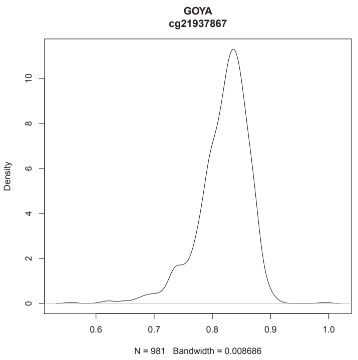
GOYA
cg21937867
Density
N = 981 Bandwidth = 0.008686

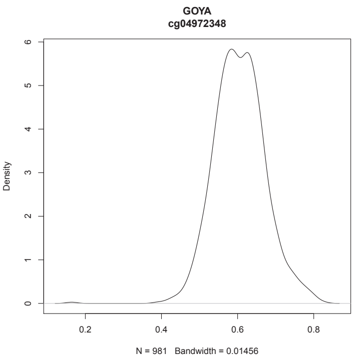
GOYA
cg04972348
Density
N = 981 Bandwidth = 0.01456

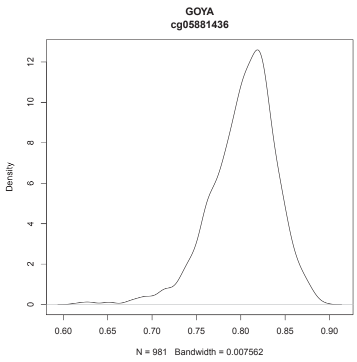
GOYA
cg05881436
Density
N = 981 Bandwidth = 0.007562

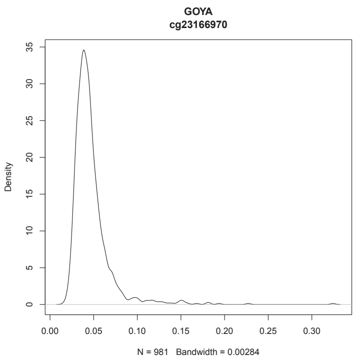
GOYA
cg23166970
Density
N = 981 Bandwidth = 0.00284

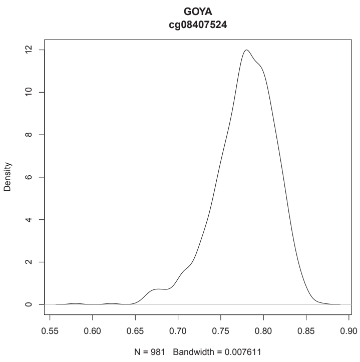
GOYA
cg08407524
Density
N = 981 Bandwidth = 0.007611

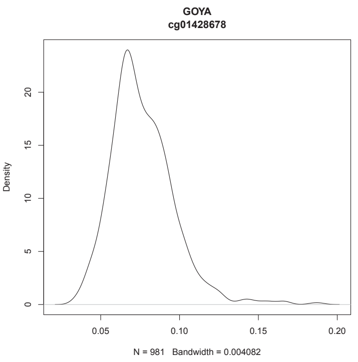
GOYA
cg01428678
Density
N = 981 Bandwidth = 0.004082

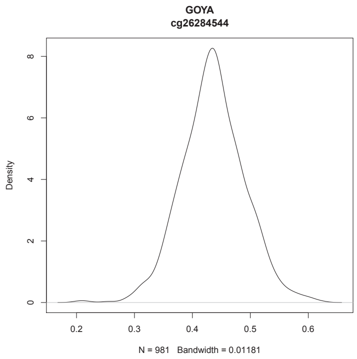
GOYA
cg26284544
Density
0
2
4
6
8
0.2
0.3
0.4
0.5
0.6
N = 981   Bandwidth = 0.01181

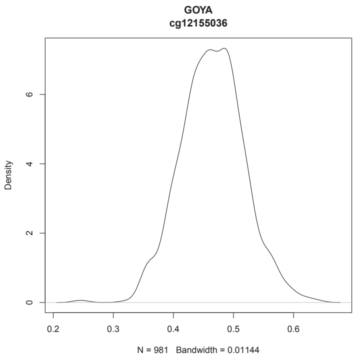
GOYA
cg12155036
Density
0
2
4
6
0.2
0.3
0.4
0.5
0.6
N = 981   Bandwidth = 0.01144

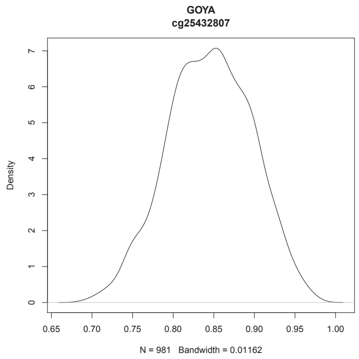
GOYA
cg25432807
Density
0
1
2
3
4
5
6
7
0.65
0.70
0.75
0.80
0.85
0.90
0.95
1.00
N = 981   Bandwidth = 0.01162

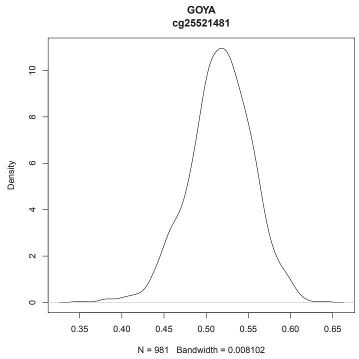
GOYA
cg25521481
Density
0
2
4
6
8
10
0.35
0.40
0.45
0.50
0.55
0.60
0.65
N = 981   Bandwidth = 0.008102

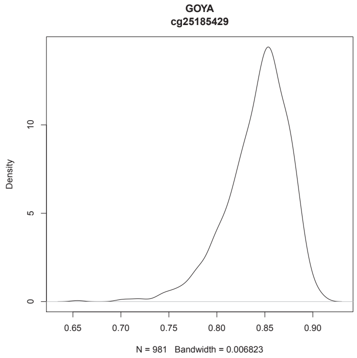
GOYA
cg25185429
Density
0
5
10
0.65
0.70
0.75
0.80
0.85
0.90
N = 981   Bandwidth = 0.006823

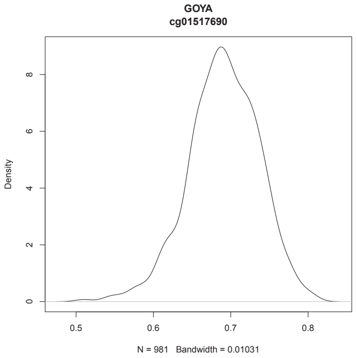
GOYA
cg01517690
Density
0
2
4
6
8
0.5
0.6
0.7
0.8
N = 981   Bandwidth = 0.01031
